# Supplementary material for: Efficient biosynthesis of heterodimeric C3-aryl pyrroloindoline alkaloids
Source: Nat Commun. 2018 Oct 24;9:4428. doi: 10.1038/s41467-018-06528-z (PMC6200733; doi:10.1038/s41467-018-06528-z)
Supplement: Supplementary file 1 — Supplementary Information [file 41467_2018_6528_MOESM1_ESM.pdf]

Supplementary Information

Efficient Biosynthesis of Heterodimeric C3-Aryl Pyrroloindoline Alkaloids

Tian *et al.*

Table of Contents

Supplementary Figures 1 – 42	1-70
Supplementary Tables 1 - 38	71-110
Supplementary Methods	111-126
General materials and methods	111
Heterologous expression of the NAS gene cluster	111
Fermentation and metabolites profile analysis	112
Cloning, expression and purification of P450 and associated proteins	112
Computational Methods	114
Construction of GB05-dir-T7	115
Construction of the whole cell catalysis system	115
Scaled biocatalytic reaction and purification of compounds NAS1-30	116
Synthesis and Identification of cyclodipeptide substrate	116
General methods for analyzing the structures of NASs	124
Bioactivity assay	125
Supplementary References	127

Supplementary Figures

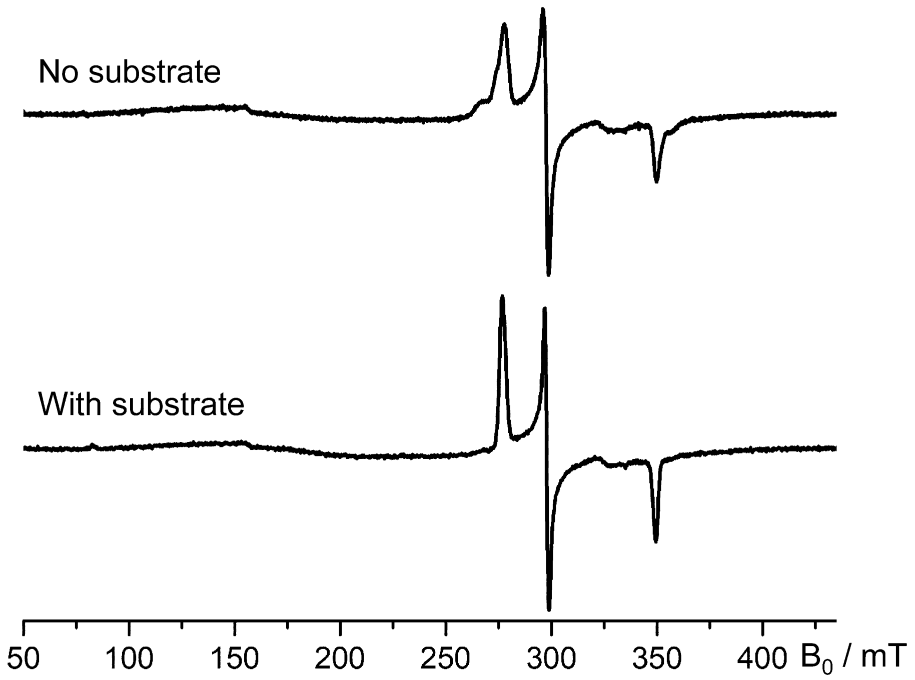

Supplementary Figure. 1. X-band (9.3810 GHz) CW EPR spectra analysis. EPR recorded at 15 K showing the high-spin and low-spin (LS) field range of ferric heme signal in the absence (top) and presence (bottom) of the substrate (2-fold excess of cW_L-P_L). The absence of any strong signals at low-field (< 250 mT), indicate that the ferric heme of the P450 is in the low-spin state and hence six coordinated. Data were recorded with a modulation amplitude of 0.5 mT, a modulation frequency of 100 KHz, with a microwave power of 2 mW.

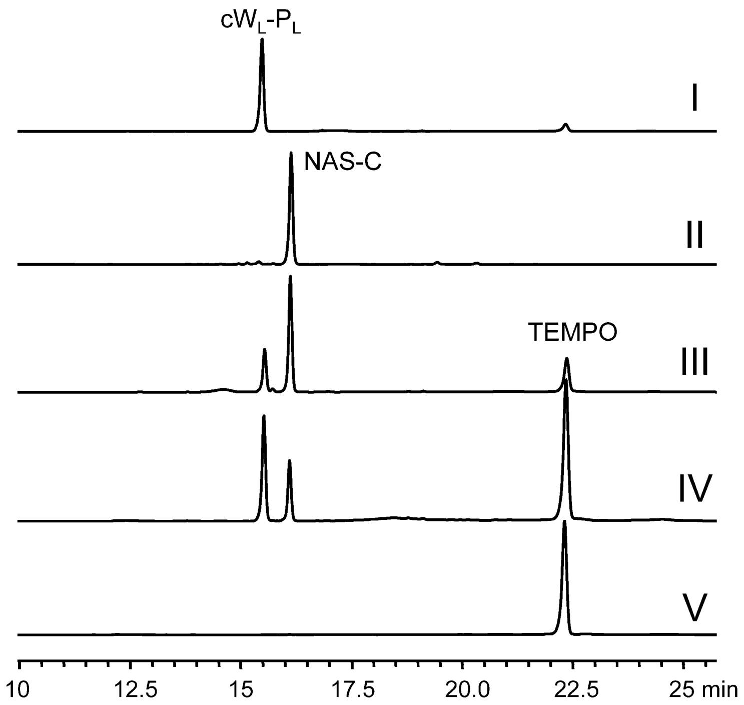

Supplementary Figure. 2. Inhibiting NascB activity by TEMPO. (I) Standard substrate cW_L-P_L; (II) NascB reaction, which can completely convert the substrate into the product NAS-C; (III) NascB reaction with 1 equiv. Of TEMPO (to cW_L-P_L); (IV) NascB reaction with 3 equiv. Of TEMPO; (V) Standard TEMPO.

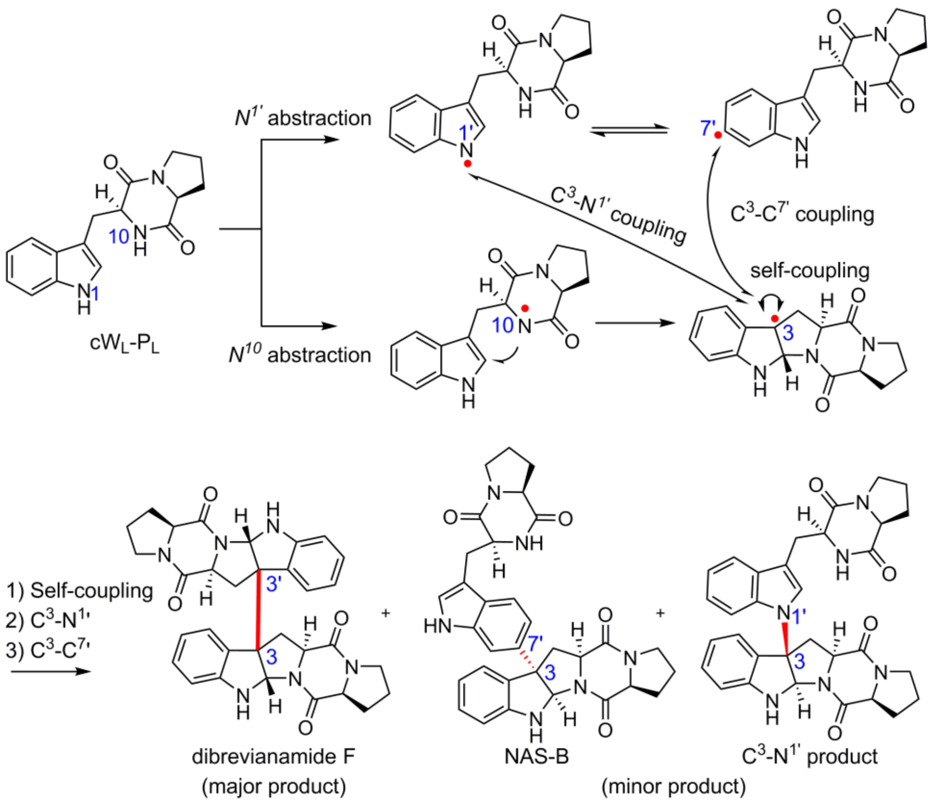

Supplementary Figure. 3. The proposed mechanism of the radical-mediated coupling reaction by DtpC[1,2] .

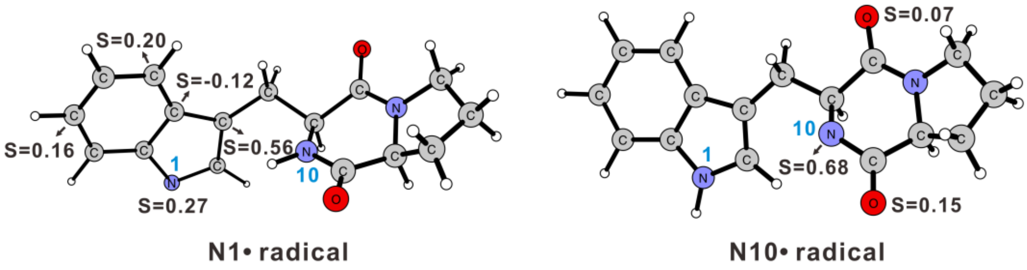

Supplementary Figure. 4. Optimized structures of the N^{1}• and N^{10}•. The unpaired spin population is shown with the indication of "S"

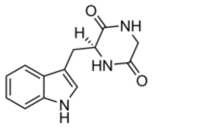

cW_L-G_L
s1

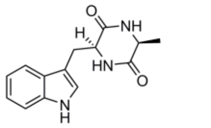

cW_L-A_L
s2

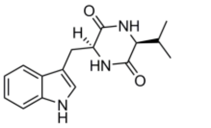

cW_L-V_L
s3

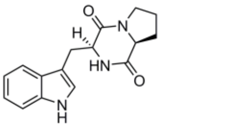

cW_L-P_L
s4

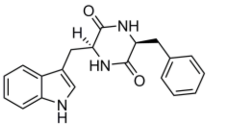

cW_L-F_L
s5

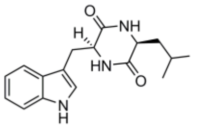

cW_L-L_L
s6

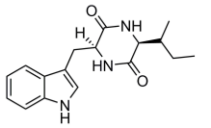

cW_L-I_L
s7

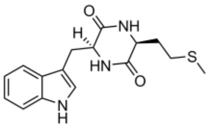

cW_L-M_L
s8

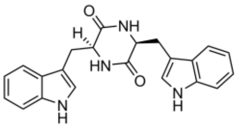

cW_L-W_L
s9

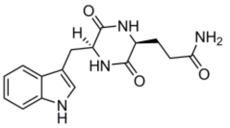

cW_L-Q_L
s10

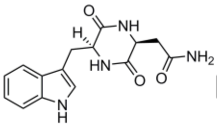

cW_L-N_L
s11

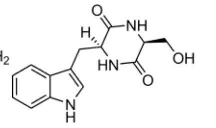

cW_L-S_L
s12

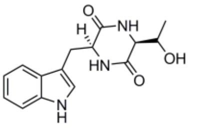

cW_L-T_L
s13

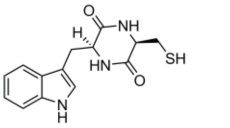

cW_L-C_L
s14

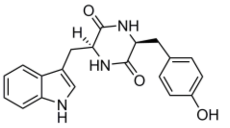

cW_L-Y_L
s15

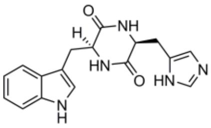

cW_L-H_L
s16

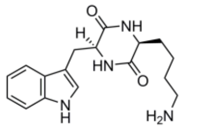

cW_L-K_L
s17

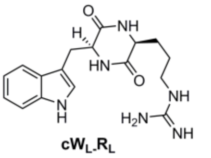

cW_L.R_L
s18

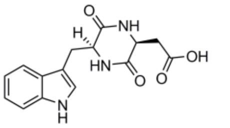

cW_L-D_L
s19

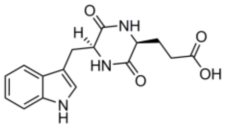

cW_L-E_L
s20

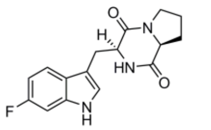

7-F-cW_L-P_L
s21

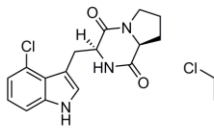

5-Cl-cW_L-P_L
s22

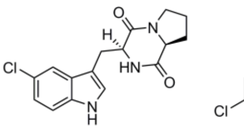

6-Cl-cW_L-P_L
s23

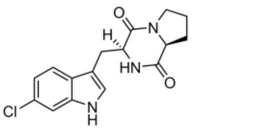

7-Cl-cW_L-P_L
s24

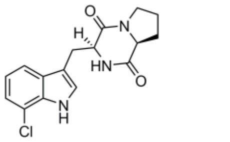

8-Cl-cW_L-P_L
s25

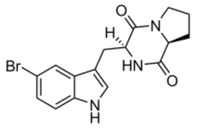

6-Br-cW_L-P_L
s26

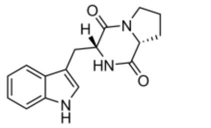

cW_D-P_D
s27

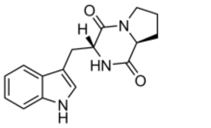

cW_D-P_L
s28

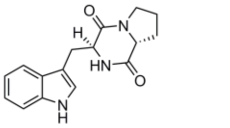

cW_L-P_D
s29

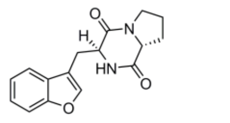

Oxo-cW_L-P_L
s30

Supplementary Figure. 5. Synthetic cyclodipeptide substrates

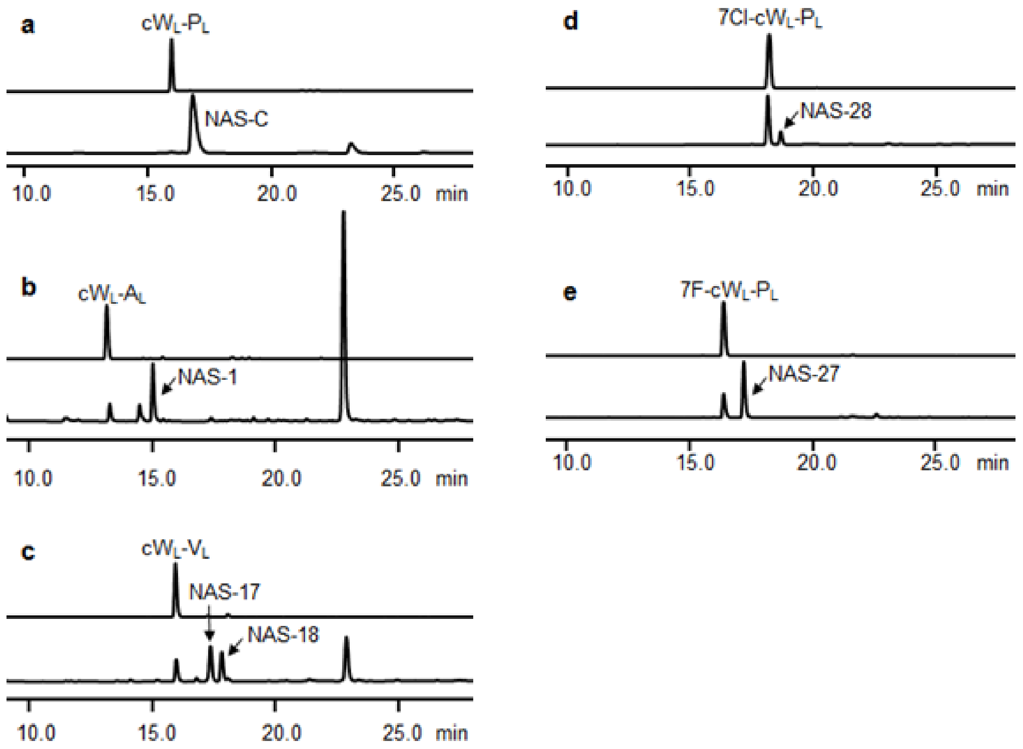

Supplementary Figure. 6. Self-dimerization of cyclodipeptides by whole-cell catalysis. The top trace is the HPLC profile of substrate while the bottom trace is that of catalysis system. The product profile in the whole cell catalysis is identical to the purified enzyme catalysis system. The substrates are cW_L-P_L (a), cW_L-A_L (b), cW_L-V_L (c), 7-Cl-cW_L-P_L (d) and 7F-cW_L-P_L (e).

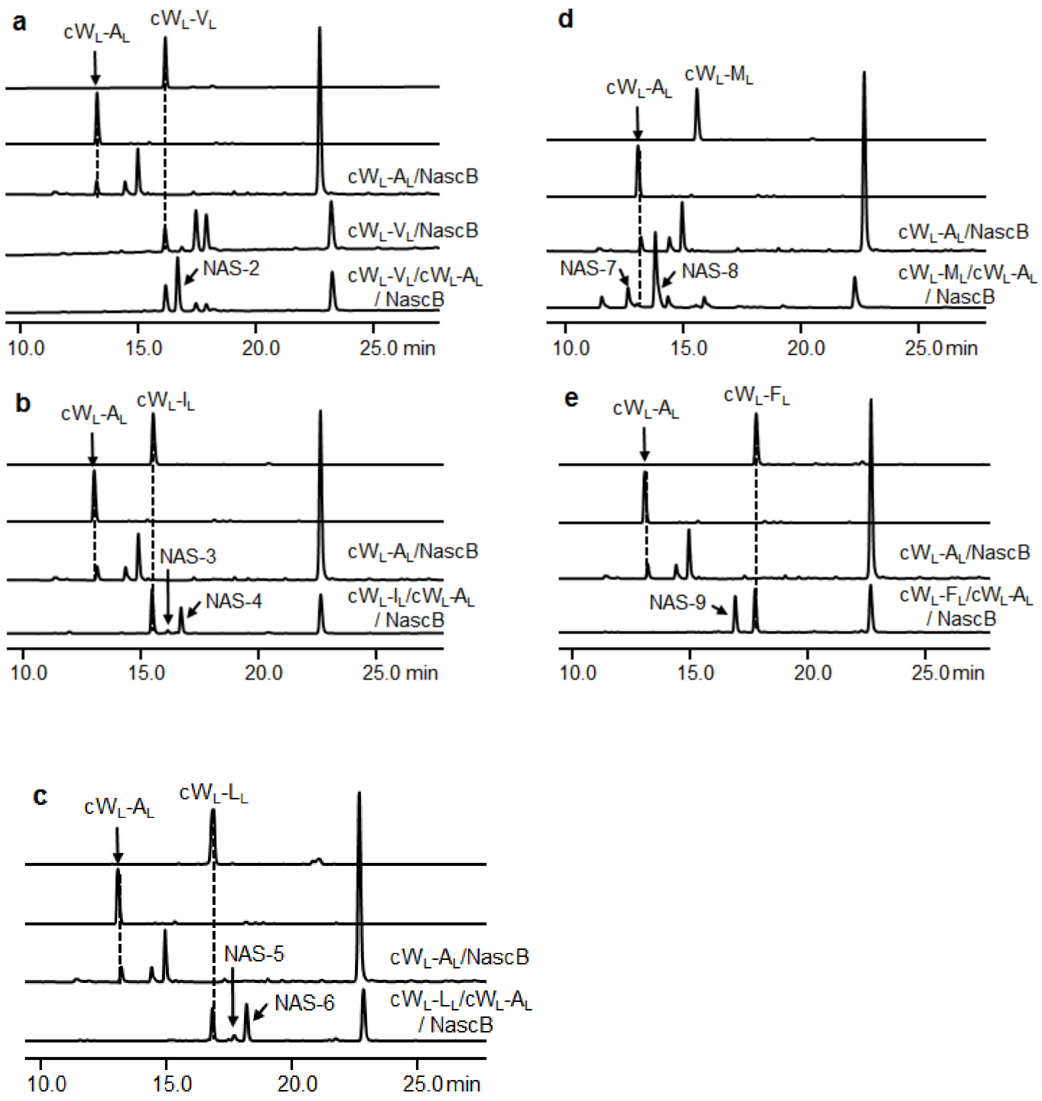

Supplementary Figure. 7. Dimerization of cW_L-A_L and other cyclodipeptides by whole-cell catalysis. The substrates are cW_L-A_L and cW_L-V_L (a), cW_L-A_L and cW_L-I_L (b), cW_L-A_L and cW_L-L_L (c), cW_L-A_L and cW_L-M_L (d) and cW_L-A_L and cW_L-F_L (e).

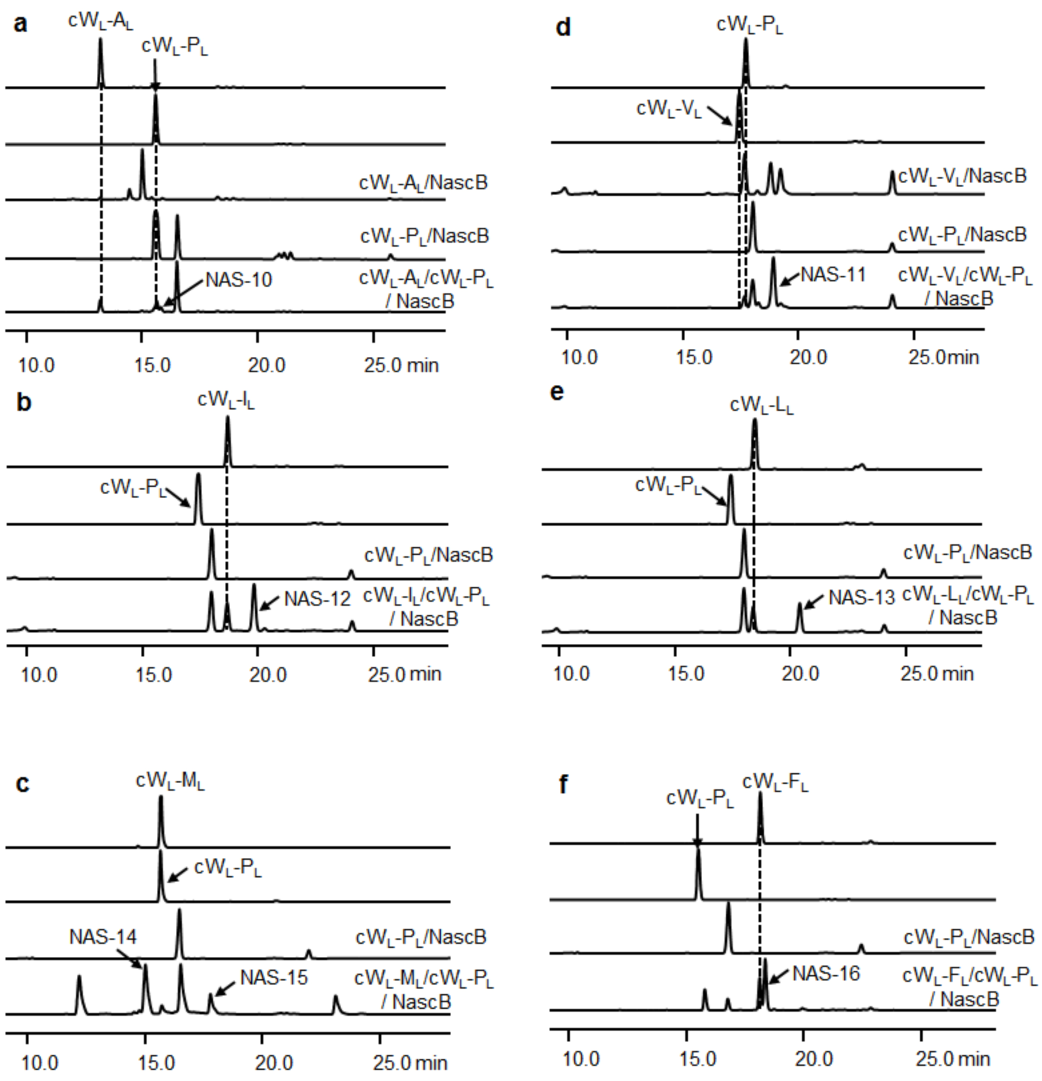

Supplementary Figure. 8. Dimerization of cW$_L$-P$_L$ and other cyclodipeptides by whole-cell catalysis. The substrates are cW$_L$-P$_L$ and cW$_L$-A$_L$ (a), cW$_L$-P$_L$ and cW$_L$-I$_L$ (b), cW$_L$-P$_L$ and cW$_L$-M$_L$ (c), cW$_L$-P$_L$ and cW$_L$-V$_L$ (d), cW$_L$-P$_L$ and cW$_L$-L$_L$ (e) and cW$_L$-P$_L$ and cW$_L$-F$_L$ (f).

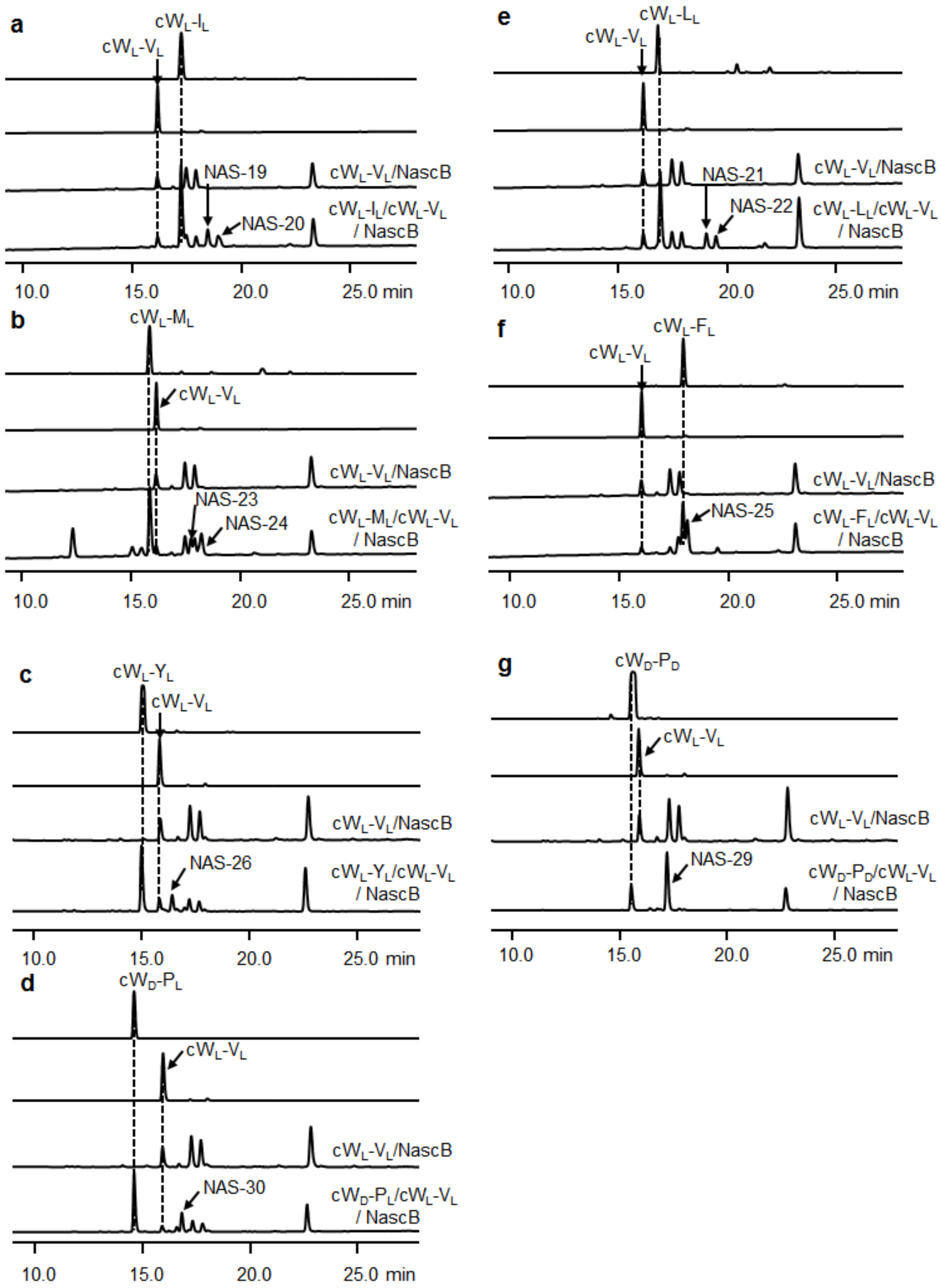

Supplementary Figure. 9. Dimerization of cW_L-V_L and other cyclodipeptides by whole-cell catalysis. The substrates are cW_L-V_L and cW_L-I_L (a), cW_L-V_L and cW_L-M_L (b), cW_L-V_L and cW_L-Y_L (c), cW_L-V_L and cW_D-P_L (d), cW_L-V_L and cW_L-L_L (e), cW_L-V_L and cW_L-F_L (f) and cW_L-V_L and cW_D-P_D.

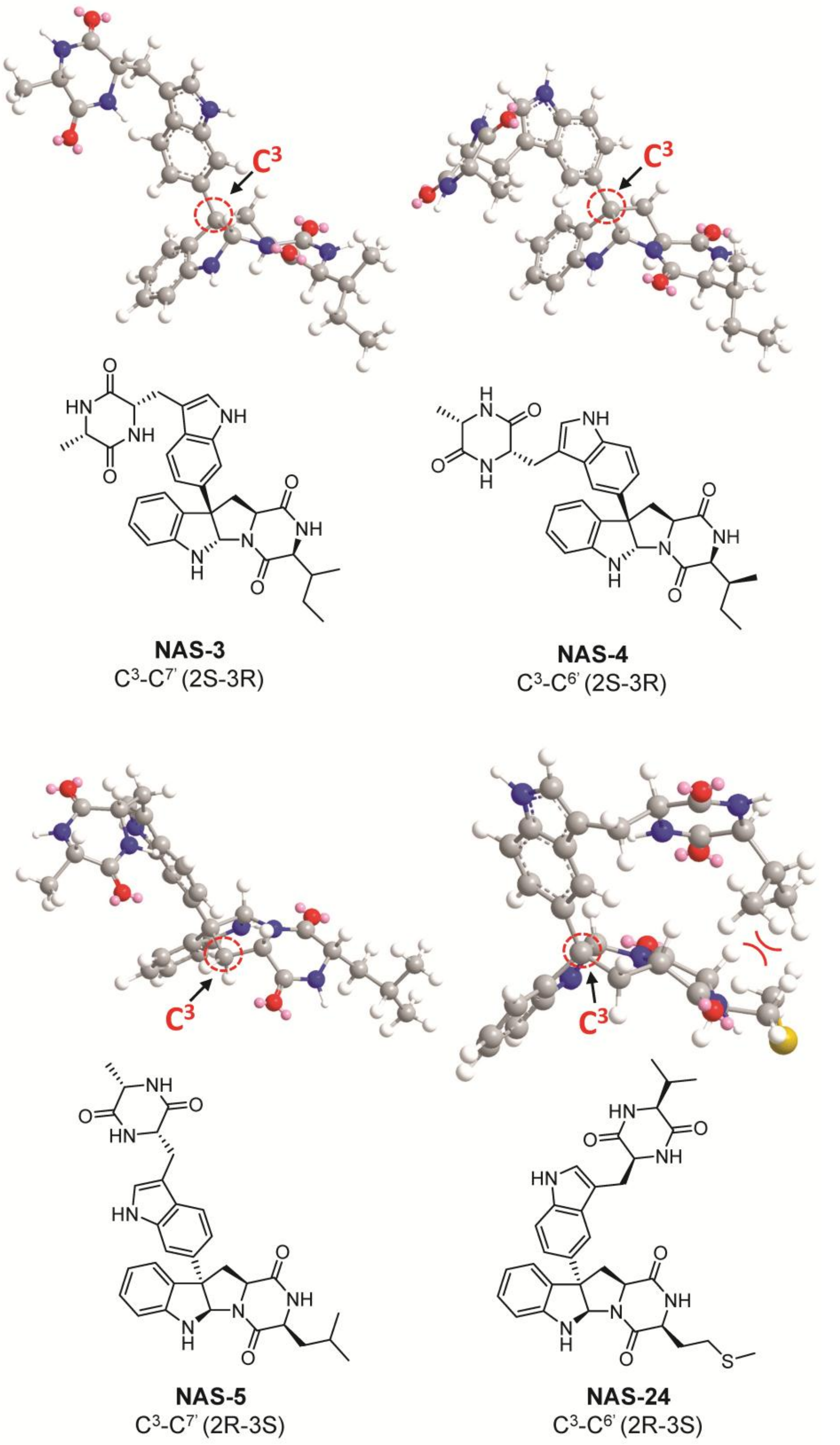

Supplementary Figure. 10. Conformation analysis of different types of NAS products. The stereo-conformations were simulated by molecular dynamics using an MM2 force field.

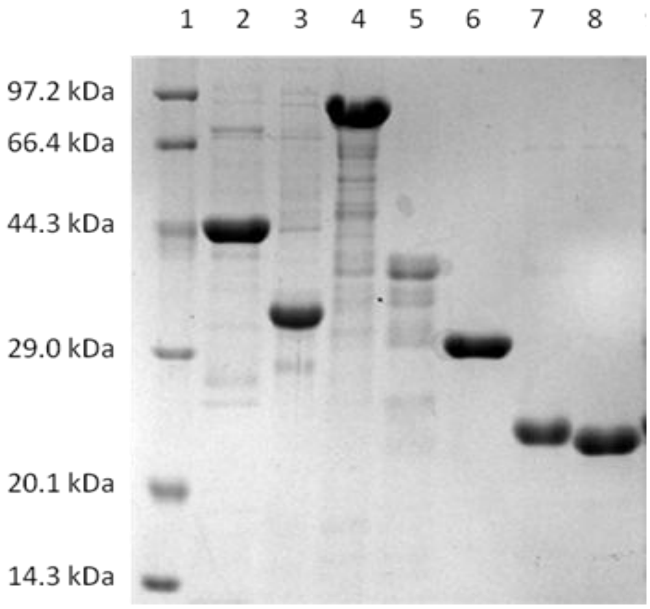

Supplementary Figure. 11. 12 % SDS-PAGE analysis of the recombinant proteins. Lane 1: Protein ladder; Lane 2: NascB, 44 kDa; Lane 3: GDH, 30.6 kDa; Lane 4: *Spinach* MBP-Fdr, 80.3 kDa; Lane 5: *Spinach* TRX-Fd, 31.3 kDa; Lane 6: *E. coli* flavodoxin reductase, 29.9 kDa; Lane 7: *E. coli* flavodoxin A, 21.9 kDa; Lane 8: *E. coli* flavodoxin B, 21.8 kDa.

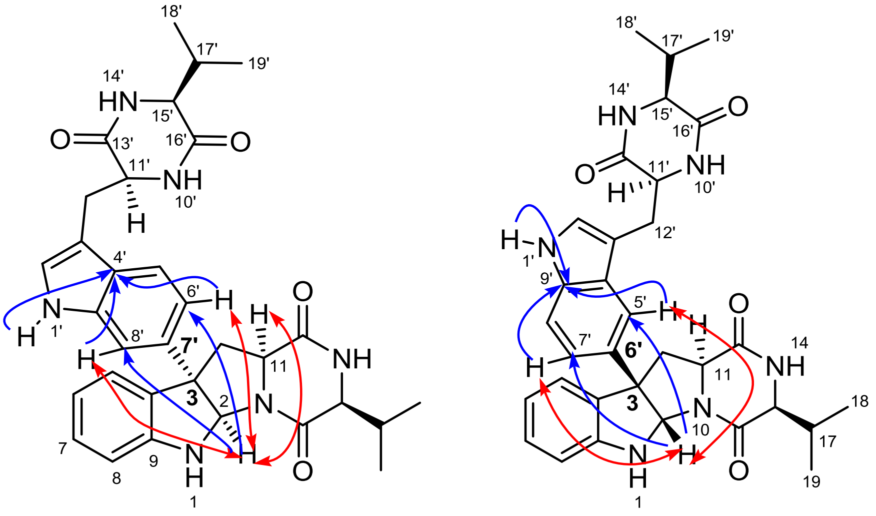

NAS-17 **NAS-18**

Supplementary Figure. 12. Key HMBC and ROESY correlations observed in NAS-17 and NAS-18. Detail analysis please see Supplementary Materials and Methods1.9 (HMBC: Blue; ROESY: Red).

a

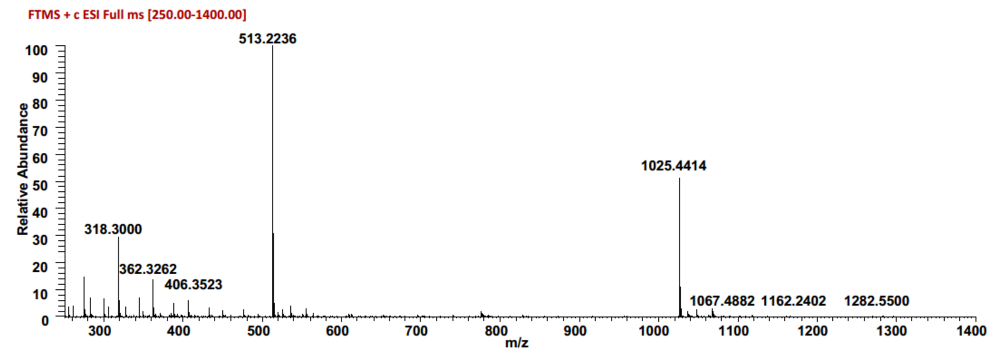

FTMS + c ESI Full ms [250.00-1400.00]
Relative Abundance
m/z
513.2236
1025.4414
318.3000
362.3262
406.3523
1067.4882
1162.2402
1282.5500

b

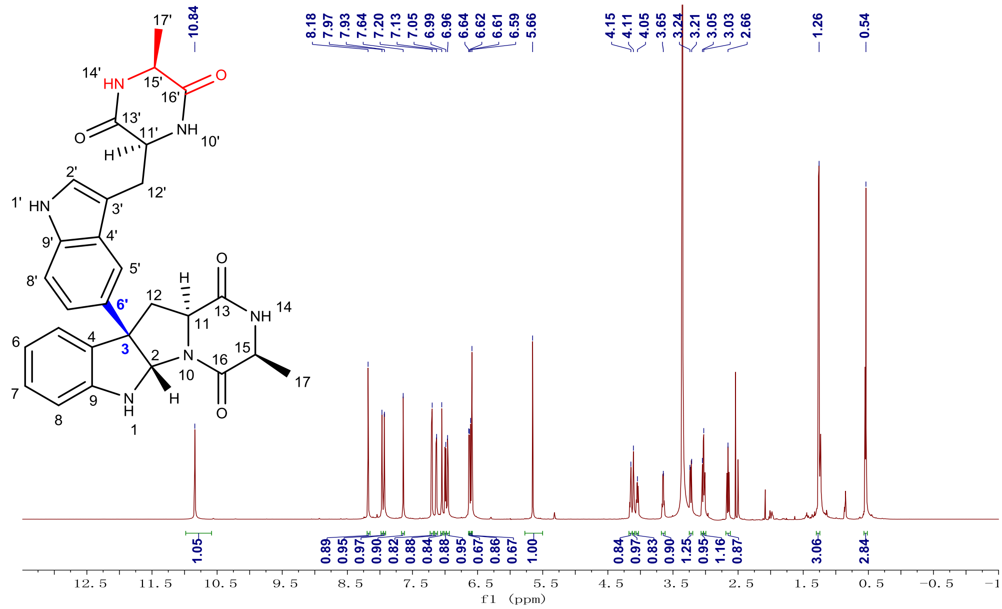

10.84
8.18
7.97
7.93
7.64
7.20
7.13
7.05
6.99
6.96
6.64
6.62
6.61
6.59
5.66
4.15
4.11
4.05
3.65
3.24
3.21
3.05
3.03
2.66
1.26
0.54
1.05
0.89
0.95
0.97
0.90
0.82
0.88
0.84
0.88
0.95
0.67
0.86
0.67
1.00
0.84
0.97
0.83
0.90
1.25
0.95
1.16
0.87
3.06
2.84
f1 (ppm)

c

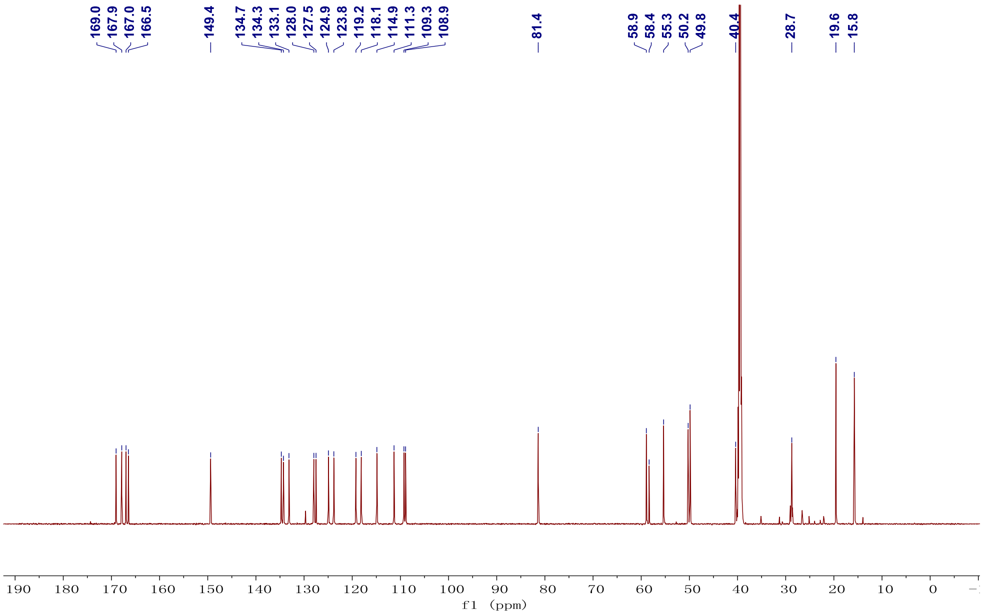

169.0
167.9
167.0
166.5
149.4
134.7
134.3
133.1
128.0
127.5
124.9
123.8
119.2
118.1
114.9
111.3
109.3
108.9
81.4
58.9
58.4
55.3
50.2
49.8
40.4
28.7
19.6
15.8
f1 (ppm)

d

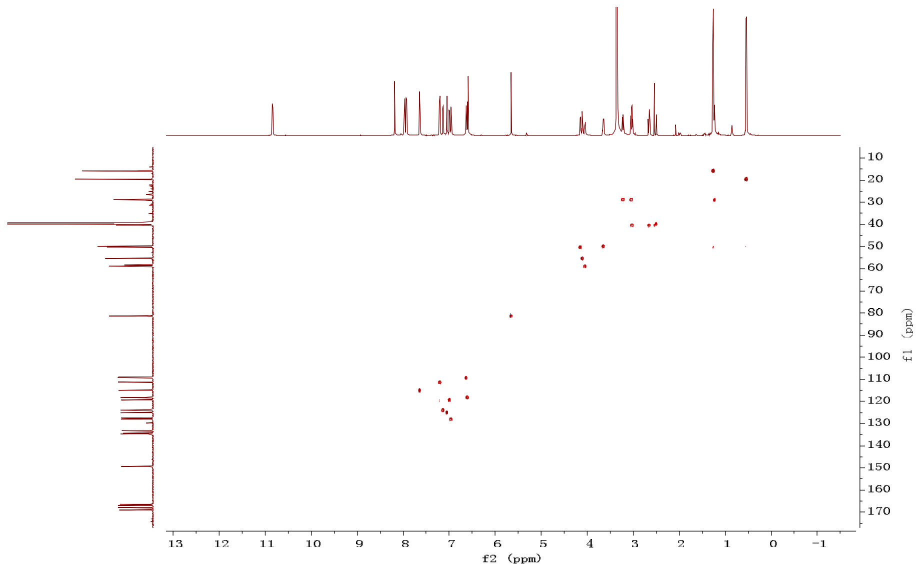

e

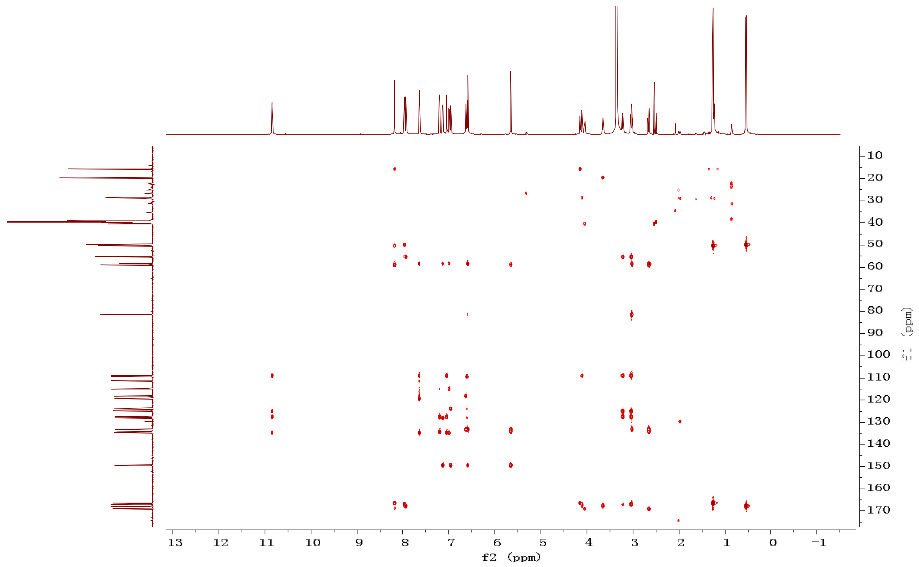

f

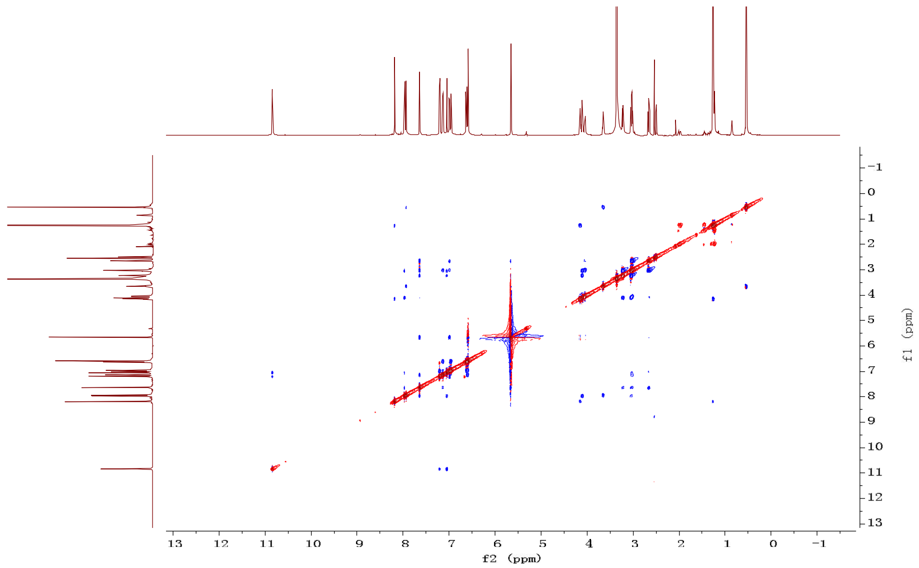

Supplementary Figure. 13. Spectra of compound NAS-1 (a) HRESIMS of compound of NAS-1 (b) ^{1}H NMR of compound of NAS-1 (c) ^{13}C NMR of compound of NAS-1 (d) HSQC of compound of NAS-1 (e) HMBC of compound of NAS-1 (f) ROSEY of compound of NAS-1

a

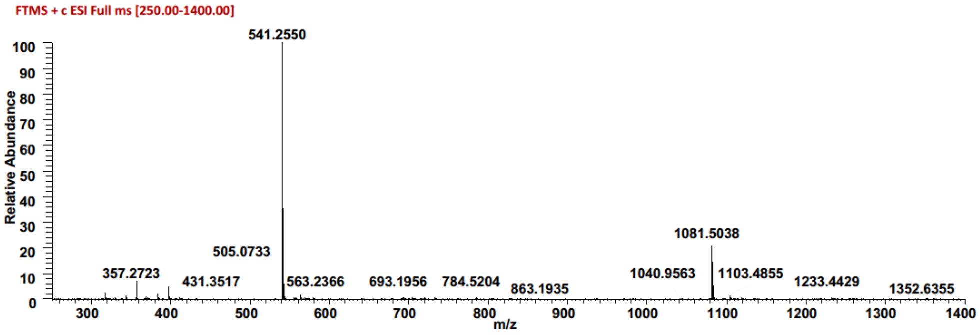

FTMS + c ESI Full ms [250.00-1400.00]
541.2550
357.2723
431.3517
505.0733
563.2366
693.1956
784.5204
863.1935
1040.9563
1081.5038
1103.4855
1233.4429
1352.6355
Relative Abundance
m/z

b

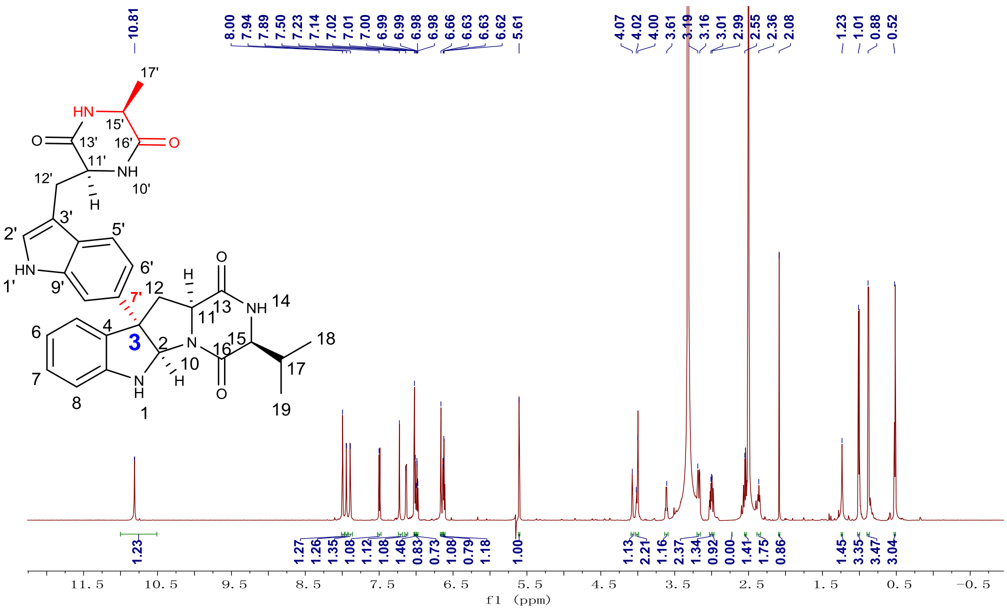

10.81
8.00
7.94
7.89
7.50
7.23
7.14
7.02
7.01
7.00
6.99
6.98
6.98
6.66
6.63
6.63
6.62
5.61
4.07
4.02
4.00
3.61
3.19
3.16
3.01
2.99
2.55
2.36
2.08
1.23
1.01
0.88
0.52
f1 (ppm)

c

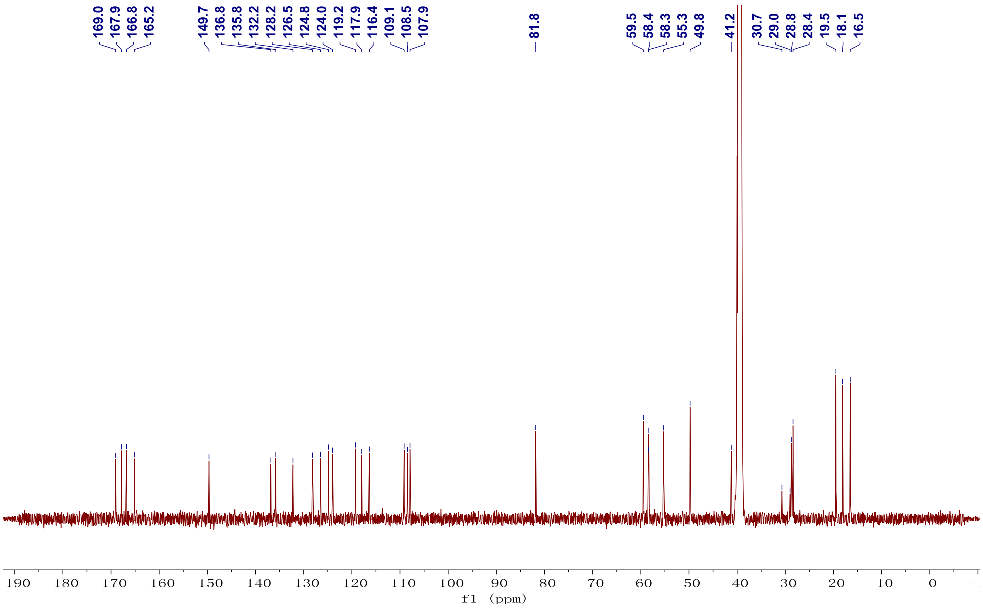

169.0
167.9
166.8
165.2
149.7
136.8
135.8
132.2
128.2
126.5
124.8
124.0
119.2
117.9
116.4
109.1
108.5
107.9
81.8
59.5
58.4
58.3
55.3
49.8
41.2
30.7
29.0
28.8
28.4
19.5
18.1
16.5
f1 (ppm)

d

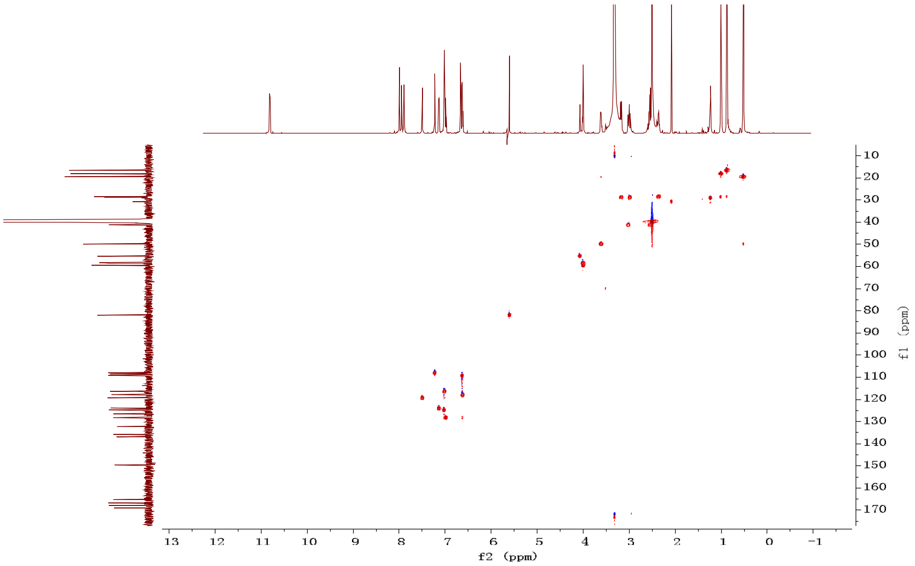

e

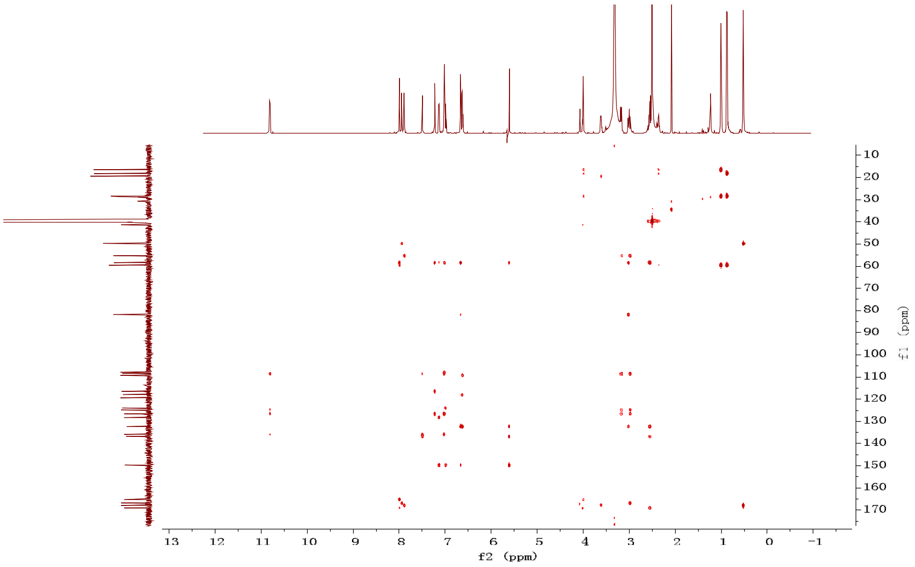

f

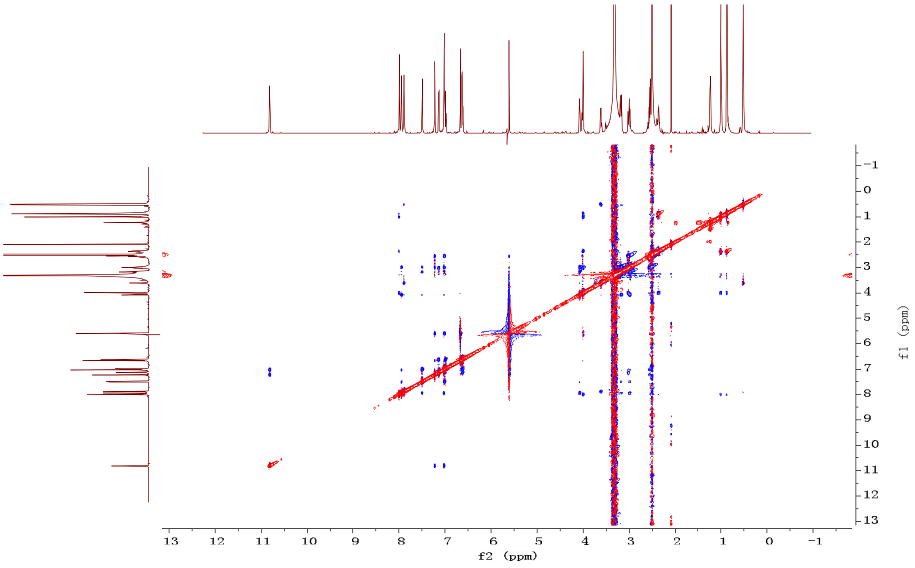

Supplementary Figure. 14. Spectra of compound NAS-2 (a) HRESIMS of compound of NAS-2 (b) ^{1}H NMR of compound of NAS-2 (c) ^{13}C NMR of compound of NAS-2 (d) HSQC of compound of NAS-2 (e) HMBC of compound of NAS-2 (f) ROSEY of compound of NAS-2

a

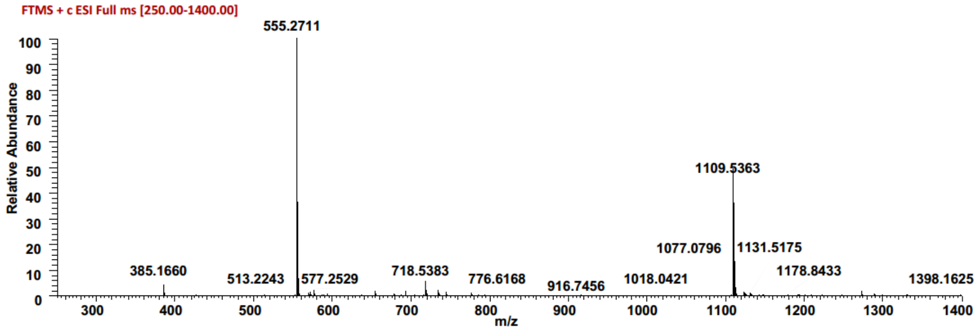
FTMS + c ESI Full ms [250.00-1400.00]
555.2711
1109.5363
1077.0796
1131.5175
385.1660
513.2243
577.2529
718.5383
776.6168
916.7456
1018.0421
1178.8433
1398.1625
Relative Abundance
m/z

b

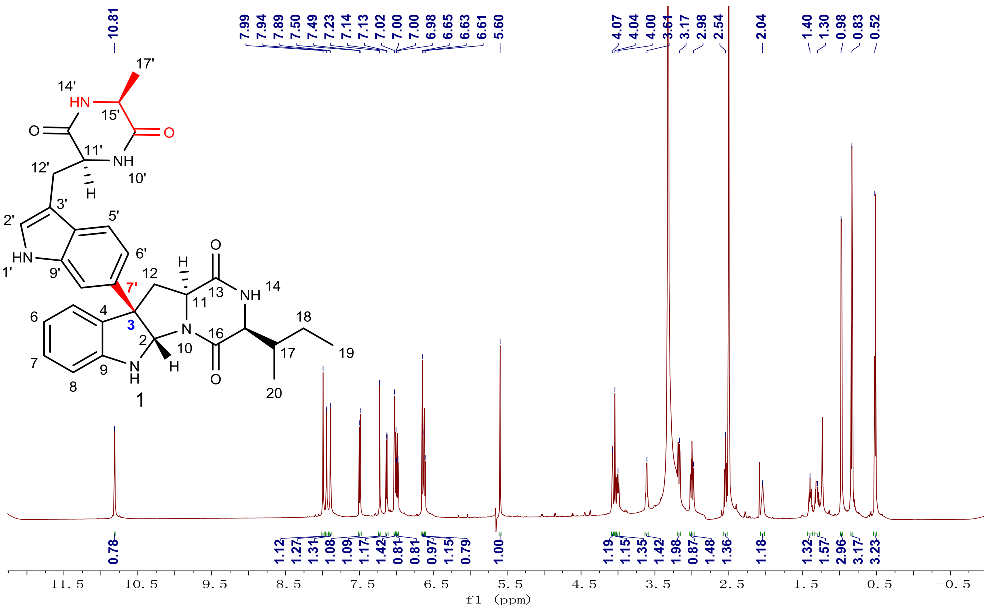
10.81
7.99 7.94 7.89 7.50 7.49 7.23 7.14 7.13 7.02 7.00 7.00 6.98 6.65 6.63 6.61 5.60
4.07 4.04 4.00 3.61 3.17 2.98 2.54 2.04 1.40 1.30 0.98 0.83 0.52
0.78 1.12 1.27 1.31 1.08 1.09 1.17 1.42 0.81 0.81 0.97 1.15 0.79 1.00
1.19 1.15 1.35 1.42 1.98 0.87 1.48 1.36 1.18 1.32 1.57 2.96 3.17 3.23
f1 (ppm)
1

c

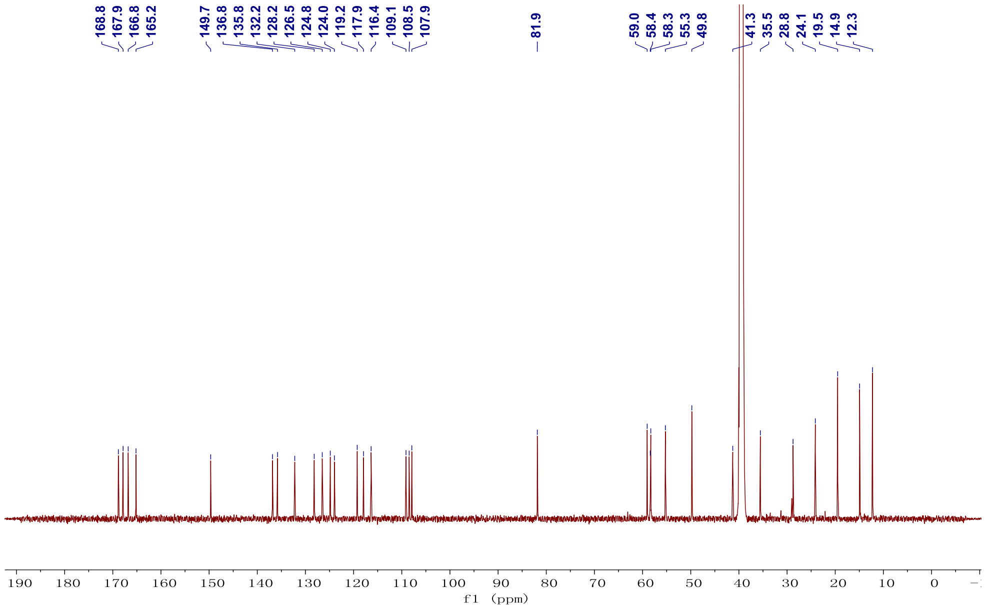
168.8 167.9 166.8 165.2
149.7 136.8 135.8 132.2 128.2 126.5 124.8 124.0 119.2 117.9 116.4 109.1 108.5 107.9
81.9
59.0 58.4 58.3 55.3 49.8
41.3 35.5 28.8 24.1 19.5 14.9 12.3
f1 (ppm)

d

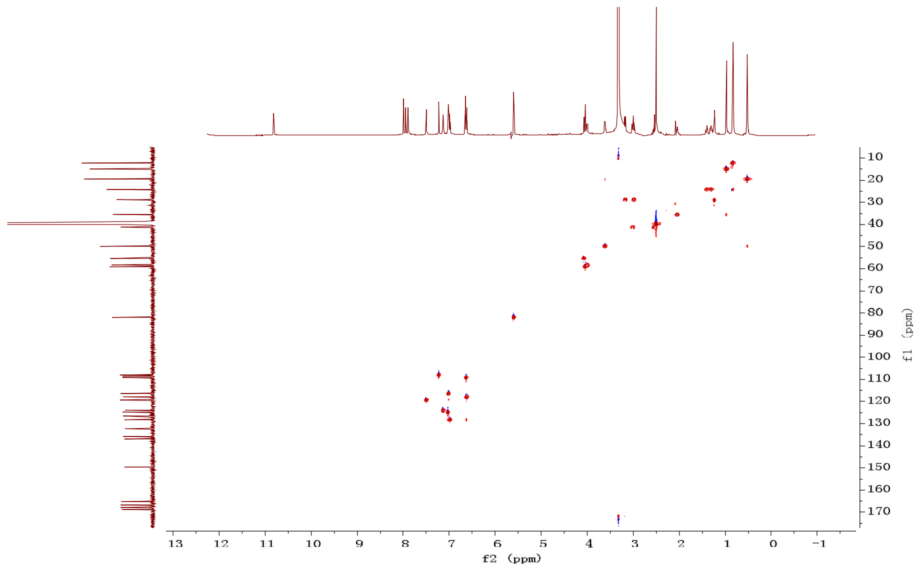

e

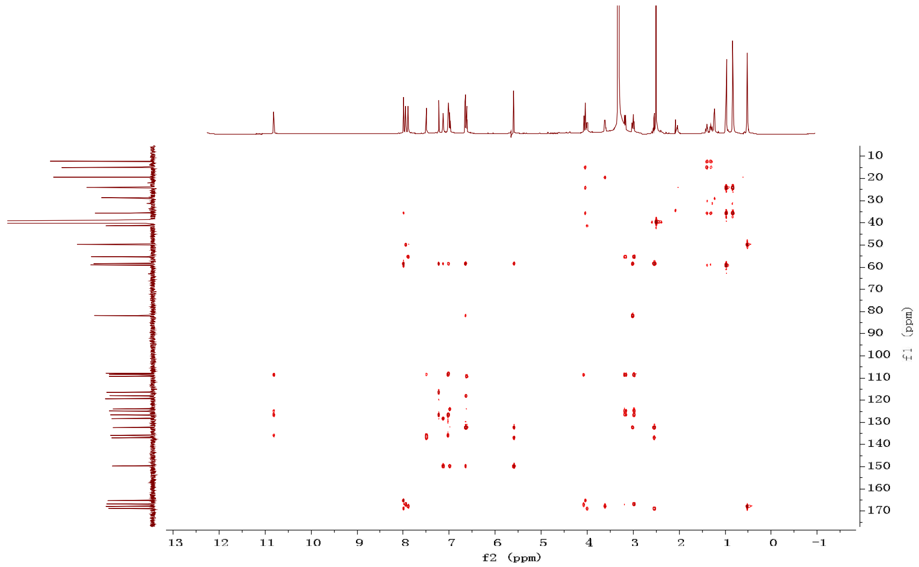

f

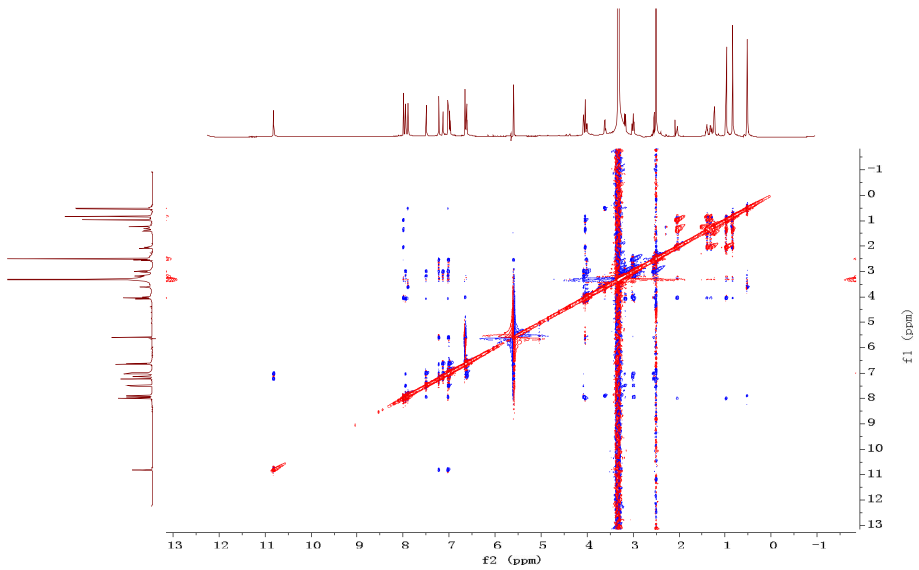

Supplementary Figure. 15. Spectra of compound NAS-3 (a) HRESIMS of compound of NAS-3 (b) ^{1}H NMR of compound of NAS-3 (c) ^{13}C NMR of compound of NAS-3 (d) HSQC of compound of NAS-3(e) HMBC of compound of NAS-3 (f) ROSEY of compound of NAS-3

a

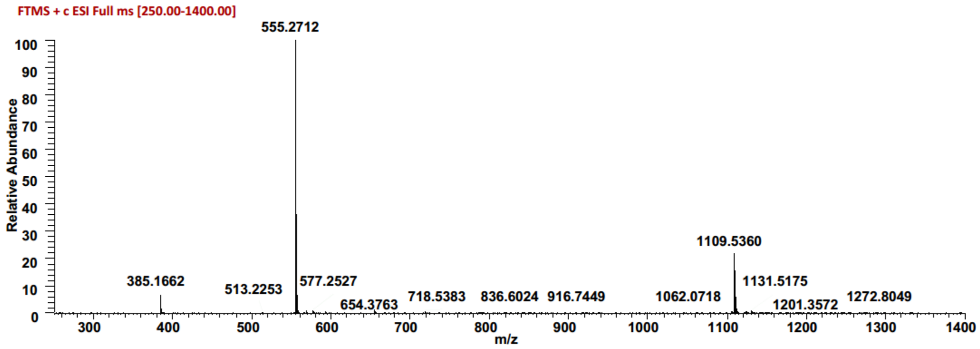
FTMS + c ESI Full ms [250.00-1400.00]
555.2712
Relative Abundance
385.1662
513.2253
577.2527
654.3763
718.5383
836.6024
916.7449
1062.0718
1109.5360
1131.5175
1201.3572
1272.8049
m/z

b

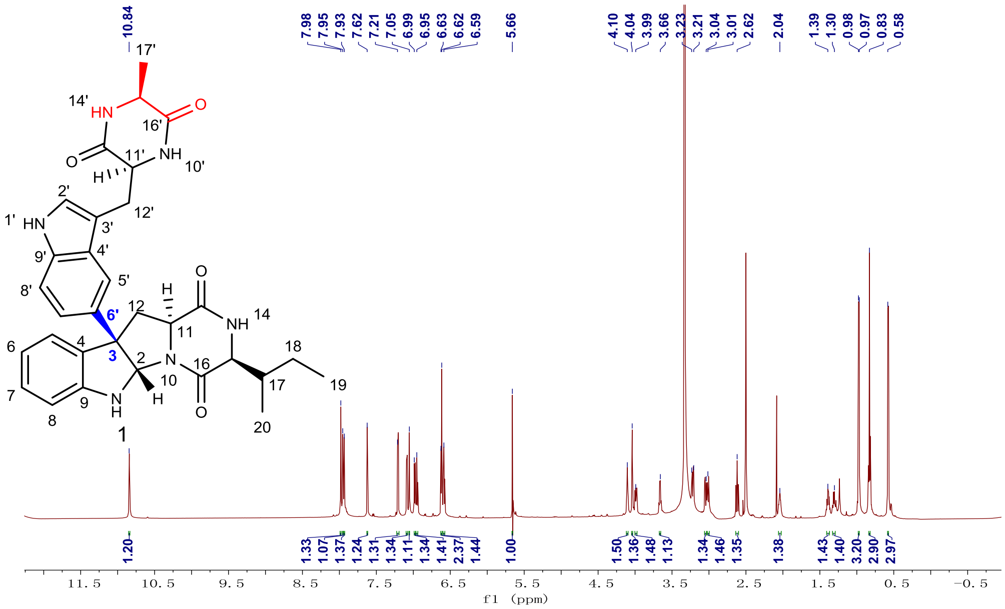
10.84
7.98 7.95 7.93 7.62 7.21 7.05 6.99 6.95 6.63 6.62 6.59
5.66
4.10 4.04 3.99 3.66 3.23 3.21 3.04 3.01 2.62
2.04
1.39 1.30 0.98 0.97 0.83 0.58
1.20
1.33 1.07 1.37 1.24 1.31 1.34 1.11 1.34 1.41 2.37 1.44
1.00
1.50 1.36 1.48 1.13
1.34 1.46 1.35
1.38
1.43 1.40 3.20 2.90 2.97
f1 (ppm)
1

c

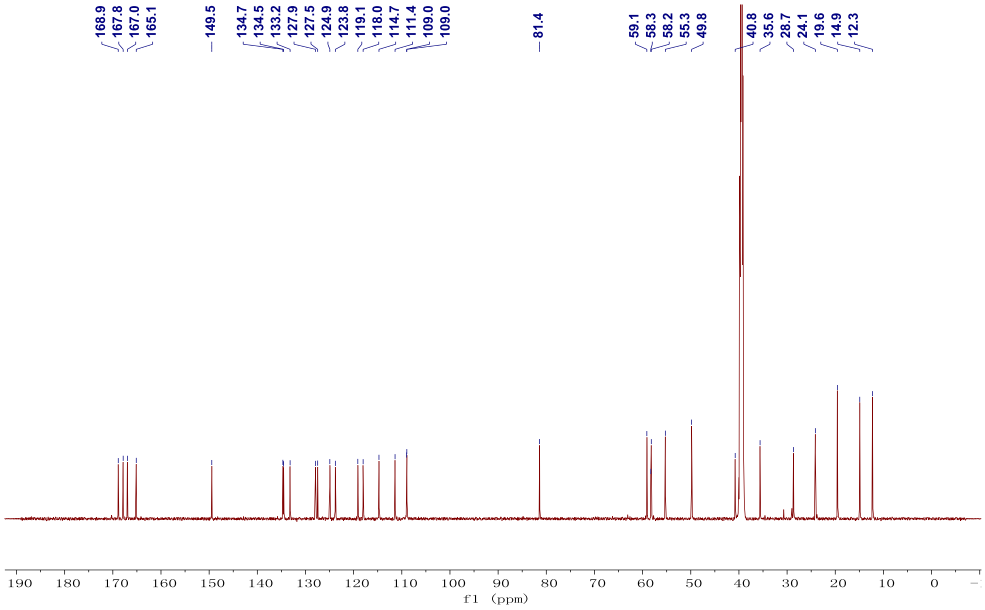
168.9 167.8 167.0 165.1
149.5
134.7 134.5 133.2 127.9 127.5 124.9 123.8 119.1 118.0 114.7 111.4 109.0 109.0
81.4
59.1 58.3 58.2 55.3 49.8
40.8 35.6 28.7 24.1 19.6 14.9 12.3
f1 (ppm)

d

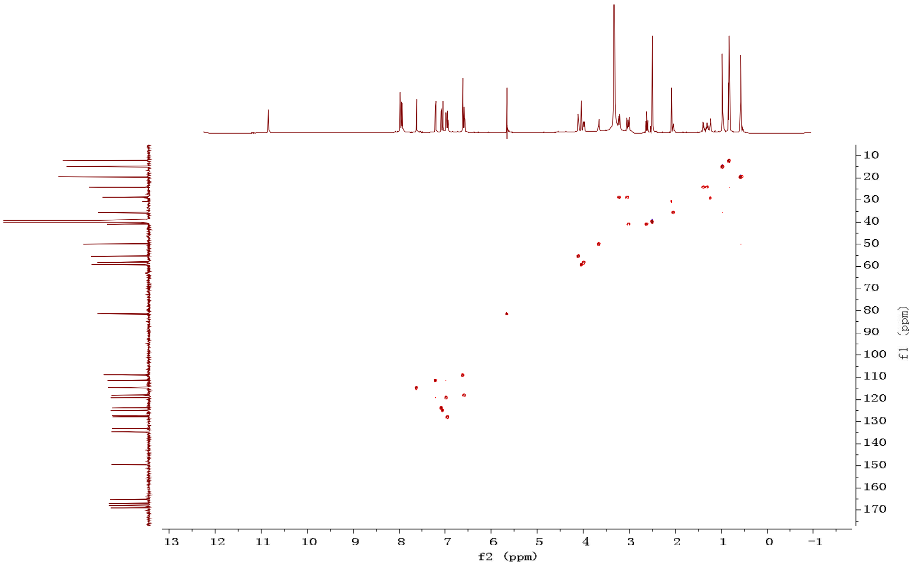

e

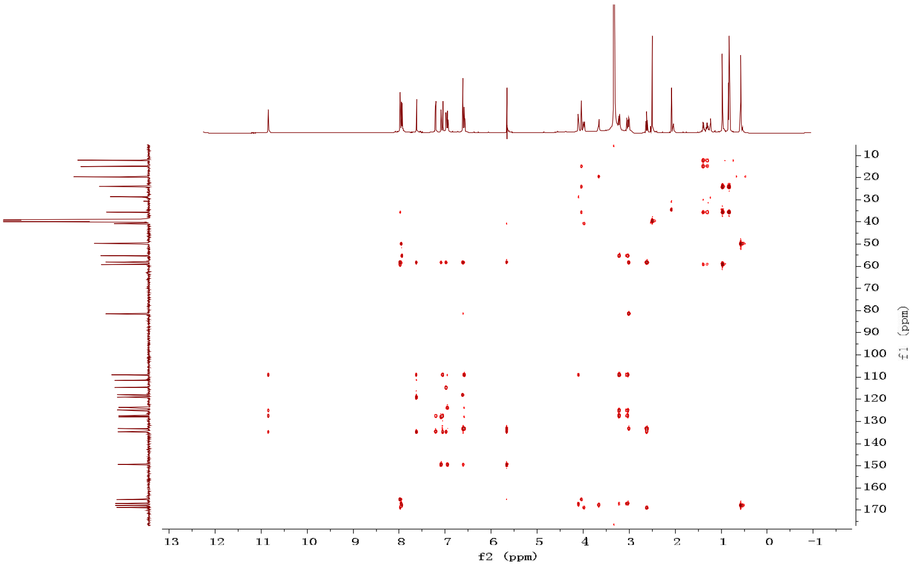

f

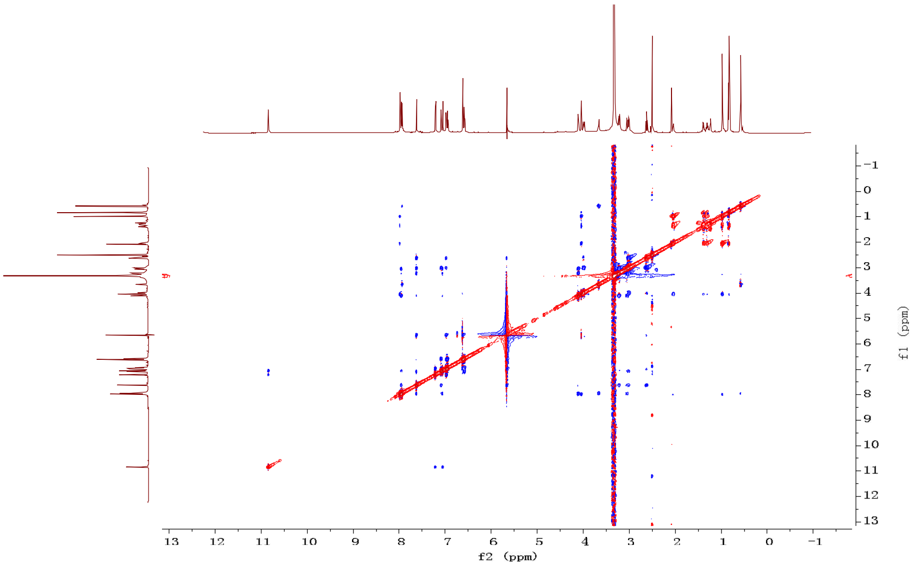

Supplementary Figure. 16. Spectra of compound NAS-4 (a) HRESIMS of compound of NAS-4 (b) ^{1}H NMR of compound of NAS-4 (c) ^{13}C NMR of compound of NAS-4 (d) HSQC of compound of NAS-4(e) HMBC of compound of NAS-4 (f) ROSEY of compound of NAS-4

a

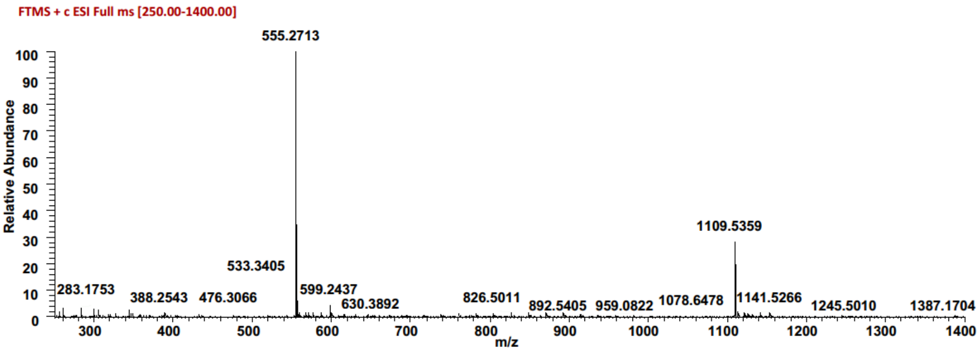

FTMS + c ESI Full ms [250.00-1400.00]
555.2713
1109.5359
533.3405
283.1753
388.2543
476.3066
599.2437
630.3892
826.5011
892.5405
959.0822
1078.6478
1141.5266
1245.5010
1387.1704
Relative Abundance
m/z

b

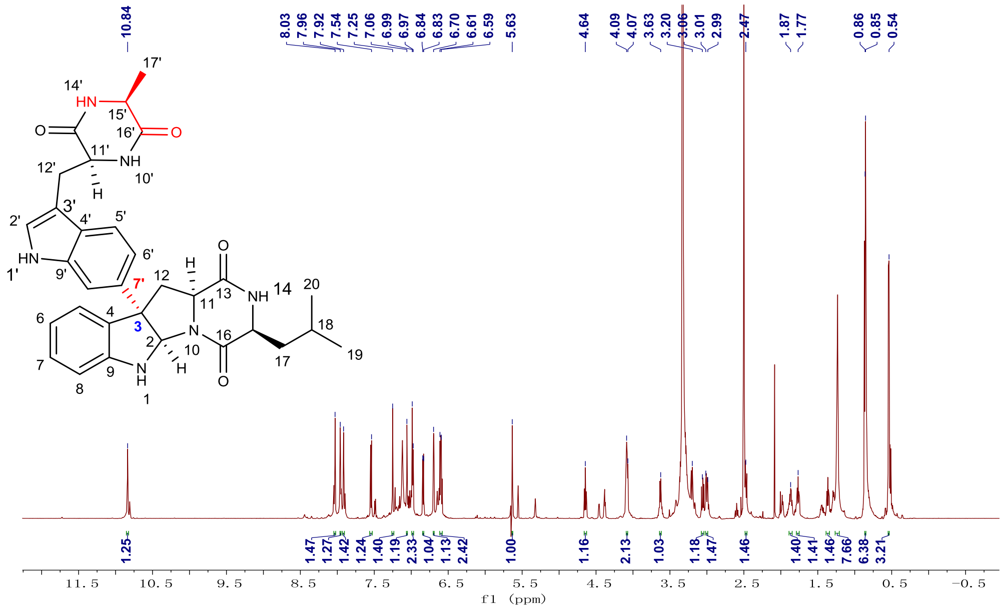

10.84
8.03 7.96 7.92 7.54 7.25 7.06 6.99 6.97 6.84 6.83 6.70 6.61 6.59
5.63
4.64
4.09 4.07 3.63 3.20 3.06 3.01 2.99
2.47
1.87 1.77
0.86 0.85 0.54
1.25 1.47 1.27 1.42 1.24 1.40 1.19 2.33 1.04 1.13 2.42 1.00 1.16 2.13 1.03 1.18 1.47 1.46 1.40 1.41 1.46 7.66 6.38 3.21
f1 (ppm)

c

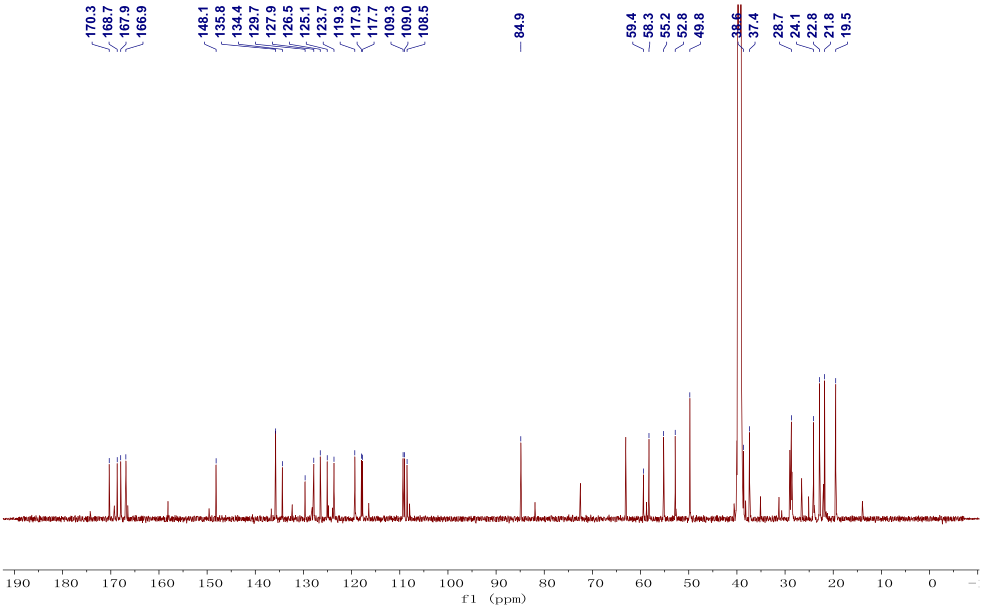

170.3 168.7 167.9 166.9
148.1 135.8 134.4 129.7 127.9 126.5 125.1 123.7 119.3 117.9 117.7 109.3 109.0 108.5
84.9
59.4 58.3 55.2 52.8 49.8
38.6 37.4 28.7 24.1 22.8 21.8 19.5
f1 (ppm)

d

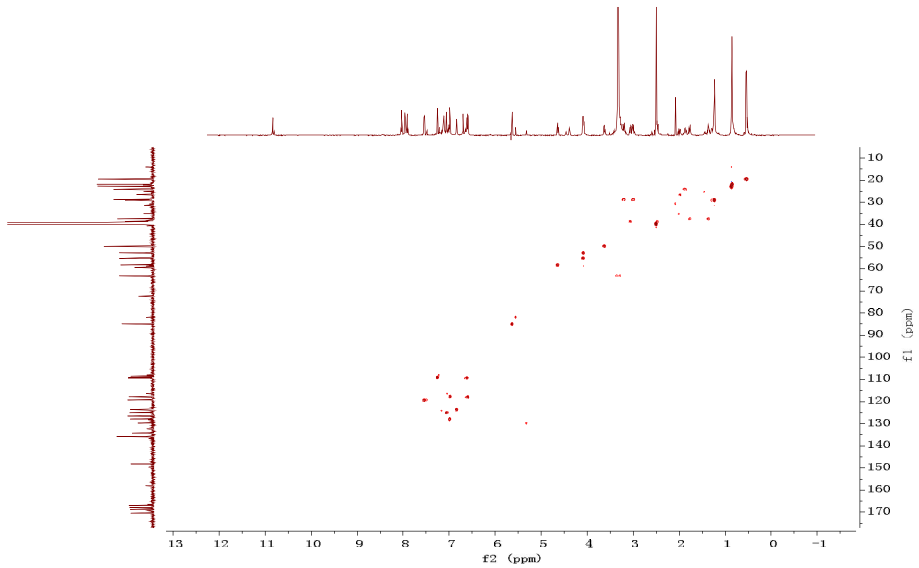

e

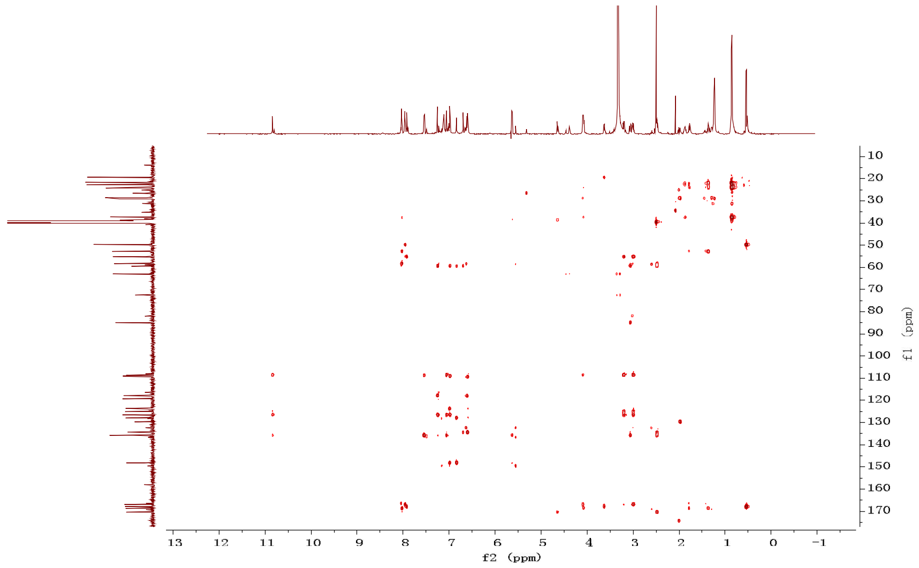

f

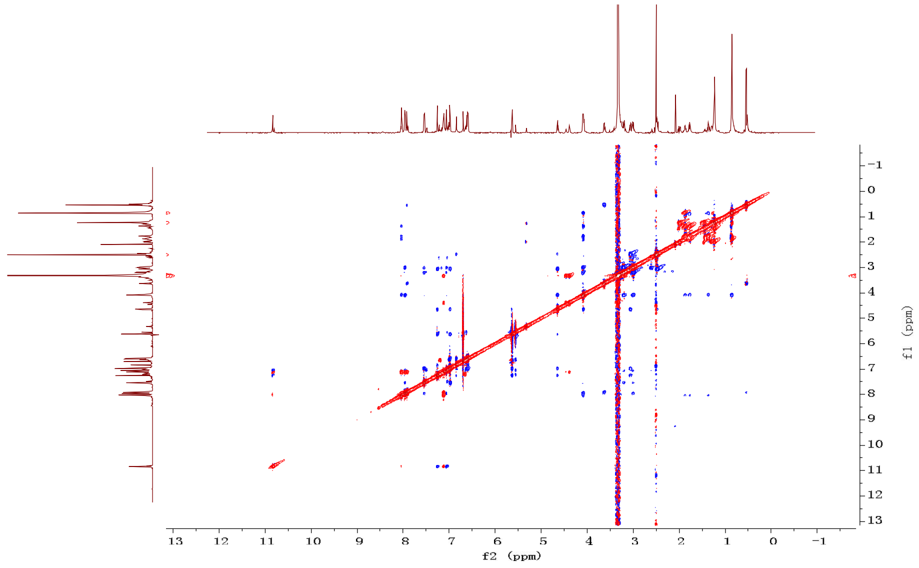

Supplementary Figure. 17. Spectra of compound NAS-5 (a) HRESIMS of compound of NAS-5 (b) ^{1}H NMR of compound of NAS-5 (c) ^{13}C NMR of compound of NAS-5 (d) HSQC of compound of NAS-5 (e) HMBC of compound of NAS-5 (f) ROSEY of compound of NAS-5

a

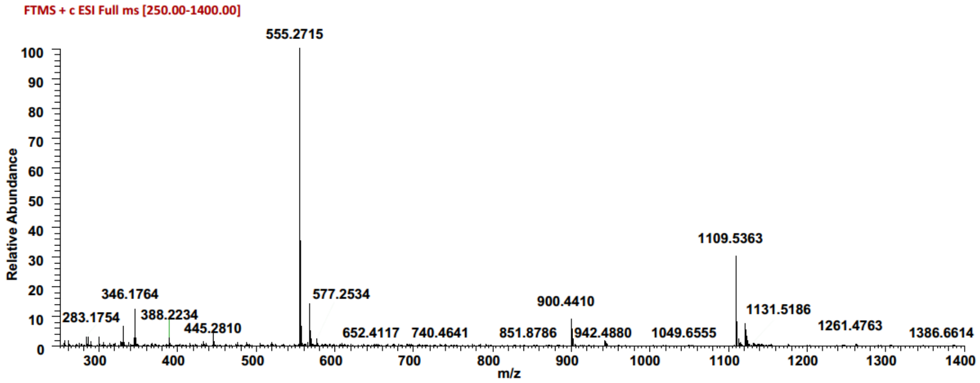

FTMS + c ESI Full ms [250.00-1400.00]
555.2715
1109.5363
346.1764
577.2534
900.4410
1131.5186
283.1754
388.2234
445.2810
652.4117
740.4641
851.8786
942.4880
1049.6555
1261.4763
1386.6614
Relative Abundance
m/z

b

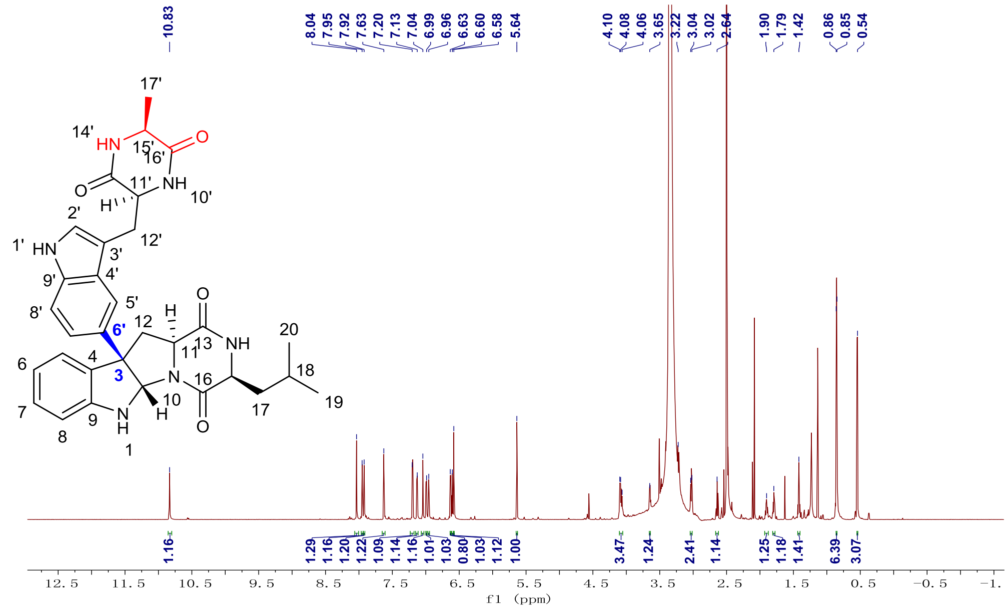

10.83
8.04 7.95 7.92 7.63 7.20 7.13 7.04 6.99 6.96 6.63 6.60 6.58
5.64
4.10 4.08 4.06 3.65 3.22 3.04 3.02 2.64
1.90 1.79 1.42 0.86 0.85 0.54
1.16
1.29 1.16 1.20 1.22 1.09 1.14 1.16 1.01 1.03 0.80 1.03 1.12 1.00
3.47 1.24 2.41 1.14 1.25 1.18 1.41 6.39 3.07
f1 (ppm)

c

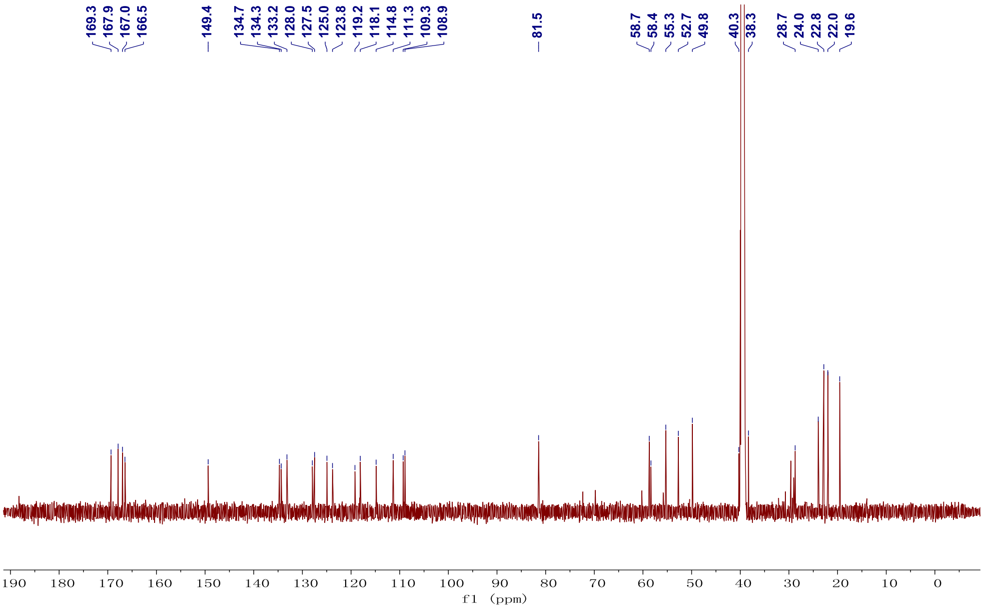

169.3 167.9 167.0 166.5
149.4
134.7 134.3 133.2 128.0 127.5 125.0 123.8 119.2 118.1 114.8 111.3 109.3 108.9
81.5
58.7 58.4 55.3 52.7 49.8
40.3 38.3
28.7 24.0 22.8 22.0 19.6
f1 (ppm)

d

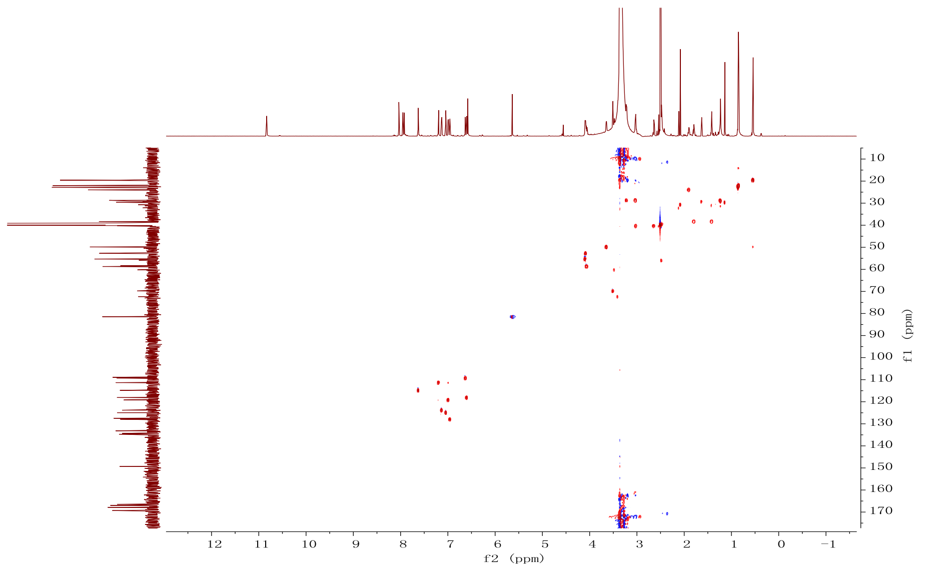

e

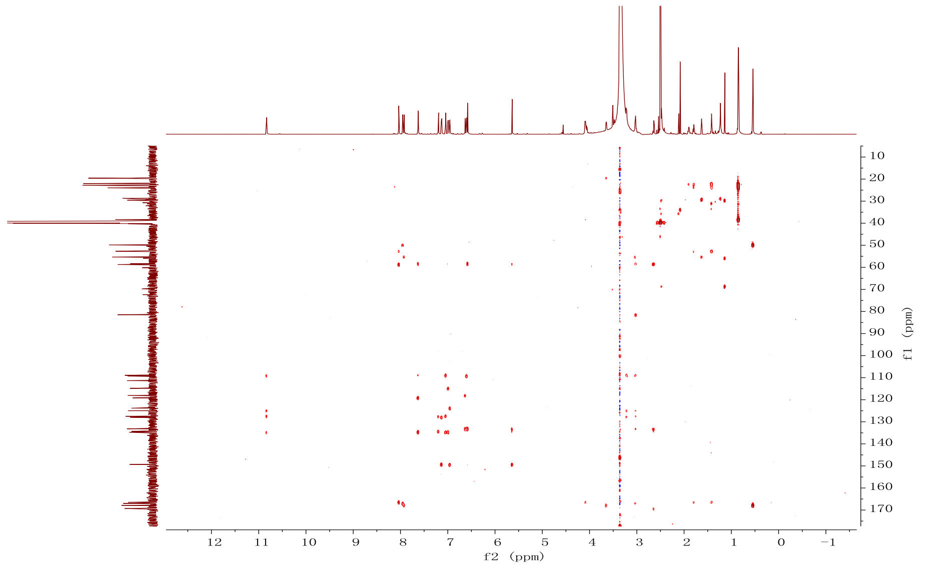

f

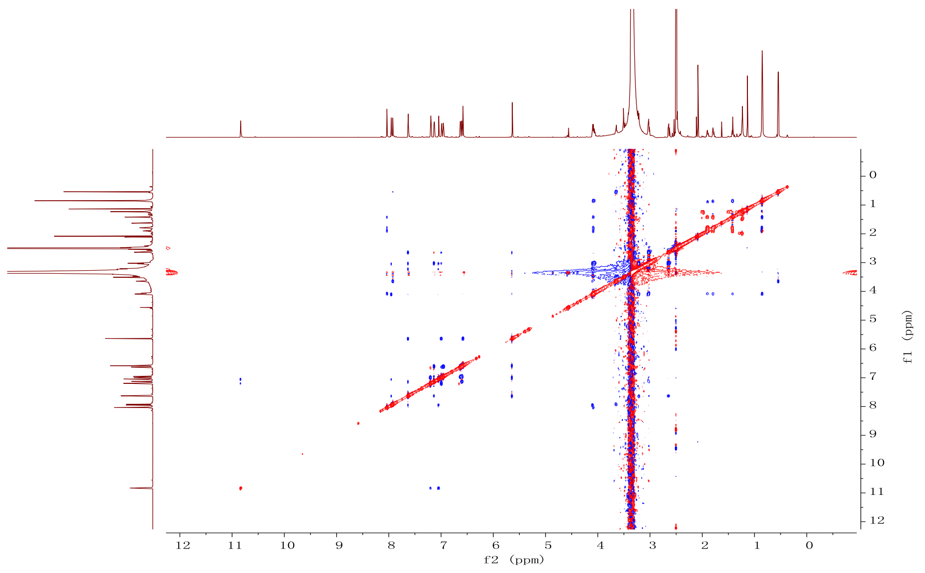

Supplementary Figure. 18. Spectra of compound NAS-6 (a) HRESIMS of compound of NAS-6 (b) ^{1}H NMR of compound of NAS-6 (c) ^{13}C NMR of compound of NAS-6 (d) HSQC of compound of NAS-6 (e) HMBC of compound of NAS-6 (f) ROSEY of compound of NAS-6

a

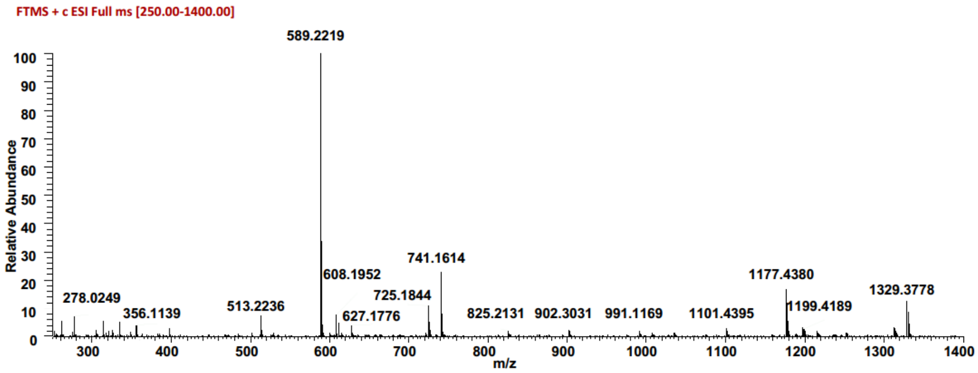

b

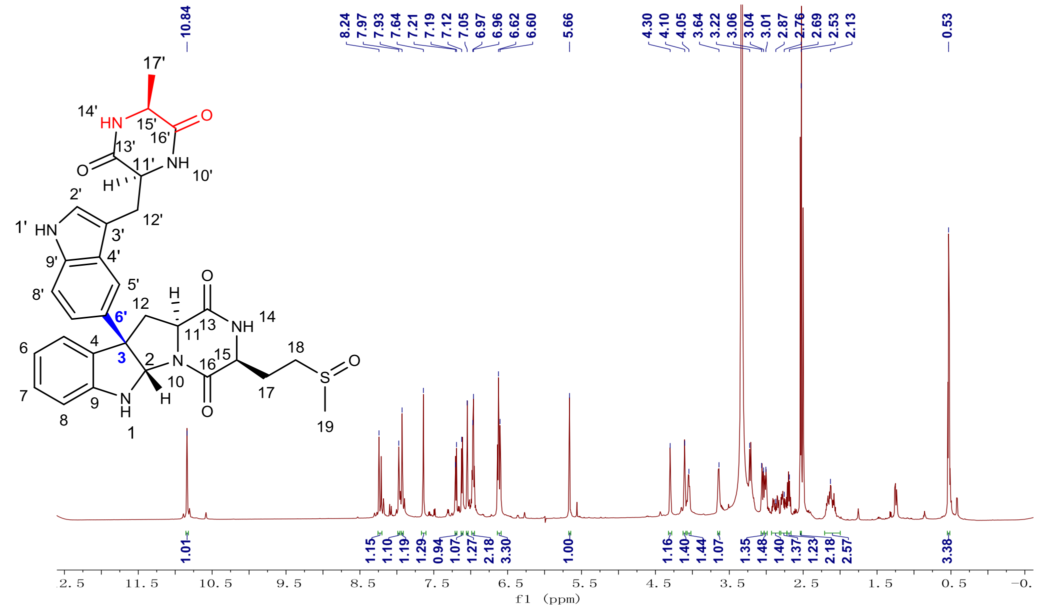

c

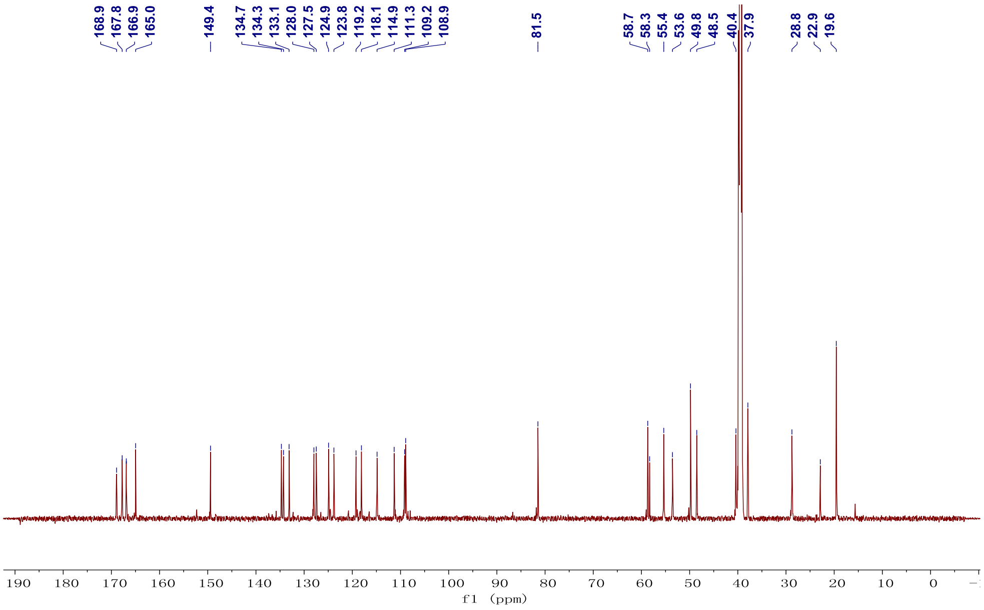

d

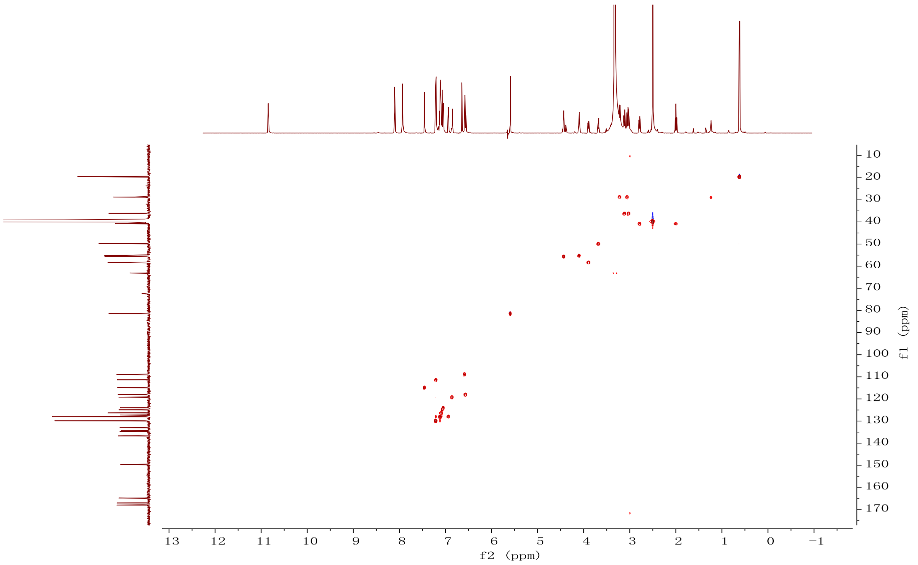

e

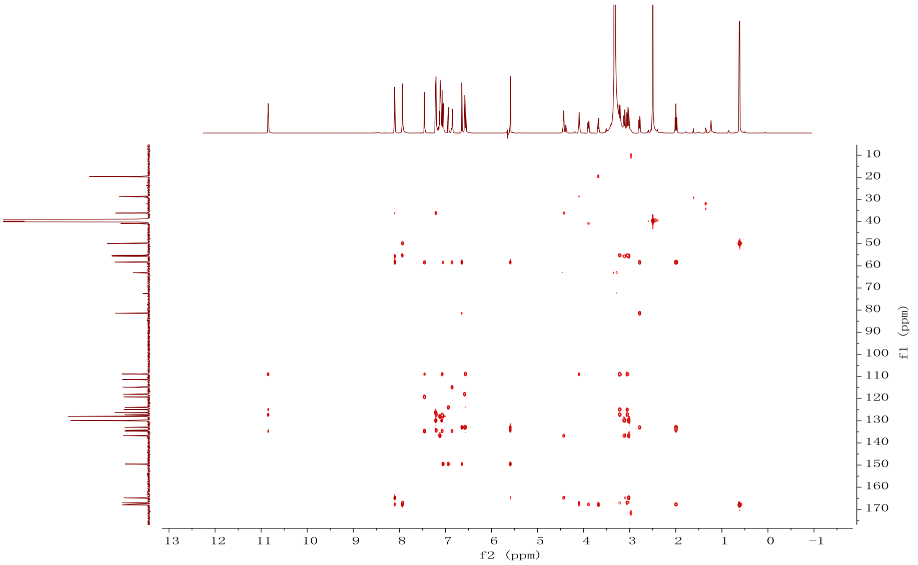

f

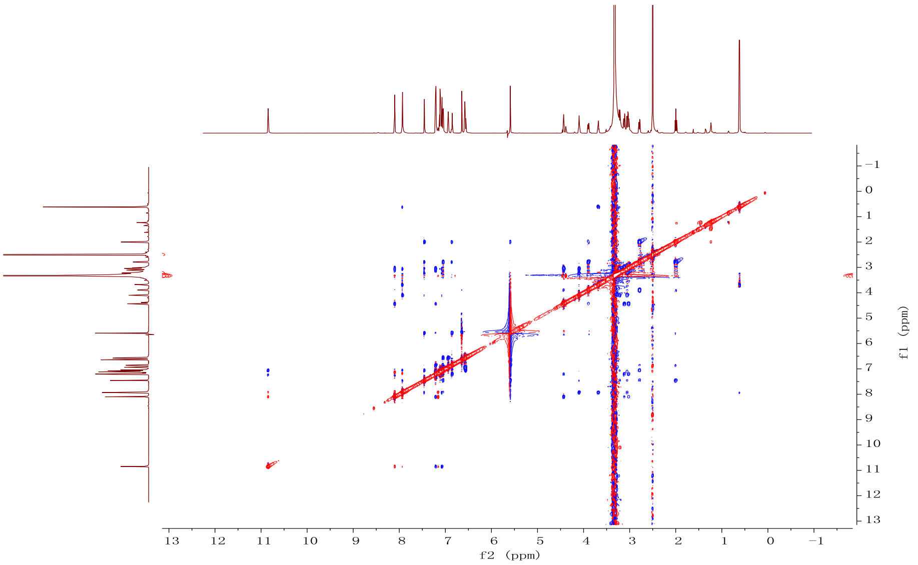

Supplementary Figure. 19. Spectra of compound NAS-7 (a) HRESIMS of compound of NAS-7 (b) ^{1}H NMR of compound of NAS-7 (c) ^{13}C NMR of compound of NAS-7 (d) HSQC of compound of NAS-7 (e) HMBC of compound of NAS-7 (f) ROSEY of compound of NAS-7

a

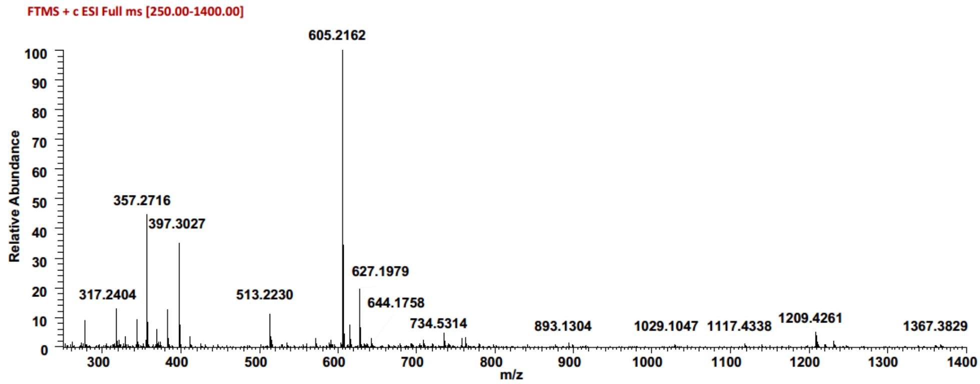

FTMS + c ESI Full ms [250.00-1400.00]
Relative Abundance
m/z
605.2162
357.2716
397.3027
317.2404
513.2230
627.1979
644.1758
734.5314
893.1304
1029.1047
1117.4338
1209.4261
1367.3829

b

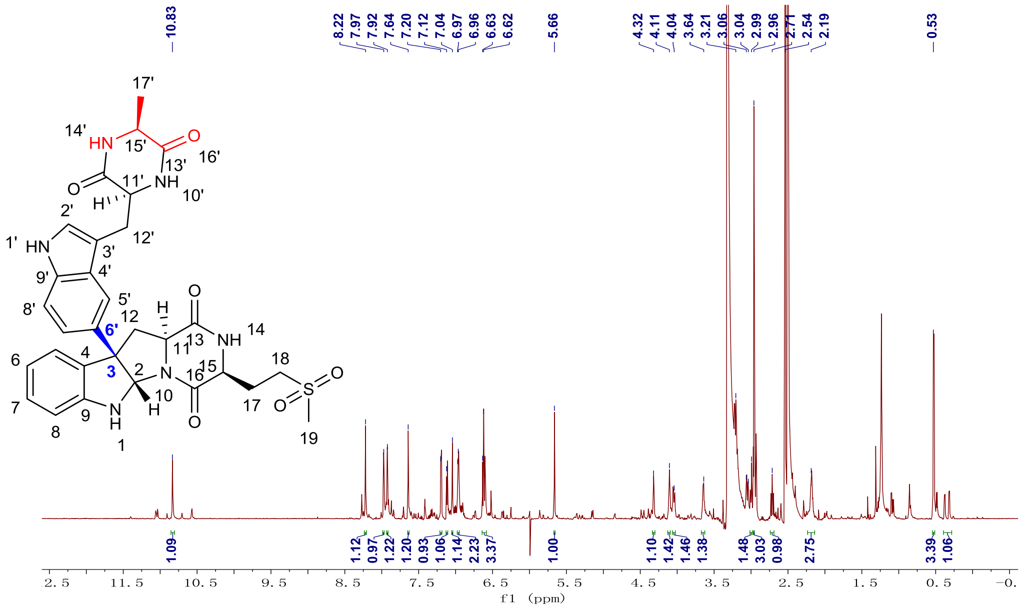

10.83
8.22 7.97 7.92 7.64 7.20 7.12 7.04 6.97 6.96 6.63 6.62
5.66
4.32 4.11 4.04 3.64 3.21 3.06 3.04 2.99 2.96 2.71 2.54 2.19
0.53
1.09 1.12 0.97 1.22 1.20 0.93 1.06 1.14 2.23 3.37 1.00 1.10 1.42 1.46 1.38 1.48 3.03 0.98 2.75 3.39 1.06
f1 (ppm)

c

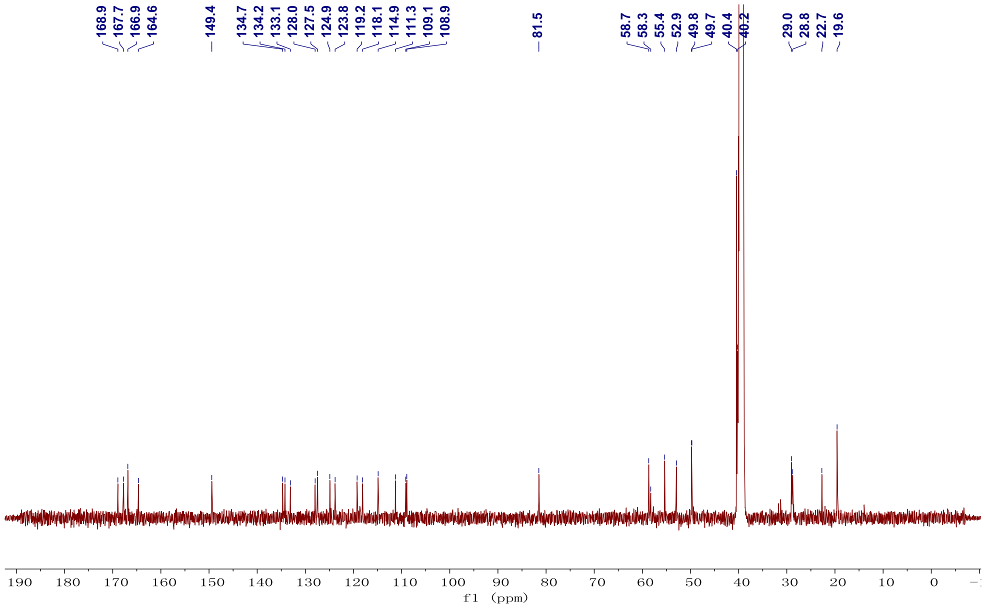

168.9 167.7 166.9 164.6
149.4
134.7 134.2 133.1 128.0 127.5 124.9 123.8 119.2 118.1 114.9 111.3 109.1 108.9
81.5
58.7 58.3 55.4 52.9 49.8 49.7 40.4 40.2
29.0 28.8 22.7 19.6
f1 (ppm)

d

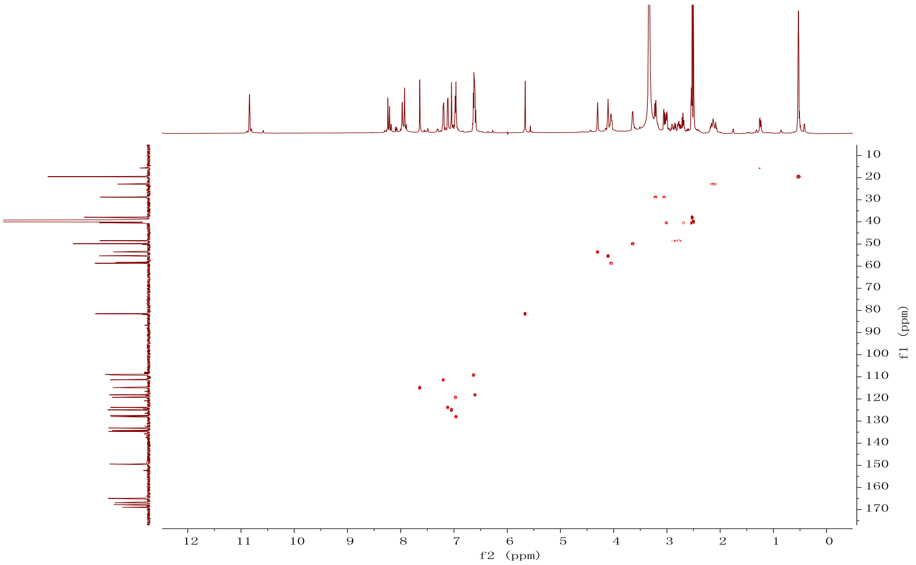

e

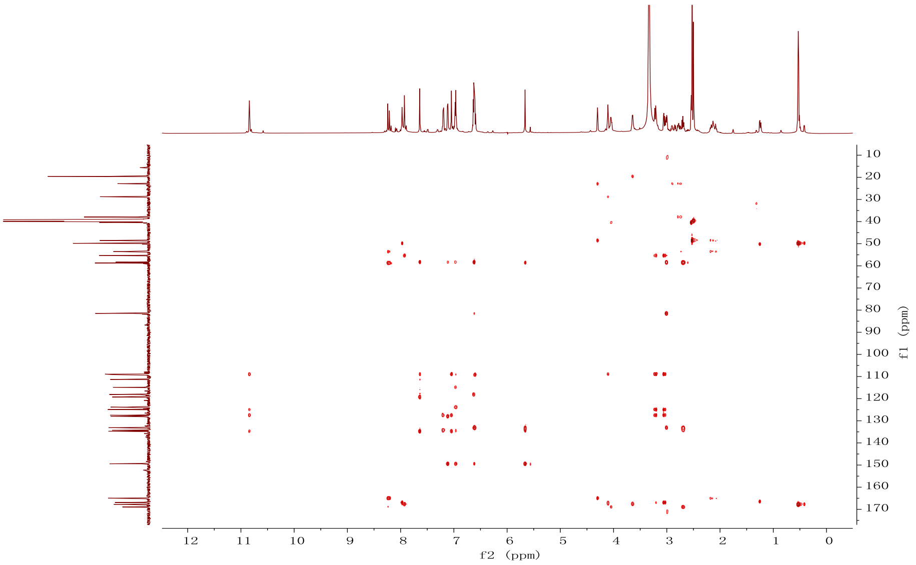

f

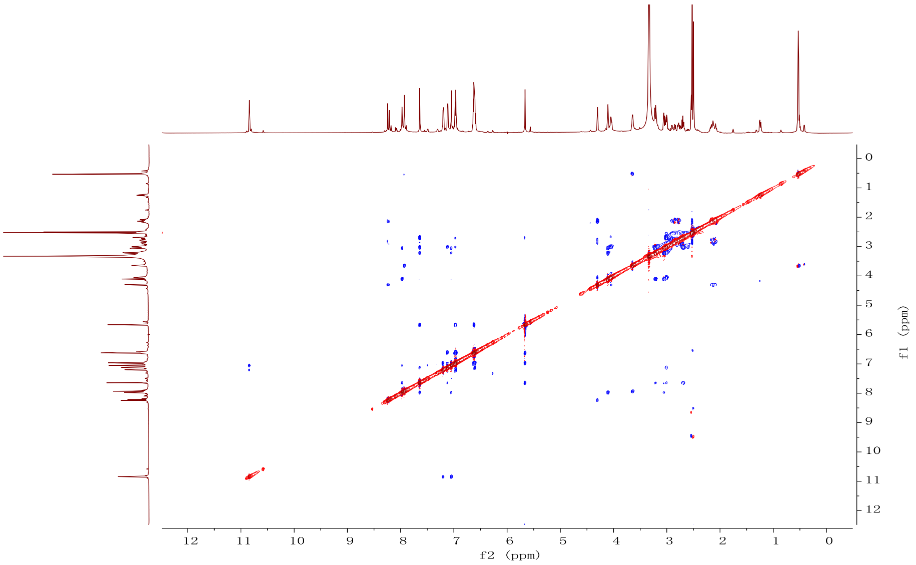

Supplementary Figure. 20. Spectra of compound NAS-8 (a) HRESIMS of compound of NAS-8 (b) ^{1}H NMR of compound of NAS-8 (c) ^{13}C NMR of compound of NAS-8 (d) HSQC of compound of NAS-8 (e) HMBC of compound of NAS-8 (f) ROSEY of compound of NAS-8

a

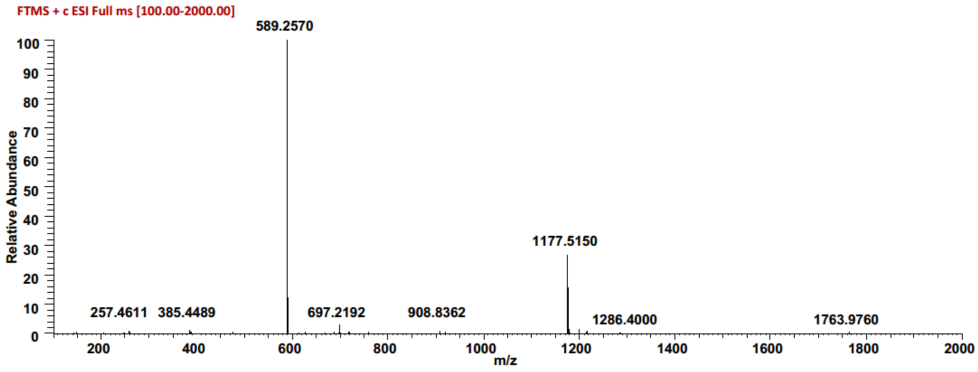
FTMS + c ESI Full ms [100.00-2000.00]
589.2570
1177.5150
257.4611
385.4489
697.2192
908.8362
1286.4000
1763.9760
Relative Abundance
m/z

b

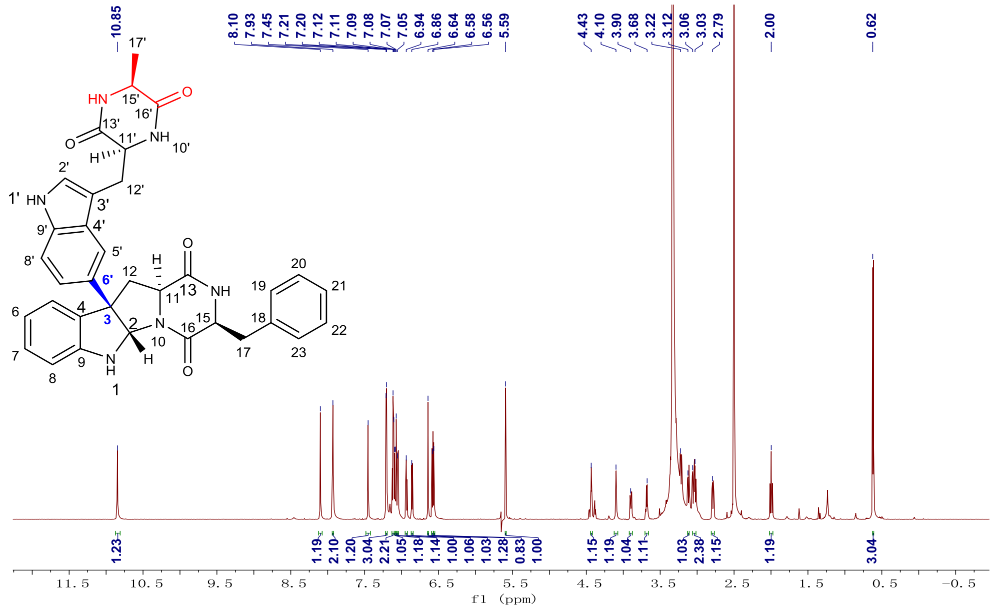
f1 (ppm)

c

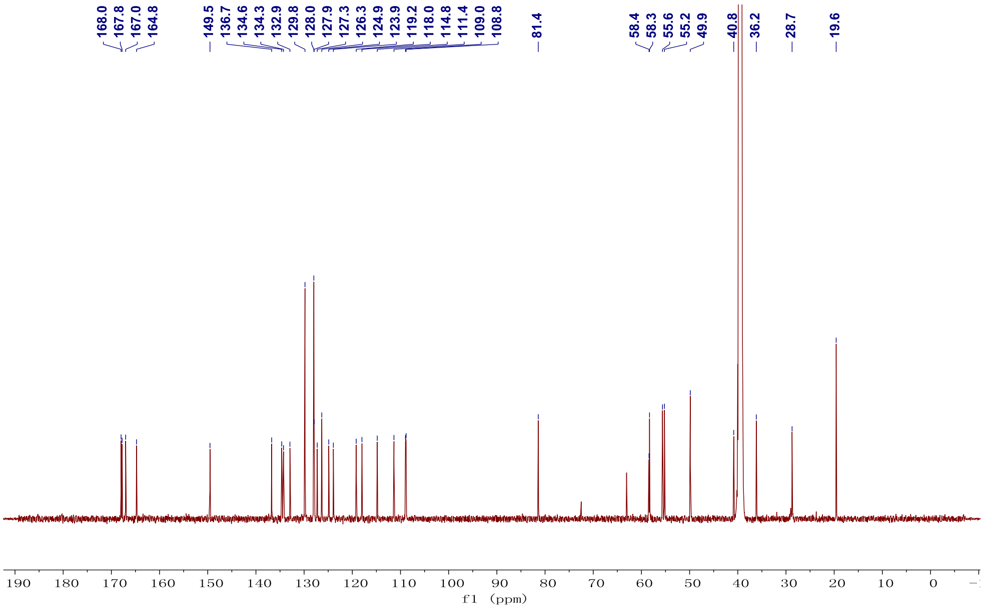
f1 (ppm)

d

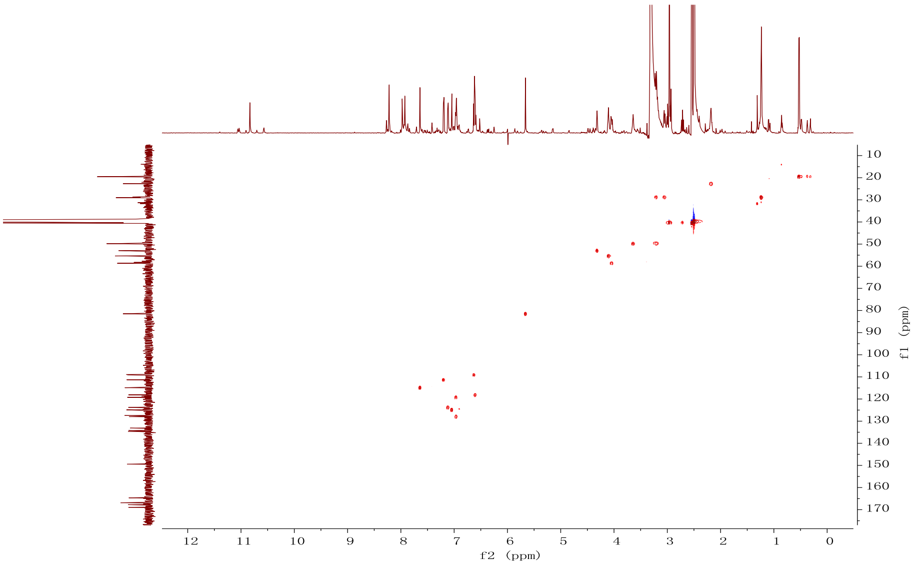

e

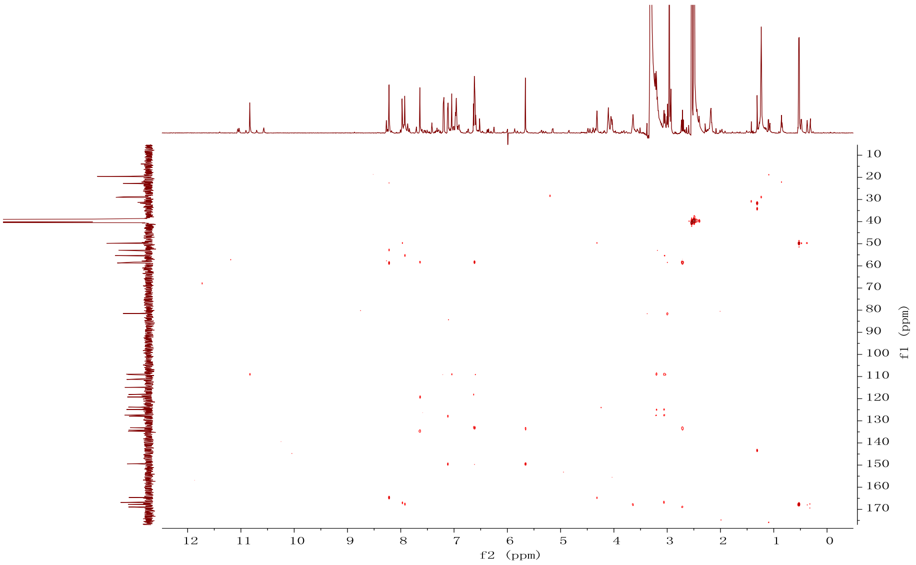

f

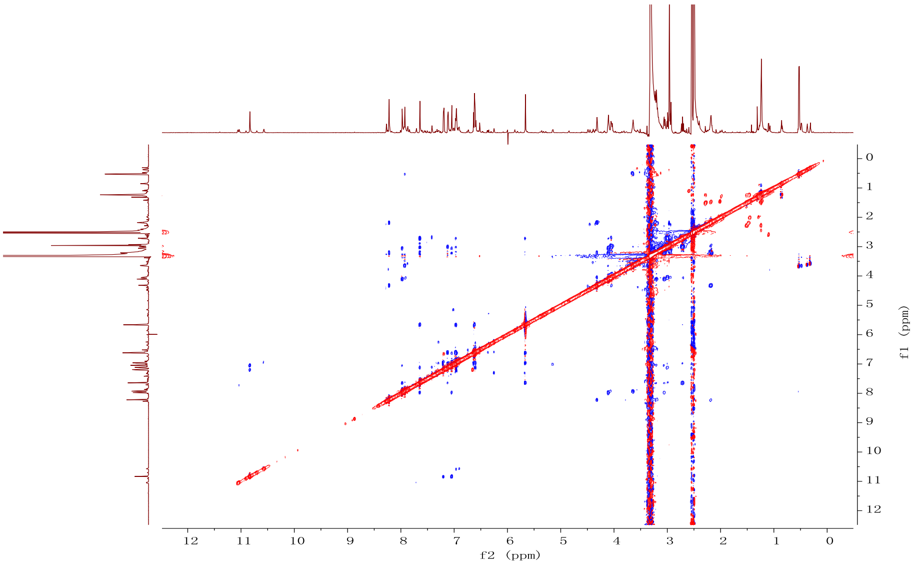

Supplementary Figure. 21. Spectra of compound NAS-9 (a) HRESIMS of compound of NAS-9 (b) ^{1}H NMR of compound of NAS-9 (c) ^{13}C NMR of compound of NAS-9 (d) HSQC of compound of NAS-9 (e) HMBC of compound of NAS-9 (f) ROSEY of compound of NAS-9

a

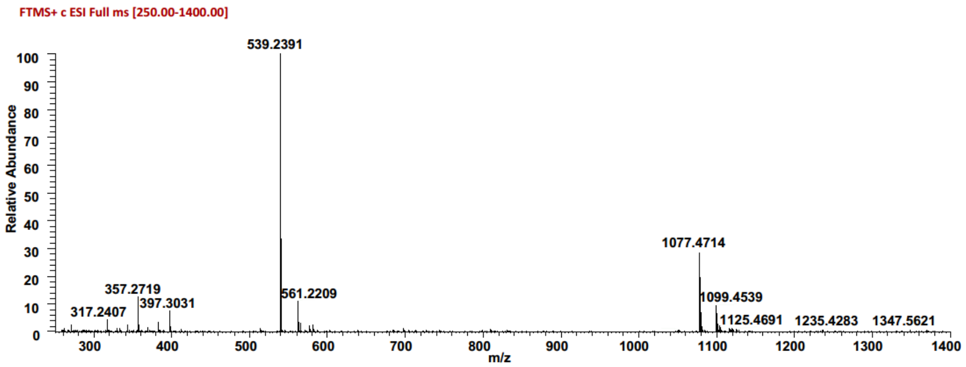
FTMS+ c ESI Full ms [250.00-1400.00]
Relative Abundance
m/z
539.2391
1077.4714
357.2719
397.3031
317.2407
561.2209
1099.4539
1125.4691
1235.4283
1347.5621

b

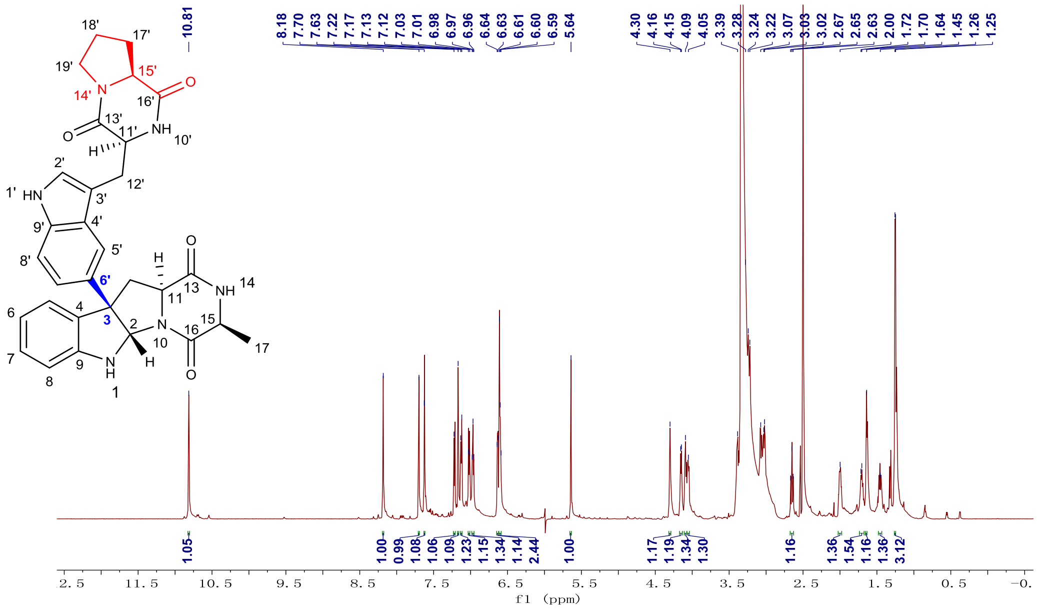
10.81
8.18 7.70 7.63 7.22 7.17 7.13 7.12 7.03 7.01 6.98 6.97 6.96 6.64 6.63 6.61 6.60 6.59 5.64
4.30 4.16 4.15 4.09 4.05 3.39 3.28 3.24 3.22 3.07 3.03 3.02 2.67 2.65 2.63 2.00 1.72 1.70 1.64 1.45 1.26 1.25
1.05 1.00 0.99 1.08 1.06 1.09 1.23 1.15 1.34 1.14 2.44 1.00 1.17 1.19 1.34 1.30 1.16 1.36 1.54 1.16 1.36 3.12
f1 (ppm)

c

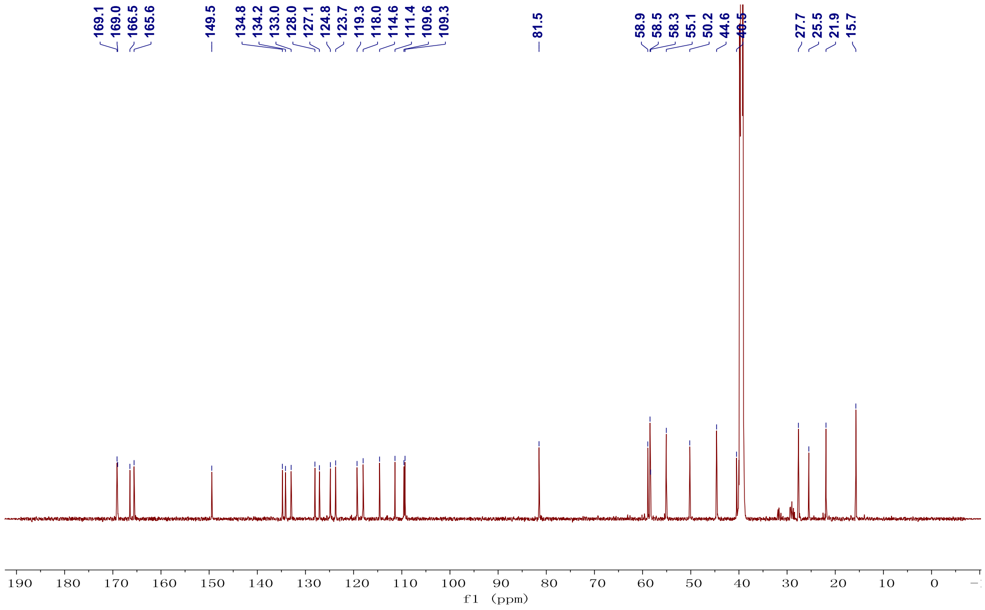
169.1 169.0 166.5 165.6 149.5 134.8 134.2 133.0 128.0 127.1 124.8 123.7 119.3 118.0 114.6 111.4 109.6 109.3 81.5 58.9 58.5 58.3 55.1 50.2 44.6 27.7 25.5 21.9 15.7
f1 (ppm)

d

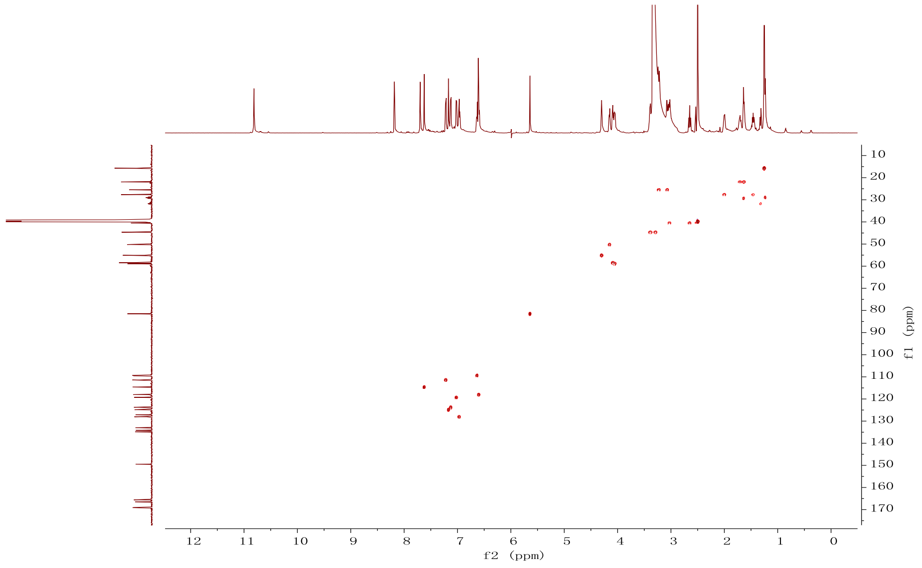

e

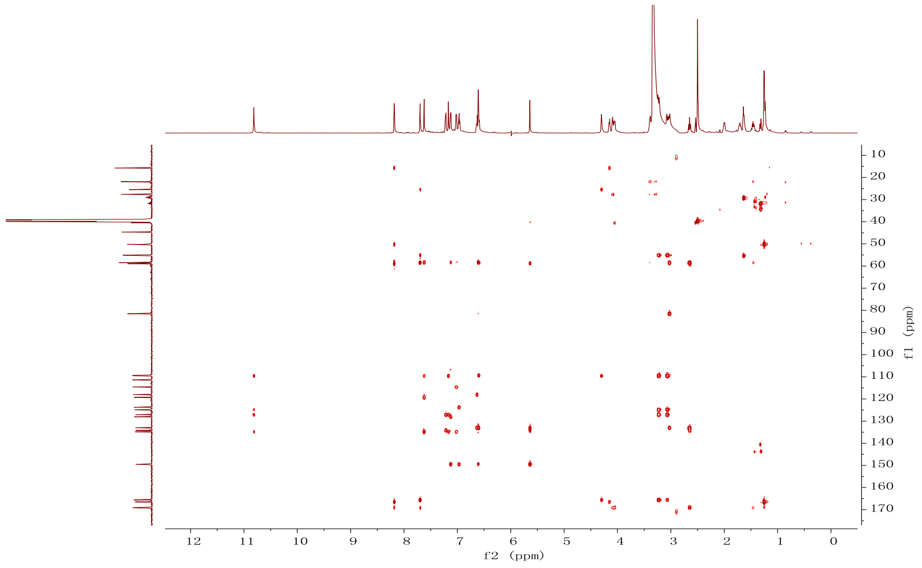

f

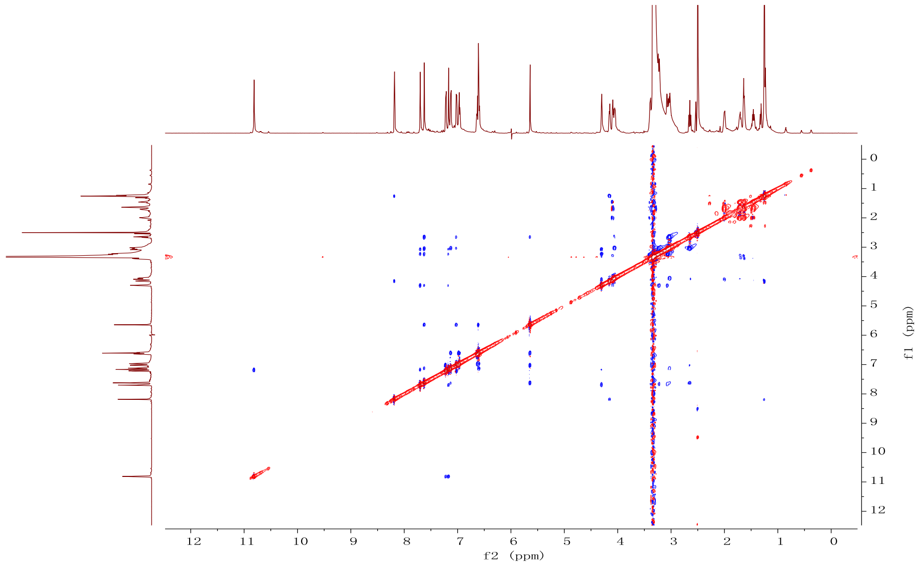

Supplementary Figure. 22. Spectra of compound NAS-10 (a) HRESIMS of compound of NAS-10 (b) ^{1}H NMR of compound of NAS-10 (c) ^{13}C NMR of compound of NAS-10 (d) HSQC of compound of NAS-10 (e) HMBC of compound of NAS-10 (f) ROSEY of compound of NAS-10

a

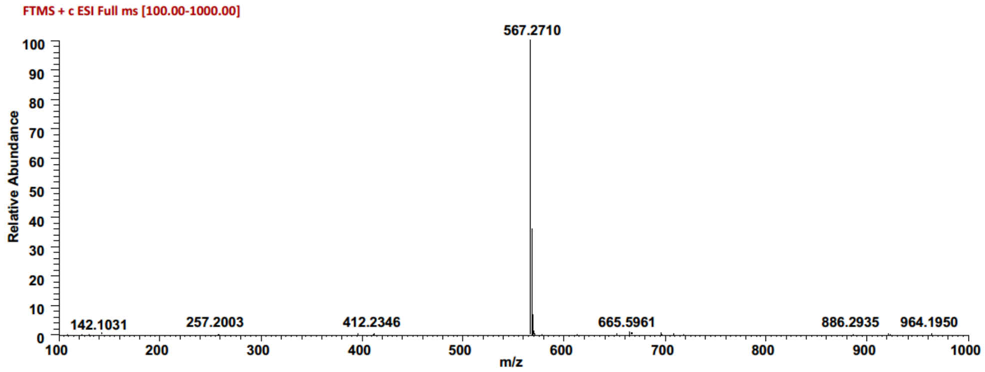
FTMS + c ESI Full ms [100.00-1000.00]
567.2710
Relative Abundance
142.1031
257.2003
412.2346
665.5961
886.2935
964.1950
m/z

b

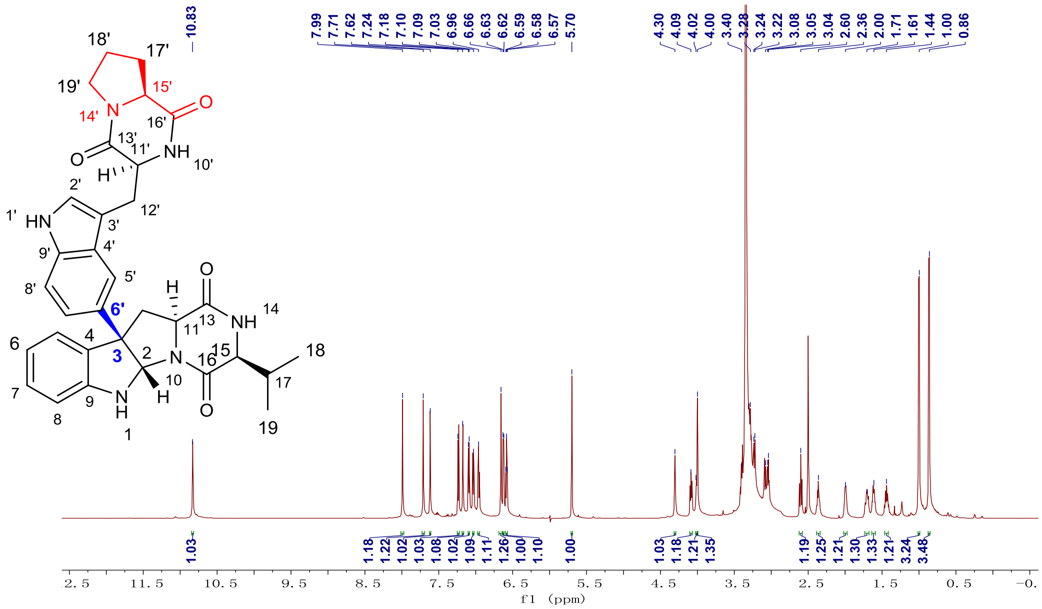
10.83
7.99
7.71
7.62
7.24
7.18
7.10
7.09
7.03
6.96
6.66
6.63
6.62
6.59
6.58
6.57
5.70
4.30
4.09
4.02
4.00
3.40
3.28
3.24
3.22
3.08
3.05
3.04
2.60
2.36
2.00
1.71
1.61
1.44
1.00
0.86
f1 (ppm)

c

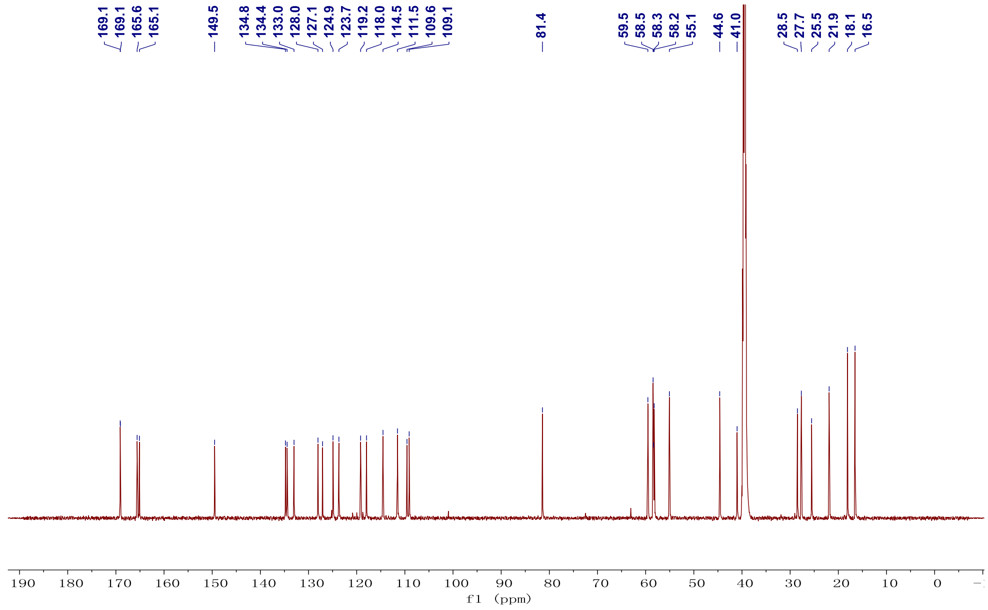
169.1
169.1
165.6
165.1
149.5
134.8
134.4
133.0
128.0
127.1
124.9
123.7
119.2
118.0
114.5
111.5
109.6
109.1
81.4
59.5
58.5
58.3
58.2
55.1
44.6
41.0
28.5
27.7
25.5
21.9
18.1
16.5
f1 (ppm)

d

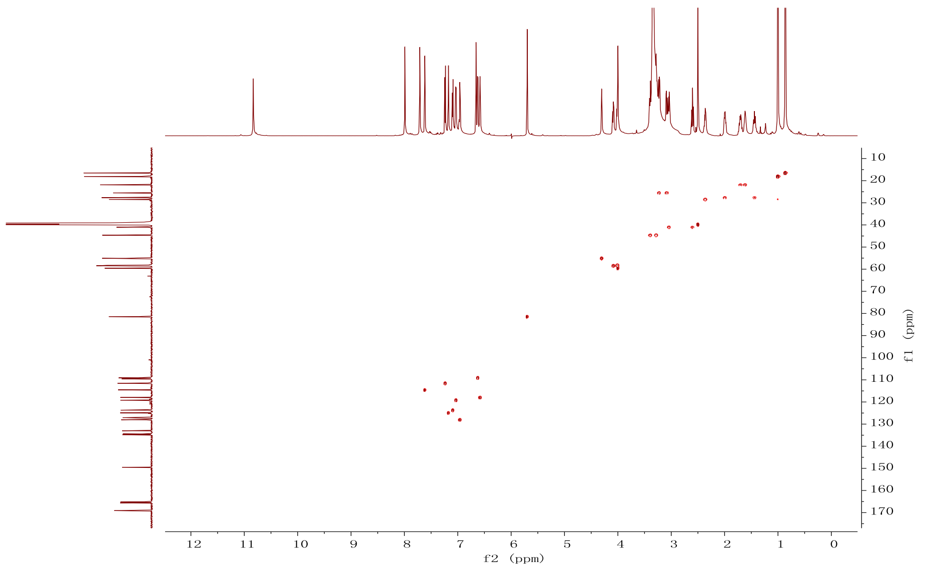

e

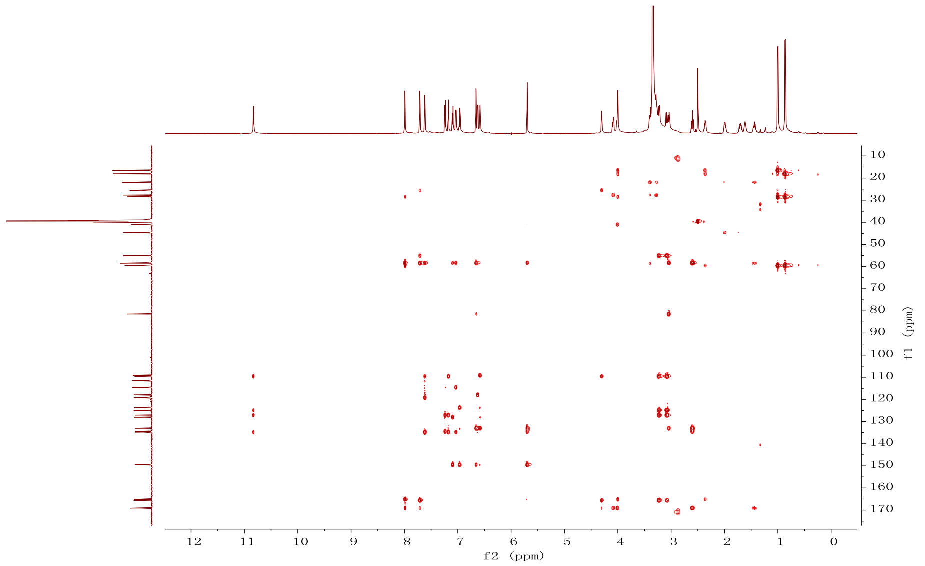

f

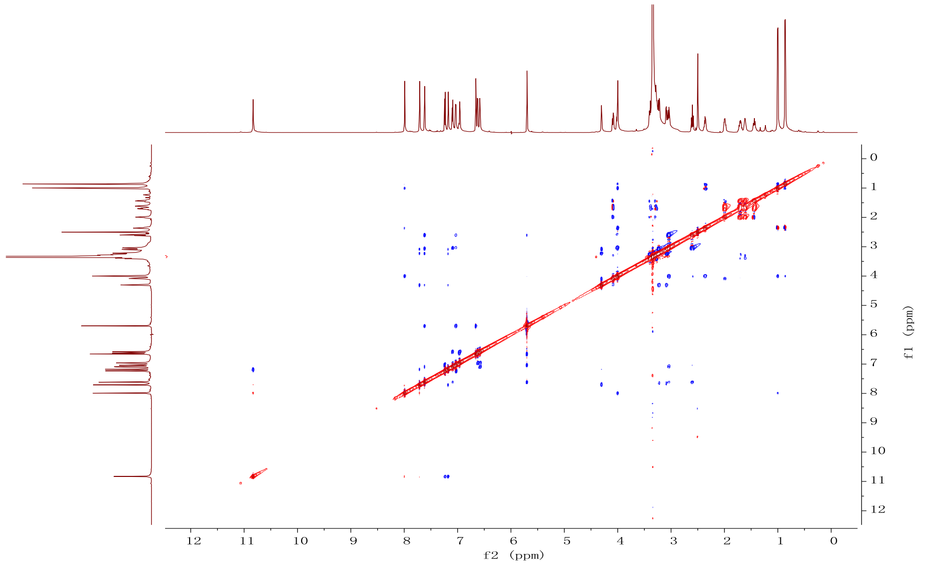

Supplementary Figure. 23. Spectra of compound NAS-11 (a) HRESIMS of compound of NAS-11 (b) ^{1}H NMR of compound of NAS-11 (c) ^{13}C NMR of compound of NAS-11 (d) HSQC of compound of NAS-11 (e) HMBC of compound of NAS-11 (f) ROSEY of compound of NAS-11

a

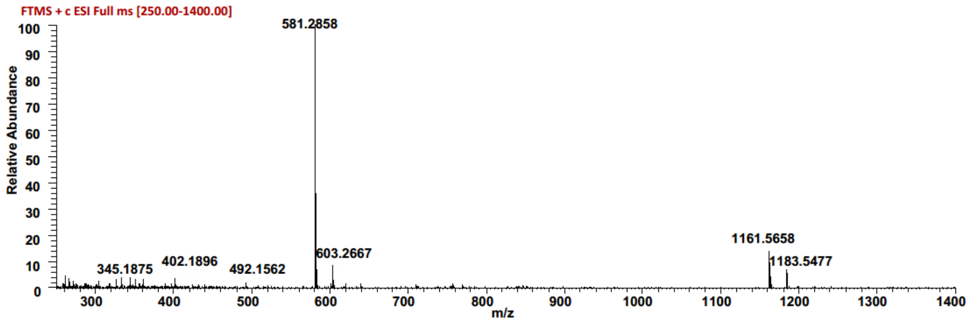

FTMS + c ESI Full ms [250.00-1400.00]
Relative Abundance
m/z
581.2858
603.2667
1161.5658
1183.5477
345.1875
402.1896
492.1562

b

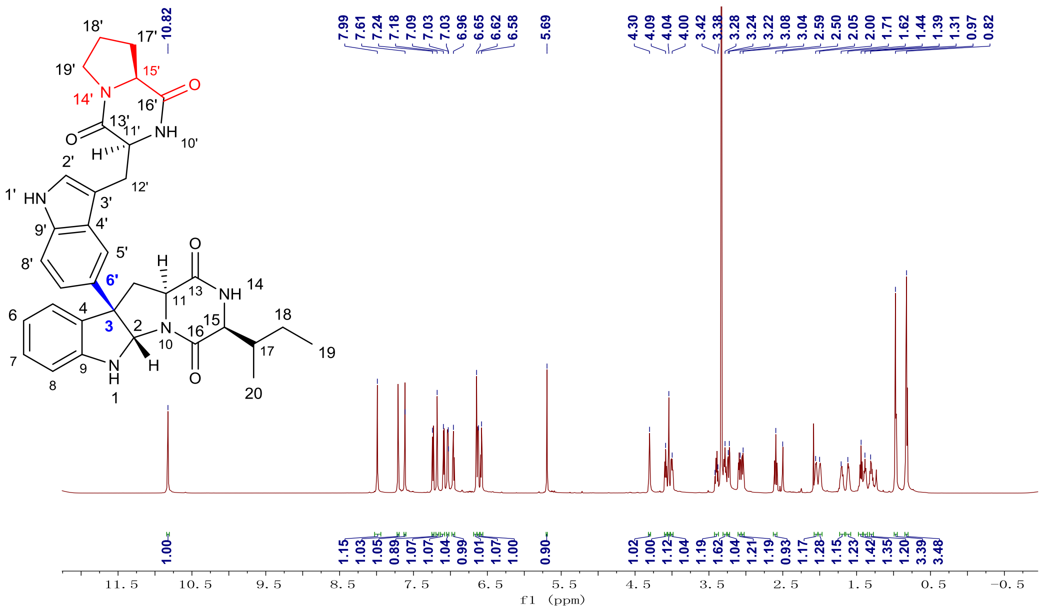

10.82
7.99 7.61 7.24 7.18 7.09 7.03 7.03 6.96 6.65 6.62 6.58
5.69
4.30 4.09 4.04 4.00 3.42 3.38 3.28 3.24 3.22 3.08 3.04 2.59 2.50 2.05 2.00 1.71 1.62 1.44 1.39 1.31 0.97 0.82
1.00
1.15 1.03 1.05 0.89 1.07 1.07 1.04 0.99 1.01 1.07 1.00
0.90
1.02 1.00 1.12 1.04 1.19 1.62 1.04 1.21 1.19 0.93 1.17 1.28 1.15 1.23 1.42 1.35 1.20 3.39 3.48
f1 (ppm)

c

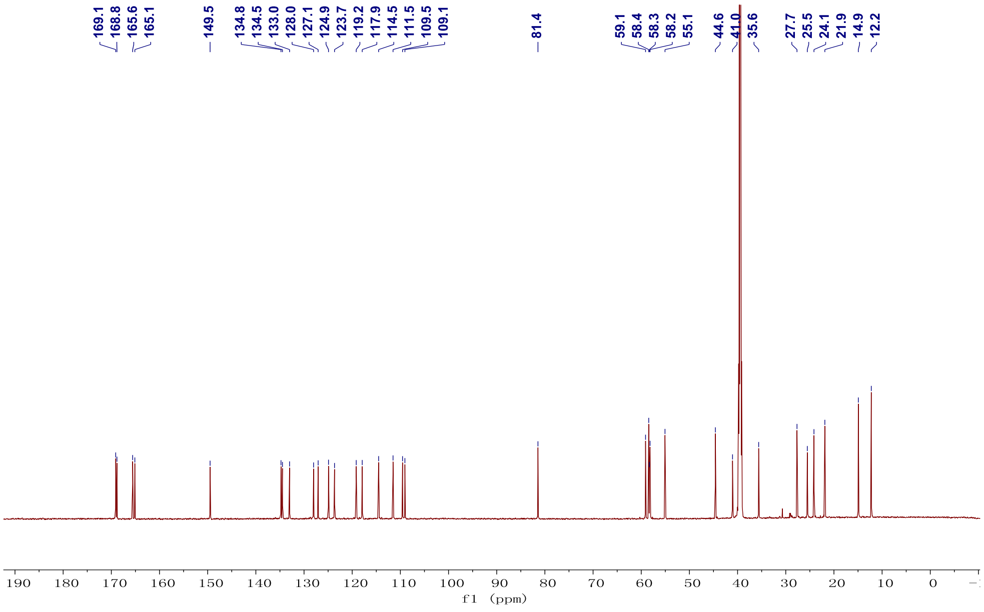

169.1 168.8 165.6 165.1
149.5
134.8 134.5 133.0 128.0 127.1 124.9 123.7 119.2 117.9 114.5 111.5 109.5 109.1
81.4
59.1 58.4 58.3 58.2 55.1
44.6 41.0 35.6
27.7 25.5 24.1 21.9 14.9 12.2
f1 (ppm)

d

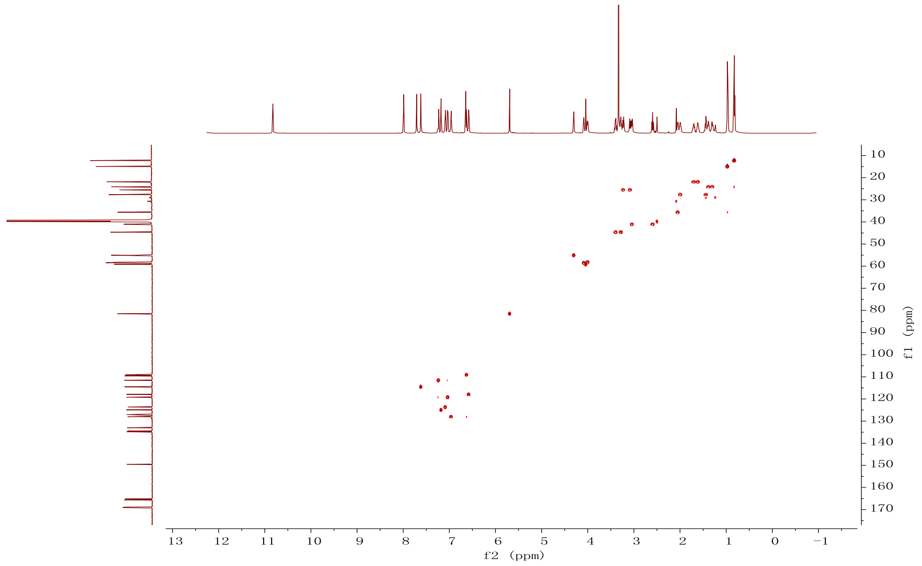

e

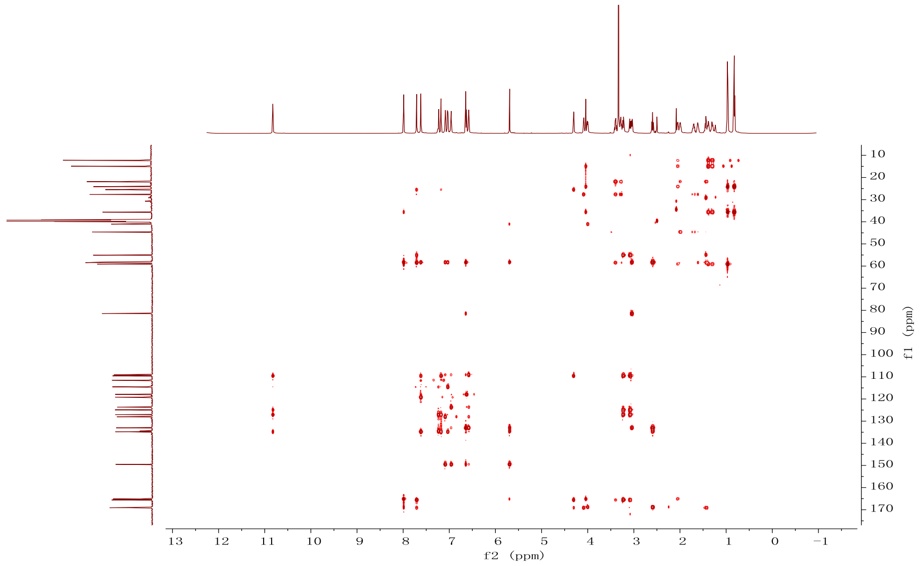

f

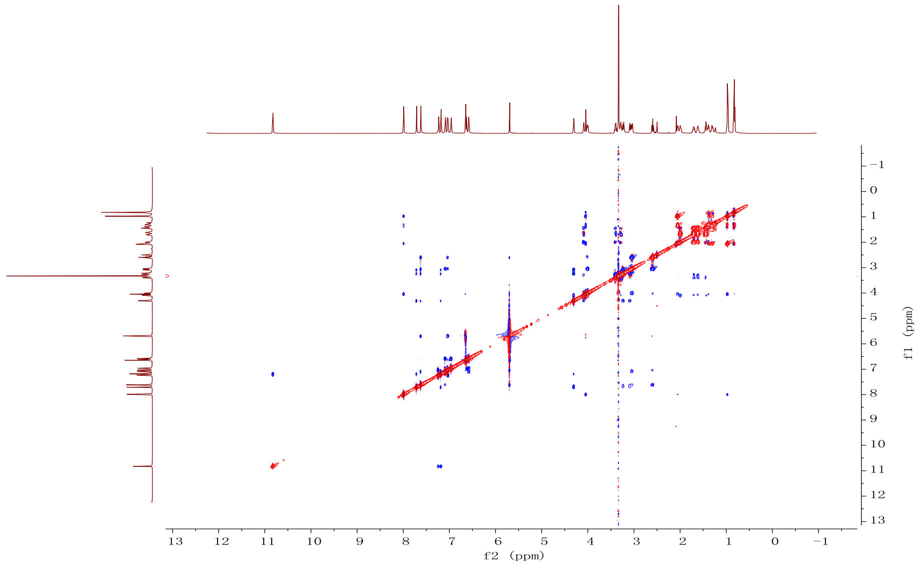

Supplementary Figure. 24. Spectra of compound NAS-12 (a) HRESIMS of compound of NAS-12 (b) ^{1}H NMR of compound of NAS-12 (c) ^{13}C NMR of compound of NAS-11 (d) HSQC of compound of NAS-12 (e) HMBC of compound of NAS-12 (f) ROSEY of compound of NAS-12

a

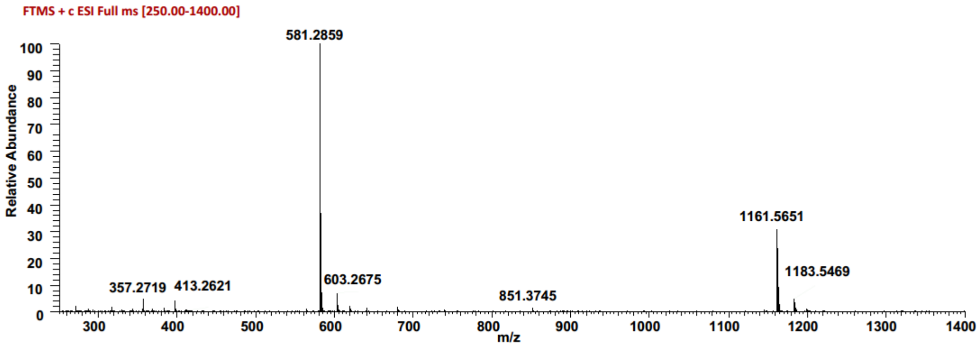

FTMS + c ESI Full ms [250.00-1400.00]
581.2859
603.2675
1161.5651
1183.5469
357.2719
413.2621
851.3745
Relative Abundance
m/z

b

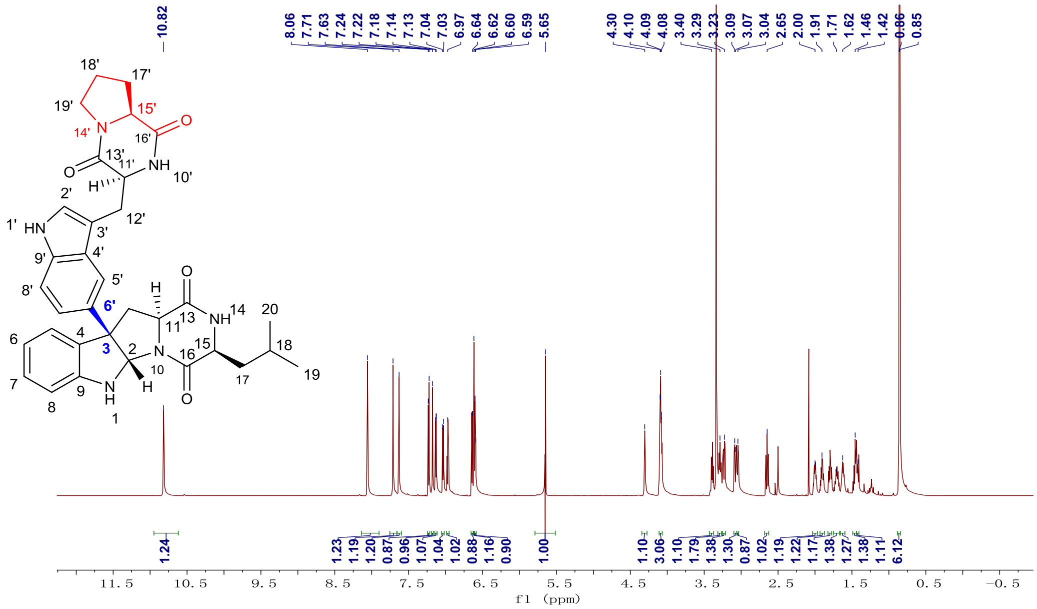

10.82
8.06
7.71
7.63
7.24
7.22
7.18
7.14
7.13
7.04
7.03
6.97
6.64
6.62
6.60
6.59
5.65
4.30
4.10
4.09
4.08
3.40
3.29
3.23
3.09
3.07
3.04
2.65
2.00
1.91
1.71
1.62
1.46
1.42
0.86
0.85
f1 (ppm)

c

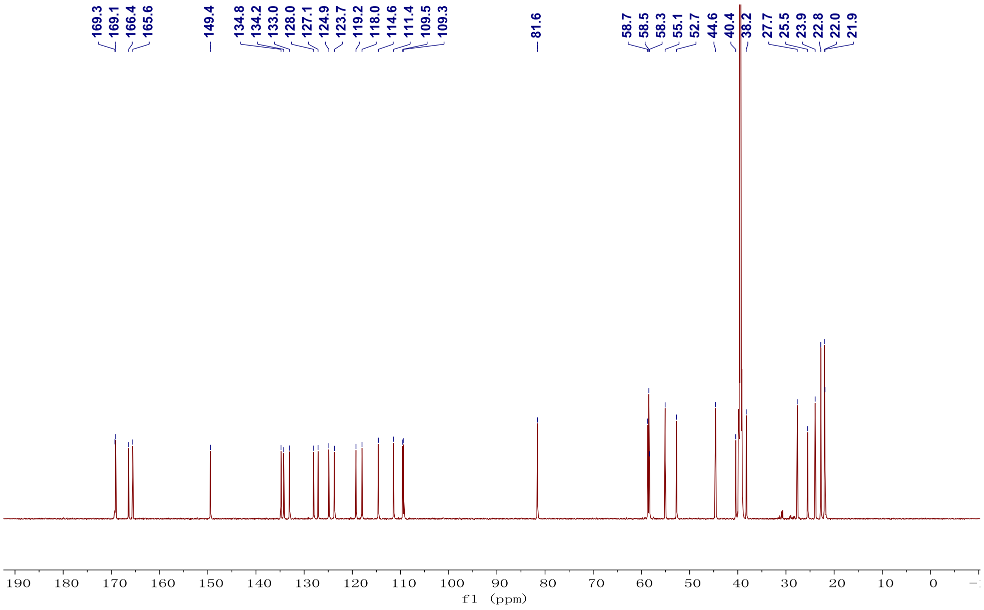

169.3
169.1
166.4
165.6
149.4
134.8
134.2
133.0
128.0
127.1
124.9
123.7
119.2
118.0
114.6
111.4
109.5
109.3
81.6
58.7
58.5
58.3
55.1
52.7
44.6
40.4
38.2
27.7
25.5
23.9
22.8
22.0
21.9
f1 (ppm)

d

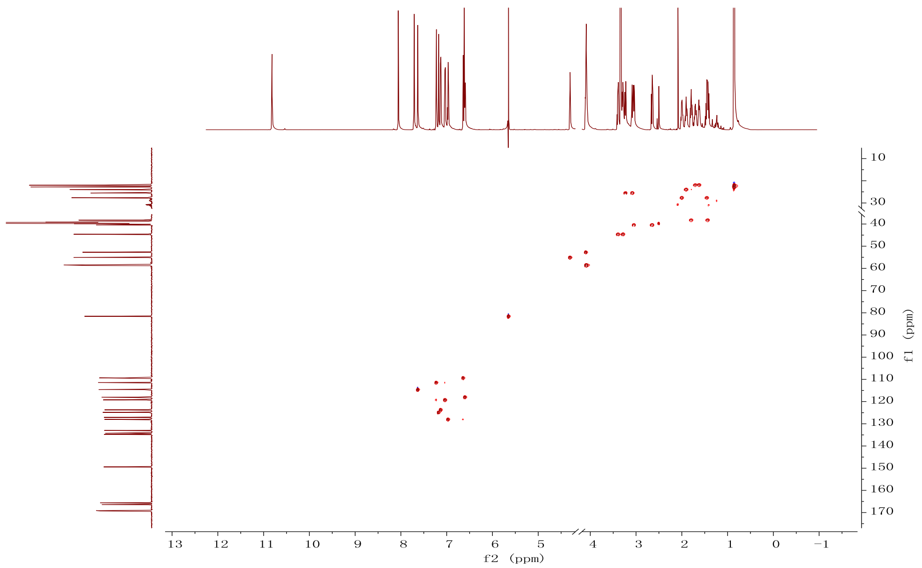

e

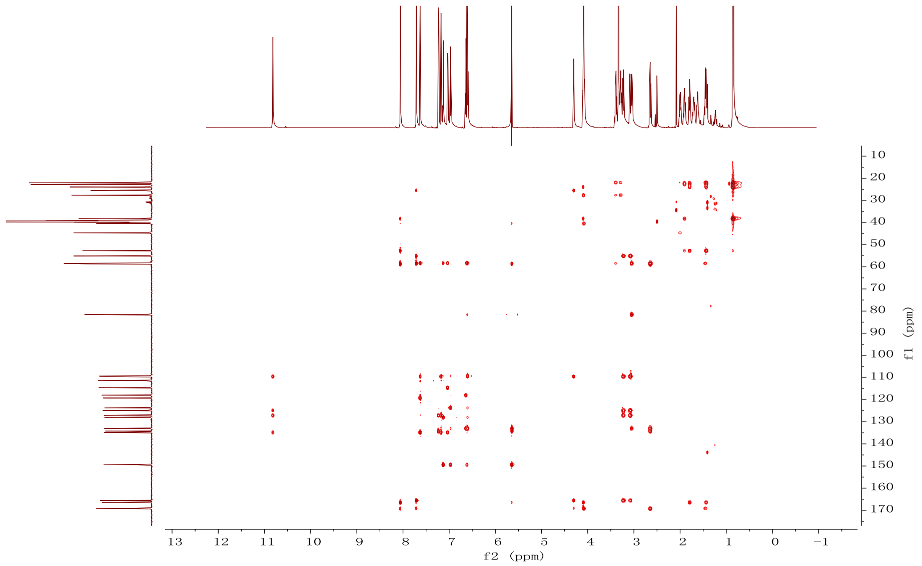

f

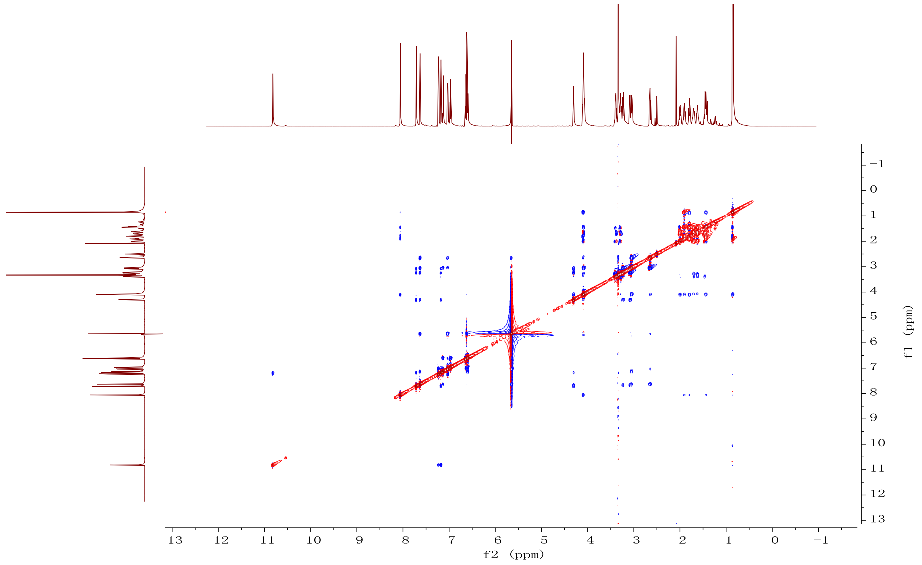

Supplementary Figure. 25. Spectra of compound NAS-13 (a) HRESIMS of compound of NAS-13 (b) ^{1}H NMR of compound of NAS-13 (c) ^{13}C NMR of compound of NAS-13 (d) HSQC of compound of NAS-13 (e) HMBC of compound of NAS-13 (f) ROSEY of compound of NAS-13

a

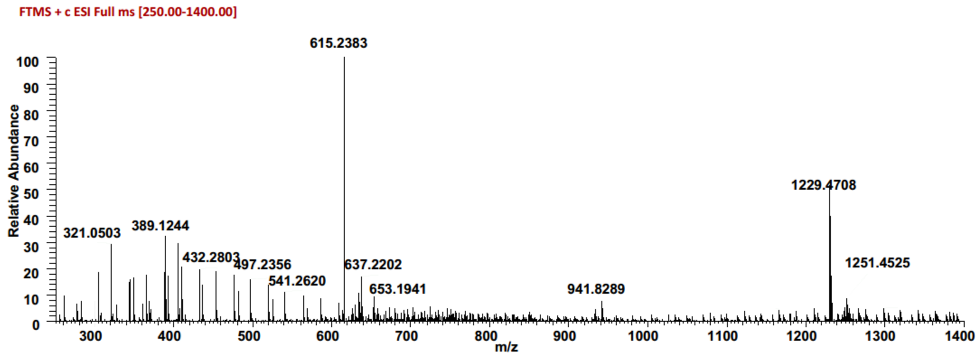

FTMS + c ESI Full ms [250.00-1400.00]
Relative Abundance
m/z
615.2383
1229.4708
1251.4525
321.0503
389.1244
432.2803
497.2356
541.2620
637.2202
653.1941
941.8289

b

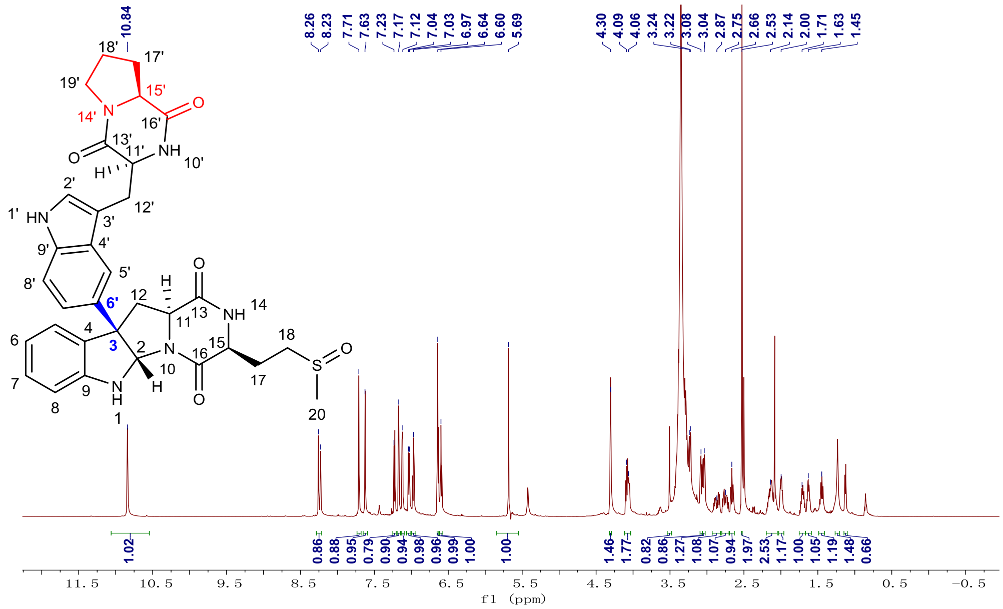

10.84
8.26
8.23
7.71
7.63
7.23
7.17
7.12
7.04
7.03
6.97
6.64
6.60
5.69
4.30
4.09
4.06
3.24
3.22
3.08
3.04
2.87
2.75
2.66
2.53
2.14
2.00
1.71
1.63
1.45
1.02
0.86
0.88
0.95
0.79
0.90
0.94
0.98
0.96
0.99
1.00
1.00
1.46
1.77
0.82
0.86
1.27
1.08
1.07
0.94
1.97
2.53
1.17
1.00
1.05
1.19
1.48
0.66
f1 (ppm)

c

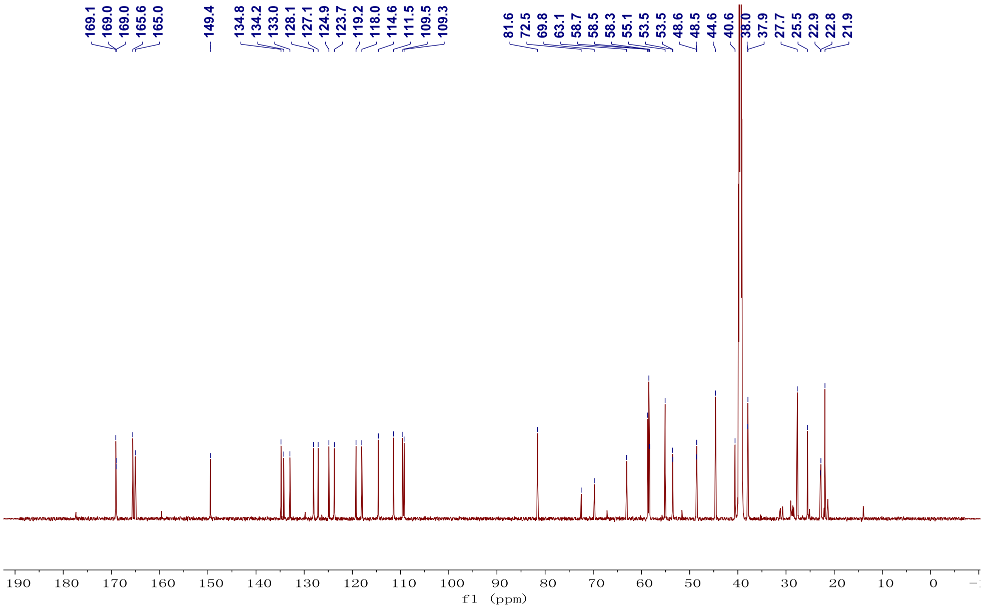

169.1
169.0
169.0
165.6
165.0
149.4
134.8
134.2
133.0
128.1
127.1
124.9
123.7
119.2
118.0
114.6
111.5
109.5
109.3
81.6
72.5
69.8
63.1
58.7
58.5
58.3
55.1
53.5
53.5
48.6
48.5
44.6
40.6
38.0
37.9
27.7
25.5
22.9
22.8
21.9
f1 (ppm)

d

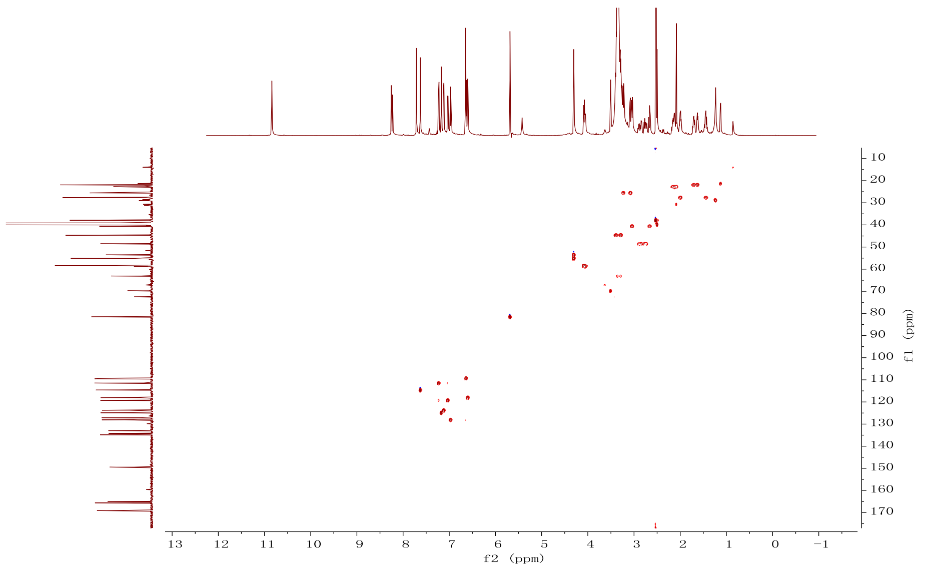

e

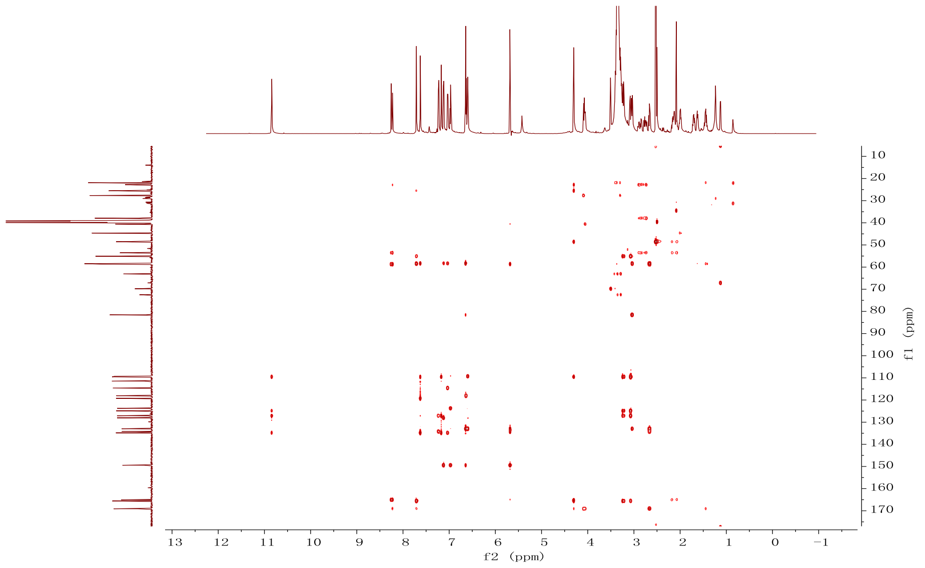

f

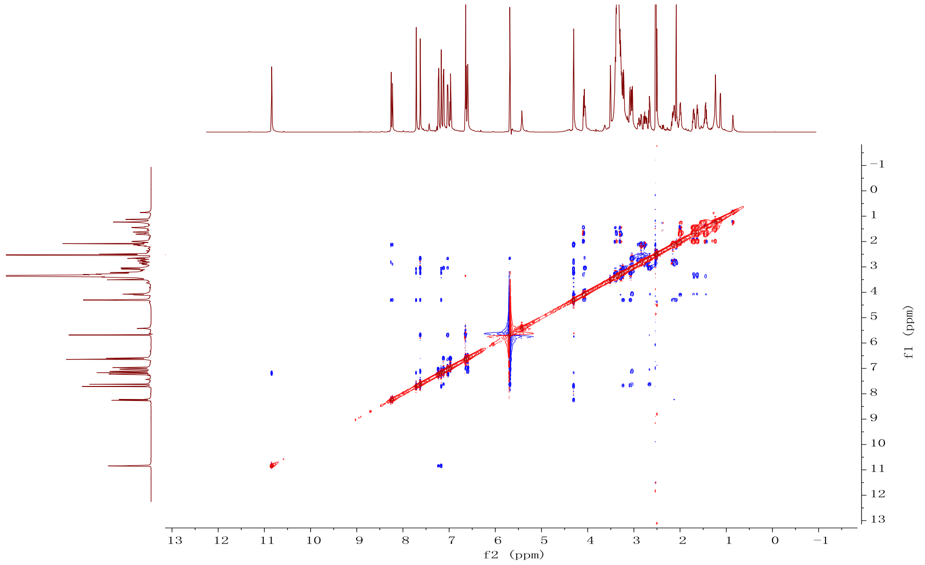

Supplementary Figure. 26.Spectra of compound NAS-14(a) HRESIMS of compound of NAS-14 (b)^{1}H NMR of compound of NAS-14 (c) ^{13}C NMR of compound of NAS-14(d)HSQC of compound of NAS-14(e) HMBC of compound of NAS-14(f) ROSEY of compound of NAS-14

a

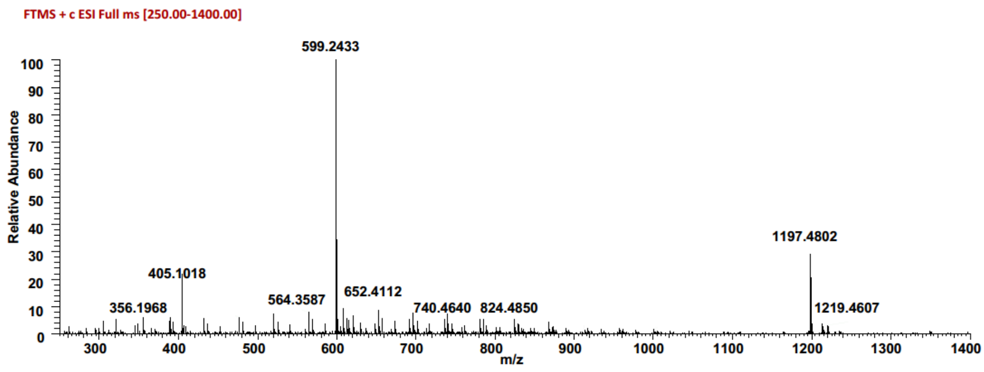
FTMS + c ESI Full ms [250.00-1400.00]
Relative Abundance
m/z
599.2433
405.1018
356.1968
564.3587
652.4112
740.4640
824.4850
1197.4802
1219.4607

b

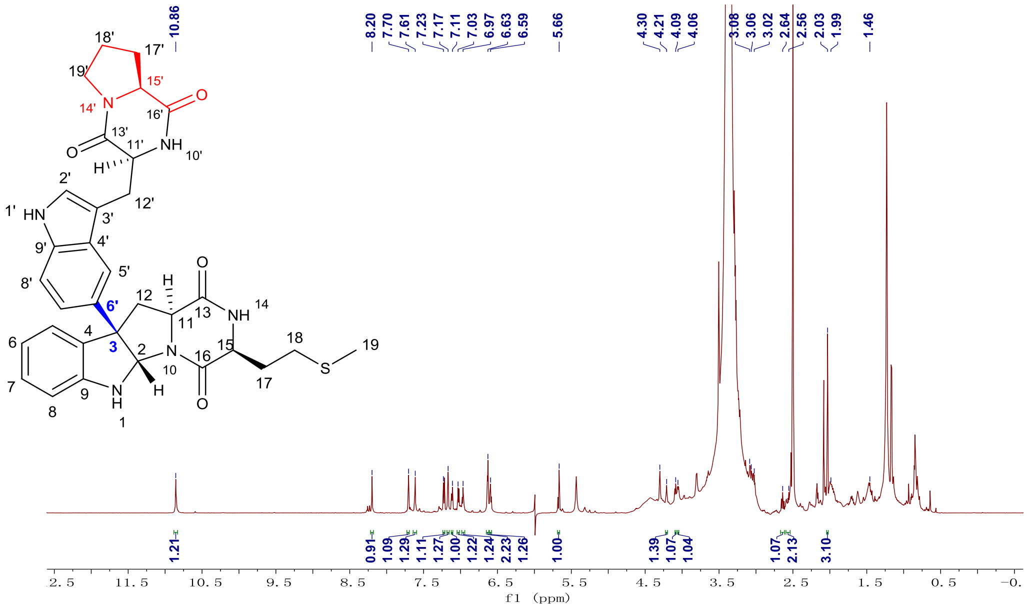
f1 (ppm)

c

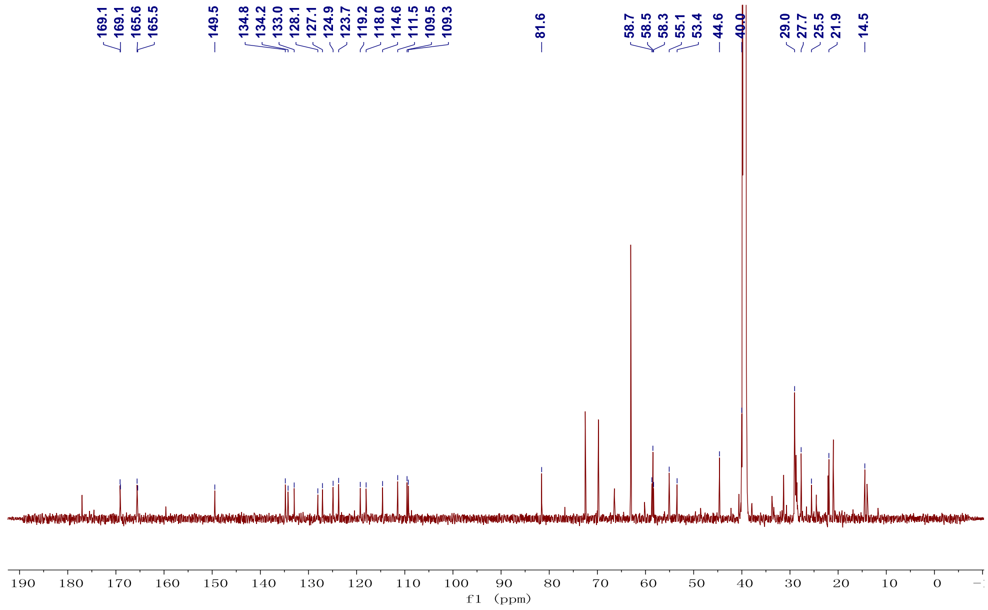
f1 (ppm)

d

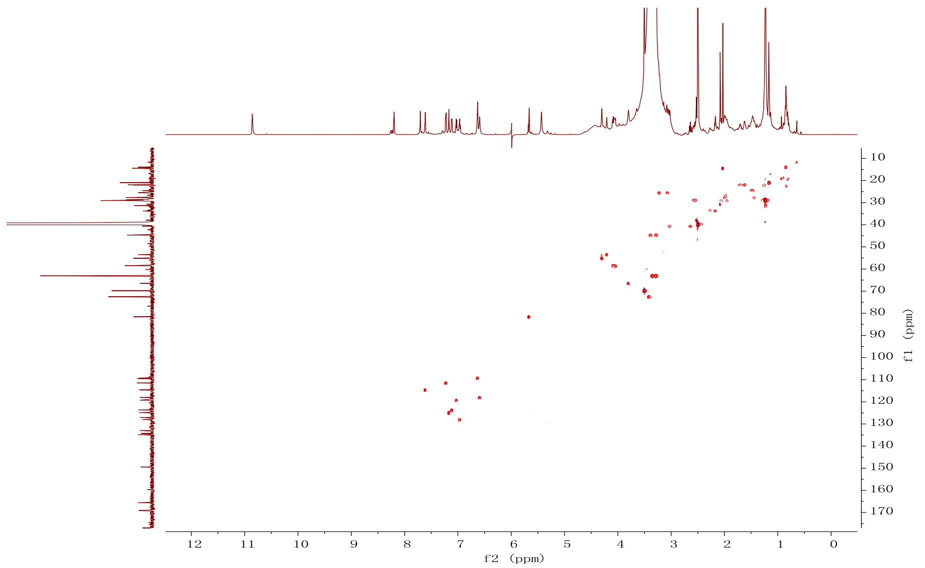

e

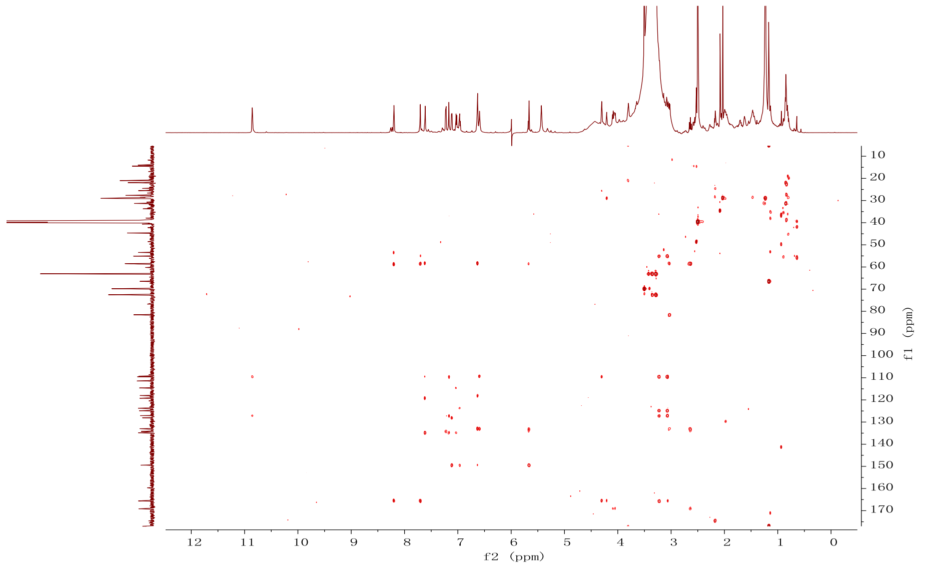

f

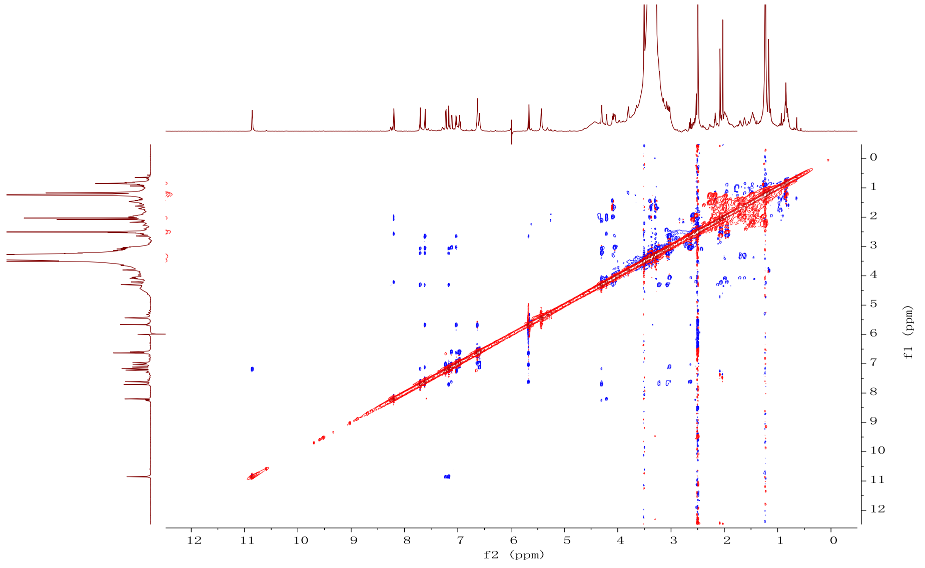

Supplementary Figure. 27.Spectra of compound NAS-15(a) HRESIMS of compound of NAS-15 (b)^{1}H NMR of compound of NAS-15 (c) ^{13}C NMR of compound of NAS-15(d)HSQC of compound of NAS-15(e) HMBC of compound of NAS-15(f) ROSEY of compound of NAS-15

a

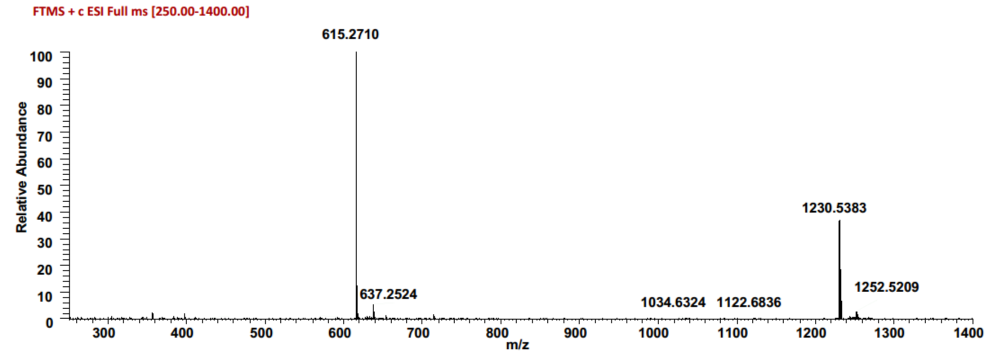
FTMS + c ESI Full ms [250.00-1400.00]
615.2710
637.2524
1034.6324
1122.6836
1230.5383
1252.5209
Relative Abundance
m/z

b

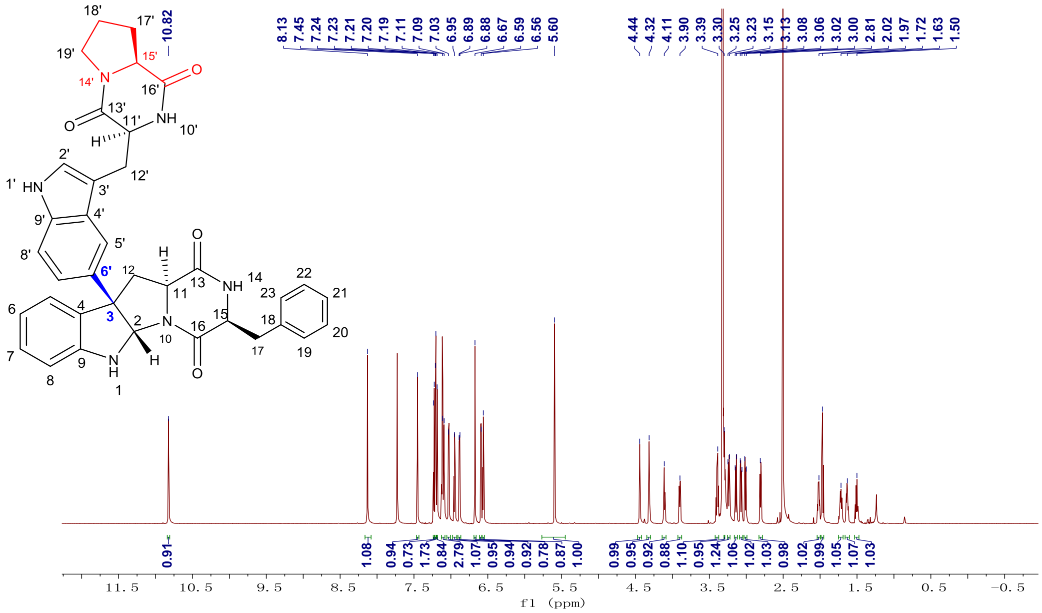
f1 (ppm)

c

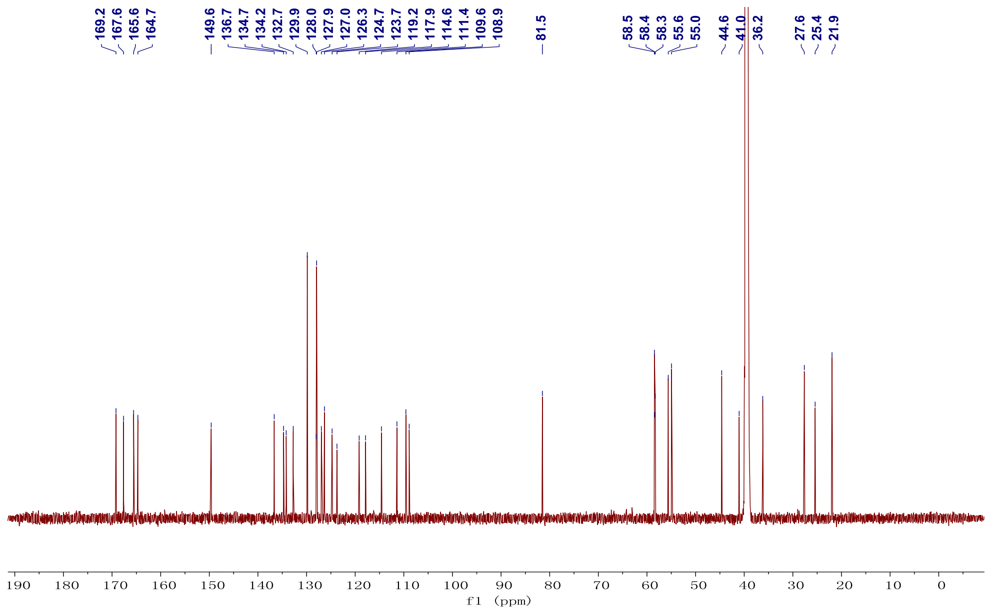
f1 (ppm)

d

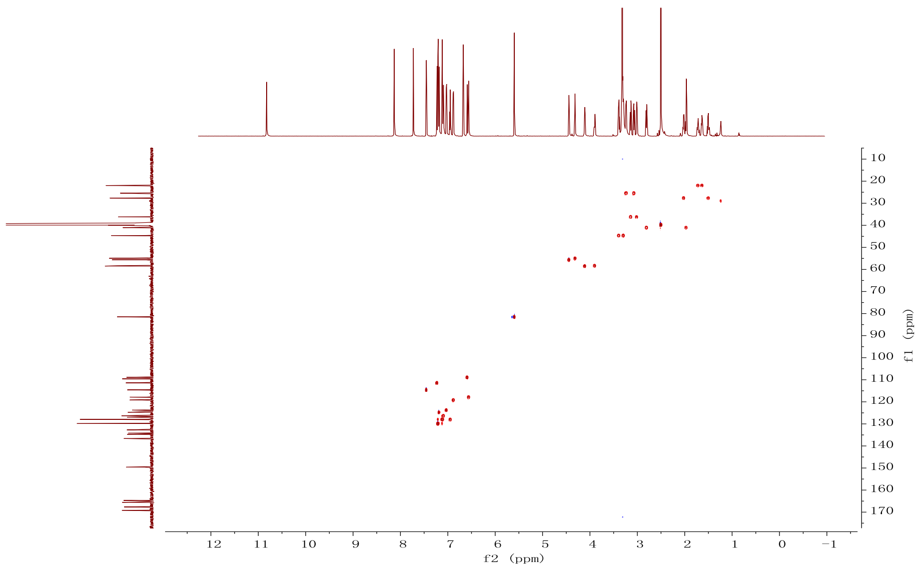

e

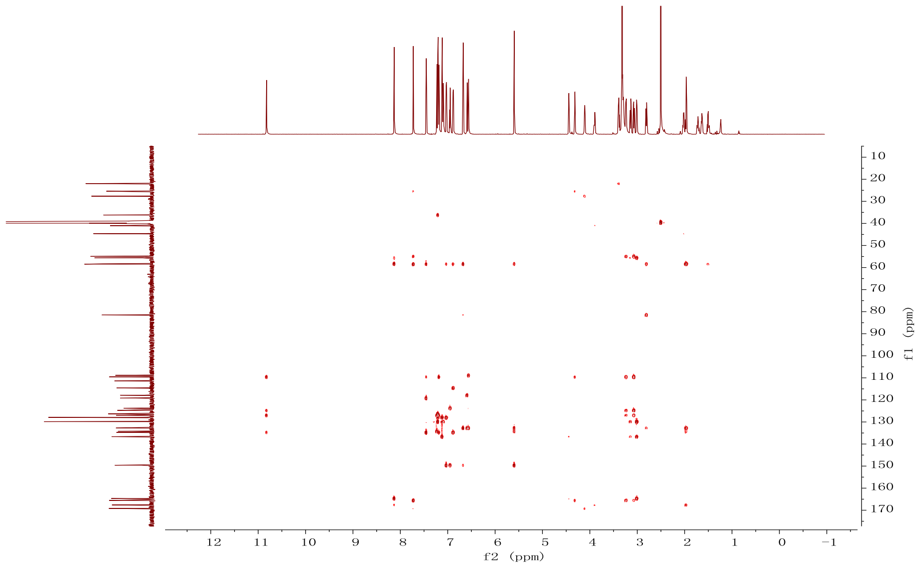

f

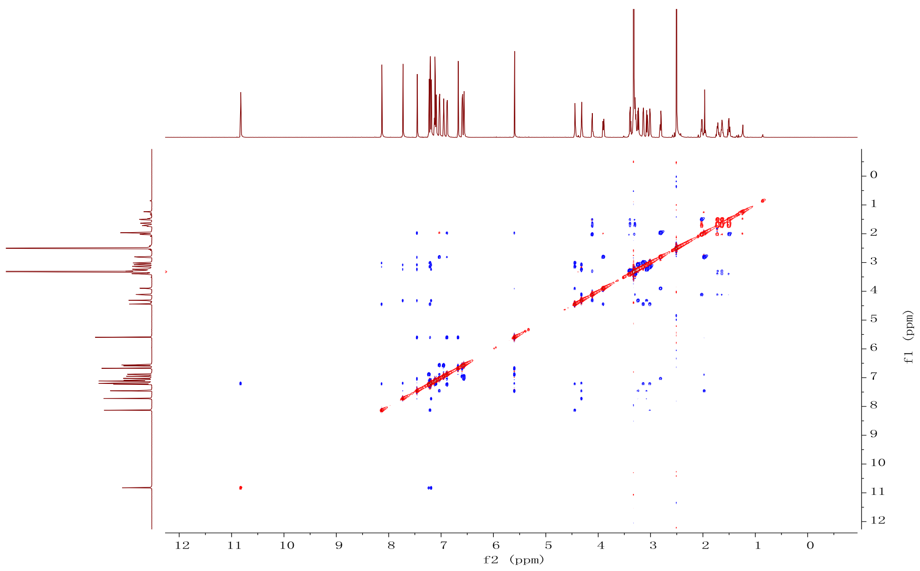

Supplementary Figure. 28.Spectra of compound NAS-16(a) HRESIMS of compound of NAS-16 (b)^{1}H NMR of compound of NAS-16 (c) ^{13}C NMR of compound of NAS-16(d)HSQC of compound of NAS-16(e) HMBC of compound of NAS-16(f) ROSEY of compound of NAS-16

a

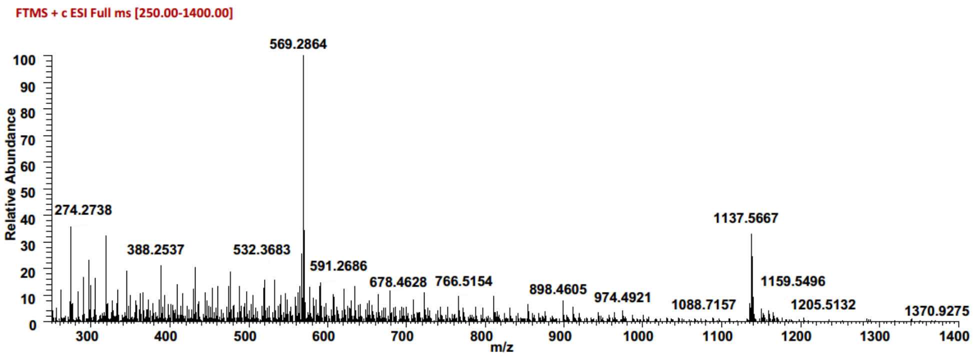
FTMS + c ESI Full ms [250.00-1400.00]
Relative Abundance
m/z
569.2864
274.2738
388.2537
532.3683
591.2686
678.4628
766.5154
898.4605
974.4921
1088.7157
1137.5667
1159.5496
1205.5132
1370.9275

b

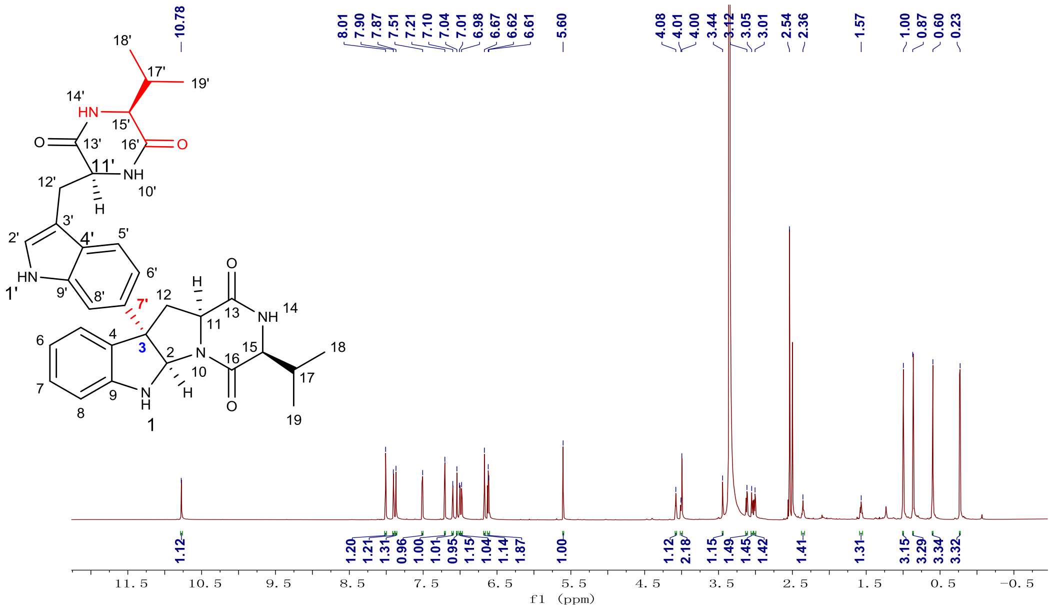
f1 (ppm)

c

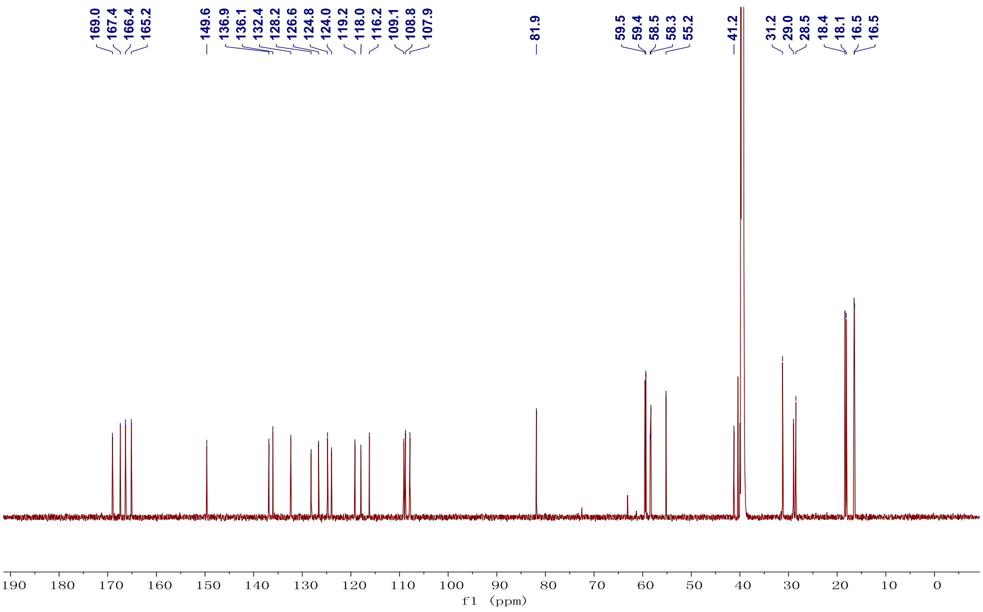
169.0
167.4
166.4
165.2
149.6
136.9
136.1
132.4
128.2
126.6
124.8
124.0
119.2
118.0
116.2
109.1
108.8
107.9
81.9
59.5
59.4
58.5
58.3
55.2
41.2
31.2
29.0
28.5
18.4
18.1
16.5
16.5
f1 (ppm)

d

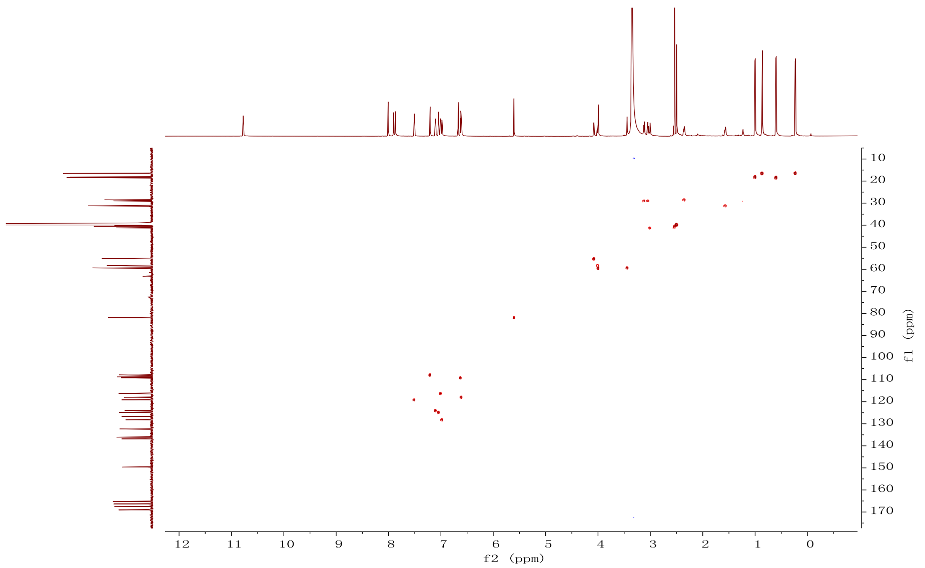

e

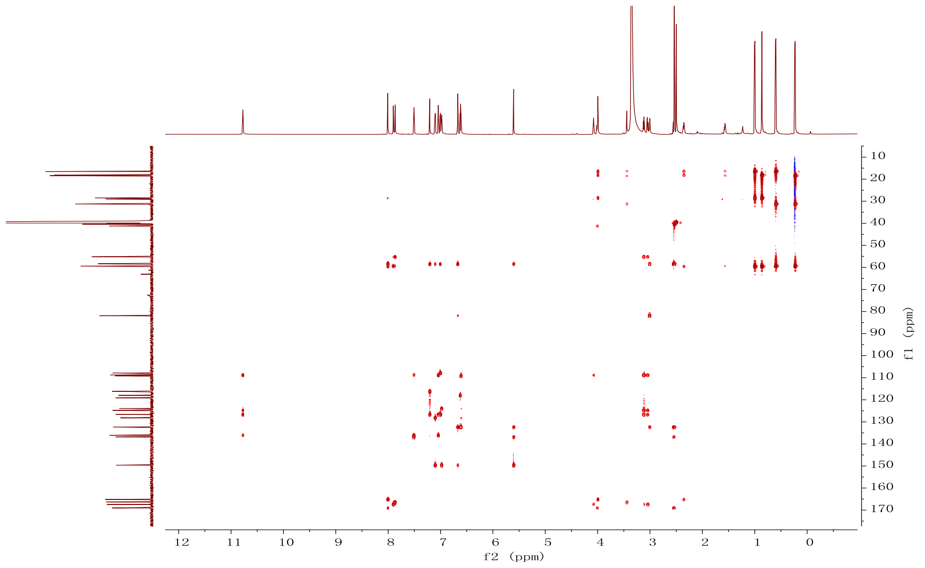

f

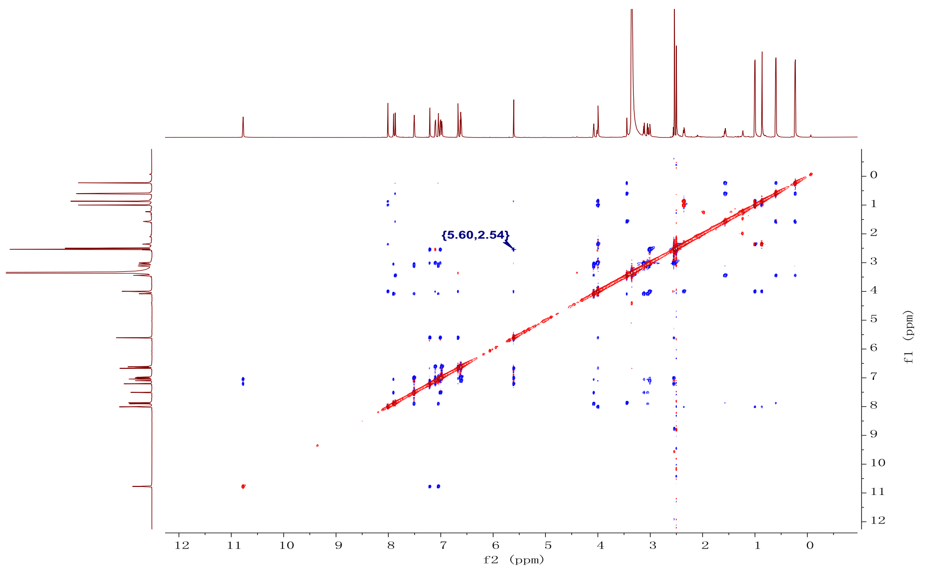

Supplementary Figure. 29.Spectra of compound NAS-17 (a) HRESIMS of compound of NAS-17(b) ^{1}H NMR of compound of NAS-17(c) ^{13}C NMR of compound of NAS-17(d) HSQC of compound of NAS-17 (e) HMBC of compound of NAS-17 (f) ROSEY of compound of NAS-17

a

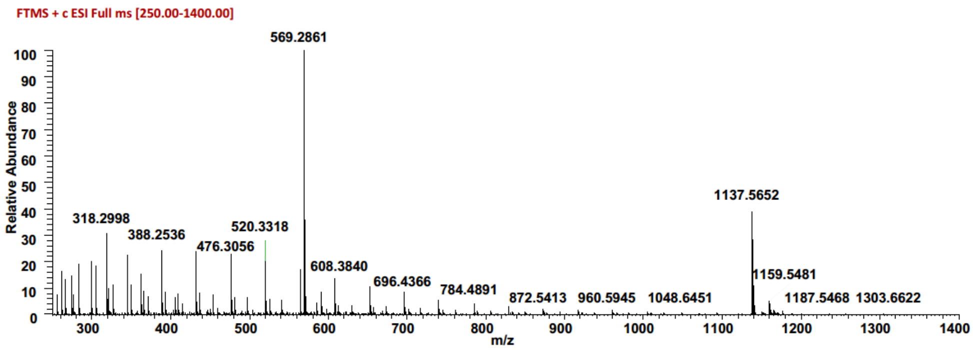
FTMS + c ESI Full ms [250.00-1400.00]
569.2861
318.2998
388.2536
476.3056
520.3318
608.3840
696.4366
784.4891
872.5413
960.5945
1048.6451
1137.5652
1159.5481
1187.5468
1303.6622
Relative Abundance
m/z

b

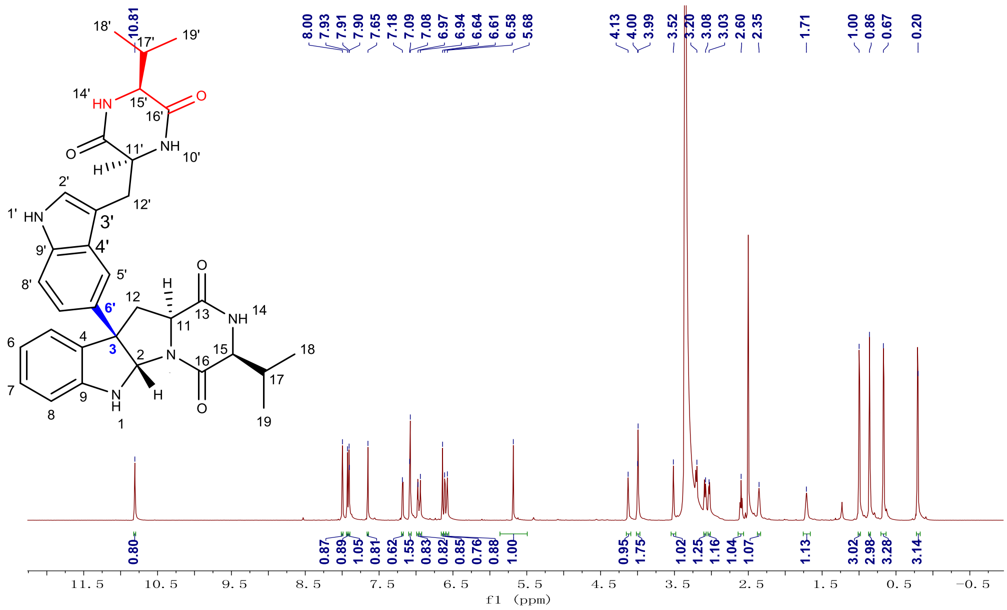
10.81
8.00 7.93 7.91 7.90 7.65 7.18 7.09 7.08 6.97 6.94 6.64 6.61 6.58 5.68
4.13 4.00 3.99 3.52 3.20 3.08 3.03 2.60 2.35 1.71 1.00 0.86 0.67 0.20
0.80 0.87 0.89 1.05 0.81 0.62 1.55 0.83 0.82 0.85 0.76 0.88 1.00 0.95 1.75 1.02 1.25 1.16 1.04 1.07 1.13 3.02 2.98 3.28 3.14
f1 (ppm)

c

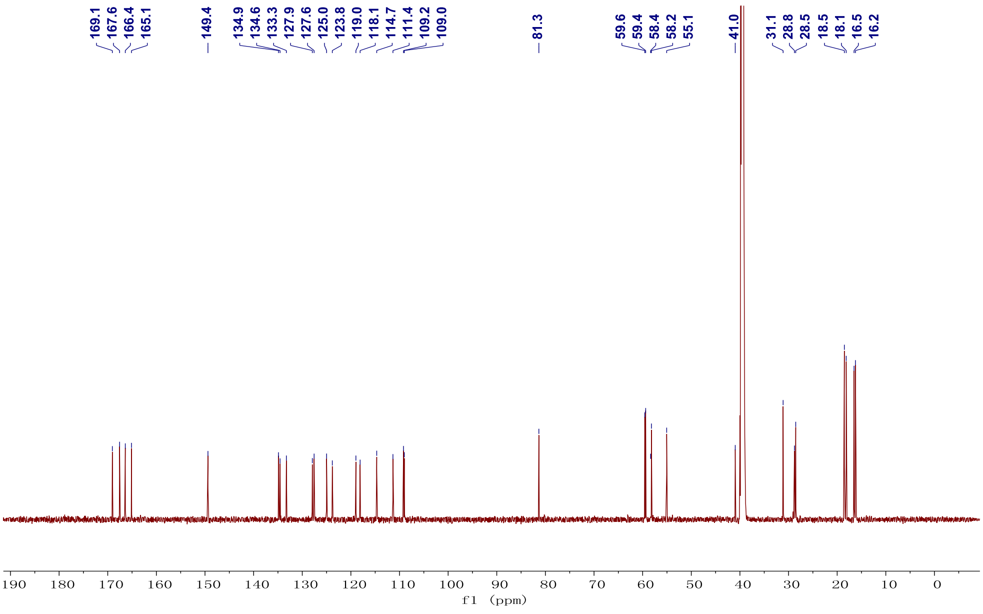
169.1 167.6 166.4 165.1
149.4
134.9 134.6 133.3 127.9 127.6 125.0 123.8 119.0 118.1 114.7 111.4 109.2 109.0
81.3
59.6 59.4 58.4 58.2 55.1
41.0
31.1 28.8 28.5 18.5 18.1 16.5 16.2
f1 (ppm)

d

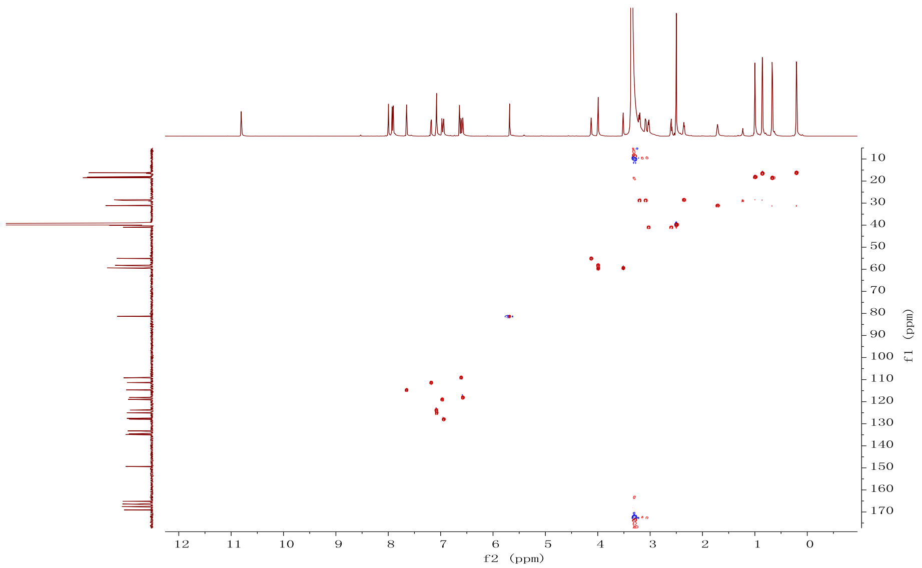

e

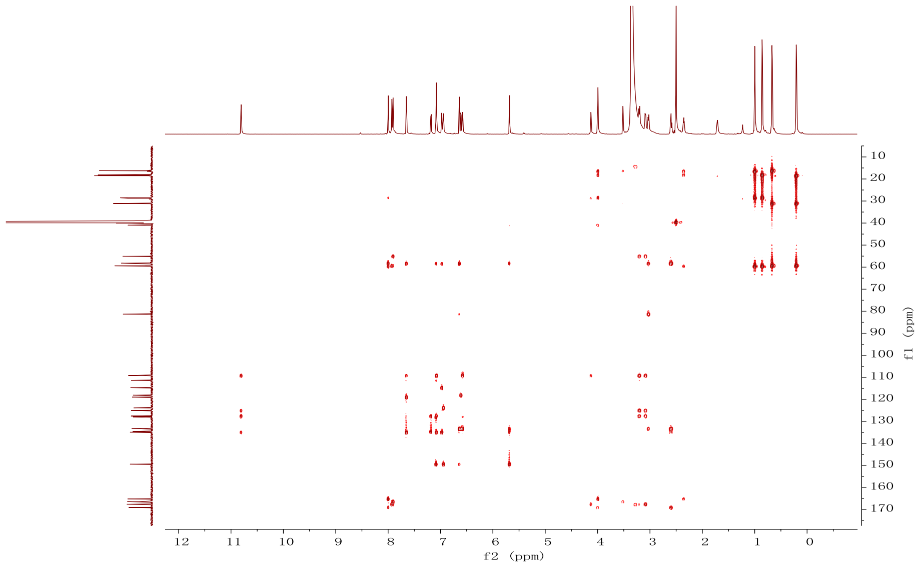

f

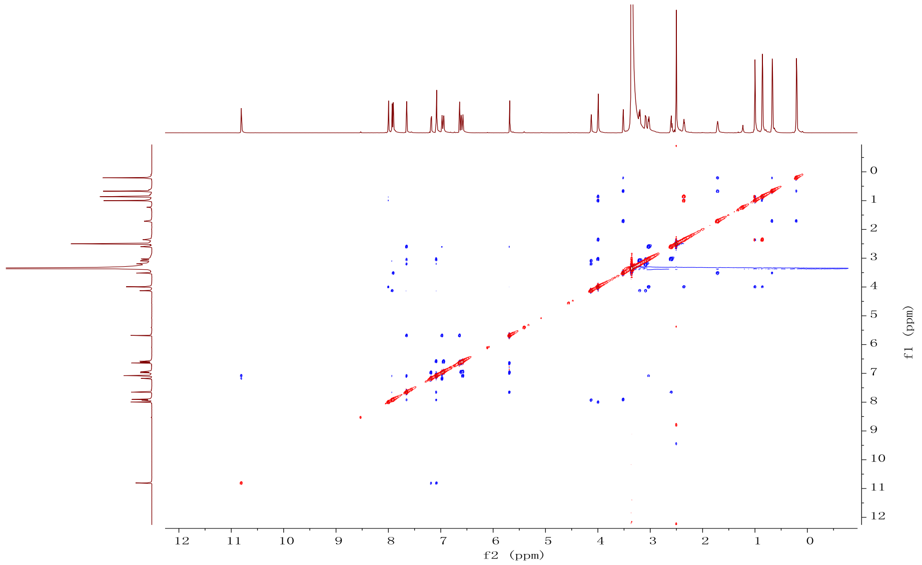

Supplementary Figure. 30.Spectra of compound NAS-18 (a) HRESIMS of compound of NAS-18(b) ^{1}H NMR of compound of NAS-18(c) ^{13}C NMR of compound of NAS-18(d) HSQC of compound of NAS-18 (e) HMBC of compound of NAS-18 (f) ROSEY of compound of NAS-18

a

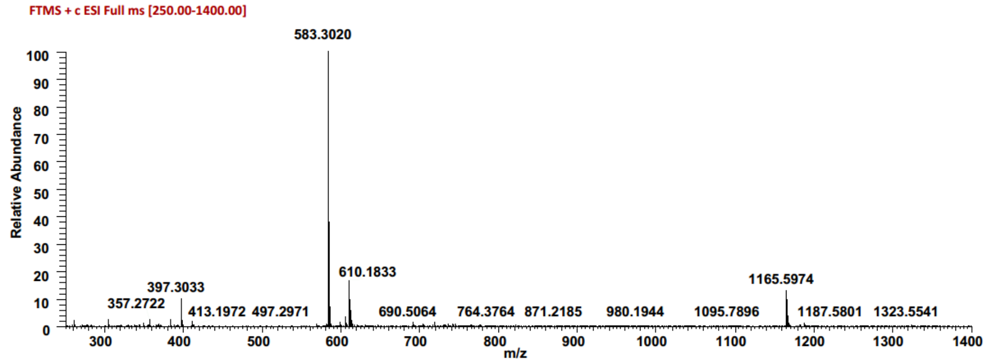

FTMS + c ESI Full ms [250.00-1400.00]
583.3020
610.1833
1165.5974
397.3033
357.2722
413.1972
497.2971
690.5064
764.3764
871.2185
980.1944
1095.7896
1187.5801
1323.5541
Relative Abundance
m/z

b

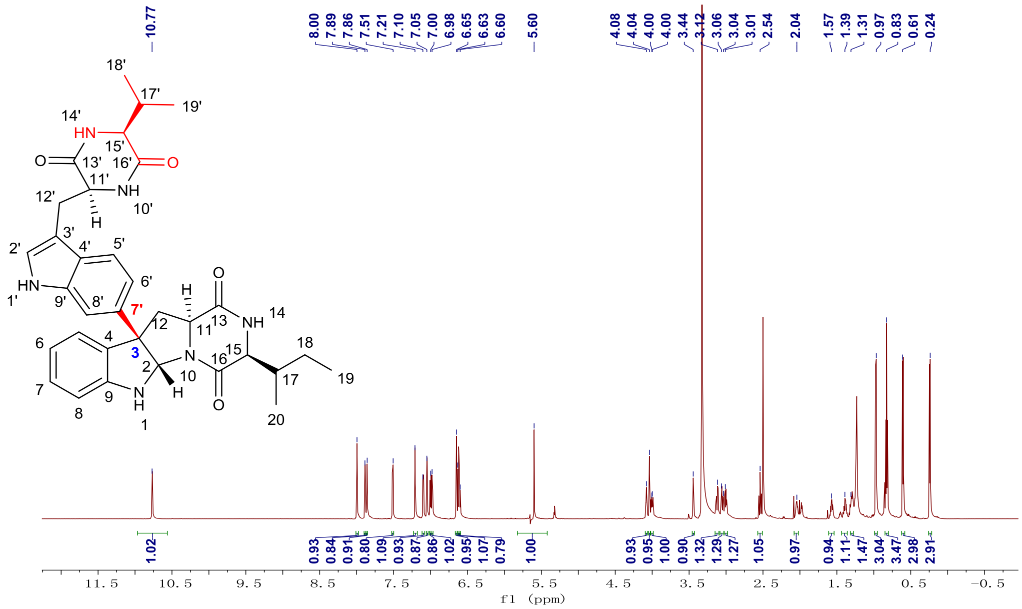

10.77
8.00 7.89 7.86 7.51 7.21 7.10 7.05 7.00 6.98 6.65 6.63 6.60
5.60
4.08 4.04 4.00 4.00 3.44 3.12 3.06 3.04 3.01 2.54
2.04
1.57 1.39 1.31 0.97 0.83 0.61 0.24
1.02
0.93 0.84 0.91 0.80 1.09 0.93 0.87 0.86 1.02 0.95 1.07 0.79
1.00
0.93 0.95 1.00 0.90 1.32 1.29 1.27 1.05
0.97
0.94 1.11 1.47 3.04 3.47 2.98 2.91
f1 (ppm)

c

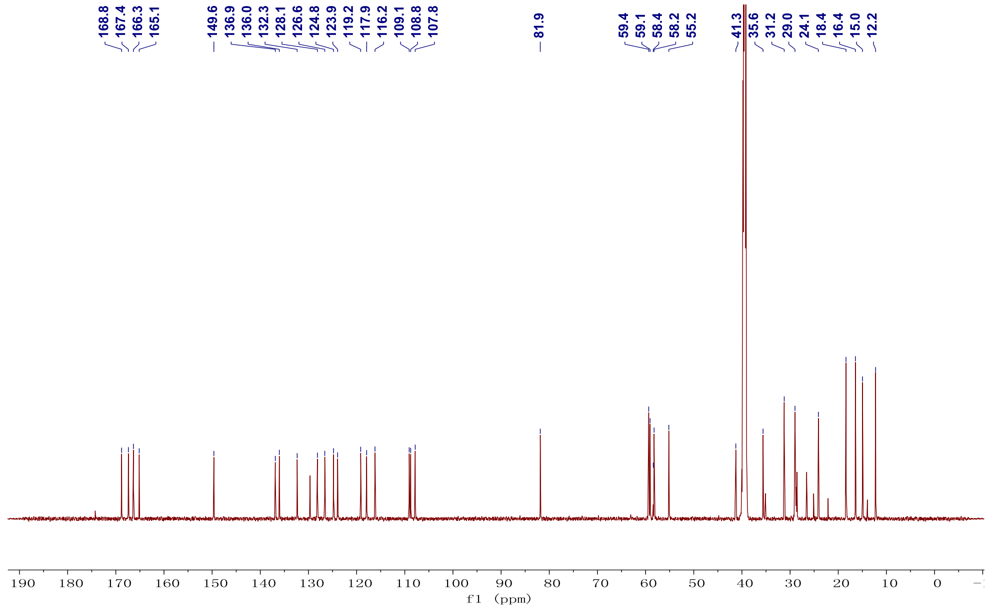

168.8 167.4 166.3 165.1
149.6 136.9 136.0 132.3 128.1 126.6 124.8 123.9 119.2 117.9 116.2 109.1 108.8 107.8
81.9
59.4 59.1 58.4 58.2 55.2
41.3 35.6 31.2 29.0 24.1 18.4 16.4 15.0 12.2
f1 (ppm)

d

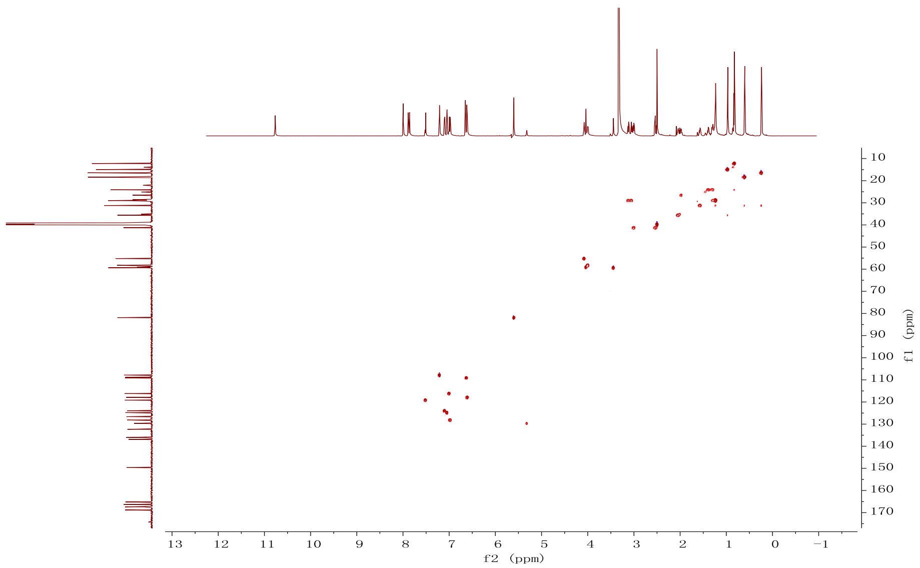

e

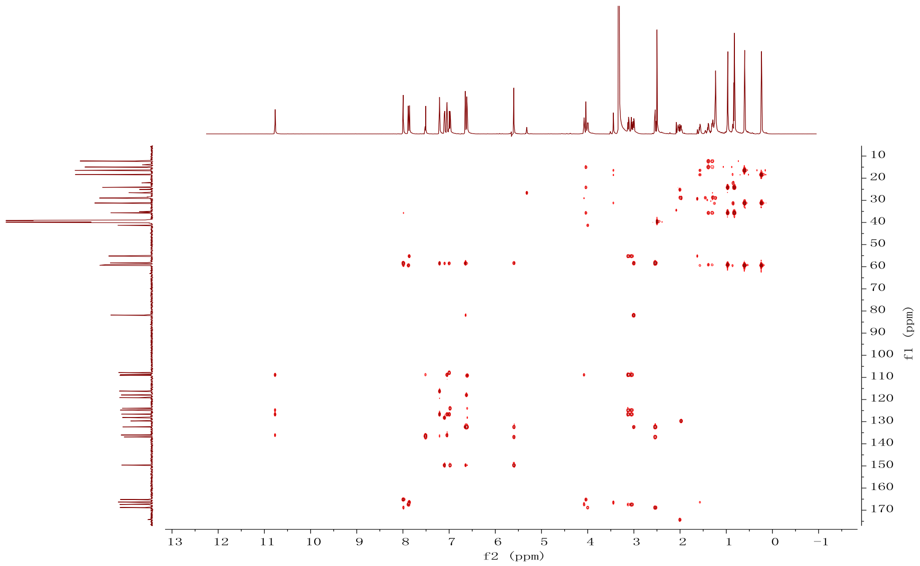

f

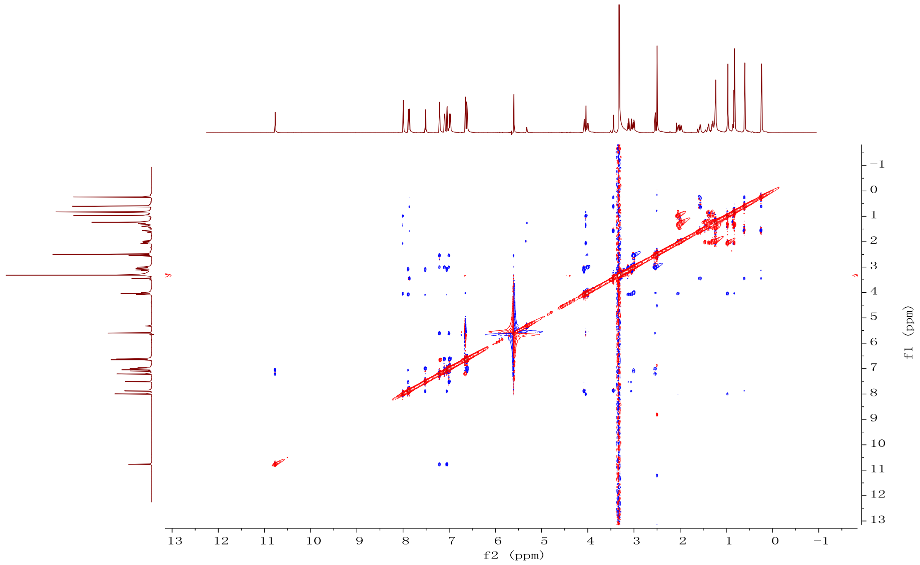

Supplementary Figure. 31.Spectra of compound NAS-19 (a) HRESIMS of compound of NAS-19(b) ^{1}H NMR of compound of NAS-19(c) ^{13}C NMR of compound of NAS-19(d) HSQC of compound of NAS-19 (e) HMBC of compound of NAS-19 (f) ROSEY of compound of NAS-19

a

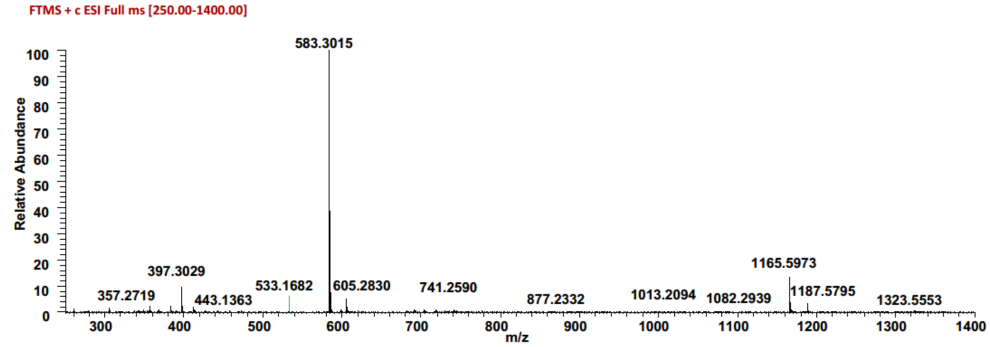
FTMS + c ESI Full ms [250.00-1400.00]
Relative Abundance
m/z
583.3015
397.3029
357.2719
443.1363
533.1682
605.2830
741.2590
877.2332
1013.2094
1082.2939
1165.5973
1187.5795
1323.5553

b

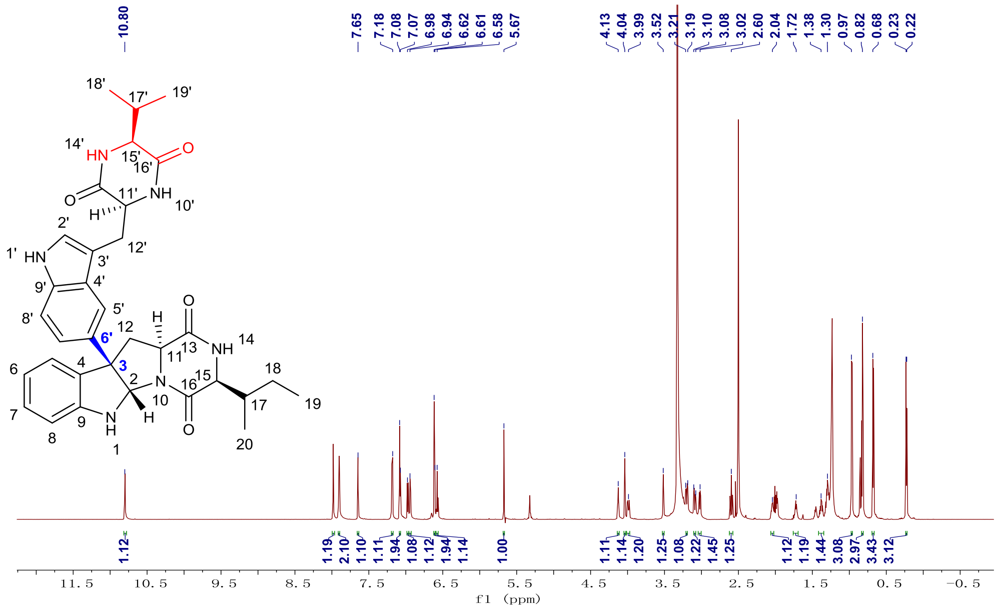
10.80
7.65
7.18
7.08
7.07
6.98
6.94
6.62
6.61
6.58
5.67
4.13
4.04
3.99
3.52
3.21
3.19
3.10
3.08
3.02
2.60
2.04
1.72
1.38
1.30
0.97
0.82
0.68
0.23
0.22
1.12
1.19
2.10
1.10
1.11
1.94
1.08
1.12
1.94
1.14
1.00
1.11
1.14
1.20
1.25
1.08
1.22
1.45
1.25
1.12
1.19
1.44
3.08
2.97
3.43
3.12
f1 (ppm)

c

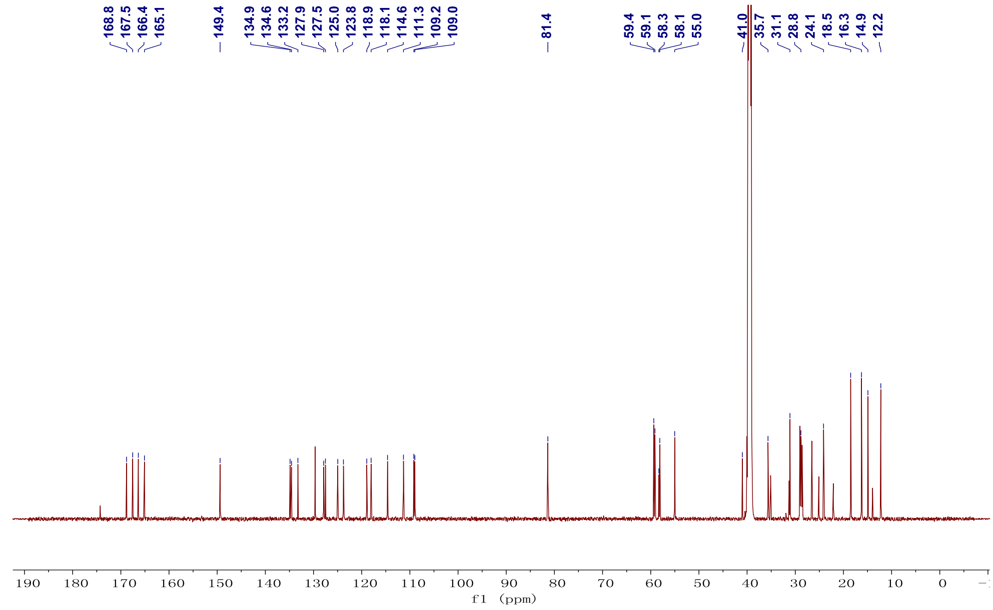
168.8
167.5
166.4
165.1
149.4
134.9
134.6
133.2
127.9
127.5
125.0
123.8
118.9
118.1
114.6
111.3
109.2
109.0
81.4
59.4
59.1
58.3
58.1
55.0
41.0
35.7
31.1
28.8
24.1
18.5
16.3
14.9
12.2
f1 (ppm)

d

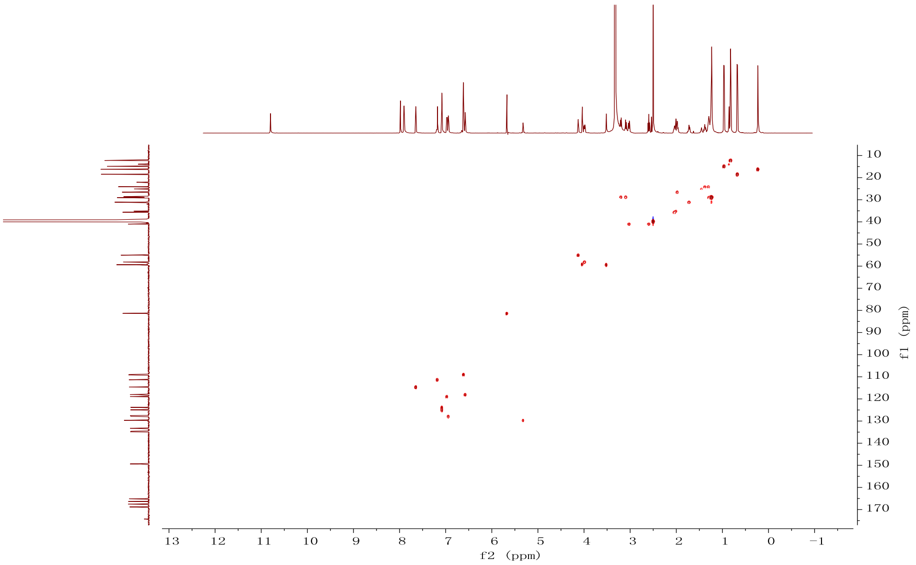

e

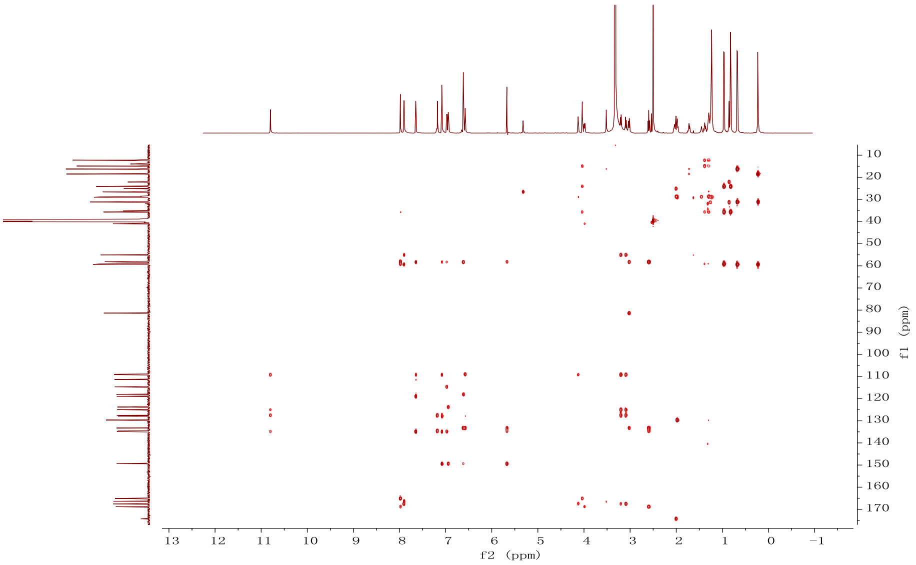

f

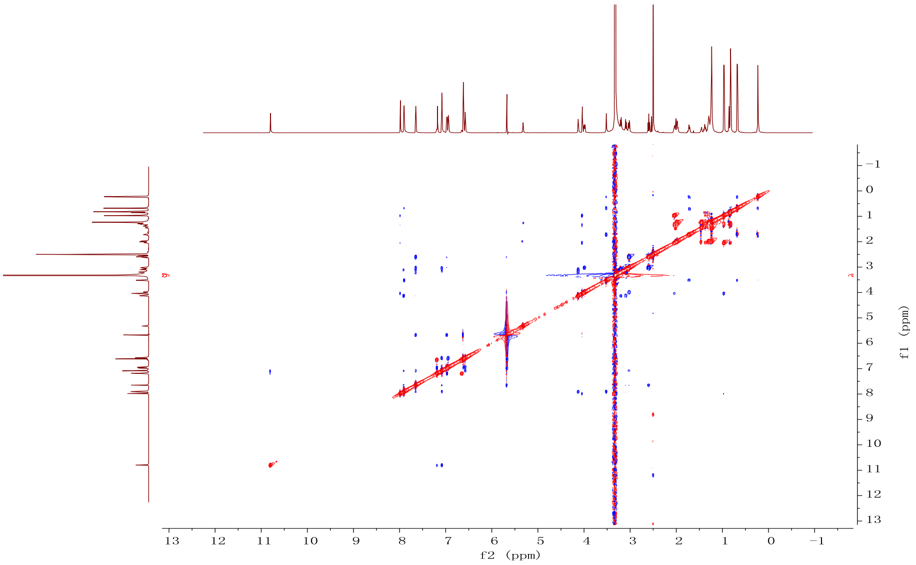

Supplementary Figure. 32.Spectra of compound NAS-20 (a) HRESIMS of compound of NAS-20(b) ^{1}H NMR of compound of NAS-20(c) ^{13}C NMR of compound of NAS-20(d) HSQC of compound of NAS-20 (e) HMBC of compound of NAS-20 (f) ROSEY of compound of NAS-20

a

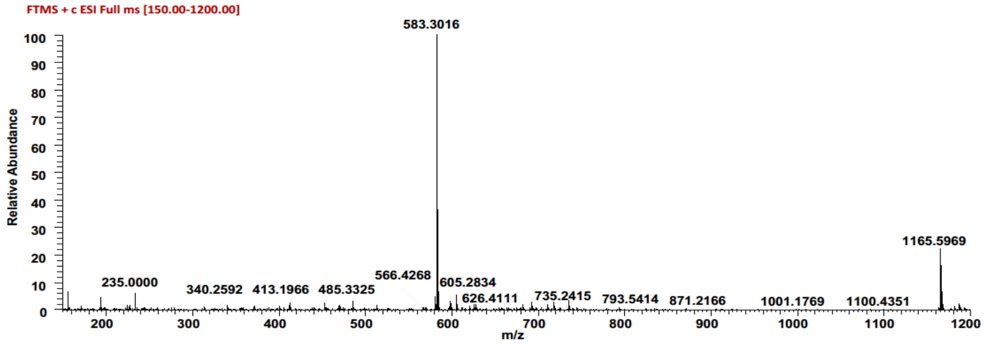
FTMS + c ESI Full ms [150.00-1200.00]
583.3016
1165.5969
235.0000
340.2592
413.1966
485.3325
566.4268
605.2834
626.4111
735.2415
793.5414
871.2166
1001.1769
1100.4351
Relative Abundance
m/z

b

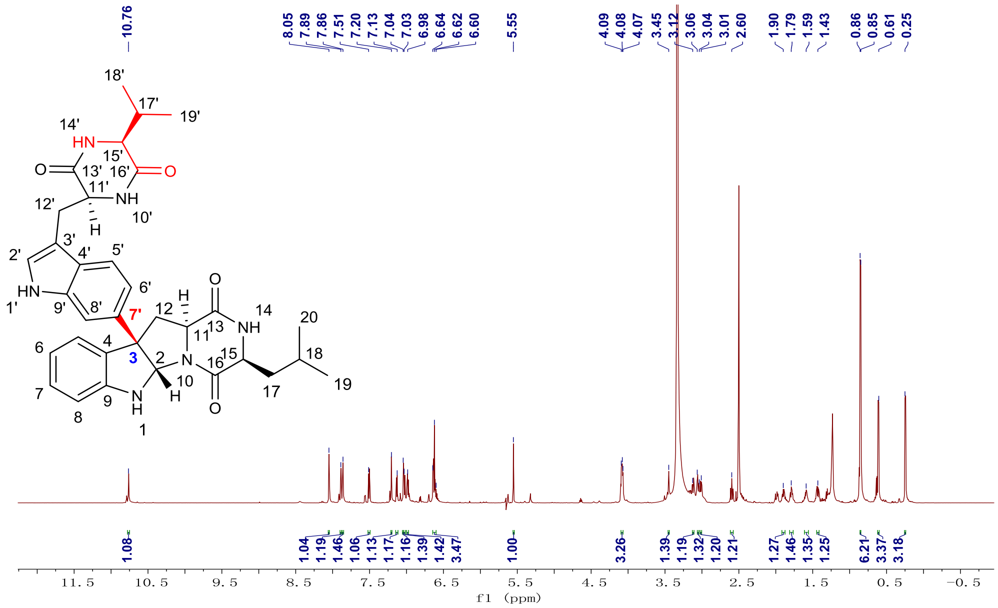
f1 (ppm)

c

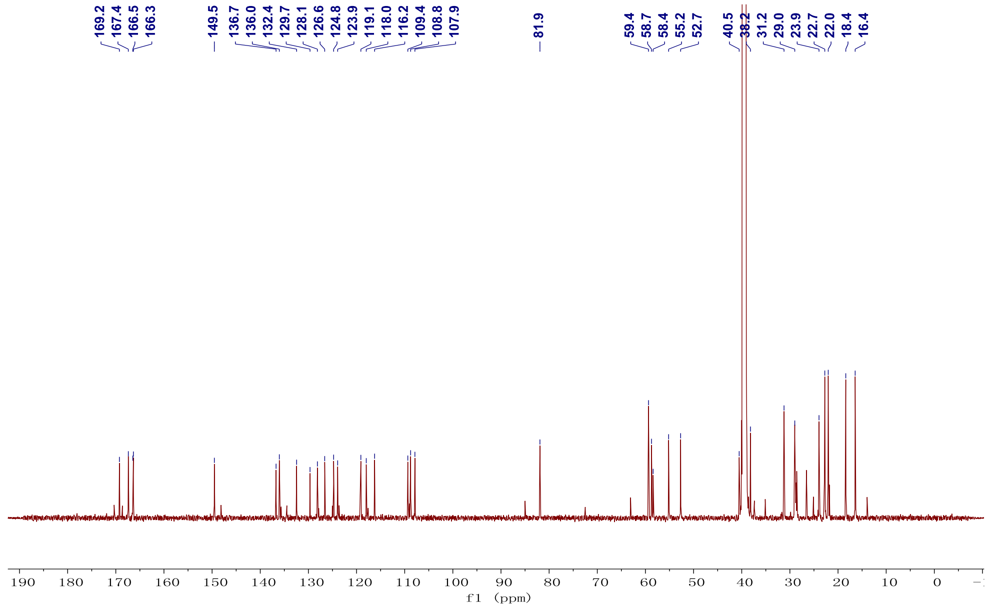
f1 (ppm)

d

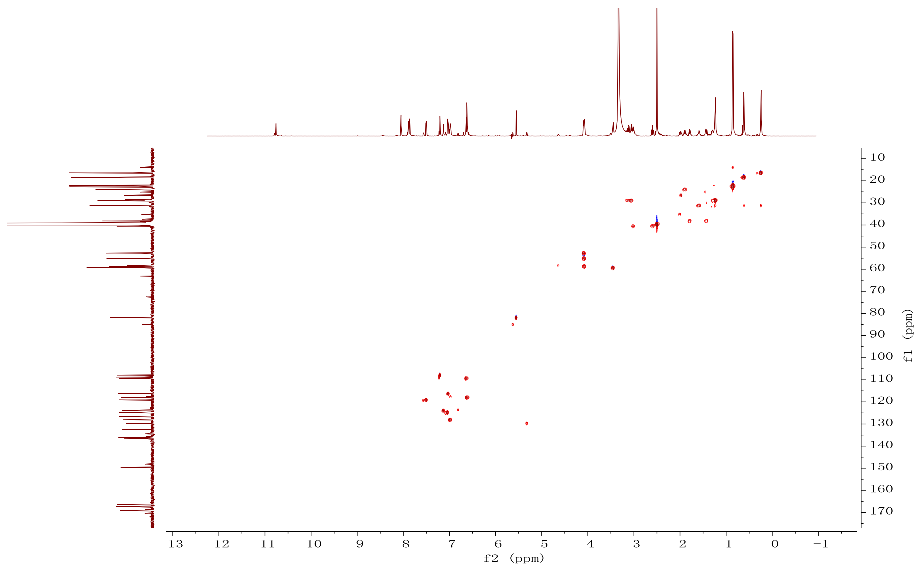

e

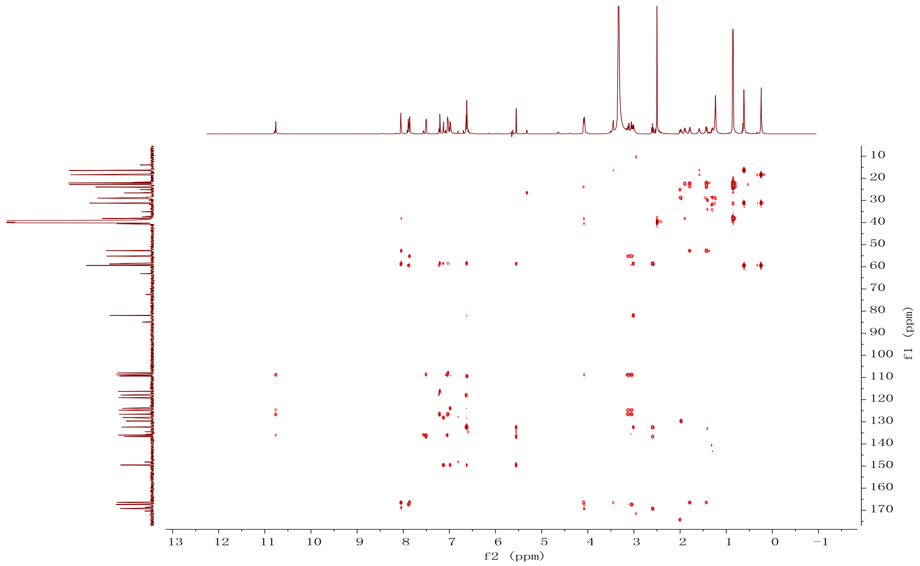

f

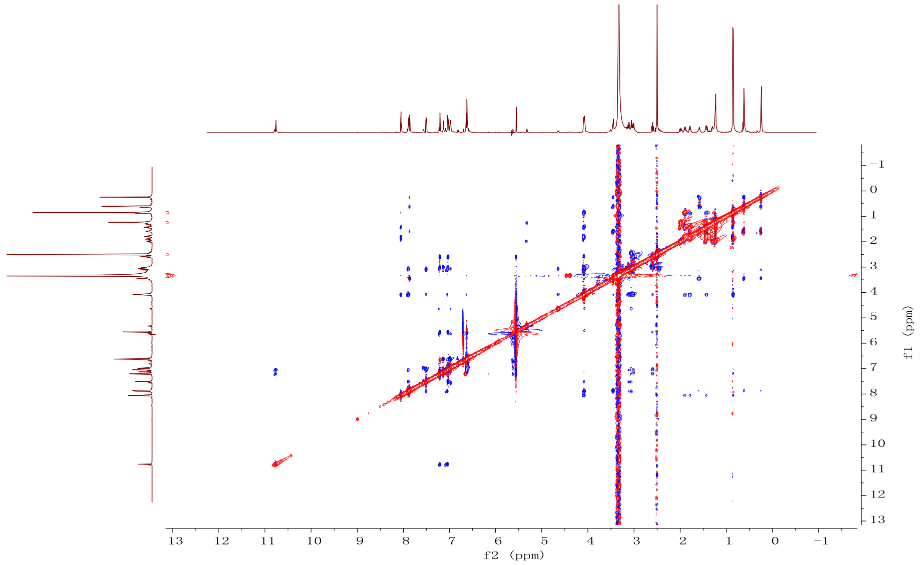

Supplementary Figure. 33.Spectra of compound NAS-21 (a) HRESIMS of compound of NAS-21(b) ^{1}H NMR of compound of NAS-21(c) ^{13}C NMR of compound of NAS-21(d) HSQC of compound of NAS-21 (e) HMBC of compound of NAS-21 (f) ROSEY of compound of NAS-21

a

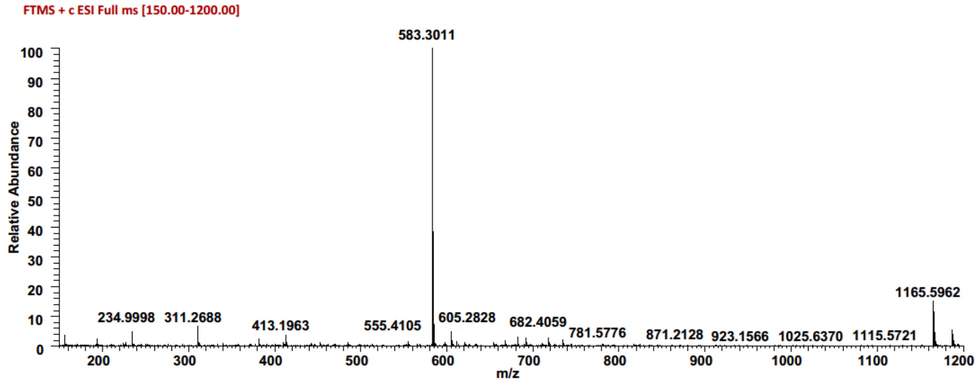
FTMS + c ESI Full ms [150.00-1200.00]
583.3011
1165.5962
Relative Abundance
m/z

b

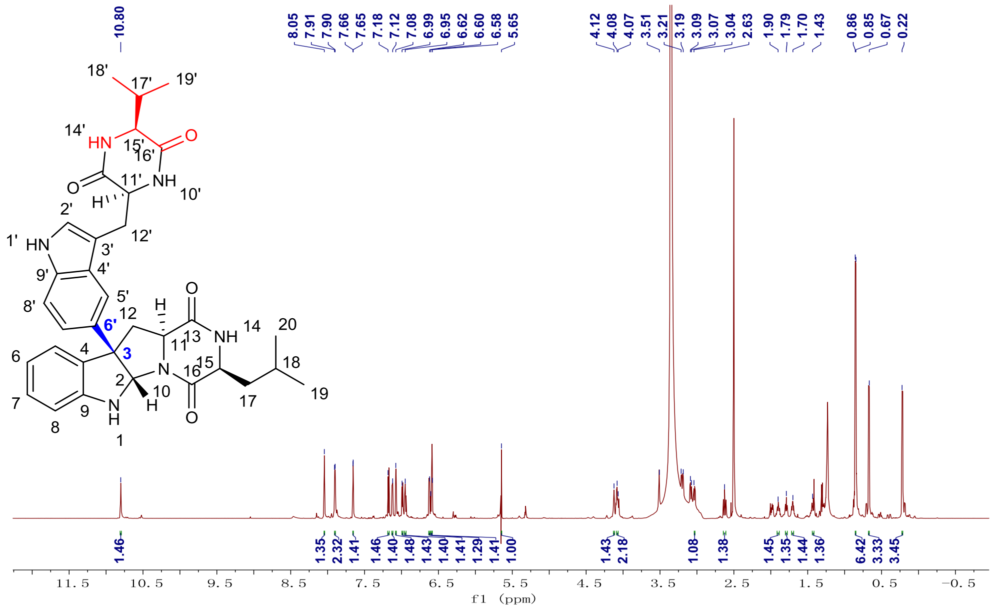
f1 (ppm)

c

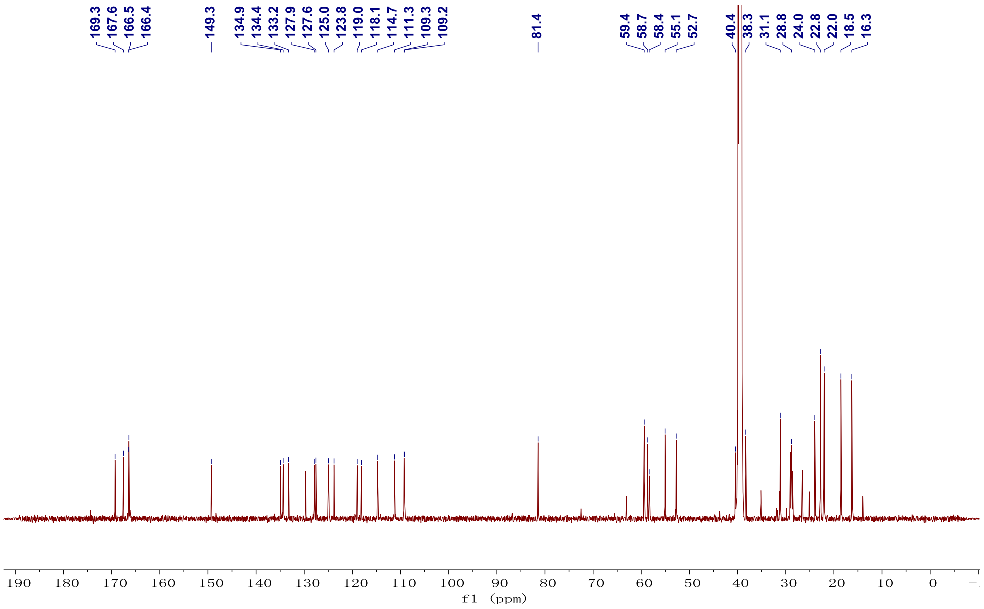
f1 (ppm)

d

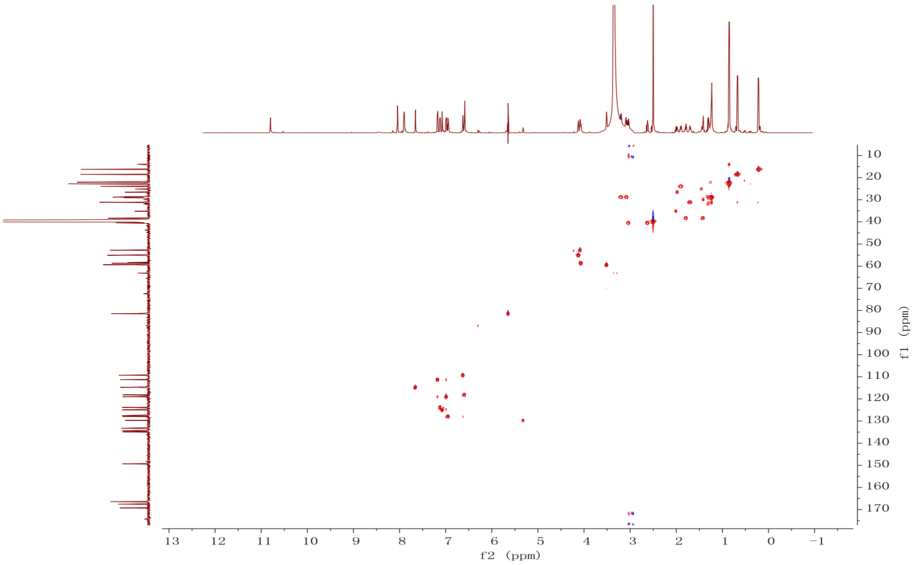

e

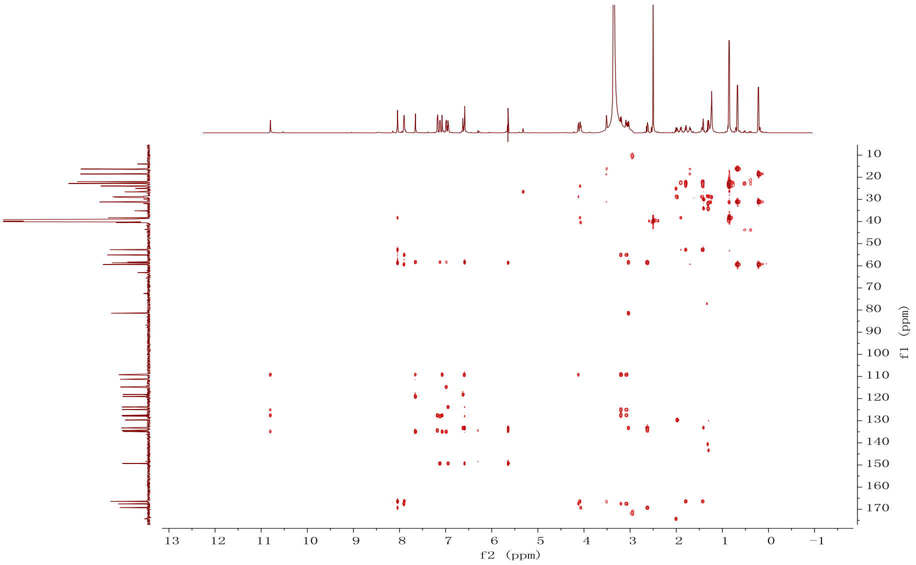

f

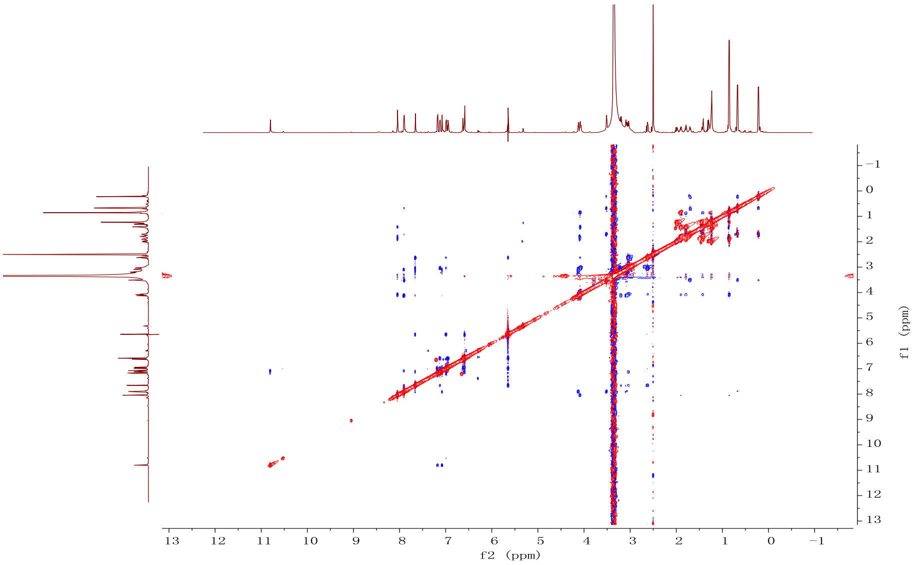

Supplementary Figure. 34.Spectra of compound NAS-22 (a) HRESIMS of compound of NAS-22(b) ^{1}H NMR of compound of NAS-22(c) ^{13}C NMR of compound of NAS-22(d) HSQC of compound of NAS-22 (e) HMBC of compound of NAS-22 (f) ROSEY of compound of NAS-22

a

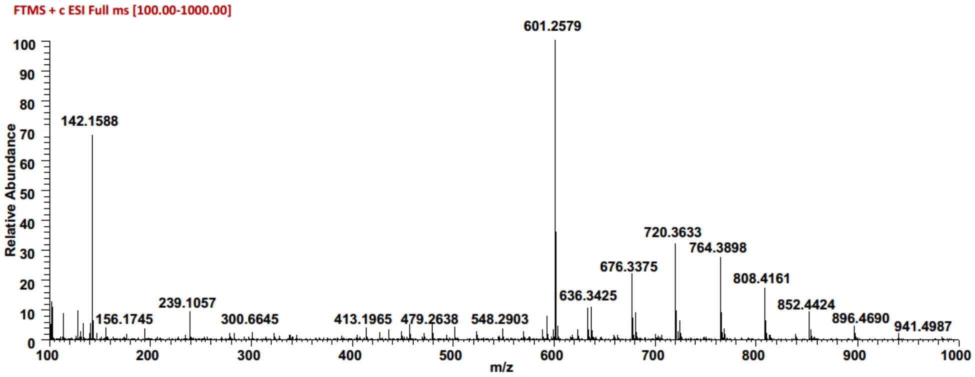
FTMS + c ESI Full ms [100.00-1000.00]
Relative Abundance
m/z
601.2579
142.1588
720.3633
764.3898
676.3375
808.4161
636.3425
852.4424
896.4690
941.4987
156.1745
239.1057
300.6645
413.1965
479.2638
548.2903

b

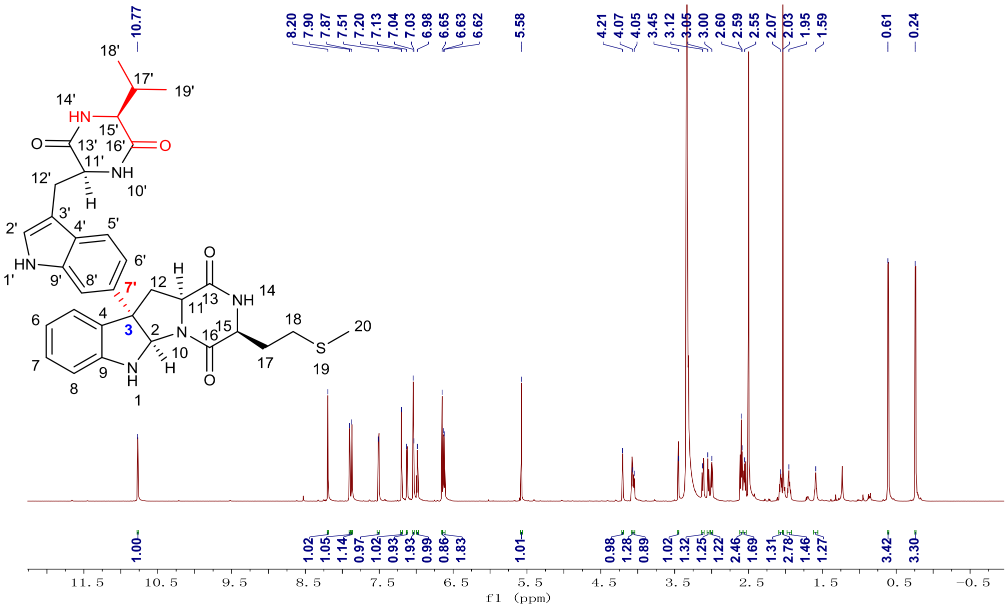
10.77
8.20 7.90 7.87 7.51 7.20 7.13 7.04 7.03 6.98 6.65 6.63 6.62
5.58
4.21 4.07 4.05 3.45 3.12 3.05 3.00 2.60 2.59 2.55 2.07 2.03 1.95 1.59
0.61
0.24
1.00
1.02 1.05 1.14 0.97 1.02 0.93 1.93 0.99 0.86 1.83
1.01
0.98 1.28 0.89 1.02 1.32 1.25 1.22 2.46 1.69 1.31 2.78 1.46 1.27
3.42 3.30
f1 (ppm)

c

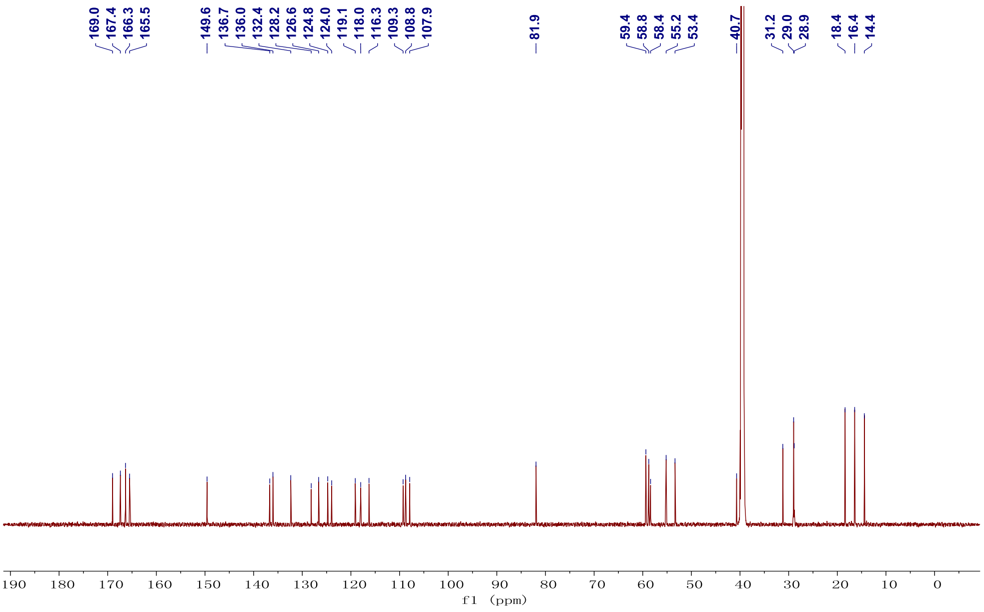
169.0 167.4 166.3 165.5
149.6 136.7 136.0 132.4 128.2 126.6 124.8 124.0 119.1 118.0 116.3 109.3 108.8 107.9
81.9
59.4 58.8 58.4 55.2 53.4
40.7
31.2 29.0 28.9
18.4 16.4 14.4
f1 (ppm)

d

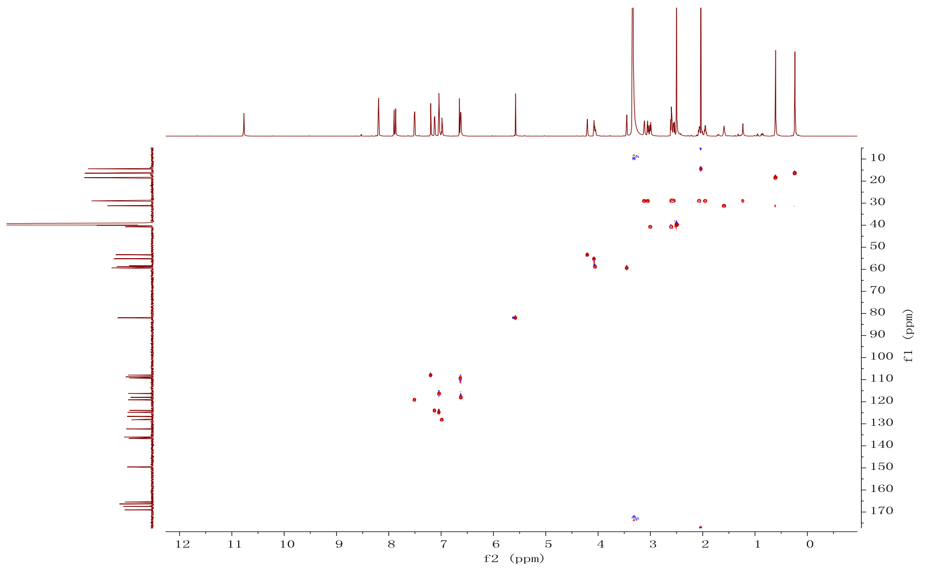

e

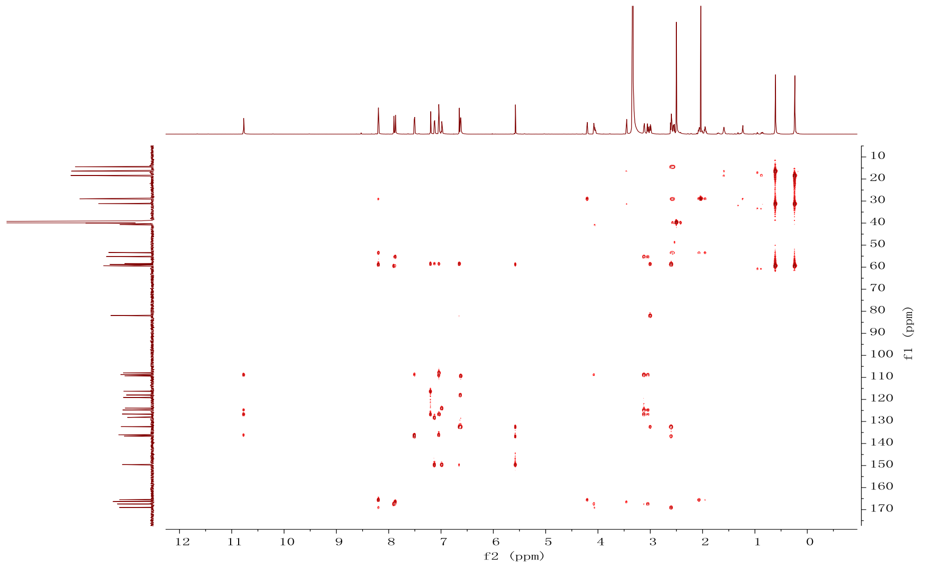

f

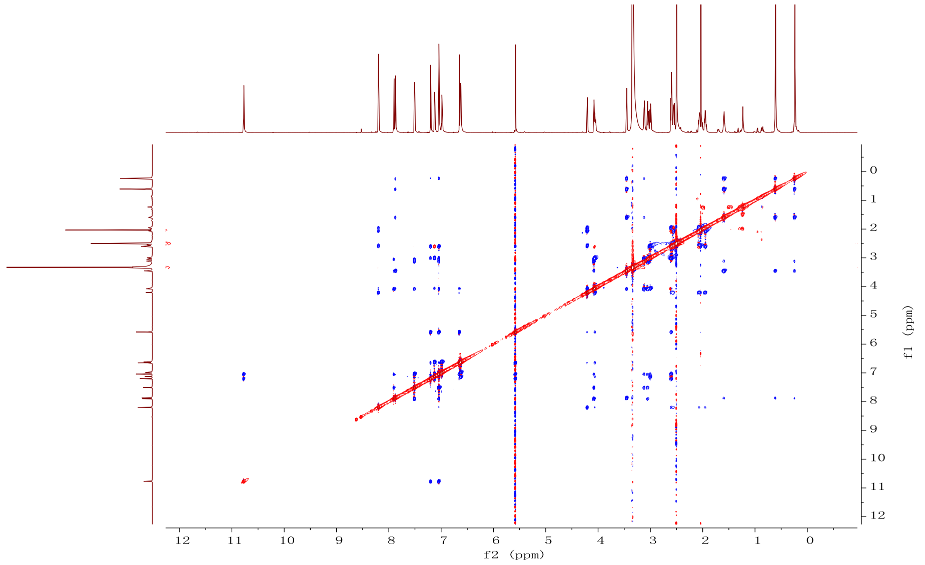

Supplementary Figure. 35.Spectra of compound NAS-23 (a) HRESIMS of compound of NAS-23(b) ^{1}H NMR of compound of NAS-23(c) ^{13}C NMR of compound of NAS-23(d) HSQC of compound of NAS-23 (e) HMBC of compound of NAS-23 (f) ROSEY of compound of NAS-23

a

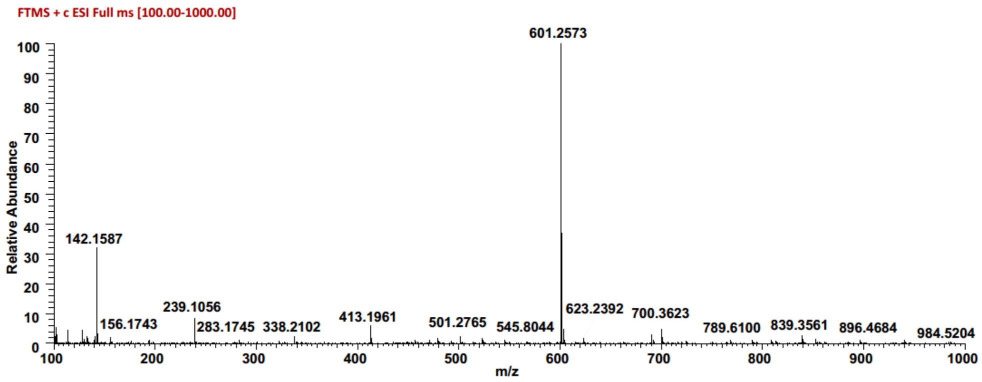

FTMS + c ESI Full ms [100.00-1000.00]
Relative Abundance
m/z
601.2573
142.1587
156.1743
239.1056
283.1745
338.2102
413.1961
501.2765
545.8044
623.2392
700.3623
789.6100
839.3561
896.4684
984.5204

b

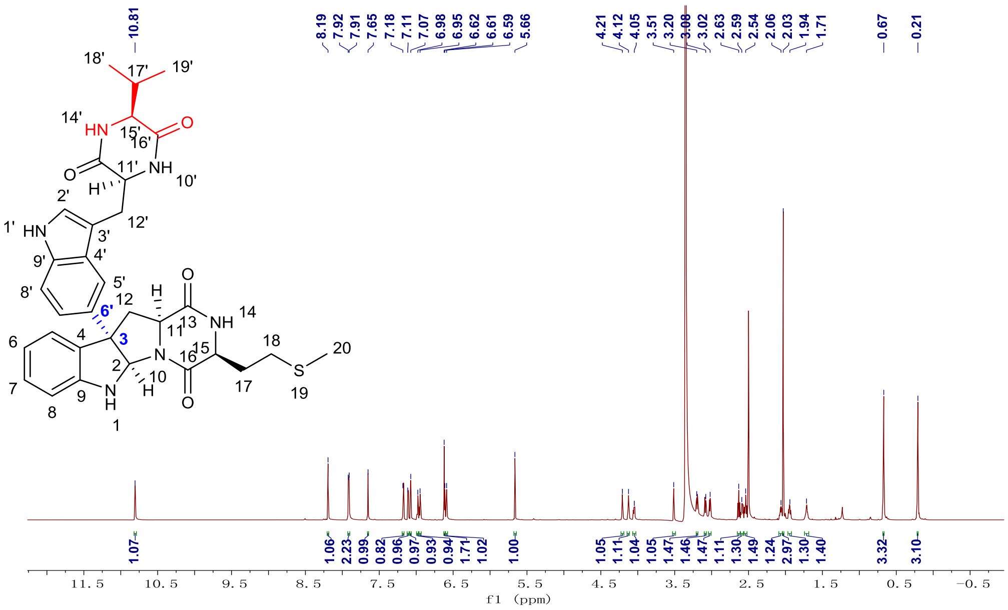

10.81
8.19
7.92
7.91
7.65
7.18
7.11
7.07
6.98
6.95
6.62
6.61
6.59
5.66
4.21
4.12
4.05
3.51
3.20
3.08
3.02
2.63
2.59
2.54
2.06
2.03
1.94
1.71
0.67
0.21
1.07
1.06
2.23
0.99
0.82
0.96
0.97
0.93
0.94
1.71
1.02
1.00
1.05
1.11
1.04
1.05
1.47
1.46
1.47
1.11
1.30
1.49
1.24
2.97
1.30
1.40
3.32
3.10
f1 (ppm)

c

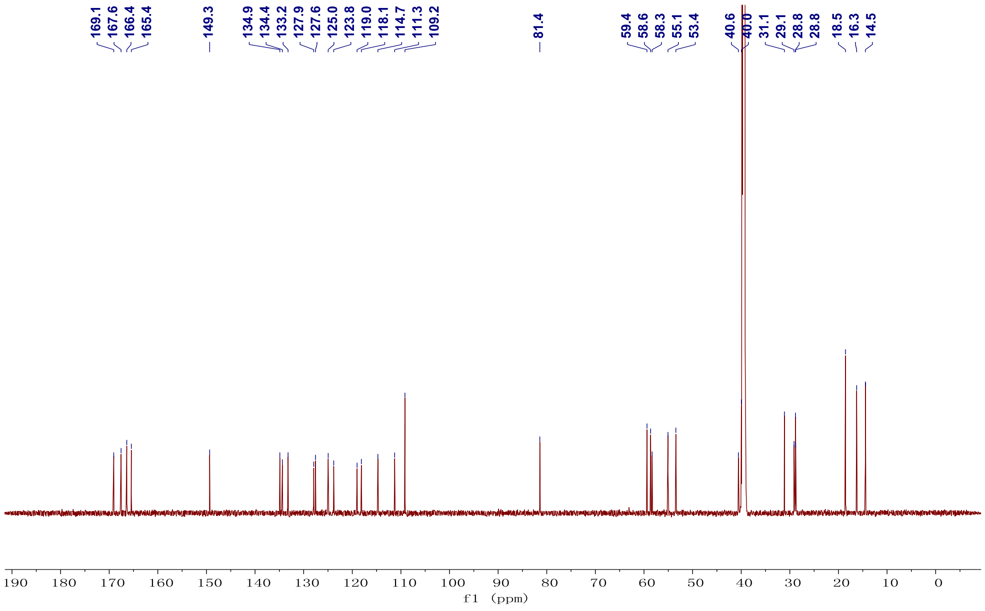

169.1
167.6
166.4
165.4
149.3
134.9
134.4
133.2
127.9
127.6
125.0
123.8
119.0
118.1
114.7
111.3
109.2
81.4
59.4
58.6
58.3
55.1
53.4
40.6
40.0
31.1
29.1
28.8
28.8
18.5
16.3
14.5
f1 (ppm)

d

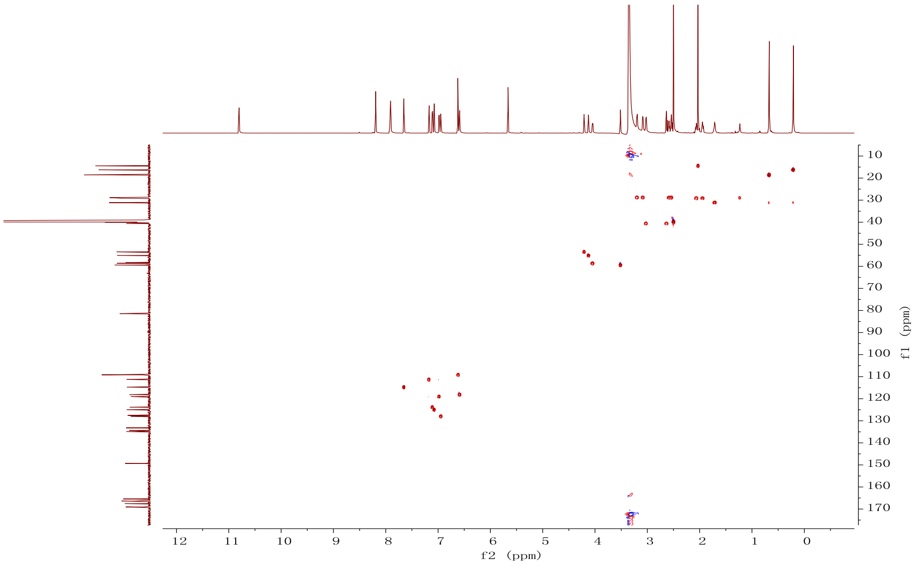

e

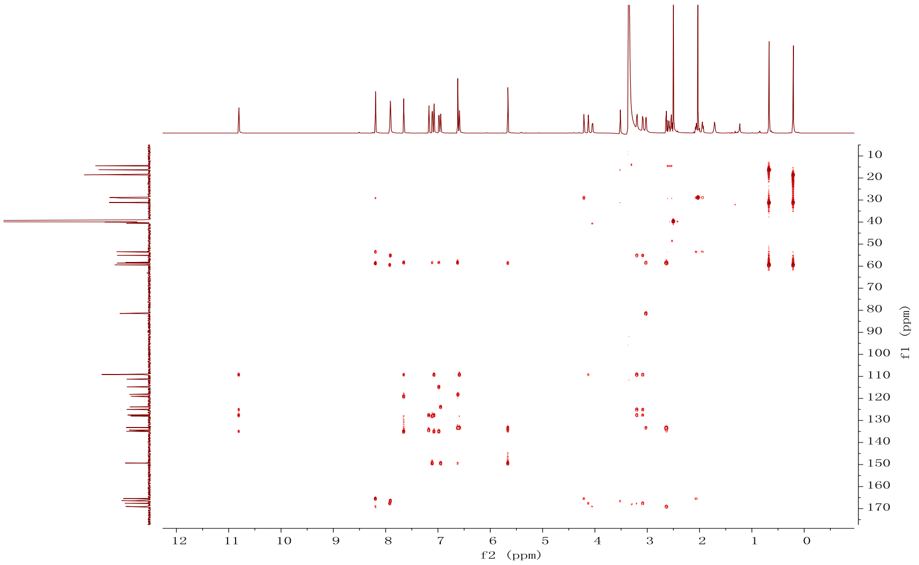

f

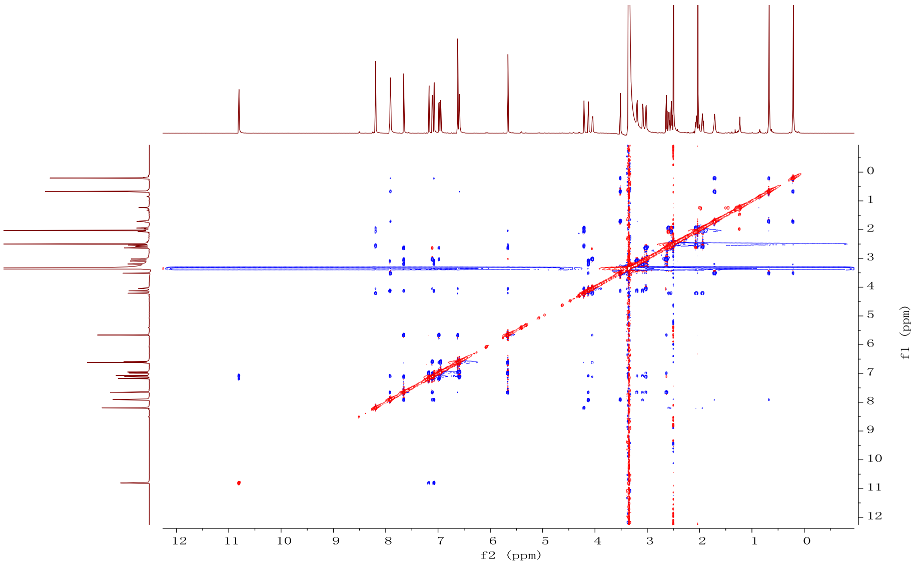

Supplementary Figure. 36.Spectra of compound NAS-24 (a) HRESIMS of compound of NAS-24(b) ^{1}H NMR of compound of NAS-24(c) ^{13}C NMR of compound of NAS-24(d) HSQC of compound of NAS-24 (e) HMBC of compound of NAS-24 (f) ROSEY of compound of NAS-24

a

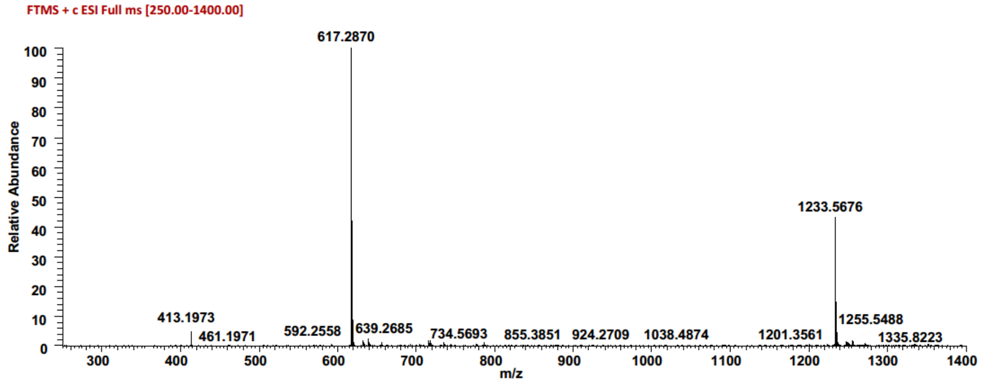
FTMS + c ESI Full ms [250.00-1400.00]
617.2870
1233.5676
413.1973
461.1971
592.2558
639.2685
734.5693
855.3851
924.2709
1038.4874
1201.3561
1255.5488
1335.8223
Relative Abundance
m/z

b

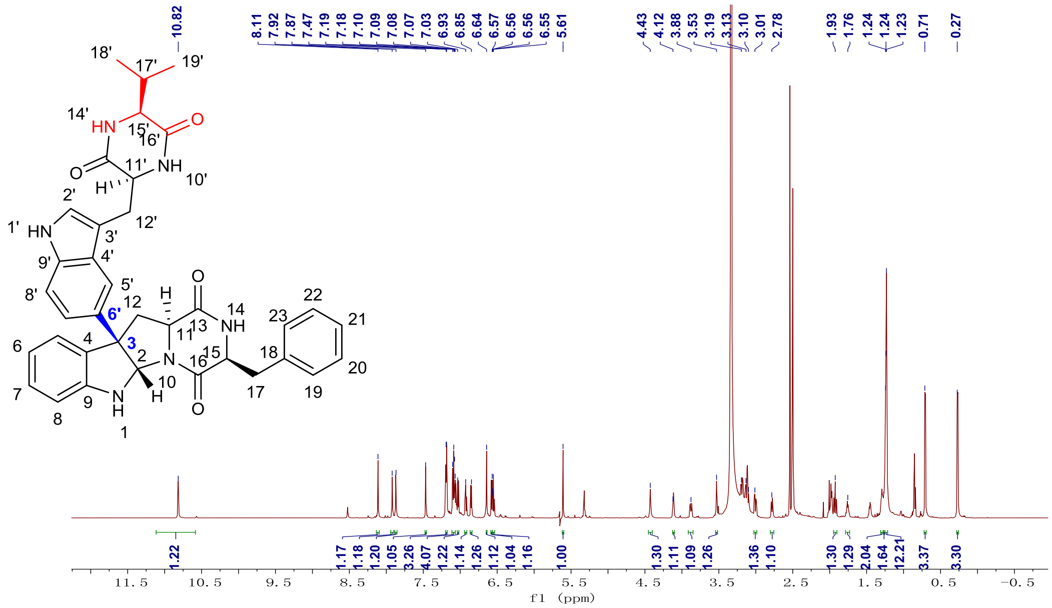
f1 (ppm)

c

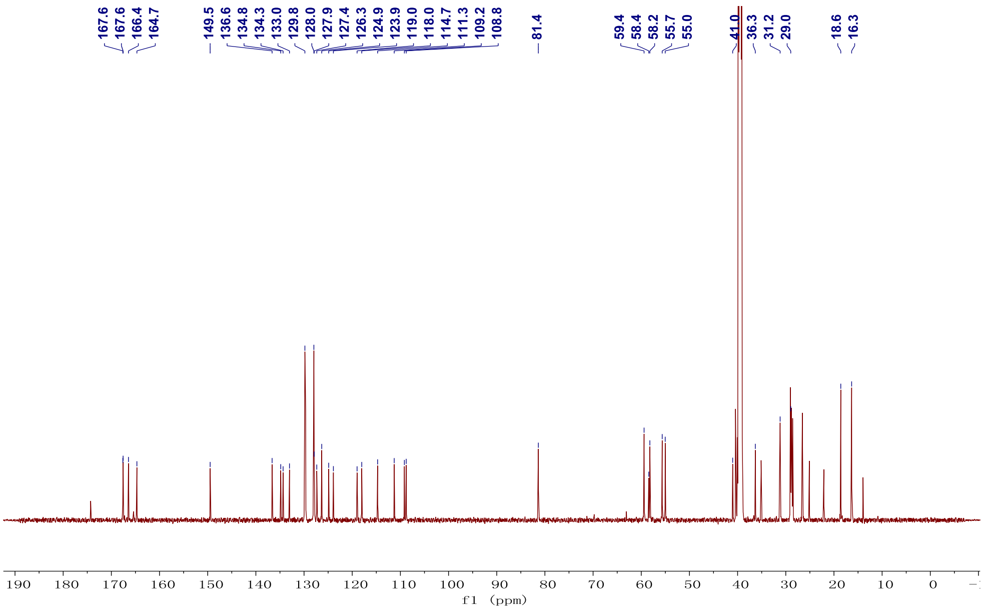
f1 (ppm)

d

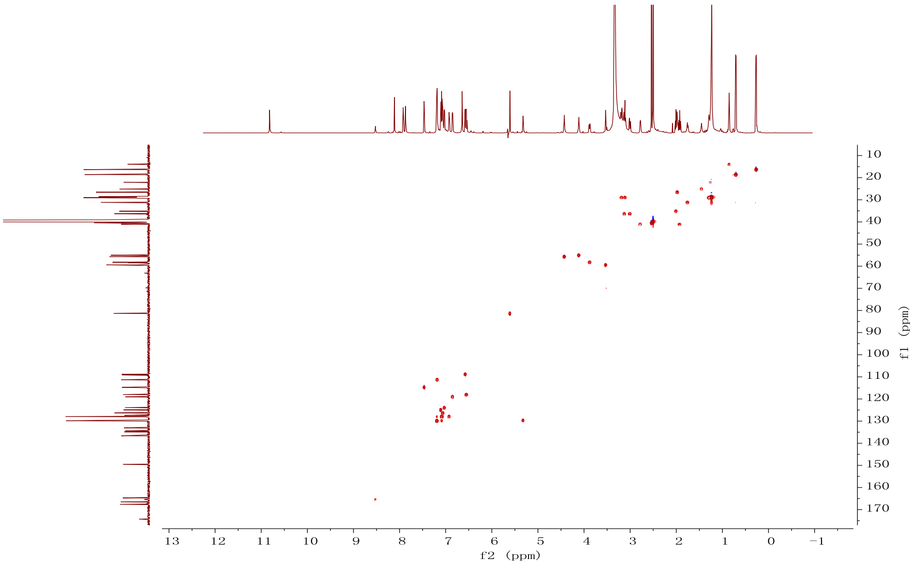

e

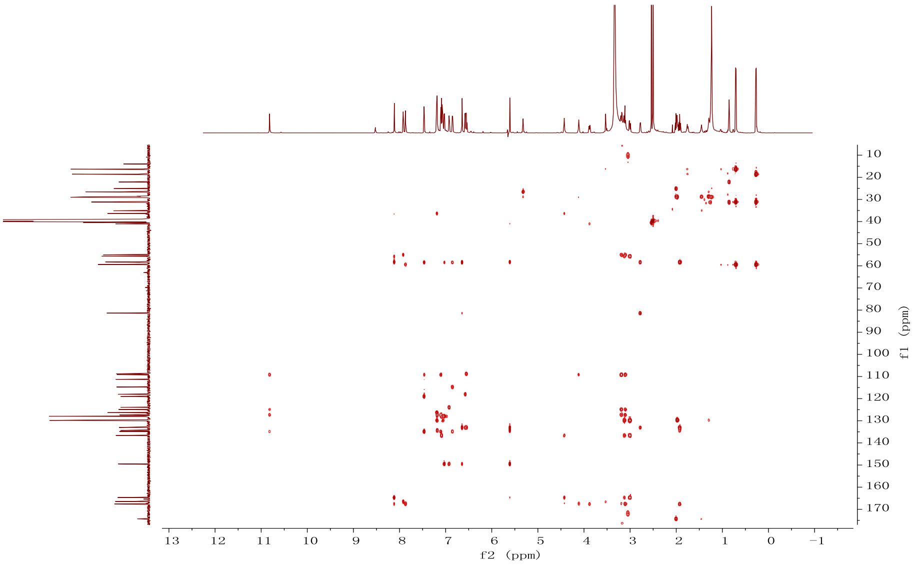

f

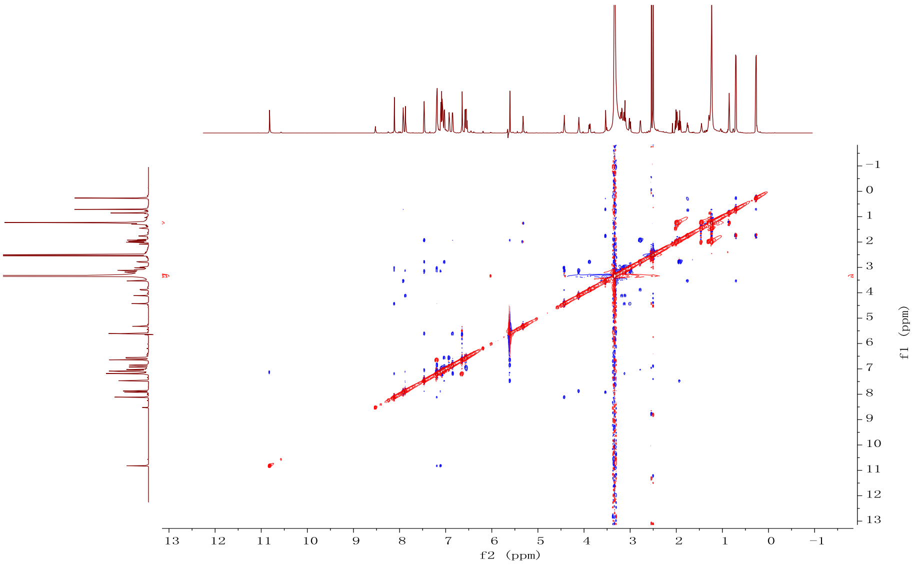

Supplementary Figure. 37.Spectra of compound NAS-25 (a) HRESIMS of compound of NAS-25(b) ^{1}H NMR of compound of NAS-25(c) ^{13}C NMR of compound of NAS-25(d) HSQC of compound of NAS-25 (e) HMBC of compound of NAS-25 (f) ROSEY of compound of NAS-25

a

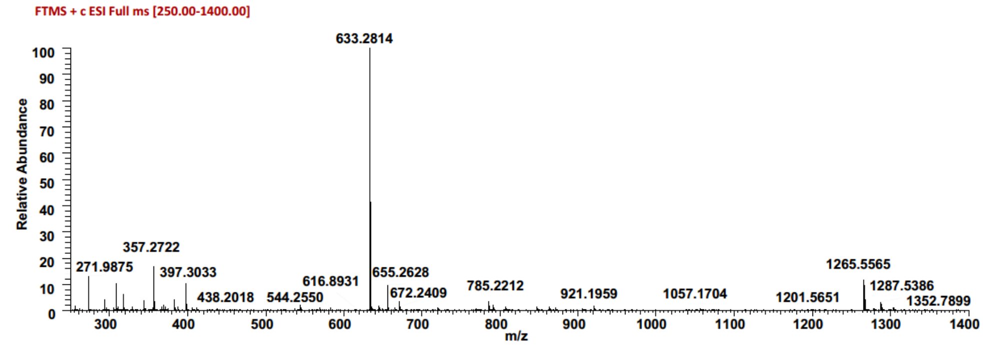
FTMS + c ESI Full ms [250.00-1400.00]
Relative Abundance
m/z
633.2814
357.2722
271.9875
397.3033
438.2018
544.2550
616.8931
655.2628
672.2409
785.2212
921.1959
1057.1704
1201.5651
1265.5565
1287.5386
1352.7899

b

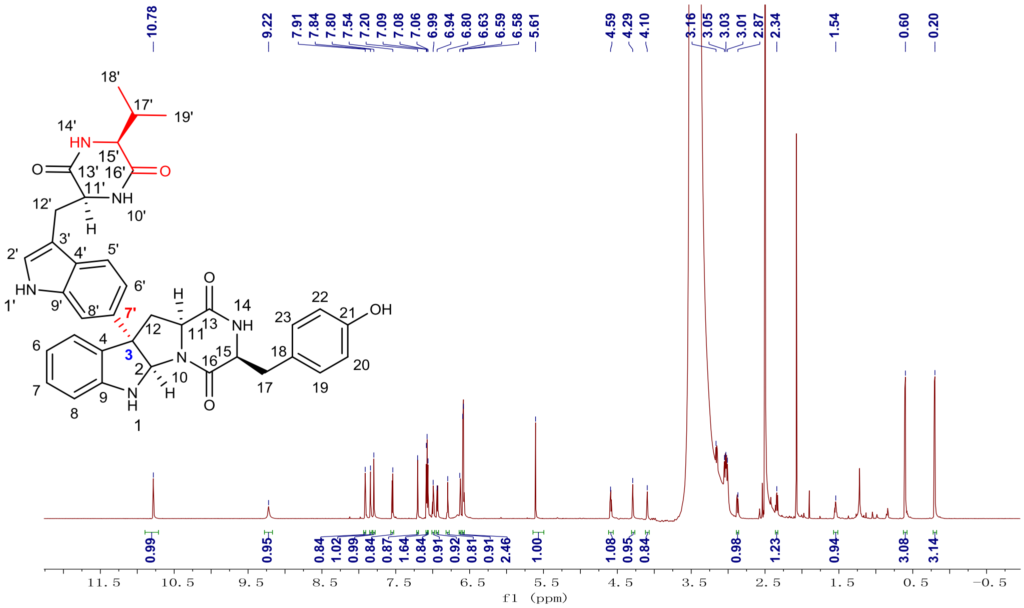
10.78
9.22
7.91
7.84
7.80
7.54
7.20
7.09
7.08
7.06
6.99
6.94
6.80
6.63
6.59
6.58
5.61
4.59
4.29
4.10
3.16
3.05
3.03
3.01
2.87
2.34
1.54
0.60
0.20
f1 (ppm)

c

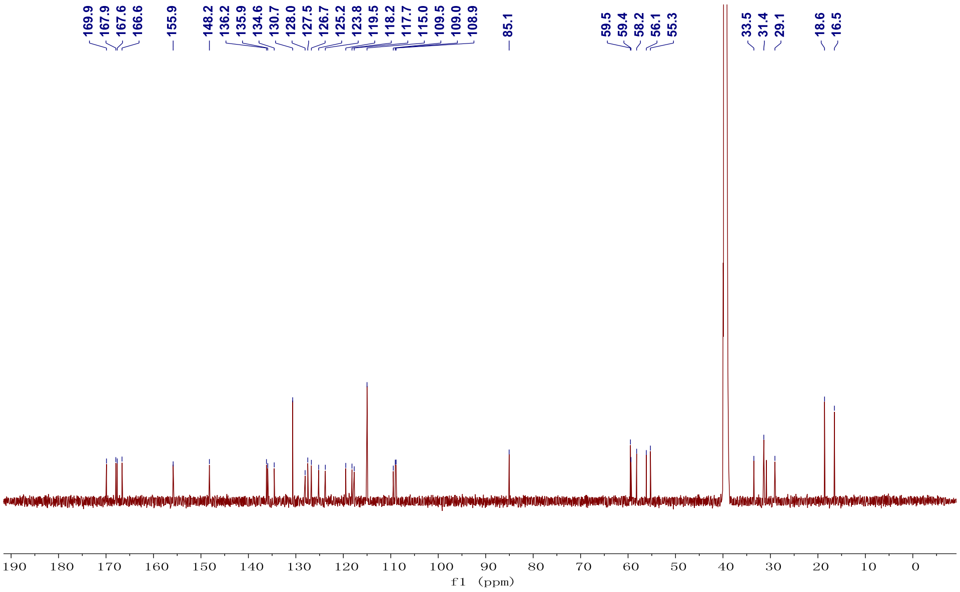
169.9
167.9
167.6
166.6
155.9
148.2
136.2
135.9
134.6
130.7
128.0
127.5
126.7
125.2
123.8
119.5
118.2
117.7
115.0
109.5
109.0
108.9
85.1
59.5
59.4
58.2
56.1
55.3
33.5
31.4
29.1
18.6
16.5
f1 (ppm)

d

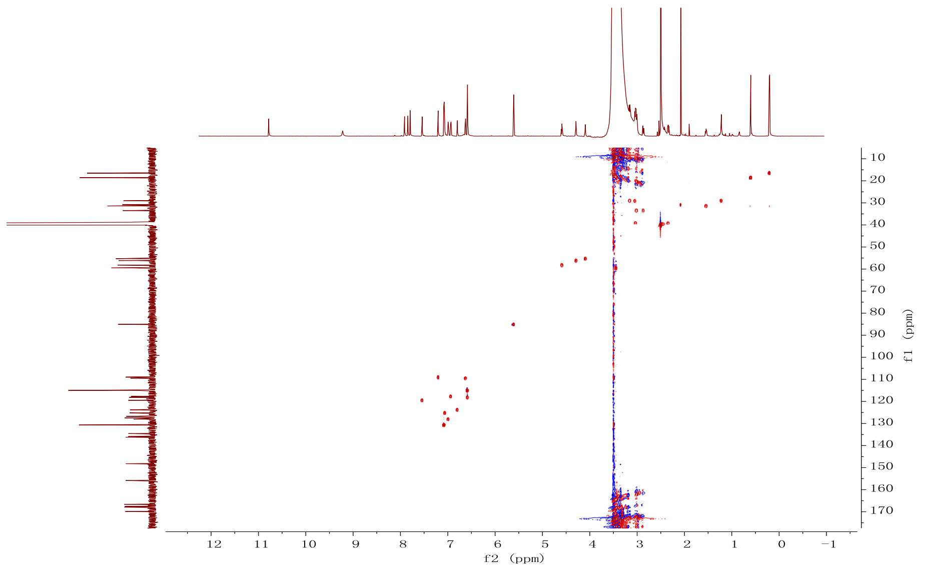

e

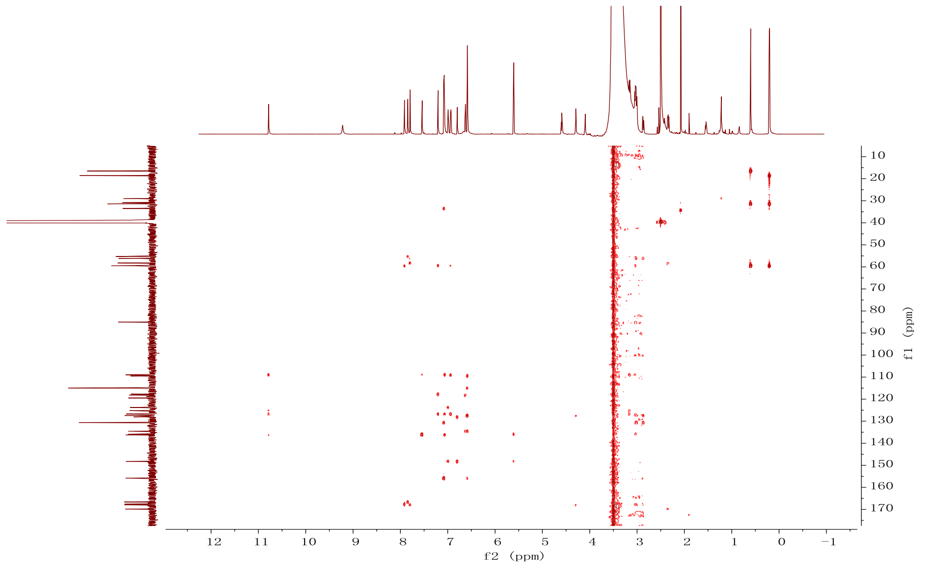

f

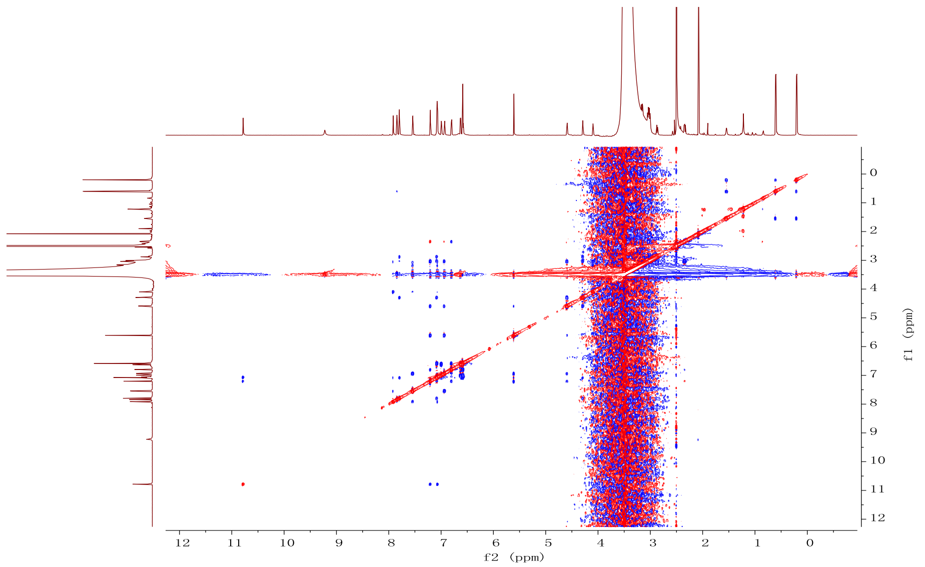

Supplementary Figure. 38.Spectra of compound NAS-26 (a) HRESIMS of compound of NAS-26(b) ^{1}H NMR of compound of NAS-26(c) ^{13}C NMR of compound of NAS-26(d) HSQC of compound of NAS-26 (e) HMBC of compound of NAS-26 (f) ROSEY of compound of NAS-26

a

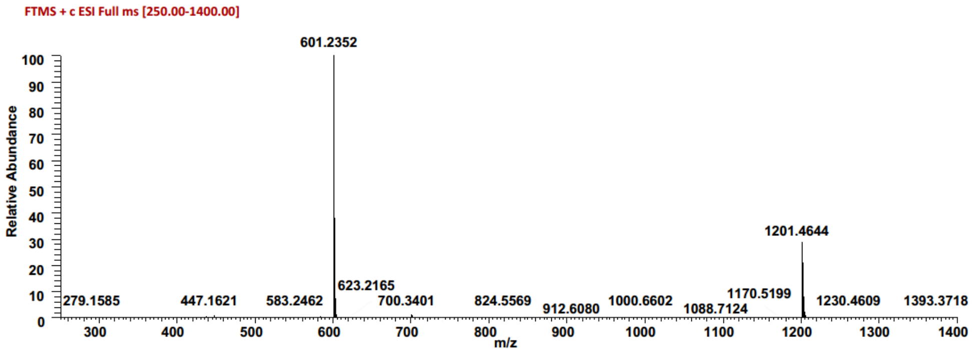
FTMS + c ESI Full ms [250.00-1400.00]
601.2352
1201.4644
623.2165
279.1585
447.1621
583.2462
700.3401
824.5569
912.6080
1000.6602
1088.7124
1170.5199
1230.4609
1393.3718
Relative Abundance
m/z

b

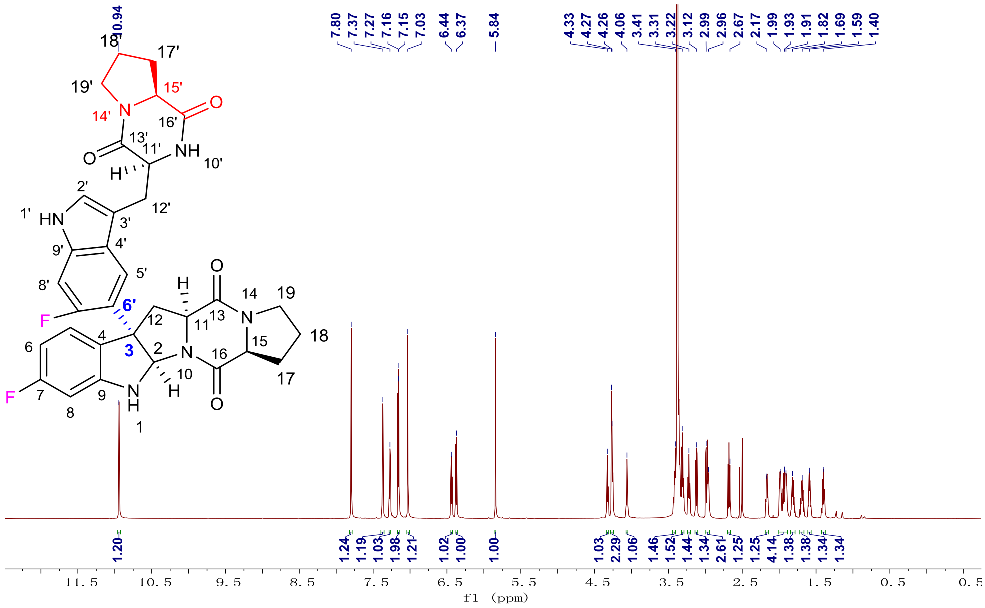
10.94
7.80
7.37
7.27
7.16
7.15
7.03
6.44
6.37
5.84
4.33
4.27
4.26
4.06
3.41
3.31
3.22
3.12
2.99
2.96
2.67
2.17
1.99
1.93
1.91
1.82
1.69
1.59
1.40
f1 (ppm)

c

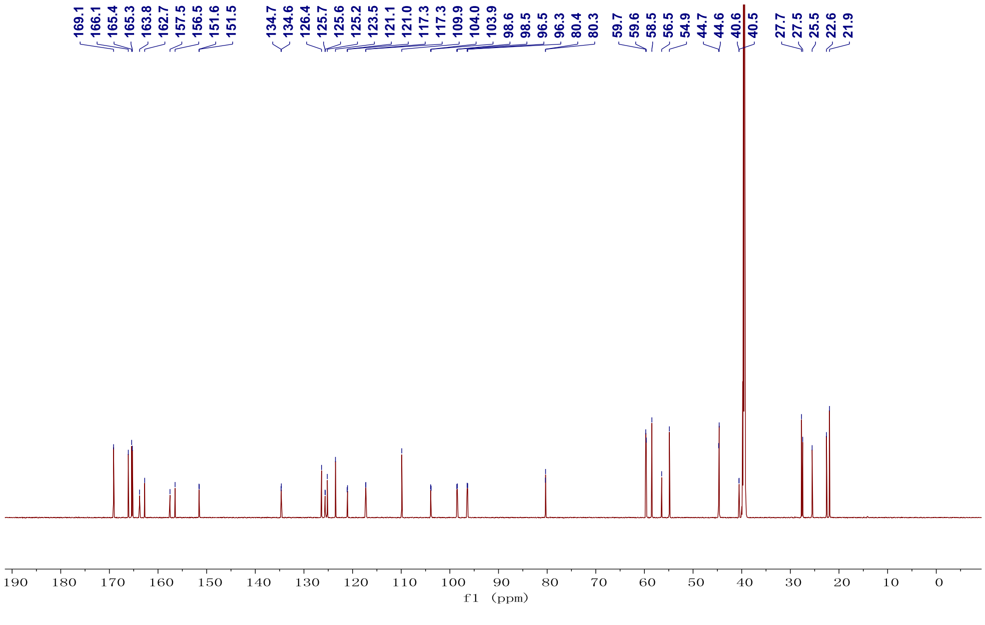
169.1
166.1
165.4
165.3
163.8
162.7
157.5
156.5
151.6
151.5
134.7
134.6
126.4
125.7
125.6
125.2
123.5
121.1
121.0
117.3
117.3
109.9
104.0
103.9
98.6
98.5
96.5
96.3
80.4
80.3
59.7
59.6
58.5
56.5
54.9
44.7
44.6
40.6
40.5
27.7
27.5
25.5
22.6
21.9
f1 (ppm)

d

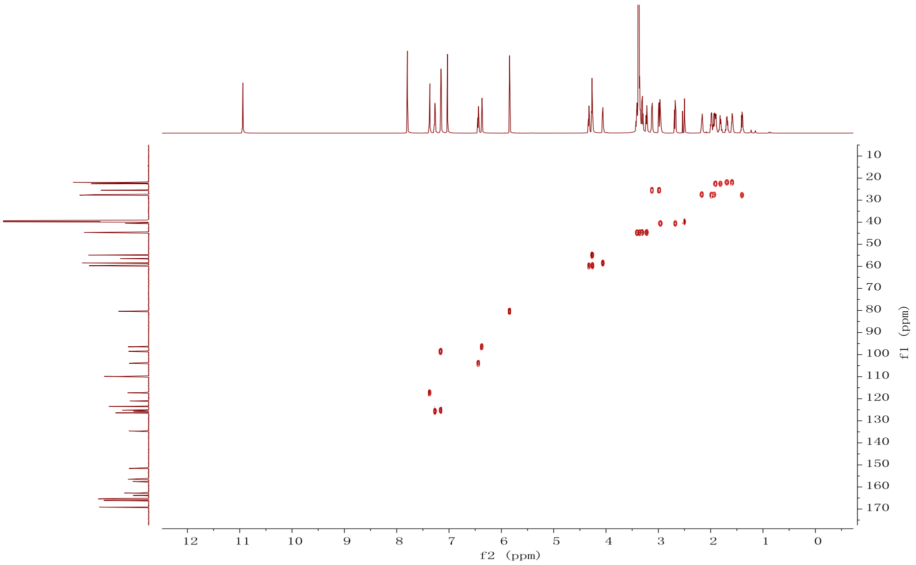

e

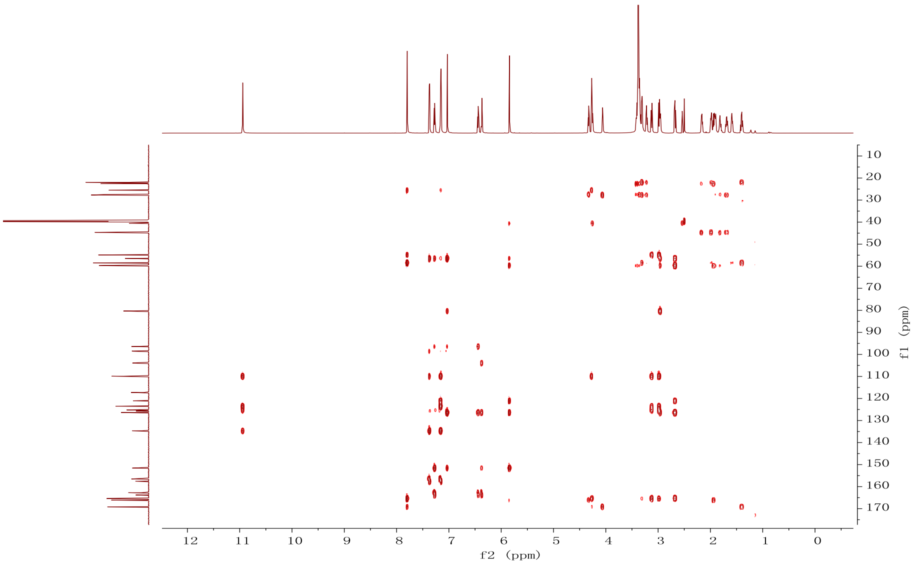

f

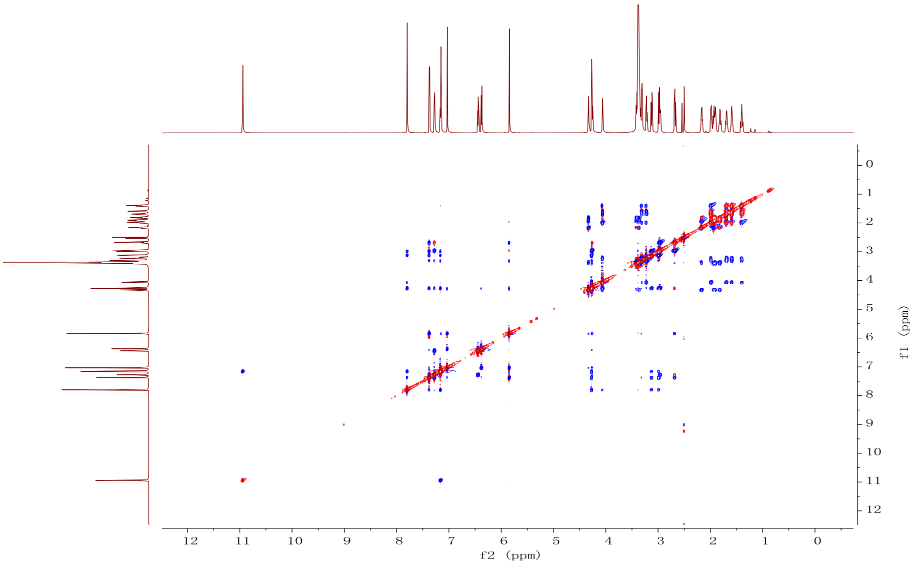

Supplementary Figure. 39.Spectra of compound NAS-27 (a) HRESIMS of compound of NAS-27(b) ^{1}H NMR of compound of NAS-27(c) ^{13}C NMR of compound of NAS-27(d) HSQC of compound of NAS-27 (e) HMBC of compound of NAS-27 (f) ROSEY of compound of NAS-27

a

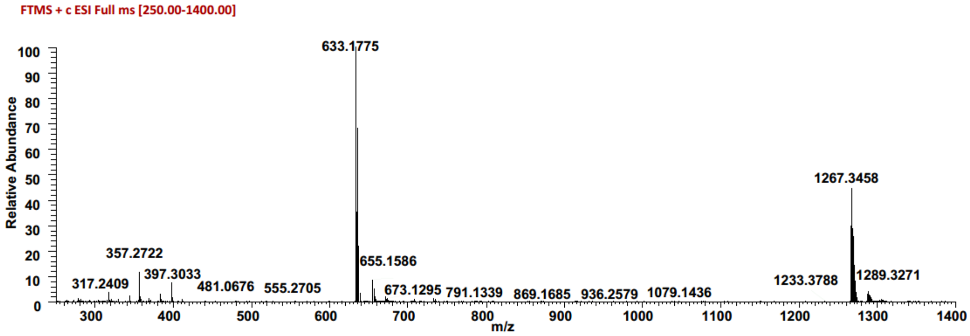
FTMS + c ESI Full ms [250.00-1400.00]
633.1775
1267.3458
357.2722
397.3033
317.2409
481.0676
555.2705
655.1586
673.1295
791.1339
869.1685
936.2579
1079.1436
1233.3788
1289.3271
Relative Abundance
m/z

b

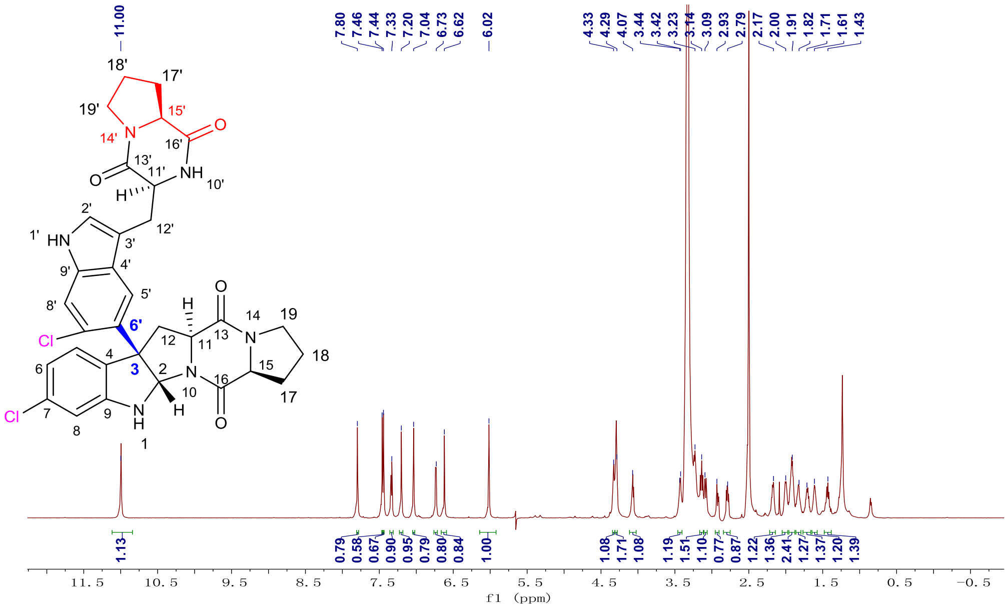
11.00
7.80 7.46 7.44 7.33 7.20 7.04 6.73 6.62
6.02
4.33 4.29 4.07 3.44 3.42 3.23 3.14 3.09 2.93 2.79 2.17 2.00 1.91 1.82 1.71 1.61 1.43
1.13
0.79 0.58 0.67 0.90 0.95 0.79 0.80 0.84
1.00
1.08 1.71 1.08 1.19 1.51 1.10 0.77 0.87 1.22 1.36 2.41 1.27 1.37 1.20 1.39
f1 (ppm)

c

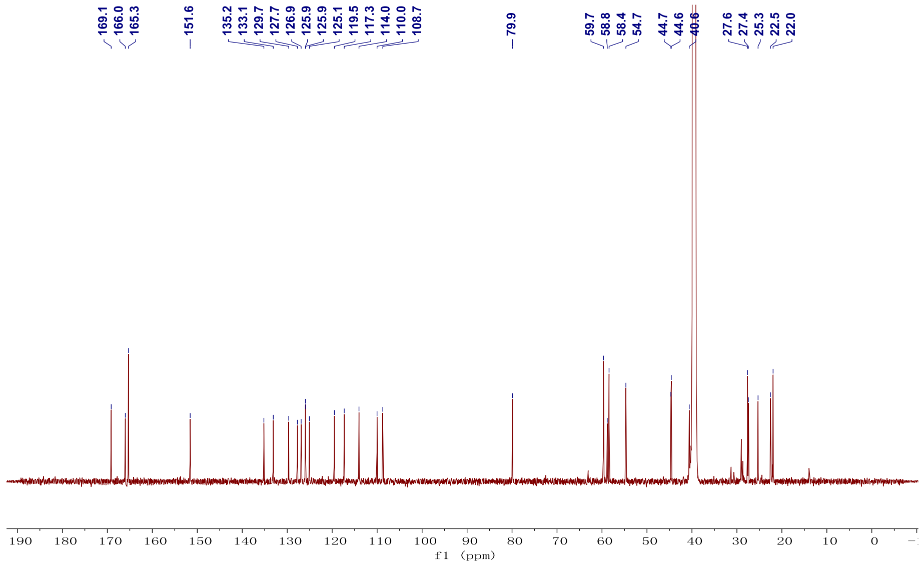
169.1 166.0 165.3
151.6
135.2 133.1 129.7 127.7 126.9 125.9 125.1 119.5 117.3 114.0 110.0 108.7
79.9
59.7 58.8 58.4 54.7
44.7 44.6 40.6
27.6 27.4 25.3 22.5 22.0
f1 (ppm)

d

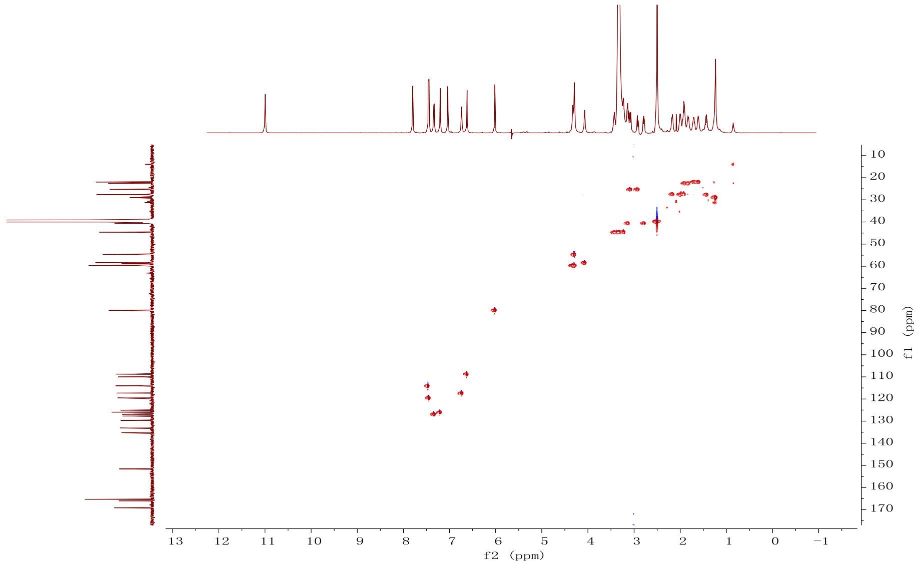

e

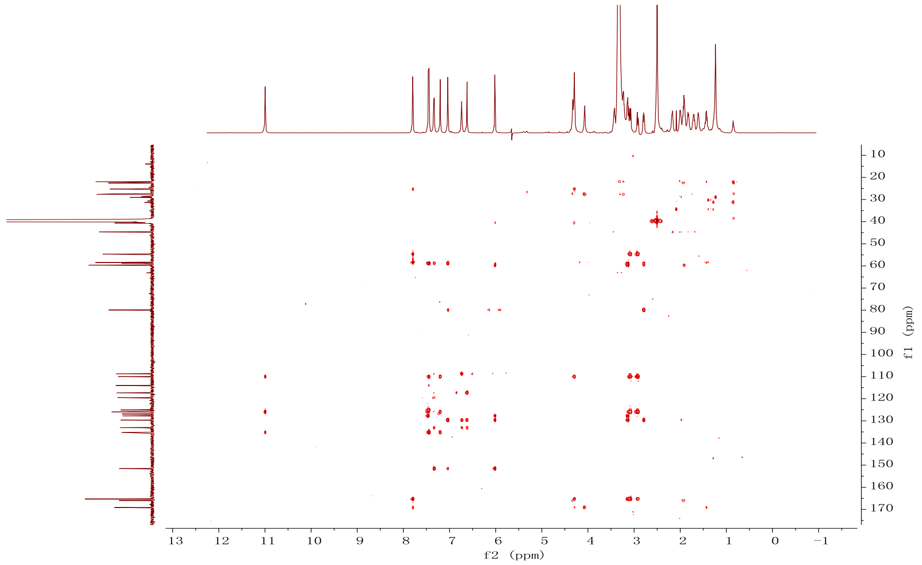

f

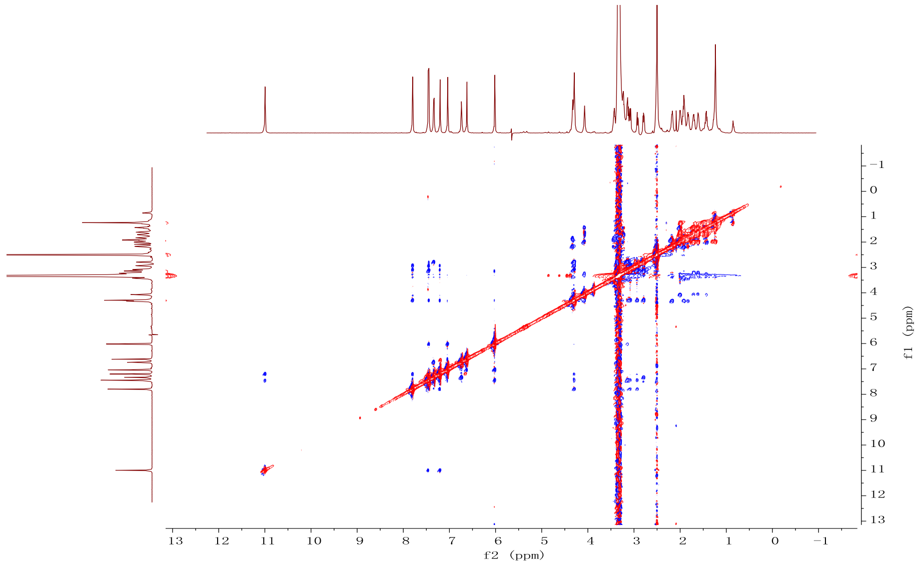

Supplementary Figure. 40.Spectra of compound NAS-28 (a) HRESIMS of compound of NAS-28(b) ^{1}H NMR of compound of NAS-28(c) ^{13}C NMR of compound of NAS-28(d) HSQC of compound of NAS-28 (e) HMBC of compound of NAS-28 (f) ROSEY of compound of NAS-28

a

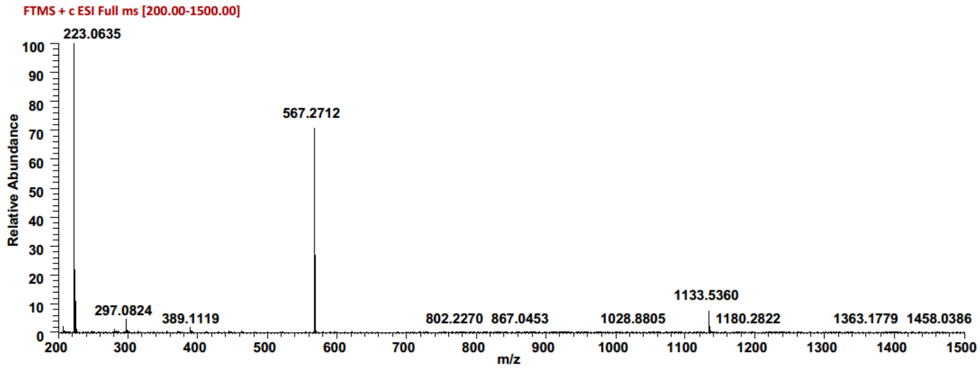
FTMS + c ESI Full ms [200.00-1500.00]
223.0635
567.2712
297.0824
389.1119
802.2270
867.0453
1028.8805
1133.5360
1180.2822
1363.1779
1458.0386
Relative Abundance
m/z

b

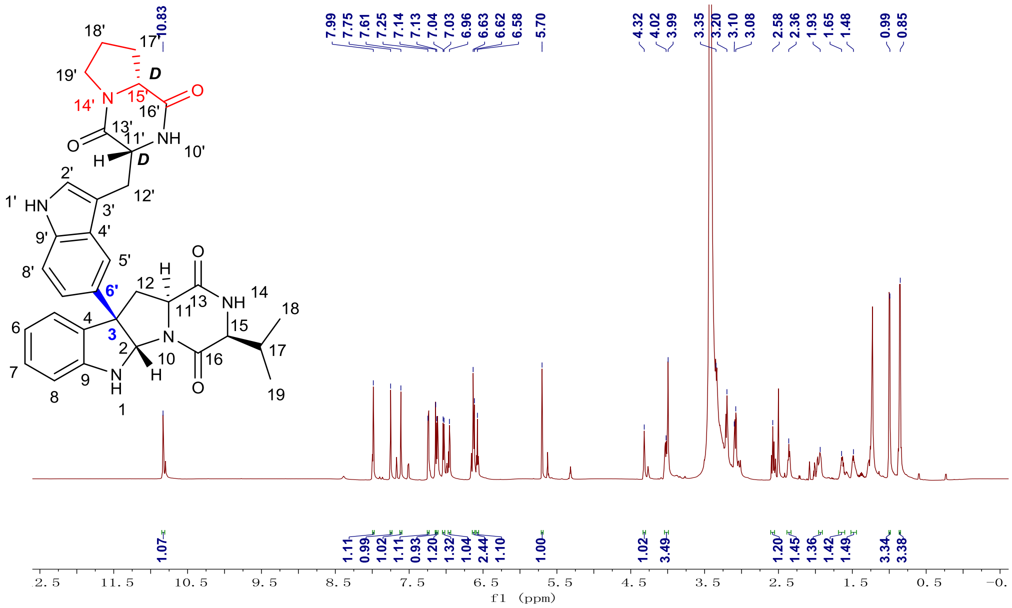
10.83
7.99 7.75 7.61 7.25 7.14 7.13 7.04 7.03 6.96 6.63 6.62 6.58
5.70
4.32 4.02 3.99 3.35 3.20 3.10 3.08 2.58 2.36 1.93 1.65 1.48 0.99 0.85
1.07 1.11 0.99 1.02 1.11 0.93 1.20 1.32 1.04 2.44 1.10 1.00 1.02 3.49 1.20 1.45 1.36 1.42 1.49 3.34 3.38
f1 (ppm)

c

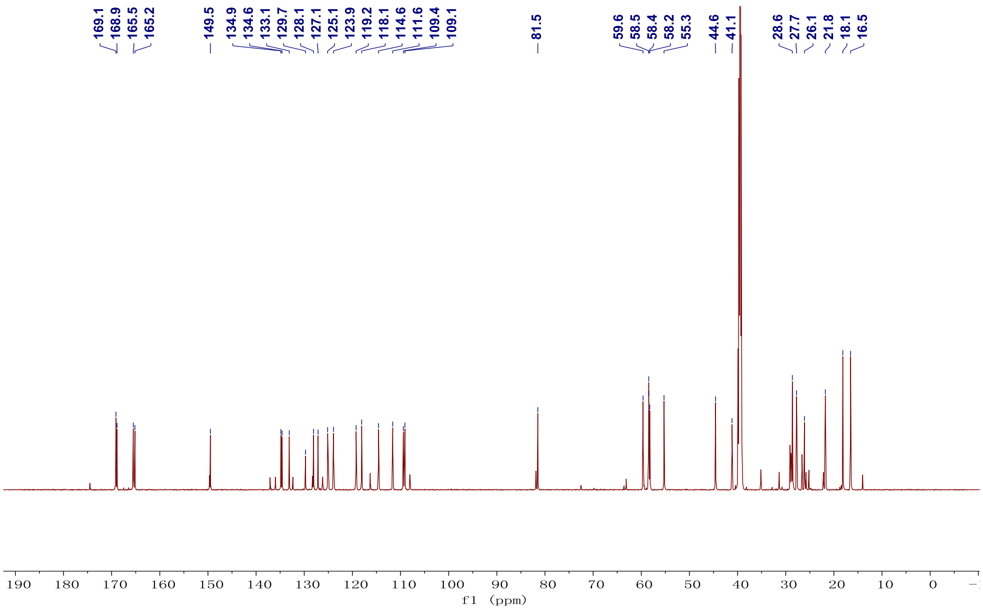
169.1 168.9 165.5 165.2
149.5
134.9 134.6 133.1 129.7 128.1 127.1 125.1 123.9 119.2 118.1 114.6 111.6 109.4 109.1
81.5
59.6 58.5 58.4 58.2 55.3 44.6 41.1
28.6 27.7 26.1 21.8 18.1 16.5
f1 (ppm)

d

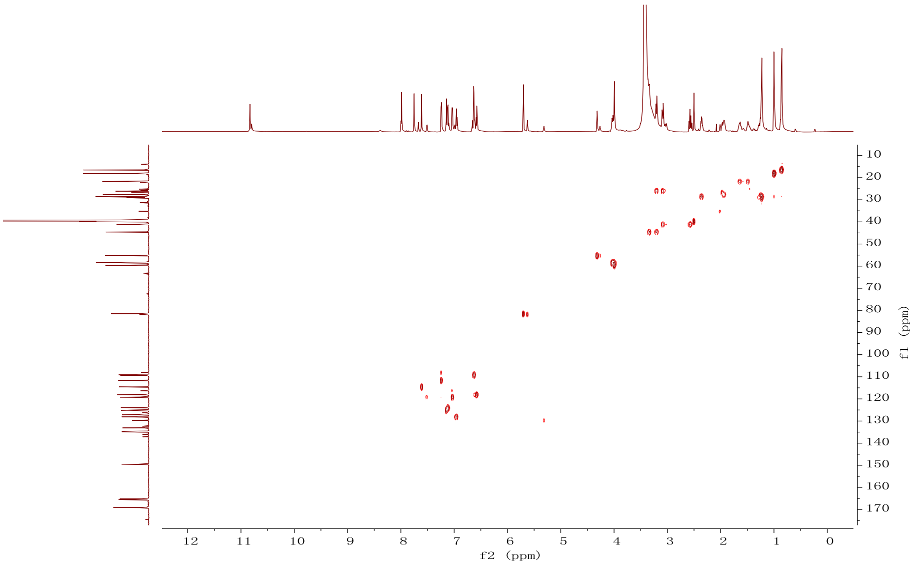

e

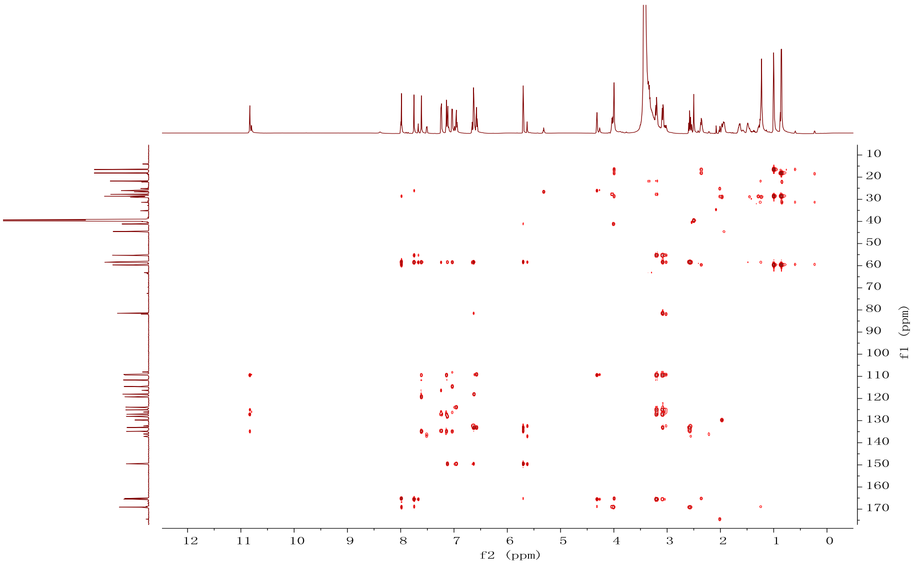

f

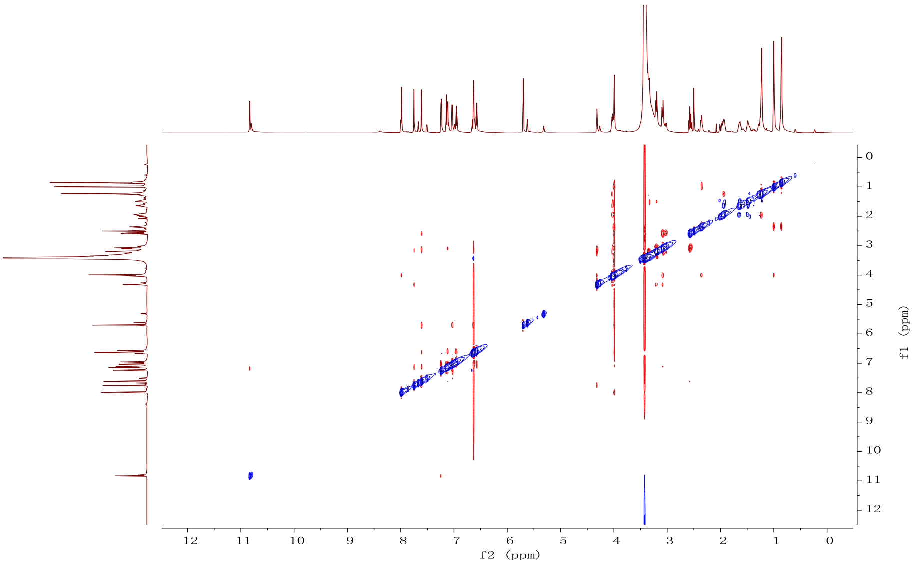

Supplementary Figure. 41.Spectra of compound NAS-29 (a) HRESIMS of compound of NAS-29(b) ^{1}H NMR of compound of NAS-29(c) ^{13}C NMR of compound of NAS-29(d) HSQC of compound of NAS-29 (e) HMBC of compound of NAS-29 (f) ROSEY of compound of NAS-29

a

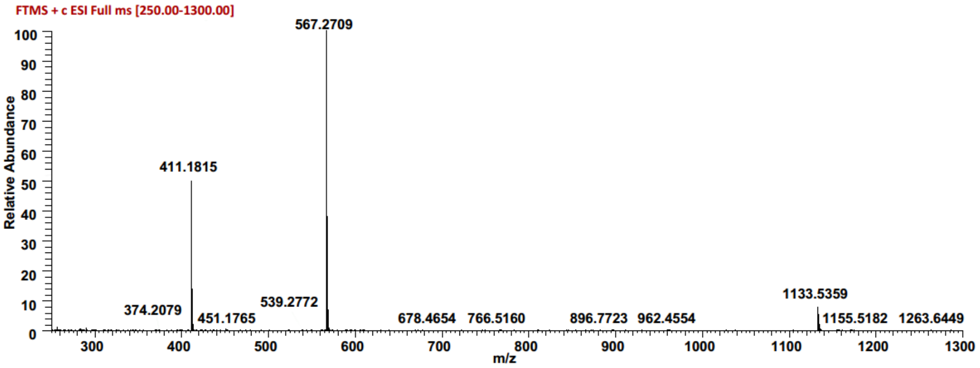

FTMS + c ESI Full ms [250.00-1300.00]
567.2709
411.1815
374.2079
451.1765
539.2772
678.4654
766.5160
896.7723
962.4554
1133.5359
1155.5182
1263.6449
Relative Abundance
m/z

b

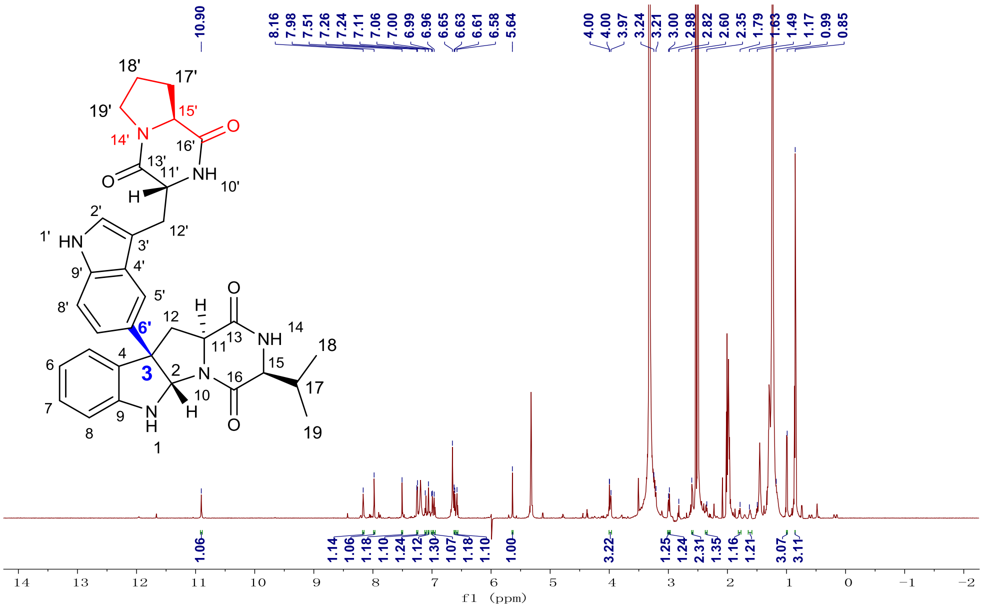

10.90
8.16 7.98 7.51 7.26 7.24 7.11 7.06 7.00 6.99 6.96 6.65 6.63 6.61 6.58
5.64
4.00 4.00 3.97 3.24 3.21 3.00 2.98 2.82 2.60 2.35 1.79 1.63 1.49 1.17 0.99 0.85
1.06 1.14 1.06 1.18 1.10 1.24 1.12 1.30 1.07 1.18 1.10 1.00 3.22 1.25 1.24 2.31 1.35 1.16 1.21 3.07 3.11
f1 (ppm)

c

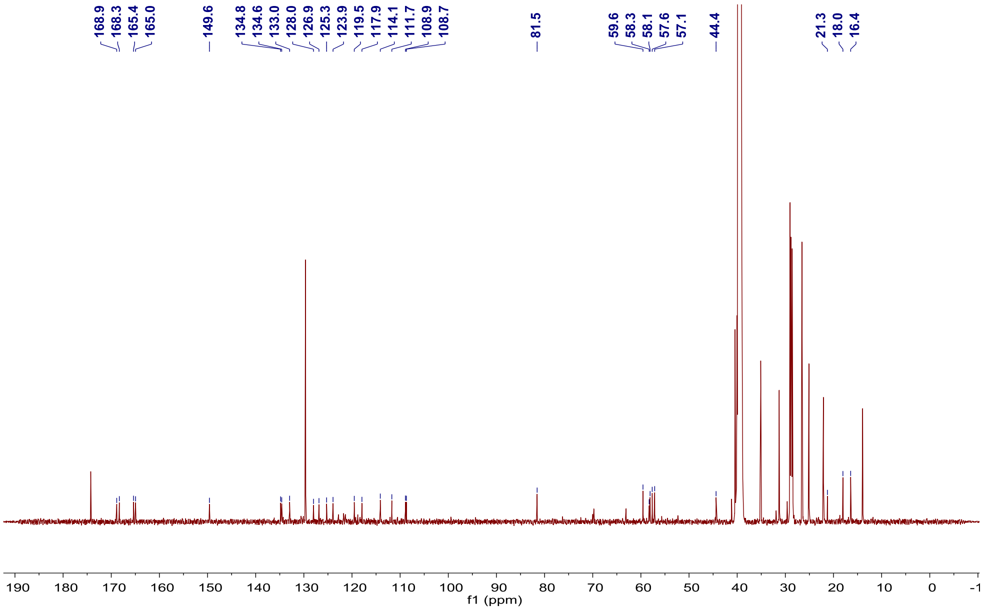

168.9 168.3 165.4 165.0
149.6
134.8 134.6 133.0 128.0 126.9 125.3 123.9 119.5 117.9 114.1 111.7 108.9 108.7
81.5
59.6 58.3 58.1 57.6 57.1
44.4
21.3 18.0 16.4
f1 (ppm)

d

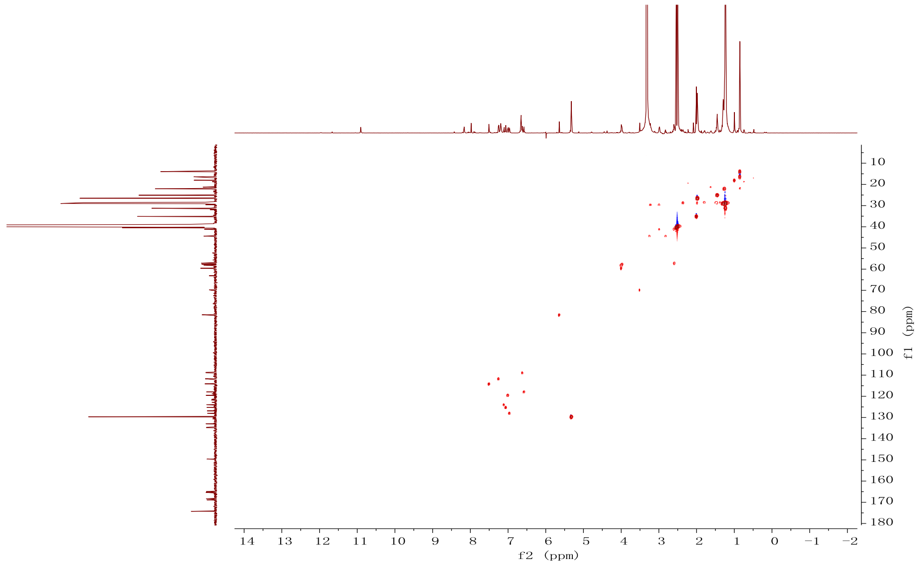

e

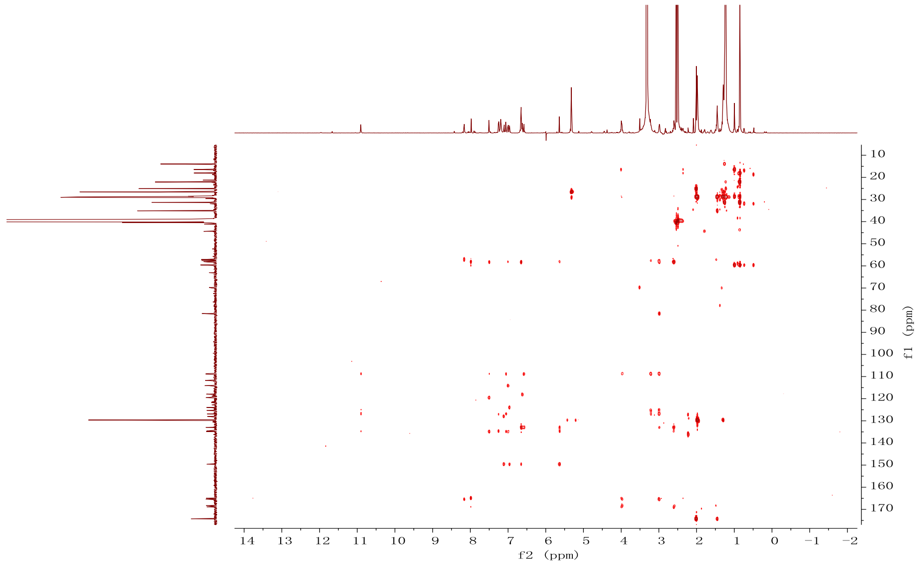

f

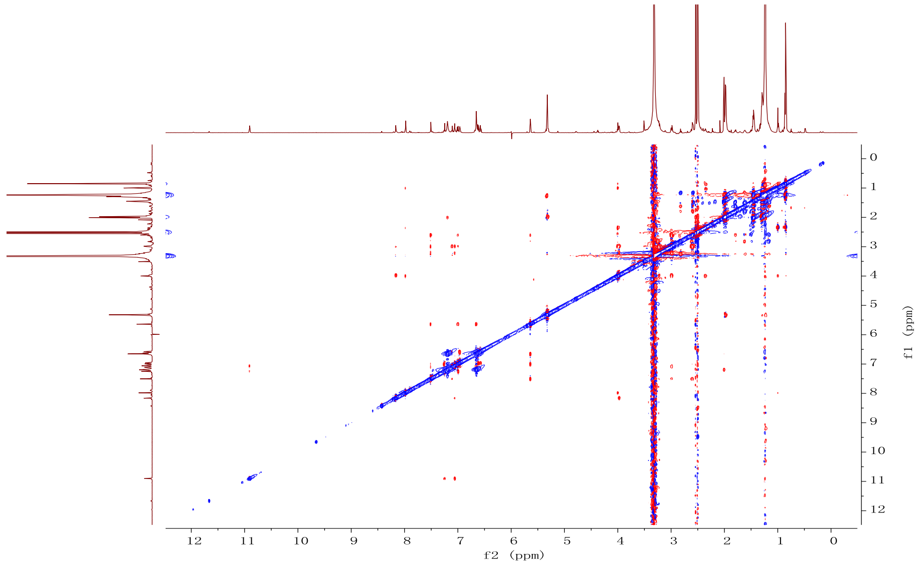

Supplementary Figure. 42.Spectra of compound NAS-30 (a) HRESIMS of compound of NAS-30(b) ^{1}H NMR of compound of NAS-30(c) ^{13}C NMR of compound of NAS-30(d) HSQC of compound of NAS-30 (e) HMBC of compound of NAS-30 (f) ROSEY of compound of NAS-30

Supplementary Tables

Supplementary Table 1 NascB mediated self-dimerization of cyclodipeptides

Code	Substrate	Reaction	Code	Substrate	Reaction
1	cW_L-P_L	YES	16	cW_L-K_L	NO
2	cW_L-A_L	YES	17	cW_L-C_L	NO
3	cW_L-V_L	YES	18	cW_L-H_L	NO
4	cW_L-G_L	NO	19	cW_L-R_L	NO
5	cW_L-I_L	NO	20	cW_L-W_L	NO
6	cW_L-L_L	NO	21	5Cl-cW_L-P_L	NO
7	cW_L-F_L	NO	22	6Cl-cW_L-P_L	NO
8	cW_L-M_L	NO	23	7Cl-cW_L-P_L	YES
9	cW_L-S_L	NO	24	8Cl-cW_L-P_L	NO
10	cW_L-T_L	NO	25	7F-cW_L-P_L	YES
11	cW_L-Y_L	NO	26	6Br-cW_L-P_L	NO
12	cW_L-N_L	NO	27	cW_D-P_D	NO
13	cW_L-Q_L	NO	28	cW_D-P_L	NO
14	cW_L-D_L	NO	29	cW_L-P_D	NO
15	cW_L-E_L	NO	30	Oxo-cW_L-P_L	NO

Supplementary Table 2 The reaction of cW_L-A_L with other cyclodipeptides

Code	Substrate	cW_L-A_L	Code	Substrate	cW_L-A_L
1	cW_L-P_L	YES	16	cW_L-K_L	NO
2	cW_L-A_L	YES	17	cW_L-C_L	NO
3	cW_L-V_L	YES	18	cW_L-H_L	NO
4	cW_L-G_L	NO	19	cW_L-R_L	NO
5	cW_L-I_L	YES	20	cW_L-W_L	NO
6	cW_L-L_L	YES	21	5Cl-cW_L-P_L	NO
7	cW_L-F_L	YES	22	6Cl-cW_L-P_L	NO
8	cW_L-M_L	YES	23	7Cl-cW_L-P_L	NO
9	cW_L-S_L	NO	24	8Cl-cW_L-P_L	NO
10	cW_L-T_L	NO	25	7F-cW_L-P_L	NO
11	cW_L-Y_L	NO	26	6Br-cW_L-P_L	NO
12	cW_L-N_L	NO	27	cW_D-P_D	NO
13	cW_L-Q_L	NO	28	cW_D-P_L	NO
14	cW_L-D_L	NO	29	cW_L-P_D	NO
15	cW_L-E_L	NO			

Supplementary Table 3 The reaction of cW_L-P_L with other cyclodipeptides

**Code**	**Substrate**	**cW_L-P_L**	**Code**	**Substrate**	**cW_L-P_L**
1	cW_L-P_L	YES	16	cW_L-K_L	NO
2	cW_L-A_L	YES	17	cW_L-C_L	NO
3	cW_L-V_L	YES	18	cW_L-H_L	NO
4	cW_L-G_L	NO	19	cW_L-R_L	NO
5	cW_L-I_L	YES	20	cW_L-W_L	NO
6	cW_L-L_L	YES	21	5Cl-cW_L-P_L	NO
7	cW_L-F_L	YES	22	6Cl-cW_L-P_L	NO
8	cW_L-M_L	YES	23	7Cl-cW_L-P_L	NO
9	cW_L-S_L	NO	24	8Cl-cW_L-P_L	NO
10	cW_L-T_L	NO	25	7F-cW_L-P_L	NO
11	cW_L-Y_L	NO	26	6Br-cW_L-P_L	NO
12	cW_L-N_L	NO	27	cW_D-P_D	NO
13	cW_L-Q_L	NO	28	cW_D-P_L	NO
14	cW_L-D_L	NO	29	cW_L-P_D	NO
15	cW_L-E_L	NO			

Supplementary Table 4 The reaction of cW_L-V_L with other cyclodipeptides

**Code**	**Substrate**	**cW_L-V_L**	**Code**	**Substrate**	**cW_L-V_L**
1	cW_L-P_L	YES	16	cW_L-K_L	NO
2	cW_L-A_L	YES	17	cW_L-C_L	NO
3	cW_L-V_L	YES	18	cW_L-H_L	NO
4	cW_L-G_L	NO	19	cW_L-R_L	NO
5	cW_L-I_L	YES	20	cW_L-W_L	NO
6	cW_L-L_L	YES	21	5Cl-cW_L-P_L	NO
7	cW_L-F_L	YES	22	6Cl-cW_L-P_L	NO
8	cW_L-M_L	YES	23	7Cl-cW_L-P_L	NO
9	cW_L-S_L	YES	24	8Cl-cW_L-P_L	NO
10	cW_L-T_L	YES	25	7F-cW_L-P_L	NO
11	cW_L-Y_L	YES	26	6Br-cW_L-P_L	NO
12	cW_L-N_L	NO	27	cW_D-P_D	YES
13	cW_L-Q_L	NO	28	cW_D-P_L	YES
14	cW_L-D_L	NO	29	cW_L-P_D	NO
15	cW_L-E_L	NO			

Supplementary Table 5 The protective effect of compounds NAS-1-28 against Aβ25-35 (A4559)-induced PC-12 cells damage (Anti-Alzheimer model)

Name	Concentration (μM)	Inhibition Rate（%）
NAS-1	10	51.05 ± 2.73
NAS-2	10	48.69 ± 2.84
NAS-3	10	46.30 ± 2.96
NAS-4	10	47.49 ± 2.90
NAS-5	10	52.27 ± 2.67
NAS-6	10	47.49 ± 2.90
NAS-7	10	53.46 ± 2.62
NAS-8	10	52.27 ± 3.22
NAS-9	10	47.49 ± 2.90
NAS-10	10	49.88 ± 2.78
NAS-11	10	46.30 ± 2.96
NAS-12	10	47.49 ± 2.90
NAS-13	10	52.27 ± 2.67
NAS-14	10	48.68 ± 2.84
NAS-15	10	49.50 ± 0.00
NAS-16	10	45.11 ± 3.06
NAS-17	10	48.69 ± 3.36
NAS-18	10	47.49 ± 2.90
NAS-19	10	47.49 ± 2.90
NAS-20	10	46.30 ± 2.96
NAS-21	10	50.07 ± 3.26
NAS-22	10	51.07 ± 2.73
NAS-23	10	51.06 ± 0.00
NAS-24	10	50.06 ± 3.24
NAS-25	10	51.04 ± 2.73
NAS-26	10	48.69 ± 3.36
NAS-27	10	47.49 ± 2.90
NAS-28	10	50.07 ± 3.26
Blank	-	-
Negative Control	-	49.88 ± 2.78

Supplementary Table 6 The protective effects of compounds NAS-1-28 against glutamate-induced PC-12 cells apoptosis (Neuron protection model).

Name	Concentration (μM)	Inhibition Rate（%）
NAS-1	10	36.57 ± 2.24
NAS-2	10	43.70 ± 2.26
NAS-3	10	37.21 ± 1.47
NAS-4	10	41.51 ± 4.86
NAS-5	10	42.81 ± 2.23
NAS-6	10	44.56 ± 2.66
NAS-7	10	35.11 ± 2.24
NAS-8	10	42.61 ± 2.94
NAS-9	10	38.57 ± 2.76
NAS-10	10	32.73 ± 2.83
NAS-11	10	33.00 ± 4.92
NAS-12	10	29.01 ± 1.67
NAS-13	10	37.54 ± 2.24
NAS-14	10	42.11 ± 2.27
NAS-15	10	40.61 ± 2.90
NAS-16	10	34.54 ± 2.79
NAS-17	10	44.00 ± 4.86
NAS-18	10	41.92 ± 1.20
NAS-19	10	41.47 ± 4.86
NAS-20	10	42.89 ± 4.86
NAS-21	10	41.25 ± 2.27
NAS-22	10	41.21 ± 2.27
NAS-23	10	49.43 ± 0.89
NAS-24	10	40.61 ± 1.55
NAS-25	10	35.93 ± 2.29
NAS-26	10	48.62 ± 2.24
NAS-27	10	29.88 ± 2.87
NAS-28	10	47.98 ± 1.65
Nimodipine	20	33.30 ± 2.79
Blank	-	-
Negative Control	-	42.04 ± 4.86

Supplementary Table 7 Bacterial strains and plasmids used in this study

Strains	Description	Source
E. coli		
DH5α	Host for general cloning	Invitrogen
BL21(DE3)	Host for protein expression	Invitrogen
Rossette (DE3)	Host for protein expression	Invitrogen
JM109 (DE3)	Host for protein expression	Promega
C41 (DE3)	Host for protein expression	Sigma
C43 (DE3)	Host for protein expression	Sigma
BL21 (DE3) pLysE	Host for protein expression	Thermo
ET12567/pUZ8002	Donor strain for conjugation	ATCC
GB05-dir	Host for PCR-targeting	[3]
GB05-dir-T7	Host for protein expression	This study
Streptomyces		
sp. CMB-MQ030	NAZ-A, NAZ-B producer	Prof. Capon
albus J1074	Host for heterologous expression	Lab storage
lividans TK24	Host for heterologous expression	Lab storage
coelicolor M1154	Host for heterologous expression	Lab storage
mWHU2475	*S. albus* J1074 contains pWHU2482	This study
mWHU2476	*S. lividans* TK24 contains pWHU2482	This study
mWHU2477	*S. coelicolor* M1154 contains pWHU2482	This study
mWHU2478	*S. albus* J1074 contains pWHU2483	This study
mWHU2479	*S. lividans* TK24 contains pWHU2483	This study
mWHU2480	*S. coelicolor* M1154 contains pWHU2483	This study
mWHU2481	*S. albus* J1074 contains pIB139	This study
mWHU2482	*S. lividans* TK24 contains pIB139	This study
mWHU2483	*S. coelicolor* M1154 contains pIB139	This study
**Plasmids**		
pSP72	*E. coli* cloning vector	Promega
pET28a	Protein expression vector in *E. coli*	Novagen
pET21a	Protein expression vector in *E. coli*	Novagen
pSJ5	Protein expression vector in *E. coli*	Lab storage
pSJ8	Protein expression vector in *E. coli*	Lab storage
pRSF-Duet	Protein expression vector in *E. coli* with	Novagen

	two MCS	
pET-Duet	Protein expression vector in *E. coli* with two MCS	Novagen
pIB139	Plasmid for heterologous expression in Streptomyces	[4]
pTRX-Fd	pSJ5 derivative for Spinach ferrodoxin expression in *E. coli*	This study
pMBP-FdR	pSJ8 derivative for Spinach ferrodoxin reductase expression in *E. coli*	This study
pETDuet-saFdR1	pET-Duet derivative, contains *S. albus* ferrodoxin reductase 1	This study
pETDuet-saFdR2	pET-Duet derivative, contains *S. albus* ferrodoxin reductase 2	This study
pETDuet-saFdR3	pET-Duet derivative, contains *S. albus* ferrodoxin reductase 3	This study
pETDuet-saFdR4	pET-Duet derivative, contains *S. albus* ferrodoxin reductase 4	This study
pETDuet-saFdR1-Fd1	pET-Duet derivative, for *S. albus* ferrodoxin reductase 1 and ferrodoxin 1 co-expression in *E. coli*	This study
pETDuet-saFdR1-Fd2	pET-Duet derivative, for *S. albus* ferrodoxin reductase 1 and ferrodoxin 2 co-expression in *E. coli*	This study
pETDuet-saFdR1-Fd3	pET-Duet derivative, for *S. albus* ferrodoxin reductase 1 and ferrodoxin 3 co-expression in *E. coli*	This study
pETDuet-saFdR2-Fd1	pET-Duet derivative, for *S. albus* ferrodoxin reductase 2 and ferrodoxin 1 co-expression in *E. coli*	This study
pETDuet-saFdR2-Fd2	pET-Duet derivative, for *S. albus* ferrodoxin reductase 2 and ferrodoxin 2 co-expression in *E. coli*	This study
pETDuet-saFdR2-Fd3	pET-Duet derivative, for *S. albus* ferrodoxin reductase 2 and ferrodoxin 3 co-expression in *E. coli*	This study
pETDuet-saFdR3-Fd1	pET-Duet derivative, for *S. albus*	This study

	ferrodoxin reductase 3 and ferrodoxin 1 co-expression in *E. coli*	
pETDuet-saFdR3-Fd2	pET-Duet derivative, for *S. albus* ferrodoxin reductase 3 and ferrodoxin 2 co-expression in *E. coli*	This study
pETDuet-saFdR3-Fd3	pET-Duet derivative, for *S. albus* ferrodoxin reductase 2 and ferrodoxin 3 co-expression in *E. coli*	This study
pETDuet-saFdR4-Fd1	pET-Duet derivative, for *S. albus* ferrodoxin reductase 4 and ferrodoxin 1 co-expression in *E. coli*	This study
pETDuet-saFdR4-Fd2	pET-Duet derivative, for *S. albus* ferrodoxin reductase 4 and ferrodoxin 2 co-expression in *E. coli*	This study
pETDuet-saFdR4-Fd3	pET-Duet derivative, for *S. albus* ferrodoxin reductase 4 and ferrodoxin 3 co-expression in *E. coli*	This study
pWHU2482	pIB139 derived，containing *cdps-p450* gene of cluster 1 (*nasc*)	This study
pWHU2483	pIB139 derived，containing *cdps-p450* gene of cluster 2	This study
pWHU2484	pET28a derivative for NascB expression in *E. coli*	This study
pWHU2485	pET28a derivative for cluster2-P450 expression in *E. coli*	This study
pWHU2486	pET21a derivative, containing NascB gene	This study
pWHU2487	pRSF-Duet derivative for Trx-Fd and MBP-FdR *co*-expression in *E. coli*	This study
pWHU2488	pRSF-Duet derivative, containing NascB gene	This study
pWHU2489	pET-Duet derived, containing NascB and GDH in two MCS	This study

Supplementary Table 8 Primers used for amplifying Fd and FdR genes from *S. albus* J1074, and the restriction sites are underlined

Name	Sequence
saFdR1-F	5'-GGGAATTCCATATGGTCGACGCGGATCAGACATTCG-3'
saFdR1-R	5'-CCGCTCGAGTCAGGCCCCGAGGCTCTCCAG-3'
saFdR2-F	5'-GGGAATTCCATATGGCCGCAACCCCGCAGAATCC-3'
saFdR1-R	5'-CCGCTCGAGTCACGACAGGGCCTCCTCGTCGG-3'
saFdR3-F	5'-GGAATTCCATATGACCTACGCCATCACCCAGACCTGC-3'
saFdR3-R	5'-CCGCTCGAGTCAGCCCGGCAGCCGCAGCC-3'
saFdR4-F	5'-GGAATTCCATATGGTGGTCGTCGGCGCG-3'
saFdR4-R	5'-CCGCTCGAGTCAGGCCGTGGTCGACTT-3'
saFd1-F	5'-CGCGGATCCATGCAGGAGGAGGCCGGGCCC-3'
saFd1-R	5'-CCCAAGCTTCTACGACGCCTCGGGTCCGTAGAC-3'
saFd2-F	5'-CGCGGATCCATGACCTACGTCATCGCGCAGCC-3'
saFd2-R	5'-CCCAAGCTTCTACTGGTTCTGCGGCGGCAG-3'
saFd3-F	5'-GGAGGATCCATGAGGATCACTGTCGATCAGG-3'
saFd3-R	5'-GTGAAAGCTTCGGCCGAAGCTTTCACTCCTCCTGTGCCA-3'

Supplementary Table 9 NMR data of compound NAS-1 in DMSO-d_6

no.	δ_H,mult (*J* in Hz)	δ_C	^{1}H-^{13}C HMBC	ROESY
2	5.66,s	81.4	4,9	12b,5’
3		58.4		
4		133.1		
5	7.13,dd,(7.5,1.2)	123.8	3,7,9	6,7
6	6.61,td,(7.4,1.1)	118.1	4,8	5,7
7	6.96,td,(7.6,1.3)	128.0	5,9	5,8
8	6.63,d,(7.8)	109.3	4,6	7
9		149.4		
11	4.05,m	58.9	12,13	12a
12a	3.03,m	40.4	2,3,4,13	11,12b
12b	2.65,m		3,4	5’,12a
13		169.0		
15	4.15,m	50.2	16,17	14-NH,17
16		166.5		
17	1.26,d,(6.9)	15.8	15,16	15
2’	7.05,d,(2.4)	124.9	3’.4’.9’	12’a/b
3’		108.9		
4’		127.5		
5’	7.64,s	114.9	3,3’,7’,9’	2,12a/b
6’		134.3		
7’	6.99,dd,(8.6,1.8)	119.2	3,5’,9’	2,12b
8’	7.20,d,(8.5)	111.3	4’,6’	1’-NH,7’
9’		134.7		
11’	4.11,m	55.3	3’,12’,13’	14’-NH,12’a/b
12’a	3.23,dd,(14.4,4.6)	28.7	2’,3’,4’,11’,13’	11’,12’b
12’b	3.05,m		2’,3’,4’,11’,13’	11’,12a’
13’		167.0		
15’	3.65,m	49.8	16’,17’	10’-NH,17’
16’		167.9		
17’	0.54,d,(7.0)	19.6		15’
1-NH	6.59,s		3,4,9	2
14-NH	8.18,s		11,13,15,16,17	15,17
1’-NH	10.84,d,(2.4)		2’,3’,4’,9’	2’,8’
10’-NH	7.93,s		11’,15’,16’	15’
14’-NH	7.97,s		11’,13’,15’	11’

Supplementary Table 10 NMR data of compound NAS-2 in DMSO-d_6

no.	δ_H	δ_C	**^{1}H-^{13}C HMBC**	**ROESY**
2	5.61,s	81.8	3,4,9,7’	1-NH,12b,8’
3		58.4		
4		132.2		
5	7.14,d,(7.4)	124.0	7,9	6,7
6	6.62,m	117.9	8	5,7
7	6.98,dd,(7.6,1.2)	128.2	9	8
8	6.63,m	109.1	4,6	7
9		149.7		
11	4.02,m	58.3	13	2,12a
12a	3.01,m	41.2	2,3,4	12b
12b	2.55,m		3,4,13,7’	8’,12a
13		169.0		
15	4.00,s	59.5	16,18,19	14-NH,17,18,19
16		165.2		
17	2.36,m	28.4	15,16,18,19	15,18,19
18	1.01,d,(7.3)	18.1	15,17,19	15,17,19
19	0.88,d,(6.9)	16.5	15,17,18	15,17,18
2’	7.02,s	124.8	3,3’,4’,9’	1’-NH,12’b
3’		108.5		
4’		126.5		
5’	7.50,d,(8.4)	119.2	3’,9’	6’
6’	7.00,dd,(7.5,1.5)	116.4	8’	2,12b,5’
7’		136.8		
8’	7.23,d,(1.5)	107.9	3,4’,6’	1’-NH,2,12a/b
9’		135.8		
11’	4.07,m	55.3	3’,12’,13’	14’-NH,12’a/b
12’a	3.17,dd,(14.5,4.6)	28.8	2’,3’,4’,11’	11’,12’b
12’b	2.99,dd,(14.5,4.5)		2’,3’,4’,11’,13’	11’,12’a
13’		166.8		
15’	3.61,m	49.8	16’,17’	10’-NH,17’
16’		167.9		
17’	0.52,d,(7.0)	19.5	15’,16’	15’
1-NH	6.66,s		3,4,9	2
14-NH	8.00,s		11,13,16	15,17
1’-NH	10.81,d,(2.4)		2’,3’,4’,9’	2’,8’
10’-NH	7.89,s		11’,16’	15’
14’-NH	7.94,s		13’,15’	11’

Supplementary Table 11 NMR data of compound NAS-3 in DMSO-d_6

no.	δ_H	δ_C	**^{1}H-^{13}C HMBC**	**ROESY**
2	5.60,s	81.9	3,4,9,12,7’	1-NH,12b,6’,8’
3		58.4		
4		132.2		
5	7.13,d,(7.4)	124.0	3,7,9	7,8
6	6.61,dd,(7.4,1.1)	117.9	5,7,8	5,7
7	6.98,dd,(7.6,1.3)	128.2	5,9	8
8	6.63,m	109.1	4,6	5,7
9		149.7		
11	4.00,m	58.3	12,13	12a
12a	3.01,m	41.3	2,3,4	11,12b
12b	2.54,m		3,4,13,7’	8’,12a
13		168.8		
15	4.04,t,(2.2)	59.0	16,17,18	14-NH,17,18a
16		165.2		
17	2.04,m	35.5	16,18	15,20
18a	1.40,m	24.1	15,17,19,20	17,19,20
18b	1.30,m		15,17,19,20	17,19,20
19	0.83,t,(7.4)	12.3	17,18	17,18a/b
20	0.98,d,(7.2)	14.9	15,17,18	15,17,18a/b
2’	7.02,dd,(5.6,2.0)	124.8	3’,4’,9’	1’-NH
3’		108.5		
4’		126.5		
5’	7.50,d,(8.4)	119.2	3’,4’,7’,9’	6’
6’	7.00,dd,(5.3,1.5)	116.4	3,8’	2,5’
7’		136.8		
8’	7.23,d,(1.4)	107.9	3,4’,6’	1’-NH,2
9’		135.8		
11’	4.07,m	55.3	3’,12’,13’	14’-NH,12’a/b
12’a	3.17,dd,(14.5,4.6)	28.8	2’,3’,4’,11’,13’	11’,12’b
12’b	2.98,m		2’,3’,4’,11’,13’	11’,12a’
13’		166.8		
15’	3.61,m	49.8	16’,17’	10’-NH,17’
16’		167.9		
17’	0.52,d,(6.9)	19.5	15’	15’
1-NH	6.65,s		2,3,4,9	2
14-NH	7.99,s		13,16,17	15,17
1’-NH	10.81,d,(2.4)		2’,3’,4’,9’	2’,8’
10’-NH	7.89,d,(2.2)		11’,15’,16’	15’
14’-NH	7.94,d,(2.1)		13’,15’	11’

Supplementary Table 12 NMR data of compound NAS-4 in DMSO-d_6

no.	δ_H	δ_C	^1H-^{13}C HMBC	ROESY
2	5.66,s	81.4	4,9,12,6’	12b,5’,7’
3		58.3		
4		133.2		
5	7.09,dd,(7.5,1.2)	123.8	3,7,9	6,7
6	6.59,td,(7.4,1.0)	118.0	4,5,7,8,9	5,7
7	6.95,td,(7.6,1.2)	127.9	5,9	6
8	6.63,m	109.0	4,6,9	7
9		149.5		
11	3.99,m	58.2	12,13	12a/b
12a	3.01,dd,(12.4,5.6)	40.8	2,3,4,11	11,12b,5’
12b	2.62,t,(12.1)		3,4,11,13,6’	11,12a,5’
13		168.9		
15	4.04,t,(2.2)	59.1	16,17,18	14-NH,17,18a,20
16		165.1		
17	2.04,m	35.6	15,16,18,19,20	20
18a	1.39,m	24.1	15,17,19,20	17,18a,19,20
18b	1.30,m		15,17,19,20	17,19,20
19	0.83,t,(7.4)	12.3	17,18	17,18a/b
20	0.97,d,(7.1)	14.9	15,17,18	15,17,18a/b
2’	7.05,d,(2.3)	124.9	3’,4’,9’,12’	1’-NH,12’a/b
3’		109.0		
4’		127.5		
5’	7.62,s	114.7	3,3’,7’,9’	2,12b
6’		134.5		
7’	6.98,dd,(8.6,1.8)	119.1	3,5’,9’	2,8’
8’	7.21,d,(8.5)	111.4	4’,6’	1’-NH,7’
9’		134.7		
11’	4.10,m	55.3	3’,12’,13’	10’-NH,12’a/b
12’a	3.22,dd,(14.4,4.6)	28.7	2’,3’,4’,11’,13’	11’,12’b
12’b	3.04,dd,(14.4,4.7)		2’,3’,4’,11’,13’	10’-NH,11’,12a’
13’		167.0		
15’	3.66,m	49.8	16’,17’	14’-NH,17’
16’		167.8		
17’	0.58,d,(6.9)	19.6	15’	15’
1-NH	6.62,s		4	2
14-NH	7.98,s		13,16,17	15,17
1’-NH	10.84,d,(2.4)		2’,3’,4’,9’	2’,8’
10’-NH	7.95,d,(2.2)		11’,13’,15’	11’,12’b
14’-NH	7.93,d,(2.2)		11’,15’,16’	15’,17’

Supplementary Table 13 NMR data of compound NAS-5 in DMSO-d_6

no.	δ_H	δ_C	**^{1}H-^{13}C HMBC**	**ROESY**
2	5.63,d,(2.7)	84.9	9	1-NH,6’,8’
3		59.4		
4		134.4		
5	6.84,dd,(7.4,1.2)	123.7	3,7,9	6
6	6.59,m	117.9	4,5,7,8	5,7
7	6.99,m	127.9	5,9	5,8
8	6.61,m	109.3	6	7
9		148.1		
11	4.64,t,(8.6)	58.3	12,13	2,12a
12a	3.06,dd,(13.6,7.9)	38.6	2,3,13	11,12b
12b	2.47,m		2,4,11,13	12a
13		170.3		
15	4.07,m	52.8	16,17,18	17b,18
16		168.7		
17a	1.77,m	37.4	15,16,19	15,17b
17b	1.36,m		15,16,18,20	15,17a
18	1.87,m	24.1	15,17,19	15,17b,20
19	0.86,d,(6.7)	22.8	17,20	
20	0.85,d,(6.6)	21.8	17,18,19	
2’	7.06,d,(2.3)	125.1	3’,4’,9’	1’-NH,12’a/b
3’		108.5		
4’		126.5		
5’	7.54,d,(8.4)	119.3	3’,9’	
6’	6.97,m	117.7	3,4’,8’	2,12a,,5’
7’		129.7		
8’	7.25,d,(1.7)	109.0	4’,6’	1’-NH,2,12a
9’		135.8		
11’	4.09,m	55.2	3’,12’,13’	14’-NH,12’a/b
12’a	3.20,dd,(14.5,4.5)	28.7	2’,4’,13’	11’,12’b
12’b	3.00,dd,(14.5,4.4)		2’,4’,13’	11’,12a’
13’		166.9		
15’	3.63,m	49.8	16’,17’	10’-NH,17’
16’		167.9		
17’	0.54,d,(7.0)	19.5	15’	15’
1-NH	6.70,s		3,4	8
14-NH	8.03,s		11,15,16,17	15,17b
1’-NH	10.84,d,(2.4)		2’,3’,4’,9’	2’,8’
10’-NH	7.91,d,(2.2)		11’,16’	
14’-NH	7.96,d,(2.2)		13’,15’	15’

Supplementary Table 14 NMR data of compound NAS-6 in DMSO-d_6

no.	δ_H	δ_C	**^{1}H-^{13}C HMBC**	**ROESY**
2	5.64,s	81.5	4,9	1-NH,12b,5',7'
3		58.4		
4		133.2		
5	7.13,dd,(7.5,1.2)	123.8	7,9	6,7
6	6.60,td,(7.4,1.0)	118.1	4,8	5,7
7	6.96,td,(7.6,1.3)	128.0	5,9	6,8
8	6.63,d,(7.4)	109.3	4,6	7
9		149.4		
11	4.06,m	58.7		12a
12a	3.02,m	40.3	2,4,11	11,12b
12b	2.64,m		4,11,13	5',12a
13		169.3		
15	4.08,m	52.7	16,17	14-NH,17a/b,18
16		166.5		
17a	1.79,m	38.3	15,16,19	15,17b,19
17b	1.42,m		15,16,20	17a,18,19
18	1.90,m	24.0	20	15,17b
19	0.86,d,(4.4)	22.8	17,18,20	15,17a/b,18
20	0.85,d,(4.4)	22.0	17,18,19	15,17a/b,18
2'	7.04,d,(2.3)	125.0	3',4',9'	1'-NH,12'a/b
3'		108.9		
4'		127.5		
5'	7.63,d,(1.8)	114.8	7',9'	8'
6'		134.3		
7'	6.99,dd,(8.5,1.8)	119.2	5',9'	2,12b
8'	7.20,d,(8.5)	111.3	4',6'	1'-NH,5'
9'		134.7		
11'	4.10,m	55.3		14'-NH,12'a/b
12'a	3.22,dd,(14.4,4.6)	28.7	2',3'	11',12'b
12'b	3.04,m		3',11',13'	11',12'a
13'		167.0		
15'	3.65,m	49.8	16',17'	10'-NH,17'
16'		167.9		
17'	0.54,d,(6.9)	19.6	15',16'	15'
1-NH	6.58,s		3,4	2
14-NH	8.04,s		11,16	15,17b
1'-NH	10.83,d,(2.4)		2',4',9'	2',8'
10'-NH	7.92,d,(2.2)		11',16'	15'
14'-NH	7.95,d,(2.2)		13',15'	11'

Supplementary Table 15 NMR data of compound NAS-7 in DMSO-d6

no.	δ_H	δ_C	^1H-^{13}C HMBC	ROESY
2	5.66,s	81.5	3,4,9,11	1-NH,12b,5',7'
3		58.3		
4		133.1		
5	7.12,d,(7.4)	123.8	3,7,9	7
6	6.60,m	118.1	4,8	5,7
7	6.96,m	128.0	5,9	
8	6.63,m	109.2	4,6	
9		149.4		
11	4.05,m	58.7	12,13	12a/b
12a	3.01,m	40.4	3,4	11,12b
12b	2.69,m		3,4,11,13	5',12a
13		168.9		
15	4.30,m	53.6	16,18	14-NH
16		165.0		
17	2.53,m	37.9	18	18a/b
18a	2.87,m	48.5	15,17,20	20
18b	2.76,m		15,17,20	20
20	2.13,m	22.9	18	18b
2'	7.05,d,(2.3)	124.9	3',4',9'	1'-NH,11',12'a/b
3'		108.9		
4'		127.5		
5'	7.64,s	114.9	3,3',7',9'	2
6'		134.3		
7'	6.97,m	119.2	3,5'	2,12a/b
8'	7.20,dd,(8.5,2.2)	111.3	4',6'	1'-NH,7'
9'		134.7		
11'	4.10,m	55.4	3',12',13'	14'-NH,12'a/b
12'a	3.22,dd,(14.5,4.6)	28.8	2',3',4',11',13'	11',12'b
12'b	3.05,dd,(14.4,4.6)		2',3',4',11',13'	11',12'a
13'		166.9		
15'	3.64,m	49.8	16',17'	10'-NH,17'
16'		167.8		
17'	0.53,d,(6.9)	19.6	15',16'	15',16'
1-NH	6.62,m		2,3,4,9	2
14-NH	8.24,s		11,13,15,16	15
1'-NH	10.84,d,(2.3)		2',3',4',9'	2',8'
10'-NH	7.93,d,(2.2)		11',15',16'	15'
14'-NH	7.97,m		11',13',15'	11'

Supplementary Table 16 NMR data of compound NAS-8 in DMSO-d6

no.	δ_H	δ_C	^1H-^{13}C HMBC	ROESY
2	5.66,s	81.5	4,9,11,	12b,5’,7’
3		58.3		
4		133.1		
5	7.12,d,(7.3)	123.8	7,9	6,7
6	6.62,m	118.1	4	7
7	6.96,m	128.0	9	
8	6.63,m	109.1	6	
9		149.4		
11	4.04,m	58.7		12a/b
12a	2.99,m	40.2		11,12b
12b	2.71,t,(12.1)		11,13	5’,12a
13		168.9		
15	4.32,m	52.9	16,18	14-NH
16		164.6		
17	2.54,m	40.4		18a/b
18a	3.25,m	49.7		20
18b	3.18,m			20
20	2.19,m	22.7		18b
2’	7.04,d,(2.4)	124.9	3’,4’	1’-NH,11’,12’a/b
3’		108.9		
4’		127.5		
5’	7.64,s	114.9	3,7’,9’	2
6’		134.2		
7’	6.97,m	119.2	3	2,12a/b
8’	7.20,d,(8.6)	111.3	4’,6’	1’-NH,7’
9’		134.7		
11’	4.11,m	55.4		14’-NH,12’a/b
12’a	3.21,dd,(14.6,5.0)	28.8	2’,3’,4’,11’	11’,12’b
12’b	3.05,dd,(14.4,4.6)		2’,3’,4’,11’,13’	11’,12’a
13’		166.9		
15’	3.64,m	49.8	16’,17’	10’-NH,17’
16’		167.7		
17’	0.53,d,(6.9)	19.5	16’	15’,16’
1-NH	6.62,m		3,4	2
14-NH	8.22,s		11,15,16	15
1’-NH	10.83,d,(2.4)		3’,4’	2’,8’
10’-NH	7.92,d,(2.3)		11’,13’	15’
14’-NH	7.97,d,(2.2)		15’,16’	11’,12’b

Supplementary Table 17 NMR data of compound NAS-9 in DMSO-d6

no.	δ_H	δ_C	^1H-^{13}C HMBC	ROESY
2	5.59,s	81.4	4,9,11,6’	1-NH,12b,5’,7’
3		58.4		
4		132.9		
5	7.05,dd,(7.5,1.4)	123.9	3,7,9	6,7
6	6.56,dd,(7.4,1.1)	118.0	4,8	7
7	6.94,td,(7.6,1.3)	127.9	5,9	8
8	6.58,m	108.8	4,6	7
9		149.5		
11	3.90,m	58.3	12,13	12a
12a	2.79,dd,(12.1,5.5)	40.8	2,4,11	11,12b
12b	2.00,t,(12.0)		3,4,13	5’,12a
13		167.8		
15	4.43,m	55.6	16,17,18	14-NH,17a/b
16		164.8		
17a	3.12,dd,(14.0,4.2)	36.2	15,16,18,19,23	15,19,23
17b	3.03,m		15,16,18,19,23	15,17a
18		136.7		
19	7.21,m	129.8	17,21,23	20,22
20	7.12,m	128.0	18,22	
21	7.09,m	126.3	19,23	
22	7.11,m	128.0	18,20	
23	7.20,m	129.8	17,19,21	
2’	7.07,d,(2.3)	124.9	3’,4’,9’	12’a/b
3’		109.0		
4’		127.3		
5’	7.45,d,(1.8)	114.8	3,3’,7’,9’	2
6’		134.3		
7’	6.86,dd,(8.6,1.8)	119.2	3,5’,9’	2,12a/b,8’
8’	7.20,m	111.4	6’	1’-NH,7’
9’		134.6		
11’	4.10,m	55.2	3’,12’,13’	10’-NH,14’-NH,12’a/b
12’a	3.22,dd,(14.5,4.9)	28.7	2’,3’,4’,11’,13’	11’,12’b
12’b	3.06,m		2’,3’,4’,11’,13’	11’,12’a
13’		167.0		
15’	3.68,m	49.9	16’,17’	14’-NH,17’
16’		168.0		
17’	0.62,d,(6.9)	19.6	15’,16’	15’
1-NH	6.64,s		4,9	2
14-NH	8.10,s		11,15,16	15
1’-NH	10.85,d,(2.4)		2’,3’,4’,9’	2’
10’-NH	7.93,m		11’,15’	15’
14’-NH	7.93,m		11’,15’	11’

Supplementary Table 18 NMR data of compound NAS-10 in DMSO-d6

no.	δ_H	δ_C	^1H-^{13}C HMBC	ROESY
2	5.64,d,(2.3)	81.5	4,9,12	1-NH,12b,5’,7’
3		58.3		
4		133.0		
5	7.13,dd,(7.4,2.3)	123.7	3,7,9	6,12a
6	6.59,m	118.0	8	5,7
7	6.97,m	128.0	5,9	5
8	6.63,dd,(8.0,2.4)	109.3	4,6	
9		149.5		
11	4.05,m	58.9	12,13	12a
12a	3.03,m	40.5	2,4	11,12b
12b	2.65,td,(12.0,2.4)		4,13,	12a
13		169.0		
15	4.15,m	50.2	16,17	14-NH
16		166.5		
17	1.25,m	15.7	15,16	15
2’	7.17,s	124.8	3’,4’,9’	1’-NH,12’a/b
3’		109.6		
4’		127.1		
5’	7.63,d,(2.4)	114.6	3,3’,7’,9’	2,12b
6’		134.2		
7’	7.02,dt,(8.7,2.2)	119.3	3,5’,9’	2,8’
8’	7.22,dd,(8.6,2.4)	111.4	4’,6’	1’-NH,7’
9’		134.8		
11’	4.30,m	55.1	3’,12’,13’	10’-NH,12’a/b
12’a	3.23,m	25.5	2’,3’,4’,11’,13’	11’,12’b
12’b	3.07,m		2’,3’,4’,11’,13’	11’,12a’
13’		165.6		
15’	4.09,m	58.5	16’,17’	17’a/b
16’		169.1		
17’a	2.00,m	27.7	19’	15’
17’b	1.45,m		16’,18’	17’a
18’a	1.71,m	21.9		17’a/b,19’a/b
18’b	1.64,m		11’	17’a/b,19’a/b
19’a	3.39,m	44.6	15’,17’,18’	19’b
19’b	3.28,m		15’,17’,18’	
1-NH	6.61,s		4,9	2
14-NH	8.18,s		11,13,15,16,17	15,17
1’-NH	10.81,d,(2.9)		2’,3’,4’,9’	2’
10’-NH	7.70,s		11’,12’,13’,15’,16’	11’,12’a/b

Supplementary Table 19 NMR data of compound NAS-11 in DMSO-d6

no.	δ_H	δ_C	^1H-^{13}C HMBC	ROESY
2	5.70,s	81.4	3,4,9,12,6’	1-NH,12b,5’,7’
3		58.3		
4		133.0		
5	7.09,d,(7.4)	123.7	3,7,9	6,7,12a
6	6.58,t,(7.4)	118.0	4,8	5,7
7	6.96,t,(7.5)	128.0	5,9	5,6,8
8	6.63,d,(7.9)	109.1	4,6	7
9		149.5		
11	4.02,m	58.2	12,13	12a
12a	3.04,dd,(12.4,5.8)	41.0	2,4,11	12b,15
12b	2.60,t,(12.0)		4,11,13,6’	12a
13		169.1		
15	4.00,m	59.5	16,17,18,19	14-NH,17,18,19
16		165.1		
17	2.36,m	28.5	15,16,18,19	15,18,19
18	1.00,d,(7.2)	18.1	15,17,19	15,17,19
19	0.86,d,(6.8)	16.5	15,17,18	17,18
2’	7.18,d,(2.3)	124.9	3’,4’,9’	1’-NH,12’a/b
3’		109.6		
4’		127.1		
5’	7.62,d,(1.8)	114.5	3,3’,7’,9’	2,12a/b
6’		134.4		
7’	7.03,dd,(8.5,1.9)	119.2	3,5’,9’	2,12a/b,8’
8’	7.24,d,(8.5)	111.5	4’,6’	1’-NH,7’
9’		134.8		
11’	4.30,t,(5.4)	55.1	3’,12’,13’	10’-NH,12’a/b
12’a	3.23,dd,(14.9,4.9)	25.5	2’,3’,4’,11’,13’	12’b
12’b	3.08,dd,(14.9,5.8)		2’,3’,4’,11’,13’	11’,12a’
13’		165.6		
15’	4.09,dd,(9.8,6.8)	58.5	16’,17’	17’a/b,18’a/b
16’		169.1		
17’a	2.00,m	27.7	19’	18’a/b
17’b	1.44,m		15’,18’	17’a,18’a/b
18’a	1.71,m	21.9		17’a/b,18’b
18’b	1.61,m			17’a/b,18’a
19’a	3.40,m	44.6	15’,17’,18’	18’a/b
19’b	3.28,m		17’,18’	19’a
1-NH	6.66,s		2,3,4,9	2
14-NH	7.99,s		11,13,15,16,17	15,17,18
1’-NH	10.83,d,(2.5)		2’,3’,4’,9’	2’
10’-NH	7.71,s		11’,12’,13’,15’,16’	11’,12’a/b

Supplementary Table 20 NMR data of compound NAS-12 in DMSO-d6

no.	δ_H	δ_C	^1H-^{13}C HMBC	ROESY
2	5.69,s	81.4	4,9,11,12,6’	1-NH,12b,5’,7’
3		58.3		
4		133.0		
5	7.09,d,(7.4)	123.7	3,7,9	6
6	6.58,t,(7.4)	117.9	4,5,7,8	5,7
7	6.96,m	128.0	5,9	6,8
8	6.62,d,(7.8)	109.1	4,6	7
9		149.5		
11	4.00,m	58.2	12,13	12a
12a	3.04,m	41.0	2,3,4	11,12b,5’
12b	2.59,t,(12.0)		3,4,6’	11,12a,5’
13		168.8		
15	4.04,m	59.1	16,17,18,20	14-NH,17a
16		165.1		
17	2.05,m	35.6	16,18,20	15,18a/b
18a	1.39,m	24.1	15,17,19,20	17
18b	1.31,m		15,17,19,20	17
19	0.82,t,(7.4)	12.2	17,18	17,18a/b,20
20	0.97,d,(7.0)	14.9	15,17,18	15,17,19
2’	7.18,s	124.9	3’.4’,9’	1’-NH,11’,12’a/b
3’		109.5		
4’		127.1		
5’	7.61,d,(1.9)	114.5	3,3’,7’,9’	2,12a/b
6’		134.5		
7’	7.03,dd,(8.5,1.8)	119.2	3,5’,9’	2,12a/b,8’
8’	7.24,dd,(8.6,1.6)	111.5	4’.6’	1’-NH,7’
9’		134.8		
11’	4.30,t,(5.5)	55.1	3’,12’,13’,16’	10’-NH,12’a/b
12’a	3.23,dd,(15.0,4.9)	25.5	2’,3’,4’,11’,13’	10’-NH,11’,12’b
12’b	3.08,dd,(15.0,5.8)		2’,3’,4’,11’,13’	10’-NH,11’,12’a
13’		165.6		
15’	4.09,m	58.4	16’,17’	17’a/b,18’a/b
16’		169.1		
17’a	2.00,m	27.7	18’,19’	15’,18’a/b
17’b	1.44,m		15’,16’,18’	17’b,18’a/b
18’a	1.71,m	21.9	15’,19’	15’,17’a/b,18’b,19’a/b
18’b	1.62,m		17’	15’,17’a/b,18’a,19’a/b
19’a	3.40,m	44.6	15’,17’,18’	17’a/b,18’a/b,19’b
19’b	3.28,m		17’,18’	17’a/b,18’a/b,19’a
1-NH	6.65,s		3,4,9	2
14-NH	7.99,s		11,13,16,17	15,17
1’-NH	10.82,d,(2.0)		2’,3’,4’,9’	
10’-NH	7.71,s		11’,12’,13’,15’,16’	2’,11’,12’a/b

Supplementary Table 21 NMR data of compound NAS-13 in DMSO-d6

no.	δ_H	δ_C	^{1}H-^{13}C HMBC	ROESY
2	5.65,s	81.6	4,9,6'	12b,5',7'
3		58.3		
4		133.0		
5	7.13,dd,(7.4,1.2)	123.7	3,7,9	6,7
6	6.60,dd,(7.4,1.1)	118.0	4,8	5,7
7	6.97,m	128.0	5,9	6
8	6.64,m	109.3	4,6	7
9		149.4		
11	4.08,m	58.7	12,13	12a
12a	3.04,m	40.4	4	11,12b
12b	2.65,t,(12.6)		4,13,6'	12a,5'
13		169.3		
15	4.10,m	52.7	16,17,18	14-NH,17a
16		166.4		
17a	1.80,m	38.2	15,16	
17b	1.42,m		16	
18	1.91,m	23.9	17	15,17b
19	0.86,d,(6.6)	22.8	17,18	
20	0.86,d,(6.6)	22.8	17,18	
2'	7.18,d(2.4)	124.9	3'.4',9'	1'-NH,11',12'a/b
3'		109.5		
4'		127.1		
5'	7.63,d,(1.8)	114.6	3,3',7',9'	2,12a/b
6'		134.2		
7'	7.03,dd,(8.5,1.8)	119.2	3,5',9'	2,12a/b
8'	7.23,d,(8.5)	111.4	4'.6'	1'-NH,7'
9'		134.8		
11'	4.30,t(5.4)	55.1	3',12',13'	10'-NH,12'a/b
12'a	3.23,dd,(14.9,4.8)	25.5	2',3',4',13'	11',12'b
12'b	3.08,m		2',3',4',13'	11',12'a
13'		165.6		
15'	4.09,m	58.5	16'	17'a/b
16'		169.1		
17'a	2.00,m	27.7	19'	15',17'a
17'b	1.46,m		16',19'	
18'a	1.71,m	21.9		
18'b	1.62,m			
19'a	3.40,m	44.6	18'	17'a,18'a/b
19'b	3.29,m		15'.17'	17'a,18'a/b
1-NH	6.62,s		3,4,9	
14-NH	8.06,s		11,13,15,16,17	15,17a
1'-NH	10.82,d,(2.4)		2',3',4',9'	
10'-NH	7.71,s		11',12',13',15',16'	11',12'a/b

Supplementary Table 22 NMR data of compound NAS-14 in DMSO-d6

no.	δ_H	δ_C	^{1}H-^{13}C HMBC	ROESY
2	5.69,s	81.6	4,9,12	12b,5',7'
3		58.3		
4		133.0		
5	7.12,d,(7.4)	123.7	3,7,9	6,12a
6	6.60,t,(7.4)	118.0	4,7,8	5,7
7	6.97,t, (7.6)	128.1	5,9	5
8	6.64,d,(3.4)	109.3	4,6	7
9		149.4		
11	4.06,m	58.7	12,13	12a
12a	3.04,m	40.6	2,3,4	11,12b
12b	2.66,m		4,13	12a,5'
13		169.0		
15	4.30,m	53.5	16,18	14-NH
16		165.0		
17	2.53,m	37.9	18	
18a	2.87,m	48.5		
18b	2.75,m			
20	2.14,m	22.9		
2'	7.17,d,(2.3)	124.9	3',4',9'	1'-NH,12'a/b
3'		109.5		
4'		127.1		
5'	7.63,d,(1.8)	114.6	3,3',7',9'	2,12b
6'		134.2		
7'	7.03,m	119.2	3,5',9'	2,8'
8'	7.23,d,(8.5)	111.5	4',6'	1'-NH,7'
9'		134.8		
11'	4.30,m	55.1	3',12'	10'-NH,12'a/b
12'a	3.23,dd,(14.9,4.8)	25.5	2',3',4',11',13'	11',12'b
12'b	3.08,m		2',3',4',11',13'	11',12a'
13'		165.6		
15'	4.09,m	58.5	16',17'	17'a/b
16'		169.1		
17'a	2.00,m	27.7	19'	
17'b	1.45,m		15',16',18'	
18'a	1.71,m	21.9	17',19'	
18'b	1.63,m			
19'a	3.40,m	44.6	17',18'	
19'b	3.29,m		17',18'	
1-NH	6.63,s		4,9	
14-NH	8.24,s		11,13,15,16	15
1'-NH	10.84,d,(2.4)		2',3',4',9'	2'
10'-NH	7.71,s		11',12',13',15',16'	12'a/b

Supplementary Table 23 NMR data of compound NAS-15 in DMSO-d6

no.	δ_H	δ_C	**^{1}H-^{13}C HMBC**	**ROESY**
2	5.66,s	81.6	4,9,11	1-NH,12b,5',7'
3		58.3		
4		133.0		
5	7.11,d,(7.4)	123.7	6,9	6,12a
6	6.59,t,(7.4)	118.0	4,8	5,7
7	6.97,t, (7.6)	128.1	5,9	5
8	6.63,d,m	109.3	4,6,9	
9		149.5		
11	4.06,m	58.7	12,13	12a
12a	3.02,m	40.6	2,3,4	11,12b
12b	2.64,t,(12.0)		4,13	12a
13		169.1		
15	4.21,m	53.4	16,18	14-NH
16		165.5		
17	2.56,m	28.8	18	15
18	2.87,m	38.1		
19	2.03,m	14.5		
2'	7.17,s	124.9	3',4',9'	1'-NH,12'a/b
3'		109.5		
4'		127.1		
5'	7.61,s	114.6	3,3',7',9'	2,12b
6'		134.2		
7'	7.03,d,(8.5)	119.2	3,5',9'	2,8'
8'	7.23,d,(8.6)	111.5	4',6'	1'-NH,7'
9'		134.8		
11'	4.30,m	55.1	3',12'	10'-NH,12'a/b
12'a	3.23,m	25.5	2',3',4',11',13'	11',12'b
12'b	3.07,m		2',3',4',11',13'	11',12a'
13'		165.6		
15'	4.09,m	58.5	16',17'	17'a/b
16'		169.1		
17'a	1.99,m	27.7	19'	15'
17'b	1.46,m		15',16',18'	17'a
18'a	1.71,m	21.9	17',19'	17'a/b,19'a/b
18'b	1.63,m			17'a/b,19'a/b
19'a	3.39,m	44.6	17',18'	19'b
19'b	3.29,m		17',18'	
1-NH	6.63,m		4,9	2
14-NH	8.20,s		11,13,15,16	15,17
1'-NH	10.86,d,(2.4)		2',3',4',9'	2'
10'-NH	7.70,s		11',12',13',15',16'	11',12'a/b

Supplementary Table 24 NMR data of compound NAS-16 in DMSO-d6

no.	δ_H	δ_C	^1H-^{13}C HMBC	ROESY
2	5.60,s	81.5	3,4,9	1-NH,12b,5',7'
3		58.4		
4		132.7		
5	7.03,dd,(7.4,1.3)	123.7	3,7,9	6
6	6.56,td,(7.4,1.1)	117.9	4,8	5,7
7	6.95,td,(7.6,1.3)	128.0	5,9	6,8
8	6.59,dd,(7.9,1.0)	108.9	4,6	1-NH,7
9		149.6		
11	3.90,m	58.3	12,13	12a
12a	2.81,dd,(12.0,5.4)	41.0	2,3	11,12b
12b	1.97,t,(11.9)		4,11	12a,5'
13		167.6		
15	4.44,m	55.6	13,18	14-NH,17a/b,23
16		164.7		
17a	3.14,dd,(14.0,4.0)	36.2	18,23	15,17b
17b	3.01,dd,(14.0,5.5)		15,16,18,23	15,17a
18		136.7		
19	7.11,m	127.9		23
20	7.11,m	127.9		22
21	7.09,m	126.3	23	
22	7.20	129.9		20
23	7.21	129.9		19
2'	7.19,d,(2.3)	124.7	3'.4'.9'	1'-NH
3'		109.6		
4'		127.0		
5'	7.45,d,(1.8)	114.6	3,3',7',9'	2,12a/b
6'		134.2		
7'	6.88,dd,(8.5,1.8)	119.2	3,5',9'	2,12b,8'
8'	7.23,d,(8.4)	111.4	4',6'	1'-NH,7'
9'		134.7		
11'	4.32,t,(5.5)	55.0	3',12',13'	10'-NH,12'a/b
12'a	3.24,m	25.4	2',3',4',11',13'	11',12'b
12'b	3.07,dd,(14.9,6.0)		2',3',4',11',13'	11',12'a
13'		165.6		
15'	4.11,m	58.5	16',17'	17'a/b,18'a
16'		169.2		
17'a	2.02,m	27.6		15',17'b,18'a
17'b	1.50,m		15'	17'a,18'a/b,19'a
18'a	1.72,m	21.9		15',17'a/b,18'b,19'a/b
18'b	1.63,m			15',17'a/b,18'a,19'a/b
19'a	3.39,m	44.6	18'	19'b
19'b	3.30,m			19'a
1-NH	6.67,s		3,4,9	2,8
14-NH	8.13,s		11,13,15,16	15,17a/b
1'-NH	10.82,d,(2.4)		2',3',4',9'	
10'-NH	7.73,s		11',13',15',16'	11',12'a/b

Supplementary Table 25 NMR data of compound NAS-17 in DMSO-d6

no.	δ_H	δ_C	^{1}H-^{13}C HMBC	ROESY
2	5.60,s	81.9	3,4,9,11,7’	1-NH,12b,6’,8’
3		58.5		
4		132.4		
5	7.10,dd,(7.4,1.1)	124.0	3,7,9	6
6	6.61,m	118.0	4,8	5,7
7	6.98,td,(7.6,1.2)	128.2	5,9	8
8	6.62,m	109.1	4,6	7
9		149.6		
11	4.01,m	58.3	12,13	2,12a
12a	3.01,dd,(12.4,5.7)	41.2	2,3,4	12b
12b	2.54,m		3,4,11,13,7’	6’,8’,12a
13		169.0		
15	4.00,m	59.5	16,17,18,19	14-NH,17,18,19
16		165.2		
17	2.36,m	28.5	15,16,18,19	15,18,19
18	1.00,d,(7.2)	18.1	15,17,19	15,17,19
19	0.87,d,(6.9)	16.5	15,17,18	15,17,18
2’	7.04,d,(2.4)	124.8	3’,4’,9’	1’-NH
3’		108.8		
4’		126.6		
5’	7.51,d,(8.4)	119.2	7’,9’	6’
6’	7.01,dd,(8.4,1.7)	116.2	3,4’,8’	2,12b,5’
7’		136.9		
8’	7.21,d,(1.6)	107.9	3,4’,6’	1’-NH,2,12a/b
9’		136.1		
11’	4.08,m	55.2	3’,12’,13’	14’-NH,12’b
12’a	3.12,dd,(14.5,5.5)	29.0	2’,3’,4’,11’,13’	11’,12’b
12’b	3.05,dd,(14.5,4.5)		2’,3’,4’,11’,13’	11’,12’a
13’		167.4		
15’	3.44,m	59.4	16’,17’,18’,19’	10’-NH,17’,18’,19’
16’		166.4		
17’	1.57,m	31.2	18’,19’	15’,18’,19’
18’	0.60,d,(7.1)	18.4	15’,17’,19’	15’,17’,19’
19’	0.23,d,(6.9)	16.4	15’,17’,18’	15’,17’,18’
1-NH	6.67,s		3,4,9	2
14-NH	8.01,s		11,13,15,16	15
1’-NH	10.78,d,(2.5)		2’,3’,4’,9’	2’,8’
10’-NH	7.87,d,(2.4)		11’,16’	15’
14’-NH	7.90,d,(2.4)		13’,15’	11’

Supplementary Table 26 NMR data of compound NAS-18 in DMSO-d6

no.	δ_H	δ_C	^{1}H-^{13}C HMBC	ROESY
2	5.68,s	81.3	3,4,9,11,6’	1-NH,5’,7’
3		58.4		
4		133.3		
5	7.09,m	123.8	3,7,9	6
6	6.58,t,(7.3)	118.1	4,8	7
7	6.94,t,(7.5)	127.9	5,9	6,8
8	6.61,d,(7.7)	109.0	4,6	7
9		149.4		
11	4.00,m	58.2	12,13	12a
12a	3.03,dd,(12.4,5.7)	41.0	2,3,4	11,12b
12b	2.60,t,(12.0)		3,4,13	5’,12a
13		169.1		
15	3.99,m	59.6	16,17,18,19	14-NH,17,18,19
16		165.1		
17	2.35,m	28.5	15,16,18,19	15,18,19
18	1.00,d,(6.7)	18.1	15,17,19	15,17,19
19	0.86,d,(6.4)	16.5	15,17,18	15,17,18
2’	7.08,m	125.0	3’,9’	1’-NH,12’a
3’		109.2		
4’		127.6		
5’	7.65,s	114.7	3,3’,7’,9’	2,12a/b
6’		134.6		
7’	6.97,d,(8.5)	119.0	3,5’,9’	2,8’
8’	7.18,d,(8.2)	111.4	4’,6’	1’-NH,7’
9’		134.9		
11’	4.13,m	55.1	3’,12’,13’	14’-NH,12’a/b
12’a	3.20,dd,(14.4,5.5)	28.8	2’,3’,4’,11’	11’,12’b
12’b	3.08,dd,(14.7,5.1)		2’,3’,4’,11’,13’	11’,12’a
13’		167.6		
15’	3.52,m	59.4	16’,19’	10’-NH,17’,18’,19’
16’		166.4		
17’	1.71,m	31.1	18’	15’,18’,19’
18’	0.67,d,(6.7)	18.5	15’,17’,19’	15’,17’,19’
19’	0.20,d,(6.6)	16.2	15’,17’,18’	17’,18’
1-NH	6.64,s		2,3,4,9	2
14-NH	8.00,s		11,13,15,16	15
1’-NH	10.81,s		2’,3’,4’,9’	2’,8’
10’-NH	7.90,d,(2.8)		11’,15’,16’	15’
14’-NH	7.93,s		13’,15’	11’

Supplementary Table 27 NMR data of compound NAS-19 in DMSO-d6

no.	δ_H	δ_C	^1H-^{13}C HMBC	ROESY
2	5.60,s	81.9	3,4,9,11,7’	1-NH,12b,6’,8’
3		58.4		
4		132.3		
5	7.10,dd,(7.4,1.2)	123.9	3,7,9	
6	6.60,dd,(7.3,1.0)	117.9	8	5,7
7	6.98,td,(7.6,1.3)	128.1	5,9	8
8	6.63,d,(7.7)	109.1	4,6	7
9		149.6		
11	4.00,m	58.2	12,13	12a
12a	3.01,dd,(12.4,5.7)	41.3	2,3,4	11,12b
12b	2.54,t,(12.0)		3,4,13,7’	6’,8’,12a
13		168.8		
15	4.04,d,(2.2)	59.1	16,17,18,20	14-NH,17,18a/b,19
16		165.1		
17	2.04,m	35.6		15,18a/b,20
18a	1.39,m	24.1	15,17,19,20	17,20
18b	1.31,m		17,19,20	17,20
19	0.83,t,(7.5)	12.2	17,18	17,18a/b,20
20	0.97,d,(7.2)	15.0	15,17,18	15,17,18a/b
2’	7.05,d,(2.4)	124.8	3’,4’,9’	1’-NH,12’a/b
3’		108.8		
4’		126.6		
5’	7.51,d,(8.4)	119.2	3’,4’	6’
6’	7.00,dd,(8.4,1.7)	116.2	3,4’,8’	2,5’
7’		136.9		
8’	7.21,d,(1.6)	107.8	3,4’,6’	1’-NH,2,12a/b
9’		136.0		
11’	4.08,m	55.2	3’,13’	14’-NH,12’a/b
12’a	3.12,dd,(14.5,5.5)	29.0	3’,4’,11’,13’	11’,12’b
12’b	3.05,dd,(14.5,4.5)		3’,4’,11’,13’	11’,12’a
13’		167.4		
15’	3.44,m	59.4	16’,17’,18’,19’	10’-NH,17’,18’,19’
16’		166.3		
17’	1.57,m	31.2	15’,18’,19’	15’,18’,19’
18’	0.61,d,(7.0)	18.4	15’,17’,19’	15’,17’,19’
19’	0.24,d,(6.8)	16.4	15’,17’,18’	15’,17’,18’
1-NH	6.65,s		3,4,9	2
14-NH	8.00,s		13,16	15,17
1’-NH	10.77,d,(2.4)		2’,3’,4’,9’	2’,8’
10’-NH	7.86,d,(2.4)		11’,15’,16’	15’
14’-NH	7.89,d,(2.4)		13’,15’	11’

Supplementary Table 28 NMR data of compound NAS-20 in DMSO-d6

no.	δ_H	δ_C	^1H-^{13}C HMBC	ROESY
2	5.67,s	81.4	4,9,11,12,6’	1-NH,12b,5’,7’
3		58.3		
4		133.2		
5	7.07,m	123.8	3	6
6	6.58,td,(7.4,1.1)	118.1	4,8	7
7	6.94,td,(7.6,1.3)	127.9	5,9	5,6,8
8	6.62,m	109.0	4,6,9	7
9		149.4		
11	3.99,m	58.1	12,13	12a
12a	3.02,dd,(12.4,5.6)	41.0	2,3,4,11	11,12b
12b	2.60,t,(12.0)		3,4,11,13,6’	5’,12a
13		168.8		
15	4.04,t,(2.2)	59.1	16,17,18,20	14-NH,17,18a/b,20
16		165.1		
17	2.04,m	35.7		15,18a/b,20
18a	1.38,m	24.1	17,19,20	17
18b	1.30,m		17,19,20	17
19	0.82,t,(7.4)	12.2	17,18	17,18a/b
20	0.97,d,(7.1)	14.9	15,17,18	17,18a/b
2’	7.08,m	125.0	3’,9’	1’-NH,12’a
3’		109.2		
4’		127.5		
5’	7.65,d,(1.8)	114.6	3,3’,7’,9’	2,12a/b
6’		134.6		
7’	6.97,dd,(8.5,1.8)	118.9	3,5’,9’	8’
8’	7.18,d,(8.6)	111.3	4’,6’	1’-NH,7’
9’		134.9		
11’	4.13,m	55.0	3’,12’,13’	14’-NH,12’a/b
12’a	3.20,dd,(14.4,5.4)	28.8	2’,3’,4’,11’,13’	11’,12’b
12’b	3.09,dd,(14.4,4.7)		2’,3’,4’,11’,13’	11’,12’a
13’		167.5		
15’	3.52,m	59.4	17’,18’,19’	10’-NH,17’,18’
16’		166.4		
17’	1.72,m	31.1	15’,16’,18’,19’	15’,18’,19’
18’	0.68,d,(7.0)	18.5	15’,17’,19’	17’,19’
19’	0.23,d,(6.7)	16.3	15’,17’,18’	17’,18’
1-NH	6.61,m		4	
14-NH	7.98,s		13,16	15
1’-NH	10.80,d,(2.4)		2’,3’,4’,9’	2’
10’-NH	7.90,m		11’,16’	15’
14’-NH	7.91,m		13’,15’	11’

Supplementary Table 29 NMR data of compound NAS-21 in DMSO-d6

no.	δ_H	δ_C	**^{1}H-^{13}C HMBC**	**ROESY**
2	5.55,s	81.9	4,9,7'	1-NH,12b,6',8'
3		58.4		
4		132.4		
5	7.13,dd,(7.5,1.2)	123.9	3,7,9	6
6	6.62,m	118.0	4	5,7
7	6.98,dd,(7.5,1.4)	128.1	5,9	8
8	6.64,m	109.4	4	7
9		149.5		
11	4.08,m	58.7	13	12a
12a	3.01,dd,(12.5,6.0)	40.5	2,3,4	11,12b
12b	2.60,m		4,11,13,7'	8',12a
13		169.2		
15	4.09,m	52.7	16,17,18	14-NH,17a/b
16		166.5		
17a	1.79,m	38.2	16,19,20	17b,18
17b	1.43,m		15,18,19	17a
18	1.90,m	23.9	17,19,20	17a/b,19
19	0.86,dd,(6.3,2.5)	22.7	17,18,20	17a/b,18
20	0.85,dd,(6.3,2.5)	22.0	17,18,19	18
2'	7.04,d,(2.4)	124.8	3',4',9'	1'-NH
3'		108.8		
4'		126.6		
5'	7.51,d,(8.4)	119.1	3',4',7',9'	6'
6'	7.03,dd,(8.5,1.7)	116.2	3,8'	2,5'
7'		136.7		
8'	7.20,d,(1.7)	107.9	3,4',6'	1'-NH,2,12a/b
9'		136.0		
11'	4.07,m	55.2	3',12',13'	14'-NH,12'a/b
12'a	3.12,dd,(14.5,5.5)	29.0	3',4',11',13'	11',12'b
12'b	3.05,dd,(14.6,4.8)		3',4',11',13'	11',12'a
13'		167.4		
15'	3.45,m	59.4	16',17',18',19'	10'-NH,17',18'
16'		166.3		
17'	1.59,td,(7.0,4.1)	31.2	18',19'	18'
18'	0.61,d,(7.0)	18.4	15',17',19'	17',19'
19'	0.25,d,(6.8)	16.4	15',17',18'	17',18'
1-NH	6.60,m			
14-NH	8.05,s		11,15,16	11,15,17b
1'-NH	10.76,d,(2.4)		2',3',4',9'	2',8'
10'-NH	7.86,d,(2.4)		11',15',16'	15'
14'-NH	7.89,d,(2.4)		13',15'	

Supplementary Table 30 NMR data of compound NAS-22 in DMSO-d6

no.	δ_H	δ_C	**^{1}H-^{13}C HMBC**	**ROESY**
2	5.65,s	81.4	4,9	1-NH,12b,5’,7’
3		58.4		
4		133.2		
5	7.12,dd,(7.4,1.2)	123.8	3,7,9	6,7
6	6.60,dd,(7.5,1.1)	118.1	8	5,7
7	6.95,td,(7.6,1.3)	127.9	5,9	6,8
8	6.62,dd,(7.8,0.9)	109.3	4,6	7
9		149.3		
11	4.07,m	58.7	12,13	12a
12a	3.04,dd,(12.5,5.9)	40.4	2,3,4	11,12b
12b	2.63,t,(12.6)		3,4,13	5’,11,12a
13		169.3		
15	4.08,m	52.7	16,17,18	14-NH,17a/b,18
16		166.5		
17a	1.79,m	38.3	15,16,19	17b,19,20
17b	1.43,m		15,16,18,20	17a,19,20
18	1.90,m	24.0	15,17,19,20	17b,19,20
19	0.86,d,(6.5)	22.8	17,18,20	15,17a/b,18
20	0.85,d,(6.5)	22.0	17,18,19	15,17a/b,18
2’	7.08,d,(2.4)	125.0	3’,4’,9’	1’-NH,12’a/b
3’		109.2		
4’		127.6		
5’	7.66,d,(1.9)	114.7	3,3’,7’,9’	12a/b
6’		134.4		
7’	6.99,dd,(8.6,1.8)	119.0	3,5’,9’	2,12a/b,8’
8’	7.18,d,(8.5)	111.3	4’,6’	1’-NH,7’
9’		134.9		
11’	4.12,m	55.1		14’-NH,12’a/b
12’a	3.20,dd,(14.4,5.4)	28.8	2’,3’,4’,11’,13’	11’,12’b
12’b	3.08,dd,(14.4,4.8)		2’,3’,4’,11’,13’	11’,12’a
13’		167.6		
15’	3.51,m	59.4	16’,17’,18’,19’	10’-NH,17’,18’
16’		166.4		
17’	1.70,td,(7.0,3.9)	31.1	15’,18’,19’	15’,18’,19’
18’	0.67,d,(7.1)	18.5	15’,17’,19’	15’,17’,19’
19’	0.22,d,(6.8)	16.3	15’,17’,20’	15’,17’,18’
1-NH	6.58,s		3,4,9	
14-NH	8.05,s		11,13,15,16,17	11,17b
1’-NH	10.80,d,(2.4)		2’,3’,4’,9’	2’
10’-NH	7.90,d,(2.5)		11’,16’	15’
14’-NH	7.91,d,(2.3)		13’,15’	11’

Supplementary Table 31 NMR data of compound NAS-23 in DMSO-d6

no.	δ_H	δ_C	**^{1}H-^{13}C HMBC**	**ROESY**
2	5.58,s	81.9	4,9,11,7’	1-NH,12b,6’,8’
3		58.4		
4		132.4		
5	7.13,d,(7.3)	123.9	3,7,9	6,7
6	6.62,m	117.9	4,8	5
7	6.98,t,(7.1)	128.2	5,9	8
8	6.63,m	109.3	6	7
9		149.5		
11	4.05,m	58.8	12,13	2,12a
12a	3.00,dd,(12.4,5.9)	40.7	2,3,4	11,12b,
12b	2.60,m		11	2,12a, 6’,8’
13		169.0		
15	4.21,m	53.4	16,17,18	14-NH,17a/b,18a/b
16		165.5		
17a	2.07,m	29.0	15,18	14-NH ,15,17b,18a/b
17b	1.95,m		15,18	14-NH ,15,17a,18a/b
18a	2.59,m	28.9	15,17,20	15,17a/b
18b	2.55,m		15,17,20	15,17a/b
20	2.03,s	14.4	18	18a/b
2’	7.04,m	124.8	3’,4’,9’	1’-NH,11’,12’a/b
3’		108.8		
4’		126.6		
5’	7.51,d,(8.4)	119.1	3’,4’,7’,9’	8’,12’a/b
6’	7.03,m	116.3	8’	2,12a/b,5’
7’		136.7		
8’	7.20,d,(1.7)	107.9	3,4’,6’	2,12a/b,1’-NH
9’		136.0		
11’	4.07,m	55.2	3’,12’,13’	2’,14’-NH,12’a/b
12’a	3.12,dd,(14.5,5.5)	29.0	2’,3’,4’,11’,13’	2’,11’,12’b
12’b	3.05,dd,(14.5,4.4)		2’,3’,4’,11’,13’	2’,11’,12’a
13’		167.4		
15’	3.45,m	59.4	16’,17’,18’,19’	10’-NH,17’,18’
16’		166.4		
17’	1.59,m	31.2	15’,16’,18’,19’	15’,18’,19’
18’	0.61,d,(7.1)	18.4	15’,17’,19’	15’,17’,19’
19’	0.24,d,(6.8)	16.4	15’,17’,18’	15’,17’,18’
1-NH	6.65,s		3,4,9	2
14-NH	8.20,s		11,13,15,16,17	12b,15,17a/b
1’-NH	10.77,d,(2.4)		2’,4’	2’,8’
10’-NH	7.87,d,(2.4)		11’,15’,16’	15’
14’-NH	7.90,d,(2.3)		11’,13’,15’	11’

Supplementary Table 32 NMR data of compound NAS-24 in DMSO-d6

no.	δ_H	δ_C	^{1}H-^{13}C HMBC	ROESY
2	5.66,s	81.4	4,9	1-NH,11,12b,5’,7’
3		58.3		
4		133.2		
5	7.11,d,(7.5)	123.8	3,7,9	6,7
6	6.59,t,(7.4)	118.1	4,7,8	5,7
7	6.95,td,(7.6,1.3)	127.9	5,9	6,8
8	6.61,m	109.2	6	7
9		149.3		
11	4.05,m	58.6	12,13	2,12a
12a	3.02,dd,(12.4,5.8)	40.6	2,3,4,11	11,12b,5’
12b	2.63,t,(12.0)		3,4,11,13	2,12a, 5’,7’
13		169.1		
15	4.21,m	53.4	16,17,18	14-NH,17a/b,18a/b
16		165.4		
17a	2.06,m	29.1	15,16,18	15,17b,18a
17b	1.94,m		15,16,18	15,17a,18a
18a	2.59,ddd,(12.9,9.6,5.9)	28.8	15,17,20	15,17a/b
18b	2.54,m		15,17,20	15,17a/b
20	2.03,s	14.3	18	17b,18a/b
2’	7.07,d,(2.3)	125.0	3’,4’,9’	1’-NH,11’,12’a/b
3’		109.2		
4’		127.6		
5’	7.65,d,(1.8)	114.7	3,3’,7’,9’	2,12a/b
6’		134.4		
7’	6.98,dd,(8.5,1.9)	119.0	3,5’,9’	2,12a/b,8’
8’	7.18,d,(8.5)	111.3	4’,6’	1’-NH,7’
9’		134.9		
11’	4.12,m	55.1	3’,12’,13’	2’,14’-NH,12’a/b
12’a	3.20,dd,(14.4,5.4)	28.8	2’,3’,4’,11’,13’	2’,11’,12’b
12’b	3.08,dd,(14.4,4.7)		2’,3’,4’,11’,13’	2’,11’,12’a
13’		167.6		
15’	3.51,m	59.4	16’,17’,18’,19’	10’-NH,17’,18’
16’		166.4		
17’	1.71,pd,(7.2,2.1)	31.1	18’,19’	15’,18’,19’
18’	0.67,d,(7.1)	18.5	15’,17’,19’	15’,17’,19’
19’	0.21,d,(6.8)	16.5	15’,17’,18’	15’,17’,18’
1-NH	6.62,m		3,4,9	2
14-NH	8.19,s		11,15,16	15,17a/b
1’-NH	10.81,d,(2.3)		2’,3’,4’,9’	2’,8’
10’-NH	7.91,d,(2.4)		11’,16’	15’
14’-NH	7.92,d,(2.3)		13’,15’	11’

Supplementary Table 33 NMR data of compound NAS-25 in DMSO-d6

no.	δ_H	δ_C	**^{1}H-^{13}C HMBC**	**ROESY**
2	5.61,s	81.4	4,9,11	1-NH,11,5',7'
3		58.4		
4		133.0		
5	7.03,dd,(7.5,1.2)	123.9	3,7,9	6
6	6.55,td,(7.4,1.0)	118.0	8	7
7	6.93,td,(7.6,1.3)	127.9	5,9	8
8	6.57,d,(7.9)	108.8	4,6	7
9		149.5		
11	3.88,m	58.2	12,13	12a
12a	2.78,dd,(12.1,5.4)	41.0	2,3,4	11,12b
12b	1.93,t,(12.0)		3,4,11,13	12a,
13		167.6		
15	4.43,t,(4.6)	55.7	16,17,18	14-NH,17a/b
16		164.7		
17a	3.13,m	36.3	15,16,18,19,23	15,17b
17b	3.01,dd,(13.9,5.4)		15,16,18,19,23	15,17a
18		136.6		
19	7.19,m	129.8	17,21,23	17a/b,20
20	7.09,m	128.0	18,22	
21	7.07,m	126.3	19,20,22,23	
22	7.08,m	128.0	18,20	
23	7.19,m	129.8	17,19,21	17a/b,19
2'	7.10,m	124.9	3',4',9'	1'-NH,12'a/b
3'		109.2		
4'		127.4		
5'	7.47,d,(1.8)	114.7	3',7',9'	2,12a/b
6'		134.3		
7'	6.85,dd,(8.5,1.9)	119.0	3,5',9'	2,8'
8'	7.18,m	111.3	6'	1'-NH,7'
9'		134.8		
11'	4.12,m	55.0	3',12',13'	14'-NH12'a/b
12'a	3.19,dd,(14.4,5.8)	29.0	2',3',4',11',13'	11',12'b
12'b	3.10,m		2',3',4',11',13'	11',12'a
13'		167.6		
15'	3.53,t,(2.4)	59.4	17',18',19'	10'-NH,17',18',19'
16'		166.4		
17'	1.76,ddtt,(10.1,7.0,3.0)	31.2	15',16',18',19'	15',18',19'
18'	0.71,d,(7.0)	18.6	15',17',19'	15',17',19'
19'	0.27,d,(6.7)	16.3	15',17',18'	17',18'
1-NH	6.64,s		3,4,9	2
14-NH	8.11,s		11,13,15,16	15,17a/b
1'-NH	10.82,d,(2.4)		2',3',4',9'	2'
10'-NH	7.92,d,(2.5)		11',16'	15'
14'-NH	7.87,d,(2.4)		13',15'	11'

Supplementary Table 34 NMR data of compound NAS-26 in DMSO-d6

no.	**δ_H**	**δ_C**	**^1H-^{13}C HMBC**	**ROESY**
2	5.61,s	85.1	9,7’	11,6’,8’
3		59.4		
4		134.6		
5	6.80,d,(7.3)	123.8	7,9	6
6	6.58,m	118.2	4,8	5,7
7	6.99,t,(7.6)	128.0	5,9	6,8
8	6.63,d,(7.9)	109.5	4,6	5,7
9		148.2		
11	4.59,m	58.2		12a
12a	3.03,m	39.1	7’	12b
12b	2.34,dd,(13.7,9.7)		11,13	12a,
13		169.9		
15	4.29,t,(5.5)	56.1	16,18	17a/b
16		167.9		
17a	3.01,m	33.5	15,18,19,23	17b
17b	2.87,dd,(14.6,5.6)		15,18,19,23	17a
18		127.5		
19	7.08,m	130.7	17,21,23	20
20	6.59,m	115.0	18,22	19
21		155.9		
22	7.09,m	115.0	18,20	23
23	6.59,m	130.7	17,19,21	19
2’	7.06,d,(2.3)	125.2	3’,4’,9’	1’-NH,11’,12’a/b
3’		108.9		
4’		126.7		
5’	7.54,d,(8.4)	119.5	3’,7’	6’
6’	6.94,d,(8.4)	117.7	3,4’,8’	2,12a,5’
7’		135.9		
8’	7.20,s	109.0	3,4’,6’	1’-NH,2,12a/b
9’		136.2		
11’	4.10,m	55.3		12’a/b
12’a	3.16,dd,(14.4,5.4)	29.1	3’,4’	
12’b	3.05,m		3’	
13’		167.6		
15’	3.44,m	59.5		
16’		166.6		
17’	1.54,m	31.4		18’,19’
18’	0.60,d,(7.0)	18.5	15’,17’,19’	17’,19’
19’	0.20,d,(6.9)	16.5	15’,17’,18’	17’,18’
1-NH	7.09,m			2
14-NH	7.80,s		11,15,16	15,17b
1’-NH	10.78,d,(2.3)		2’,3’,4’,9’	8’
10’-NH	7.84,d,(2.4)		11’,16’	15’
14’-NH	7.91,d,(2.3)		13’,15’	11’

Supplementary Table 35 NMR data of compound NAS-27 in DMSO-d6

no.	δ_H	δ_C	^{1}H-^{13}C HMBC	ROESY
2	5.84 s	80.3,80.4	3,4,9,11,12,6’	1-NH,11,12a/b,5’
3		56.5		
4		126.4		
5	7.27,dd,(8.2,5.7)	125.6,125.7	3,7,9	6,12a/b
6	6.44,m	103.9,104.0	4,7,8	5,8
7		162.7,163.8		
8	6,37,dd,(10.2,2.4)	96.3,96.5	4,6,7,9	1-NH,6
9		151.5,151.6		
11	4.26,m	59.6	12,13	2,12a/b
12a	2.96,m	40.5,40.6	2,3,4,11	11,12b,5’
12b	2.67,d,(13.0)		3,4,11,13,6’	2,11,12a,5’
13		165.3		
15	4.33,m	59.7	16,17	17a/b,18a/b,19b
16		166.1		
17a	2.17,m	27.5	15,18,19	15,17b,19a/b
17b	1.93,m		15,16,18	15,17a,19a/b
18a	1.91,m	22.6	15,17	15,18b,19a/b
18b	1.82,m		15,17,19	15,18a,19a/b
19a	3.41.m	44.7	11,17,18	17a/b,18a/b,19b
19b	3.35,m		11,17.18	17a/b,18a/b,19a
2’	7.15,m	125.2	3’,4’,9’,12’	1’-NH,11’,12’a/b
3’		109.9		
4’		123.5		
5’	7.37,d,(7.9)	117.3	3,3’,7’,9’	11
6’		121.0,121,1		
7’		156.5,157.5		
8’	7.16,m	98.5,98.6	4’,6’,7’	1’-NH
9’		134.6,134.7		
11’	4.27,m	54.9	3’,12’,13’,16’	2’,10’-NH,12’a/b,15’
12’a	3.12,m	25.5	2’,3’,11’,13’	2’,10’-NH,11’,12’b
12’b	2.99,m		2’,3’,11’,13’	2’,10’-NH,11’,12’a
13’		165.4		
15’	4.06,m	58.5	16’,17’,18’	10’-NH,17’a/b,18’a/b
16’		169.1		
17’a	1.99,m	27.7	15’,18’,19’	15’,17’b,18’a,19’b
17’b	1.40,m		15’,16’,18’	15’,17’a,18’a/b,19’a
18’a	1.69,m	21.9	17’,19’	15’,17’a/b,18’b,19’b
18’b	1.59,m		15’	15’,17’a/b,18’b,19’b
19a’	3.31,m	44.6	13’,15’,17’,18’	17’a/b,18’b,19’b
19’b	3.22,m		15’,17’,18’	15’,17’a/b,18’b,19’a
1-NH	7.03,s		2,3,4,9	2,8
1’-NH	10.94,d,(2.3)		3’,4’,9’	2’,8’
10’-NH	7.80,s		11’,12’,13’,15’,16’	11’,12’a/b

Supplementary Table 36 NMR data of compound NAS-28 in DMSO-d6

no.	δ_H	δ_C	^{1}H-^{13}C HMBC	ROESY
2	6.02 s	79.9	3,4,9,11,12,6’	1-NH,12a,5’
3		58.8		
4		129.7		
5	7.33,dd,(8.0,1.9)	126.9	3,7,9	6
6	6.73,dt,(7.9,2.0)	117.3	4,7,8	5
7		133.1		
8	6,62,m	108.7	4,6,7,9	1-NH
9		151.6		
11	4.33,m	59.7	12,13	
12a	3.14,m	40.6	3,4,11,13,7’	11,12b,5’
12b	2.79,m		2,3,4	12a
13		166.0		
15	4.33,m	59.7	16,17	17a/b,18b
16		165.3		
17a	2.17,m	27.4	19	15,18a,19b
17b	1.91,m		15,16,18	15,17a,19b
18a	1.91,m	22.5	15,17,19	15,17a,19b
18b	1.82,m			15,19b
19a	3.43,m	44.7	15,17,18	17b
19b	3.38,m			19a
2’	7.20,d,(2.3)	125.9	3’,4’,9’,12’	1’-NH,11’,12’a/b
3’		110.0		
4’		125.9		
5’	7.44,d,(1.8)	119.5	3,4’,7’,9’	2,12b,11’,12’a/b
6’		127.7		
7’		125.1		
8’	7.46,m	114.0	4’,6’,7’	1’-NH
9’		135.2		
11’	4.29,m	54.7	3’,12’,13’,16’	2’,10’-NH,12’a/b
12’a	3.09,dd,(15.0,4.8)	25.3	2’,3’,4’,11’,13’	10’-NH,11’,12’b
12’b	2.93,m			10’-NH,11’,12’a
13’		165.3		
15’	4.07,m	58.4	16’	17’a/b,18’a/b,19’b
16’		169.1		
17’a	2.00,m	27.6	15’,16’,18’	15’,17’b,18’a/b
17’b	1.43,m		18’,19’	15’,17’a,18’a/b,19’a
18’a	1.71,m	22.0	17’,19’	15’,17’a/b,18’b,19’a
18’b	1.61,m			15’,17’a/b,18’a,19’a
19’a	3.31,m	44.6	13’,15’,18’,19’	
19’b	3.23,m		18’,19’	
1-NH	7.04,s		2,3,4,9	2,8
1’-NH	11.00,d,(2.4)		3’,4’,9’	2’,8’
10’-NH	7.80,s		11’,13’,15’,16’	11’,12’a/b

Supplementary Table 37 NMR data of compound NAS-29 in DMSO-d6

no.	δ_H	δ_C	^{1}H-^{13}C HMBC	ROESY
2	5.70,s	81.5	4,9,11,6’	1-NH,12b,5’,7’
3		58.4		
4		133.1		
5	7.13,d,(7.6)	123.9	3,7,9	
6	6.58,t,(7.4)	118.1	4,8	
7	6.96,t,(7.6)	128.1	5,9	
8	6.62,m	109.1	4,6	
9		149.5		
11	4.00,m	58.2	12,13	
12a	3.08,m	41.1	2,4,11	
12b	2.58,t,(12.1)		4,11,13,6’	
13		169.1		
15	3.99,m	59.6	16,17,18,19	
16		165.2		
17	2.36,m	28.6	15,16,18,19	
18	0.99,d,(7.1)	18.1	15,17,19	
19	0.85,d,(6.9)	16.5	15,17,18	
2’	7.14,d,(2.3)	125.1	3’,4’,9’	
3’		109.4		
4’		127.1		
5’	7.61,s	114.6	3,3’,7’,9’	2,12a/b
6’		134.6		
7’	7.04,d,(8.5)	119.2	3,5’,9’	2
8’	7.25,d,(8.5)	111.6	4’,6’	
9’		134.9		
11’	4.32,m	55.3	3’,12’,13’	
12’a	3.21,m	26.1	2’,3’,4’,11’,13’	
12’b	3.08,m		2’,3’,4’,11’,13’	
13’		165.5		
15’	4.02,m	58.5	16’,17’	
16’		168.9		
17’a	1.93,m	27.7	19’	
17’b	1.44,m			
18’a	1.65,m	21.8		
18’b	1.48,m			
19’a	3.35,m	44.6	15’,17’,18’	
19’b	3.20,m		17’,18’	
1-NH	6.63,m		3,4,9	
14-NH	7.99,s		11,13,15,16,17	
1’-NH	10.83,d,(2.4)		2’,3’,4’,9’	
10’-NH	7.75,s		11’,12’,13’,15’,16’	

Supplementary Table 38 NMR data of compound NAS-30 in DMSO-d6

no.	δ_H	δ_C	**^{1}H-^{13}C HMBC**	**ROESY**
2	5.64,s	81.5	4,9,11,6’	1-NH,12b,5’,7’
3		58.3		
4		133.0		
5	7.11,d,(6.9)	123.9	3,7,9	
6	6.58,td,(7.4,1.1)	117.9	4,8	
7	6.96,td,(7.5,1.3)	128.0	5,9	
8	6.62,d,(7.8)	108.9	4,6	
9		149.6		
11	4.00,m	58.1	13	14-NH,12a
12a	2.98,m	41.1	2,4	
12b	2.60,m		4,11	
13		168.9		
15	4.00,m	59.6	16,17,19	
16		165.0		
17	2.35,m	28.7	16,18,19	
18	0.99,d,(7.2)	18.0	15,17,19	
19	0.85,d,(6.9)	16.4	15,17,18	
2’	7.06,d,(2.3)	125.3	3’,4’,9’	
3’		108.7		
4’		126.9		
5’	7.51,d,(1.8)	114.1	3,3’,7’,9’	2,12a/b,7’
6’		134.6		
7’	7.00,dd,(8.5,1.8)	119.5	11,5’,9’	2,12a/b
8’	7.25,d,(8.5)	111.7	4’,6’	
9’		134.8		
11’	3.97,m	57.6	3’,12’,13’	
12’a	3.21,m	29.6	2’,3’,4’,11’	
12’b	3.00,m		2’,3’,4’,11’	
13’		165.4		
15’	2.60,m	57.1		
16’		168.3		
17’a	1.79,m	28.5	19’	
17’b	1.49,m		15’,16’	
18’a	1.63,m	21.3		
18’b	1.17,m			
19’a	3.24,m	44.4	13’	
19’b	2.82,m		13’	
1-NH	6.65,m		3,4,9	
14-NH	7.98,s		11,13,15,16	
1’-NH	10.90,d,(2.4)		2’,4’,9’	
10’-NH	8.16,s		13’,15’	

Supplementary Methods

General materials and methods

Escherichia coli and *Streptomyces* strains were cultivated and manipulated according to the standard methods[5,6]. Strains and plasmids used in this study are listed in Supplementary Table 7. *S.* sp. CMB-MQ030 and *S. lividens* were cultivated on the MS agar (agar 2 g, mannitol 2 g, soya flour 2 g in 100 mL tap water) for sporulation or tryptic soy broth (TSB) for growth. DNA isolation and manipulation in *E. coli* were followed with standard methods[5]. Primer synthesis and DNA sequencing were performed at Genewiz Biotech Co., Ltd. (China). Restriction enzymes and DNA polymerases (Taq and PrimeSTAR) were purchased from Takara Biotechnology Co., Ltd. (China). All chemicals and reagents were purchased from Santa Cruz Biotechnology, Inc. (USA) or Shanghai Sangon Biotech (China) Co., Ltd. unless noted otherwise. Orfs were identified using the FramePlot 4.0 beta program (http://nocardia.nih.go.jp/fp4/) and BLAST methods (http://blast.ncbi.nlm.nih.gov/Blast.cgi).

Heterologous expression of the NAS gene cluster

Primers 5'- AATTTTCATATGGCCTTGCTGTCCCACTCCTCTCC - 3' and 5' - TTTGAATTCCTGCCGGCCTCCACACATACG - 3' (restriction sites of *Nde*I and *EcoR*I are underlined), 5'- AATTTTCATATGCGTCCACAACCGAGCAGAGCC-3' and 5'- TTTGAATTCGTCCTCGGCTATCACAACCCG -3' (restriction sites of *Nde*I and *EcoR*I are underlined) were used to amplify Cluster1-*cdps-p450* and Cluster2-*cdps-p450* respectively from the *S*. sp. CMB-MQ030. The resulting DNA fragments were cloned into pMD18-T. After confirmed by DNA sequencing, the two fragments were released by *Nde*I-*Eco*RI digestion, subsequently transferred into the *Nde*I-*Eco*RI site of pIB139 to generate pWHU2482 and pWHU2483. Then these plasmids and the pIB139 (serves as a control) were transformed into *E. coli* ET12567/pUZ8002 and conjugated into *S. albus* J1074 , *S. lividans* TK24 and *S. coelicolor* M1154 to individually generate the recombinant strains mWHU2475-2483 following the standard conjugation procedure[6]. Briefly, the donor strain, *E. coli* ET12567/pUZ8002 harboring the plasmid, was prepared by growth to an OD_{600} of 0.4 - 0.6, and then the cell was washed twice and resuspended in LB medium with a concentration of ca. 5×10^9 cells per mL. Spores of the recipient *Streptomyces* were heat-shocked for 10 min at 50 °C and then resuspended in 500 μL TSB for a 3 h incubation at 37 °C. They were further resuspended in LB medium at a concentration of ca. 1×10^9 as the recipient. Each 100 μL donor and 100 μL recipient were mixed

and spread on mannitol soya flour (MS) medium plates and grown for 15 - 18 h at 30 °C. The plates were washed with sterilized water and then covered with antibiotics (50 μg mL^{-1} apramycin and 50 μg mL^{-1} nalidixic acid). The plates were incubated at 30 °C until exconjugants appear. Colonies of exconjugants were selected into TSB medium with 50 μg mL^{-1} apramycin. Their integration into the genome of recipient strain was confirmed by PCR using the same primers for cloning Cluster1-*cdps-p450* and Cluster2-*cdps-p450*.

Fermentation and metabolites profile analysis.

Wide type and recombinant strains were cultured in media MS agar for 7 days. After fermentation, the MS agar was extracted with the equal volume ethyl acetate under sonication for 20 min. Organic phase was transferred and dried by vacuum at low temperature. Metabolites were subsequently re-dissolved by 1 mL methanol and filtrated by a 0.45 μm membrane to remove particles before HPLC or HPLC-MS analysis. HPLC analysis was carried out on a SHIMADZU LC-20A Prominence HPLC system. HPLC-MS analysis was carried out on a Thermo Instruments HPLC system connected to LCQ Fleet electrospray ionization (ESI) mass spectrometer (ThermoFisher Scientific Inc.). HPLC-ESI-high resolution MS (HPLC-ESI-HRMS) analysis was carried out on ESI-LTQ Orbitrap (ThermoFisher Scientific Inc.). HPLC and HPLC-MS analysis of metabolites were performed on a column of Diamonsil (C18, 5 μm, 250 × 4.6 mm, Dikma Technologies Inc.) at a flow rate of 1 mL min^{-1} and a PDA detector under 254 nm, over a 40 min gradient program with water (eluent A) and acetonitrile (eluent B): T = 0 min, 5% B; T = 30min, 100% B; T = 33 min, 100% B; T = 34 min, 5% B; T= 40 min, 5% B.

Cloning, expression and purification of P450 and associated proteins

The DNA sequences of NascB (cluster1-p450) and cluster2-*p450* gene were optimized based on *E. coli*-bias and synthesized (GENEWIZ Co. Ltd.). The two DNA fragments were released by *Nde*I-*Xho*I digestion, subsequently transferred into the same sites of pET28a to generate pWHU2484 and pWHU2485. These resulted plasmids were transferred into *E. coli* BL21 (DE3) for protein expression. After expression at 16 °C and 220 rpm for 20 h under 100 μM isopropyl-*β*-D-thiogalactopyranoside (IPTG) induction (IPTG was added when OD_{600} reaches ~0.6), cells of 500 mL culture were then harvested by centrifugation at 2000 g at 4 °C. The cell pellet was resuspended in 20 mL lysis buffer (25 mM HEPES, pH 7.5, 300 mM NaCl, 5 mM imidazole, 10% glycerol). The cells were lysed by ultrasonication, and the insoluble debris was removed by centrifugation 1 h at 10,000 g, 4°C. The

protein supernatant was then incubated with 1 mL Ni-NTA sepharose for 1.5 hours with slow, constant rotation at 4 °C. Subsequently, the protein resin mixtures were loaded into a gravity flow column, and proteins were eluted with increasing concentrations of imidazole (25 mM, 50 mM, 100 mM, 300 mM) in Buffer A (25 mM HEPES, pH 7.5, 300 mM NaCl, 10% glycerol). Purified proteins were then loaded into PD-10 desalting columns and desalted using buffer B (25 mM HEPES, pH 7.5, 50 mM NaCl, 10% glycerol) and concentrated by centrifugation using an Amicon Ultra-4 (GE Healthcare). The purified proteins were evaluated by 12% acrylamide SDS-PAGE (Supplementary Fig. 11). We didn't obtain the soluble protein of Cluster2-P450. We obtained the soluble protein of cluster1-p450. Proteins concentration was determined by the Bradford method using a BSA calibration curve. The purified proteins were stored at -80 °C and used for in vitro assays.

The sequences of the ferredoxin/ferredoxin reductase from Spinach were optimized based on *E. coli* codon-bias and synthesized (GENEWIZ Co. Ltd.; sequences see below). These genes were tried in many expression ways and the best results are infused with TRX- (thioredoxin) and MBP- tag respectively. For constructing the expression vector, synthetic ferredoxin gene fragment was released by *Eco*RI-*Hin*dIII digestion, and cloned into the same sites of pSJ5 to generate the plasmid pTRX-Fd. Similarly, the synthetic ferrodoxin reductase gene fragment was released by *Eco*RI-*Hin*dIII digestion, and cloned into the same sites of pSJ8 to generate the plasmid pMBP-FdR. These resulted plasmids were transferred into *E. coli* BL21 (DE3) for protein expression at 25 °C. Following the similar procedure, these proteins were expressed, purified and used for in vitro assays (12% acrylamide SDS-PAGE analysis, Supplementary Fig. 11).

Synthetic Spinach ferrodoxin sequence (restriction sites are underlined):
GAATTCGCCGCCTACAAGGTGACCCTGGTGACCCCGACCGGCAACGTGGAGTT
CCAGTGCCCGGACGACGTGTACATCCTGGACGCCGCAGAAGAAGAGGGGATCG
ACCTGCCGTACAGCTGTCGCGCAGGCAGTTGCAGCAGCTGCGCAGGTAAGCTG
AAAACCGGCAGCCTGAACCAGGACGACCAGAGCTTCCTGGACGACGACCAGAT
CGACGAGGGCTGGGTTCTGACATGCGCCGCCTACCCGGTGAGCGATGTGACCA
TCGAGACCCACAAAGAAGAGGAGCTGACCGCCTAAAAGCTT

Synthetic Spinach ferrodoxin reductase sequence (restriction sites are underlined):
GAATTCCAGATCGCCTCTGATGTGGAGGCACCTCCACCTGCTCCTGCTAAGGTA
GAGAAACATTCAAAGAAAATGGAGGAAGGCATTACAGTTAACAAGTTTAAGCCTA
AGAcCCCTTACGTTGGAAGATGTCTTCTTAACACCAAAATTACTGGGGATGATGC
ACCCGGAGAGACCTGGCACATGGTTTTTTCCCATGAAGGAGAGATCCCTTACAG
AGAAGGGCAATCCGTTGGGGTTATTCCAGATGGGGAAGACAAGAATGGAAAGCC

CCATAAGTTGAGATTGTACTCGATCGCCAGCAGTGCTCTTGGTGATTTTGGTGAT
GCTAAATCTGTTTCGTTGTGTGTAAAACGACTCATCTACACCAATGACGCTGGAG
AGACGATCAAGGGAGTCTGCTCCAACTTCTTGTGTGACTTGAAACCCGGTGCTG
AAGTGAAGTTAACAGGACCAGTTGGAAAGGAGATGCTCATGCCCAAAGACCCTA
ACGCGACAATTATCATGCTTGGAACTGGAACTGGGATTGCTCCTTTCCGTTCATT
CTTGTGGAAGATGTTCTTCGAAAAGCATGATGATTACAAGTTTAACGGCTTGGCT
TGGCTTTTCTTGGGTGTACCCACAAGCAGTTCTCTTCTCTACAAAGAGGAATTTG
AGAAGATGAAGGAAAAGGCTCCAGACAACTTCAGGCTGGATTTTGCAGTGAGCA
GAGAGCAAACTAACGAGAAAGGGGAGAAGATGTACATTCAAACCCGAATGGCAC
AATACGCAGTTGAGCTATGGGAAATGTTGAAGAAAGATAATACTTATTTCTACATGT
GTGGTCTCAAGGGAATGGAAAAGGGAATTGACGACATTATGGtTTCATTGGCTGC
TGCAGAAGGCATTGATTGGATTGAATACAAGAGGCAGTTGAAGAAGGCAGAACA
ATGGAACGTTGAAGTCTACTAAGCTT.

Ferredoxin reductase (accession numbers: AGI90801, AGI87112, AGI88862, AGI91094) and ferredoxin (accession numbers: AGI91495, AGI88059, AGI92011) from *S. albus* J1074 were cloned into pETDuet-1 for co-expression. Briefly, saFdR1 (AGI90801), saFdR2 (AGI87112), saFdR3 (AGI88862) and saFdR4 (AGI91094) were individually amplified by primers saFdR1-F/R, saFdR2-F/R, saFdR3-F/R and saFdR4-F/R (Supplementary Table 8) from the *S. albus*. These fragments were digested by *Nde*I-*Xho*I and cloned into the same sites of pETDuet-1 to yield plasmid pETDuet-saFdR1 to pETDuet-saFdR4. saFd1 (AGI91495), saFd2 (AGI88059) and saFd3 (AGI92011) were individually amplified by primers saFd1-F/R, saFd2-F/R and saFd3-F/R (Supplementary Table 8) from the *S. albus*. These fragments were further digested by *Bam*HI-*Hin*dIII and individually cloned into above plasmids pETDuet-saFdR1-4 to generate the plasmids from 12 combinations, including pETDuet-saFdR1-Fd1, pETDuet-saFdR1-Fd2, pETDuet-saFdR1-Fd3, pETDuet-saFdR2-Fd1, pETDuet-saFdR2-Fd2, pETDuet-saFdR2-Fd3, pETDuet-saFdR3-Fd1, pETDuet-saFdR3-Fd2, pETDuet-saFdR3-Fd3, pETDuet-saFdR4-Fd1, pETDuet-saFdR4-Fd2, pETDuet-saFdR4-Fd3. These expression plasmids were further used for whole-cell biocatalysis.

Computational Methods

The calculations were performed using unrestricted density functional theory (UDFT) with the hybrid functional B3LYP[7-9] as implemented in the Jaguar 8.8 package[10]. Geometry optimizations were carried out with the LACVP**+ basis set[11]. Based on the optimized geometries, more accurate energies were obtained by performing single point calculations with a larger basis set, i.e., cc-pVTZ(-f)+ for all the atoms. Since the

reactions take place inside enzymes, therefore, the effects of the biological protein environment on the calculated energies were estimated by performing single point calculations on the optimized structures using the self-consistent reaction field (SCRF) method with a Poisson-Boltzmann solver[12,13] and a dielectric constant (ε) of 4 which is a standard value that has been used in the modeling of enzymes[14-16]. By means of the Gaussian 09 program package[17], frequency calculations were performed at the same theory level as the optimizations to confirm the nature of the stationary points and to obtain entropy effects. Dispersion effects were calculated using the empirical formula by Grimme et al. (i.e., DFT-D3)[18-21]. The energies reported in the paper are the free energies corrected for solvation, dispersion, and entropy effects.

Construction of GB05-dir-T7

Primers T7-F (5'-ATGACCATGATTACGGATTCACTGGCCGTCGTGGCCCGCTCCGGATTTACTAACTGGAAGAGGC-3') and T7-R (5'-GCACTCCACCGCTGATGACATGTATATCTCCTTTTACGCGAACGCGAAGTCCGAC-3') were used to amplify the T7 RNA Polymerase gene fragment from *E.coli* BL21 (DE3). Primers Am-F (5'-GTCGGACTTCGCGTTCGCGTAAAAGGAGATATACATGTCATCAGCGGTGGAGTGC-3') and Am-R (5'-GCCATCAAAAATAATTCGCGTCTGGCCTTCCTGTAGCCATCAGCCAATCGACTGGCGAGCGGC-3') were used to amplify the *aac (3)IV* gene fragment coding aminoglycoside 3-N-acetyltransferase (Am) from plasmid pIB139. Then their forward primer T7-F and reverse primer Am-R were used to overlapping PCR to generate the T7-Am fusion fragment. The overlap fragment was transferred into *E. coli* GB05-dir for PCR-targeting to incorporate the T7-Am cassette into the *lacZ.* Primers T7-For and Am-Rev were used to screen out the correct mutant strain which was named as GB05-dir-T7.

Construction of the whole cell catalysis system

In order to co-express NascB with partner proteins of *Spinach*, NascB gene fragment was released by *Nde*I-*Xho*I digestion from pWHU2484, and subsequently transferred into the *Nde*I-*Xho*I site of pET21a to generate pWHU2486. Because pTRX-Fd contains internal *Nde*I and *Nco*I sites in the multi-cloning site which are required to be moved before cloning into pRSF-Duet. Primer pairs of 5'-TTTTTTCCATGGGAAGCGATAAAATTATTCACGTG-3', 5'-GCCAGAACCAGAACCGGCCAGG-3' and 5'-TGGCCGGTTCTGGTTCTGGCTCCCACCATCACCATCACCA-3', 5'-TTTTTTAAGCTTTTAGGCGGTCAGCTCCTC-3' (*Nco*I and *Hin*dIII sites are underlined) were used to amplify the TRX tag and Fd fragments from the pTRX-Fd respectively. These two fragments were fused by overlapping PCR using their

forward and reverse primers. This TRX-Fd fragment was further digested by *Nco*I-*Hin*dIII and cloned into the same site of pRSF-Duet. Similarly, MBP-FdR fragment was released by *Nde*I-*Xho*I digestion from the plasmid pMBP-FdR and subsequently transferred into the *Nde*I-*Xho*I site of above pRSF-Duet-TRX-Fd plasmid to generate pWHU2487. Both of pWHU2486 and pWHU2487 were transferred into *E. coli* GB05-dir-T7 and other strains for biocatalysis.

In order to co-express NascB with partner proteins of *S. albus*. NascB was released by *Nde*I-*Xho*I digestion from pWHU2484, and cloned into the same sites of pRSF-Duet to generate pWHU2488. This plasmid together with each plasmid containing Fd-FdR from *S. albus* was transferred into *E. coli* GB05-dir-T7. And total 12 recombinant strains were obtained for biocatalysis.

In order to co-express glucose dehydrogenase (GDH) with NascB, NascB gene fragment was released by *Nde*I-*Xho*I digestion from pWHU2484, and subsequently transferred into the *Nde*I-*Xho*I site of pET-Duet. GDH (accession number: ABL29718) was synthesized and cloned into *Eco*RI-*Hin*dIII sites of above pET-Duet-NascB plasmid to generate pWHU2489. Both of pWHU2487 and pWHU2489 were transferred into *E. coli* GB05-dir-T7 for biocatalysis.

Scaled biocatalytic reaction and purification of compounds NAS1-30

Recombinant strain was inoculated in LB medium (5 L) by growth to an OD_{600} of 0.8-1.0 at 37 °C. After expression at 18 °C 220 rpm for 20 h under 100 μM isopropyl-*β*-D-thiogalactopyranoside (IPTG), 400 μM δ-aminolevulinic acid (ALA) and 200 μM $(NH_4)_2Fe(SO_4)2{\cdot}6H_2O$ induction (they were added when OD_{600} reaches 0.8-1.0), cells of 5 L culture were harvested by centrifugation at 2,000 g at 4 °C and were washed twice and resuspended in 200 mL M9 medium. Then 100 mM cyclodipeptide substrates (1 mL) were added in the M9 medium. After 48 h incubation at 18 °C, the reaction mixture was extracted with 400 mL of ethyl acetate three times. Organic phase was transferred and dried by vacuum at low temperature. Metabolites were subsequently redissolved by methanol with the right amount of DMSO and filtrated by a 0.45 μm membrane to remove particles. Compounds **NAS-1-30** were semi-prepared on a SHIMADZU LC-20A Prominence HPLC system using Venusil MP C18(2) (5 μm, 250 × 10 mm, Agela Technologies Inc.) at a flow rate of 3 mL min^{-1}.

Synthesis and Identification of cyclodipeptide substrate

Reagents were purchased from commercial suppliers and used without further purification unless otherwise noted. NMR spectra were recorded on Bruker Avance 400 MHz spectrometer. Chemical shifts values are given in ppm and referred to the

internal standard of TMS (tetramethylsilane). The peak patterns are indicated as follows: s, singlet; d, doublet; t, triplet; q, quadruplet; m, multiplet and dd, doublet of doublets. The coupling constants (J) are reported in hertz (Hz). Silica gel column chromatography was performed over silica gel 200−300 mesh, and the eluent was a mixture of ethyl acetate and petroleum ether, or a mixture of Methanol and dichloromethane.

Compounds ***s1-s11*** were synthesized according to known literature procedures[22] .

Cyclo-L-Trp-L-Gly (s1) Prepared from L-tryptophan methyl ester hydrochloride and N-Boc-L-Leu according to General Procedure (I) on 0.23 mmol scale to afford ***s1*** as a white solid (74.8 mg, 86% yield). ^{1}H NMR (400 MHz, DMSO): 10.97 (s, 1H), 8.11 (d, J = 34.6 Hz, 1H), 7.81 (s, 1H), 7.56 (d, J = 7.8 Hz, 1H), 7.32 (t, J = 12.9 Hz, 1H), 7.08 (s, 1H), 7.05 (d, J = 7.8 Hz, 1H), 6.97 (t, J = 7.3 Hz, 1H), 4.03 (s, 1H), 3.31 (d, J = 18.5 Hz, 1H), 3.29 – 3.22 (m, 1H), 3.02 (dd, J = 14.4, 4.1 Hz, 1H), 2.78 (d, J = 17.2 Hz, 1H).

Cyclo-L-Trp-L-Ala (s2) Prepared from L-tryptophan methyl ester hydrochloride and N-Boc-L-Leu according to General Procedure (I) on 0.23 mmol scale to afford ***s2*** as a white solid (56.3 mg, 82% yield). ^{1}H NMR (400 MHz, DMSO): 10.72 (s, 1H), 7.92 – 7.77 (m, 1H), 7.73 (t, J = 14.0 Hz, 1H), 7.47 – 7.22 (m, 1H), 7.06 (t, J = 11.9 Hz, 1H), 6.81 (d, J = 2.2 Hz, 1H), 6.80 – 6.77 (m, 1H), 6.73 – 6.68 (m, 1H), 3.88 (s, 1H), 3.35 (q, J = 6.7 Hz, 1H), 3.01 (dd, J = 14.4, 4.0 Hz, 1H), 2.77 (dd, J = 14.4, 4.5 Hz, 1H), 0.15 (t, J = 7.0 Hz, 1H). ^{13}C NMR (100MHz, DMSO): 167.70, 166.72, 135.75, 127.76, 124.55, 120.76, 118.95, 118.34, 111.08, 108.40, 55.37, 49.74, 28.81, 19.53.

Cyclo-L-Trp-L-Val (s3) Prepared from L-tryptophan methyl ester hydrochloride and N-Boc-L-Leu according to General Procedure (I) on 0.23 mmol scale to afford ***s3*** as a white solid (58 mg, 84% yield). ^{1}H NMR (400 MHz, DMSO): 10.87 (d, J = 1.4 Hz, 1H), 8.08 (t, J = 14.9 Hz, 1H), 7.90 (dd, J = 21.0, 11.6 Hz, 1H), 7.61 (d, J = 7.9 Hz, 1H), 7.27 (dd, J = 19.3, 4.7 Hz, 1H), 7.06 (d, J = 2.3 Hz, 1H), 7.05 – 6.99 (m, 1H), 6.96 – 6.91 (m, 1H), 6.91 – 6.90 (m, 1H), 4.22 – 4.06 (m, 1H), 3.57 – 3.45 (m, 1H), 3.26 (dd, J = 14.4, 4.4 Hz, 1H), 3.03 (dt, J = 16.6, 8.3 Hz, 1H), 0.49 (d, J = 7.1 Hz, 1H), 0.45 (dd, J = 10.5, 4.8 Hz, 1H). 13C NMR (100MHz, DMSO): 167.15, 166.20, 135.95, 127.97, 124.58, 120.58, 118.96, 118.16, 110.92, 108.71, 58.84, 55.14, 28.39, 22.77, 14.40, 11.60.

Cyclo-L-Trp-L-Pro (s4) Prepared from L-tryptophan methyl ester hydrochloride and N-Boc-L-Leu according to General Procedure (I) on 0.23 mmol scale to afford ***s4*** as a white solid (58.3 mg, 85% yield). ^{1}HNMR (400 MHz, $CDCl_3$): 8.38 (s, 1H), 7.54 (dd, J = 8.1, 1.3 Hz, 1H), 7.33-7.24 (m, 1H), 7.12 (ddd, J = 8.2, 7.0, 1.2 Hz, 1H), 7.05 (ddd, 1H), 6.95 (d, 1H), 6.24 (d, 1H), 4.16 (dt, 1H), 3.47 (m, 1H), 3.34 (dd, 1H), 3.10 (m, 2H), 2.73 (dd, 1H), 2.00 (m, 1H), 1.77(m, 1H), 1.60(m, 1H), 1.36 (m, 1H).

Cyclo-L-Trp-L-Phe (s5) Prepared from L-tryptophan methyl ester hydrochloride and N-Boc-L-Leu according to General Procedure (I) on 0.23 mmol scale to afford ***s5*** as a white solid (56 mg, 80% yield). ^{1}H NMR (400 MHz, DMSO): 10.95 (d, J = 1.5 Hz, 1H), 7.99 (d, J = 2.3 Hz, 1H), 7.78 (d, J = 2.4 Hz, 1H), 7.52 (d, J = 7.9 Hz, 1H), 7.34 (t, J = 9.4 Hz, 1H), 7.22 – 7.16 (m, 3H), 7.13 – 7.07 (m, 1H), 7.04 – 7.01 (m, 1H), 6.99 (d, J = 2.2 Hz, 1H), 6.72 (d, J = 2.0 Hz, 1H), 6.70 (d, J = 1.4 Hz, 1H), 4.01 (s, 1H), 3.88 (t, J = 6.8 Hz, 1H), 2.83 (dd, J = 14.4, 4.3 Hz, 1H), 2.57 (d, J = 5.8 Hz, 1H), 2.54 – 2.48 (m, 1H), 2.46 (d, J = 4.6 Hz, 1H), 1.84 (dd, J = 13.4, 7.1 Hz, 1H).

Cyclo-L-Trp-L-Leu (s6) Prepared from L-tryptophan methyl ester hydrochloride and N-Boc-L-Leu according to General Procedure (I) on 0.23 mmol scale to afford ***s6*** as a white solid (58.3 mg, 85% yield). ^{1}H NMR (400 MHz, DMSO): 10.96 (s,1H), 8.12 (s,1H), 7.98 (s,1H), 7.55 (d, *J*=7.8 Hz, 1H), 7.29 (d, *J*= 8.1 Hz, 1H), 7.02 (t,1H), 6.91 (t,1H), 4.09 (m,1H), 3.26 (dd, *J*= 14.4, 3.7 Hz, 1H), 2.97 (dd, *J*=14.3, 9.6 Hz, 1H), 1.17 (m,1H), 0.88 (m,1H), 0.61 (m,2H), 0.51 (d, *J*= 6.7 Hz, 3H), 0.40 (d, *J*= 6.6 Hz, 3H).

Cyclo-L-Trp-L-Ile (s7) Prepared from L-tryptophan methyl ester hydrochloride and N-Boc-L-Ile according to *General Procedure (I)* on 0.23 mmol scale to afford ***s7*** as a white solid (56.3 mg, 82% yield). ^{1}H NMR (400 MHz, DMSO): 10.85 (s, 1H), 8.02 (s, 1H), 7.89 (s, 1H), 7.60 (d, J = 7.9 Hz, 1H), 7.28 (d, J = 8.0 Hz, 1H), 7.07 (d, J = 2.3 Hz, 1H), 7.05 – 6.98 (m, 1H), 6.97 – 6.90 (m, 1H), 4.17 – 4.11 (m, 1H), 3.53 – 3.45 (m, 1H), 3.21 (dd, J = 14.4, 4.9 Hz, 1H), 3.06 (dd, J = 14.4, 4.5 Hz, 1H), 1.70 – 1.57 (m, 1H), 0.59 (d, J = 7.1 Hz, 3H), 0.14 (d, J = 6.8 Hz, 3H).

Cyclo-L-Trp-L-Met (s8) Prepared from L-tryptophan methyl ester hydrochloride and N-Boc-L-Met according to *General Procedure (I)* on 0.23 mmol scale to afford ***s8*** as a white solid (62.9 mg, 85% yield). ^{1}HNMR (400 MHz, DMSO): 10.92 (s,1H), 8.22 (s,1H), 8.01 (s,1H), 7.61 (d, *J* = 8.1 Hz, 1H), 7.30 (d, *J* = 7.9 Hz, 1H), 7.03 (m,2H), 6.93 (m,1H), 4.15 (s,1H), 3.68 (t,1H), 3.29 (dd, *J* = 14.3, 3.6 Hz, 1H), 2.99 (dd, *J* = 14.3, 4.6

Hz, 1H), 1.74 (s,3H), 1.61 (m, 2H), 1.21 (m,1H), 0.92 (m,1H); ^{13}C NMR (100MHz, DMSO) δ 167.43, 166.83, 136.29, 128.28, 125.13, 121.14, 119.49, 118.77, 111.71, 108.86, 55.82, 53.45, 33.17, 31.17, 29.23, 28.08.

Cyclo-L-Trp-L-Trp (s9) Prepared from L-tryptophan methyl ester hydrochloride and N-Boc-L-Tryptophan according to *General Procedure (I)* on 0.23 mmol scale to afford ***s9*** as a white solid (74.8 mg, 86% yield). ^{1}H-NMR(400MHz, DMSO) : 10.87 (s,2H), 7.75 (s,2H), 7.33 (d, *J* = 7.8 Hz, 2H), 7.31 (d, *J* = 8.0 Hz, 2H), 7.04 (t, 2H), 6.98 (t, 2H), 6.61 (s,2H), 3.89 (s,2H), 2.72 (dd, *J* =14.2, 3.8 Hz, 2H), 2.20 (dd, *J* =14.2, 6.6 Hz,2H); ^{13}C-NMR (100 MHz, DMSO): 167.28, 136.51, 127.81, 124.84, 121.36, 118.62, 111.66, 109.20, 55.61, 30.32.

Cyclo-L-Trp-L-Gln (s10) Prepared from L-tryptophan methyl ester hydrochloride and N-Boc-L-Gln according to *General Procedure (I)* on 0.23 mmol scale to afford ***s10*** as a white solid (57.3 mg, 78% yield). ^{1}H-NMR (400 MHz, DMSO) : 10.84 (s,1H), 7.99 (s,1H), 7.88 (s,1H), 7.48 (d, *J* = 7.9 Hz, 1H), 7.21 (d, *J* = 8.0 Hz,1H), 6.99 (d, *J* = 2.3 Hz,1H), 6.94 (t,1H), 6.86 (t,1H), 6.69 (s,1H), 6.61 (s,1H), 4.02 (brs,1H), 3.54 (brs, 1H), 3.11 (dd, *J* = 14.4, 4.7 Hz, 1H), 2.96 (dd, *J* = 14.5, 4.5 Hz,1H), 1.40 (m,2H), 1.25 (m,1H), 0.95 (m,1H); ^{13}C-NMR (100 MHz, DMSO): 173.49, 167.29, 166.79, 135.79, 127.65, 124.52, 120.65, 113.76, 113.26, 111.04, 108.65, 55.13, 53.19, 29.63, 29.66, 28.97.

Cyclo-L-Trp-L-Asn (s11) Prepared from L-tryptophan methyl ester hydrochloride and N-Boc-L-Asn according to *General Procedure (I)* on 0.23 mmol scale to afford ***s11*** as a white solid (56 mg, 80% yield). ^{1}H-NMR (400 MHz, DMSO): 10.93 (s,1H), 7.97 (s,1H), 7.69 (s,1H), 7.57 (d, *J* = 7.9 Hz,1H), 7.34 (d, *J* = 7.9 Hz,1H), 7.12 (s,1H), 7.05 (t,1H), 6.96 (d, *J* = 7.8 Hz,1H), 6.92 (m,2H), 4.13 (brs,1H), 3.99 (brs,1H), 3.20 (dd, *J* = 14.5, 5.5 Hz, 1H), 3.10 (dd, *J* = 14.5, 4.4 Hz, 1H), 2.16 (dd, *J* = 15.6, 4.4 Hz, 1H), 1.47 (dd, *J* = 15.6, 8.3 Hz,1H); ^{13}C-NMR (100 MHz, DMSO): 171.80, 167.82, 167.33, 136.34, 128.12, 124.86, 121.19, 119.23, 118.74, 111.53, 109.09, 55.61, 51.70, 38.36, 29.04.

Compounds ***s12-s16*** *&* ***s21-s30*** were synthesized as below: To a solution of amino acid methyl ester hydrochloride (1.0 equiv) in CH_2Cl_2 (0.1 M) at 0 °C was added Et_3N (4.5 equiv) dropwise. HOBt•H_2O (1.5 equiv) and N-Boc-L-tryptophan (1.5 equiv) were sequentially added and stirred vigorously. Once homogenous, EDC•HCl (1.5 equiv) was added in a single portion and the solution allowed to warm to 23 °C. The reaction was stirred for 15 hours, at which time it was quenched by the addition of 1N HCl, and the aqueous layer extracted with CH_2Cl_2 (2 x). The combined organics were then

washed with saturated aqueous $NaHCO_3$, and the aqueous layer back extracted with CH_2Cl_2 (2 x). The organics were pooled, then dried over Na_2SO_4, filtered, and concentrated in vacuo. The resulting oil/ foam was subsequently dissolved in CH_2Cl_2 (0.2 M), and cooled to 0 °C. TFA (1.5 mL / 5 mL CH_2Cl_2) was added dropwise, then the solution was warmed to 23°C and stirred for 2 h. The mixture was concentrated in vacuo and the resulting viscous residue dissolved in methanol (0.25 M), and cooled to 0 °C. Ammonium hydroxide (28–30% in H_2O, 1 mL / 6 mL MeOH) was then added dropwise and the reaction mixture allowed to warm to 23 °C and stirred for 24 h. The resulting suspension was cooled to 0 °C, and the fine white precipitate was filtered and rinsed with cold methanol.

Cyclo-L-Trp-L-Ser (s12) Prepared from L-Serine methyl ester hydrochloride (0.32 mmol) and N-Boc-L-tryptophan according to afford ***s12*** as a white solid (68.7 mg, 78% yield). ^{1}H NMR (400 MHz, DMSO): 10.94 (s,1H), 7.93 (m,2H), 7.53 (d, J = 7.9 Hz, 1H), 7.32 (d, J = 8.1 Hz,1H), 7.11 (d, J = 2.3 Hz,1H), 7.04 (m,1H), 6.96 (m,1H), 4.98 (t,1H), 4.00 (t,1H), 3.69 (m,1H), 3.30 (m,1H), 3.17 (t,2H), 3.02 (m,1H); ^{13}C NMR (100 MHz, DMSO) δ 167.27, 165.76, 135.96, 127.59, 124.14, 120.77, 118.57, 118.29, 111.21, 109.05, 62.93, 57.24, 55.47, 48.56, 30.38.

Cyclo-L-Trp-L-Thr (s13) Prepared from L-Thr methyl ester hydrochloride (50 mg) and N-Boc-L-tryptophan to afford ***s13*** as a white solid (70.5 mg, 83% yield). ^{1}H NMR (400 MHz, DMSO): 10.91 (s,1H), 8.08 (s,1H), 7.82 (s,1H), 7.54 (d, 1H), 7.33 (d, J = 8.0 Hz, 1H), 7.16 (d, J = 2.3 Hz,1H), 7.06 (m,1H), 6.96 (m,1H), 5.04 (d, J = 5.9 Hz,1H), 3.91 (m,1H), 3.76 (m,1H), 3.52 (t,1H), 3.19 (m,2H), 0.94 (d, J = 6.6 Hz,3H); ^{13}C NMR (100 MHz, DMSO): 167.89, 166.46, 136.13, 127.59, 123.99, 120.74, 118.50, 118.23, 111.22, 109.65, 67.13, 60.35, 55.75, 31.17, 19.65.

Cyclo-L-Trp-L-Cys (s14) Prepared from L-Cysteine methyl ester hydrochloride (50 mg) and N-Boc-L-tryptophan to afford ***s14*** as a white solid (68.2 mg, 81% yield). ^{1}H-NMR(400 MHz, DMSO) : 10.94 (s,1H), 8.28 (s,1H), 7.99 (s,1H), 7.53 (d, J = 7.9 Hz,1H), 7.28 (d, J = 8.1 Hz,1H), 7.00 (m,3H), 6.94 (t,1H), 4.14 (brs,1H), 3.71 (brs,1H), 3.24 (dd, J = 19.8, 5.4 Hz, 1H), 3.04 (dd, J = 19.8, 5.4 Hz, 1H), 2.22 (dd, J = 13.4, 3.7 Hz, 1H), 1.21 (m, 1H); ^{13}C-NMR (100 MHz, DMSO):167.07, 166.35, 135.12, 127.85, 124.83, 121.42, 119.26, 118.93, 111.41, 109.00, 56.03, 53.31, 41.92, 29.38.

Cyclo-L-Trp-L-Tyr (s15) Prepared from L-Tyr methyl ester hydrochloride (50 mg) and N-Boc-L-tryptophan to afford ***s15*** as a white solid (66.5 mg, 88% yield). ^{1}H-NMR (400 MHz, DMSO+D2O): 7.48 (d, J = 7.9 Hz,1H), 7.32 (d, J = 8.1 Hz,1H), 7.07 (t,1H), 6.98 (t,2H), 6.57 (d, J = 8.3 Hz,2H), 6.44 (d, J = 8.3 Hz, 2H), 3.97 (t,1H), 3.78 (t,1H), 2.80 (dd, J = 14.4, 4.1 Hz, 1H), 2.42 (m,2H), 1.58 (m,1H); ^{13}C-NMR (100 MHz,

DMSO+D2O): 167.55, 167.08, 156.10, 136.51, 130.89(2C), 127.72, 126.63, 124.80, 121.54, 119.19, 119.09, 115.31, 111.81, 108.98, 56.13, 55.52, 30.17, 28.76.

Cyclo-L-Trp-L-His (s16) Prepared from Methyl L-Histidinate dihydrochloride (50 mg) and N-Boc-L-tryptophan to afford ***s16*** as a white solid (30.7 mg, 46% yield). ^{1}H-NMR(400 MHz, DMSO) :11.93(s,1H), 9.52(s,1H), 9.15(s,1H), 8.69(s,1H) , 8.41(d, J = 7.8 Hz,1H), 8.20(d, J = 7.9 Hz 1H), 7.94(m, 2H), 7.83 (t,1H), 6.57(s,1H), 4.96(s,1H), 4.58(m,1H), 4.10(s,1H), 4.08(s,1H), 3.81(d, J = 14.2 Hz,1H), 3.12(d, J = 14.2 Hz, 1H),1.75(t,1H); ^{13}C-NMR(100 MHz, DMSO):168.40, 136.94, 135.17, 129.32, 129.06, 125.27, 122.18, 120.62, 119.89, 118.07, 112.69, 109.86, 56.73, 54.20, 30.74, 30.17.

Cyclo-7-F-L-Trp-L-Pro (s21) Prepared from L-Pro methyl ester hydrochloride (50 mg) and N-Boc-6-F-L-tryptophan afford ***s21*** as a white solid (79.1 mg, 87% yield). ^{1}H-NMR (400 MHz, DMSO): 11.25 (s,1H), 7.92 (s,1H), 7.58 (d, J = 7.8 Hz, 1H), 7,27 (s,1H), 7.14 (d, J = 7.4 Hz,1H), 6.99(t,1H), 4.34 (s,1H), 4.06 (t,1H), 3.35 (m,1H), 3.25 (m,1H), 3.15 (m,2H), 1.99 (s,1H), 1.66 (m,2H), 1.40 (m,1H); ^{13}C-NMR (100 MHz, DMSO):169.05, 165.36, 132.64, 129.42, 125.74, 120.33, 119.24, 117.90, 115.60, 110.77, 58.38, 55.13, 44.57, 27.70, 25.63, 21.83.

Cyclo-5-Cl-L-Trp-L-Pro (s22) Prepared from L-Pro methyl ester hydrochloride (50 mg) and N-Boc-4-Cl-L-tryptophan to afford ***s22*** as a white solid (76.5 mg, 80% yield). ^{1}H-NMR (400 MHz, DMSO): 11.28 (s,1H), 7.61 (s,1H), 7.39 (s,1H), 7.34 (d, J = 7.9 Hz,1H), 7.03 (m,2H), 4.37 (m,1H), 4.23 (t,1H), 3.79 (dd, J = 15.3, 3.9 Hz, 1H), 2.96 (dd, J = 15.3, 8.4 Hz,1H), 2.09 (m,1H), 1.92 (m,1H), 1.87 (m,1H); ^{13}C-NMR(100 MHz, DMSO):170.14, 166.88, 137.90, 126.18, 124.53, 123.5, 121.75, 119.24, 170.77, 109.90, 58.56, 55.36, 44.85, 27.31, 25.71, 22.52.

Cyclo-6-Cl-L-Trp-L-Pro (s23) Prepared from L-Pro methyl ester hydrochloride (50 mg) and N-Boc-5-Cl-L-tryptophan to afford ***s23*** as a white solid (77.5 mg, 81% yield). ^{1}H-NMR(400 MHz, MeOD): 7.49 (s,1H), 7.20 (d, J = 8.6 Hz,1H), 7.05 (s,1H), 6.95 (d, J = 10.5 Hz,1H), 4.53 (s,1H), 4.29 (s,1H), 3.88 (m,1H), 3.37 (m,1H), 3.24 (m,1H), 3.12 (dd, J = 14.8, 4.4 Hz, 1H), 1.87 (m,1H), 1.80 (m,1H), 1.49 (m,1H); ^{13}C-NMR(100 MHz, MeOD): 170.80, 167.30, 136.41, 130.07, 127.44, 125.72, 122.69, 119.09, 113.64, 110.35, 60.20, 57.20, 45.85, 29.07, 28.81, 22.11.

Cyclo-7-Cl-L-Trp-L-Pro (s24) Prepared from L-Pro methyl ester hydrochloride (50 mg) and N-Boc-6-Cl-L-tryptophan to afford ***s24*** as a white solid (81.4 mg, 85% yield). ^{1}H-NMR (400 MHz, DMSO): 11.01 (s,1H), 7.89 (s,1H), 7.60 (d, J = 8.6 Hz,1H), 7.37

(s,1H), 7.21 (s,1H), 7.01 (m,1H), 4.31 (s,1H), 4.06 (t,1H), 3.42 (s,0.5H), 3.36 (s,0.5H), 3.24 (m,1H), 3.16 (d, J = 14.8Hz,1H), 3.10 (d, J = 9.7Hz,1H), 1.97 (m,1H), 1.66 (m,1H), 1.33 (m,1H); ^{13}C-NMR(100 MHz, DMSO): 169.38, 166.81, 136.74, 126.74, 126.06, 120.67, 119.01, 111.26, 110.13, 58.86, 55.70, 45.03, 28.24, 26.21, 22.27.

Cyclo-8-Cl-L-Trp-L-Pro (s25) Prepared from L-Pro methyl ester hydrochloride (50 mg) and N-Boc-7-Cl-L-tryptophan to afford ***s25*** as a white solid (74.7 mg, 78% yield). ^{1}H-NMR (400 MHz, DMSO) : 11.02 (s,1H), 7.90 (s,1H), 7.60 (d, J = 8.4 Hz,1H), 7.38 (s,1H), 7.21 (s,1H), 6.69 (d, J = 8.6 Hz, 1H), 4.31 (s,1H), 4.03 (s,1H), 3.18 (m,3H), 1.97 (m,1H), 1.65 (m,1H), 1.29 (m,1H); ^{13}C-NMR (100 MHz, DMSO): 168.86, 165.32, 136.24, 126.23, 125.56, 120.17, 118.51, 110.77, 109.62, 58.36, 55.20, 44.52, 27.74, 25.72, 21.76.

Cyclo-6-Br-L-Trp-L-Pro (s26) Prepared from L-Pro methyl ester hydrochloride (50mg) and N-Boc-5-Br-L-tryptophan to afford ***s26*** as a white solid (74.1 mg, 68% yield). ^{1}H-NMR(400 MHz, MeOD): 7.74 (s,1H), 7.25 (d, J = 3.9 Hz,1H), 7.17 (m,1H), 7.13 (s,1H), 4.40 (m,1H), 3.99 (m,1H), 3.50 (m,1H), 3.34 (m,1H), 3.21 (m,1H), 1.96 (m,1H), 1.69 (m,1H), 1.49 (m,1H), 1.29 (m,1H); ^{13}C-NMR (100MHz, MeOD): 170.52, 167.67, 136.50, 130.35, 127.24, 125.13, 122.58, 113.78, 113.02, 109.42, 59.64, 57.08, 45.72, 29.22, 28.47, 22.54.

Cyclo-D-Trp-D-Pro (s27) Prepared from D-Pro methyl ester hydrochloride (50 mg) and N-Boc -D-tryptophan to afford ***s27*** as a white solid (70.1 mg, 82% yield). ^{1}HNMR (400 MHz, $CDCl_3$): 8.25 (s, 1H), 7.52 (dt, 1H), 7.30 (m, 1H), 7.18 (m, 1H), 7.03 (m, 2H), 5.71 (s, 1H), 4.28 (m, 1H), 4.00 (m, 1H), 3.69 (m, 1H), 3.52 (m, 2H), 2.91 (m, 1H), 2.26 (m, 1H), 1.92 (m, 2H), 1.80 (m, 1H); ^{13}C NMR (100 MHz, CDCl3): 169.66, 165.83, 136.97, 127.02, 123.64, 123.07, 120.29, 118.80, 111.86, 110.22, 59.53, 54.89, 45.72, 28.61, 27.15, 22.92.

Cyclo-D-Trp-L-Pro (s28) Prepared from L-Pro methyl ester hydrochloride (50 mg) and N-Boc-D-tryptophan to afford ***s28*** as a white solid (65.8 mg, 77% yield). ^{1}HNMR (400 MHz, $CDCl_3$): 8.38 (s, 1H), 7.54 (dd, J = 8.1, 1.3 Hz, 1H), 7.33-7.24 (m, 1H), 7.12 (ddd, J = 8.2, 7.0, 1.2 Hz, 1H), 7.05 (ddd, 1H), 6.95 (d, 1H), 6.24 (d, 1H), 4.16 (dt, 1H), 3.47 (m, 1H), 3.34 (dd, 1H), 3.10 (m, 2H), 2.73 (dd, 1H), 2.00 (m, 1H), 1.77(m, 1H), 1.60(m, 1H), 1.36 (m, 1H); ^{13}C NMR (100 MHz, CDCl3): 169.86, 165.81, 136.48, 127.30, 124.51, 122.78, 120.16, 119.21, 111.55, 109.65, 58.67, 58.18, 45.39, 30.95, 29.25, 21.85

Cyclo-L-Trp-D-Pro (s29) Prepared from D-Pro methyl ester hydrochloride (50 mg) and N-Boc -L-tryptophan to afford ***s29*** as a white solid (64.1 mg, 75% yield). ^{1}HNMR (400 MHz, $CDCl_3$): 8.29 (s, 1H), 7.54 (ddt, 1H), 7.30 (m, 1H), 7.09 (m, 2H), 6.96 (m, 1H), 6.12 (d, 1H), 4.15 (m, 1H), 3.48 (m, 1H), 3.34 (m, 1H), 3.10 (m, 2H), 2.74 (m, 1H),

2.00 (m, 1H), 1.78 (m, 1H), 1.60 (m, 1H), 1.35 (m, 1H). ^{13}C NMR (101 MHz, CDCl3): 169.86, 165.81, 136.48, 127.30, 124.51, 122.78, 120.16, 119.21, 111.55, 109.65, 58.67, 58.18, 45.39, 30.95, 29.25, 21.85.

Oxo-Cyclo-L-Trp-L-Pro (Oxo-cW$_L$-P$_L$. s30) Prepared from L-Pro methyl ester hydrochloride (50 mg) and 3-(benzofuran-3-yl)-2-((tert-butoxycarbonyl)amino)propanoic acid (which was synthesized by using the known procedures[23]) to afford ***s30*** as a white solid (74.9 mg, 87% yield). ^{1}H-NMR(400 MHz, MeOD): 7.66(s,1 H), 7.62(m,1H), 7.53(m,1H), 7.37(t,1H), 7.29(t,1H), 4.29(t,1H), 3.55(m, 1H), 3.38 (m,1H), 3.18(m,2H), 3.06(m,1H), 2.08(m,1H), 1.89(s,1H), 1.68(m,1H), 1.52(m,1H); ^{13}C NMR (100 MHz, MeOD) 169.57, 166.03, 155.32, 143.97, 127.29, 124.18, 122.50, 119.18, 114.45, 110.85, 57.88, 57.12, 44.77, 28.37, 27.40, 21.22.

Procedure For the Synthesis of Compound Cyclo-L-Trp-L-Lys (s17). Prepared from L-tryptophan methyl ester hydrochloride (50 mg) and N$^{\alpha}$-Boc-N$^{\varepsilon}$-Cbz-L-Lysine according to General Procedure[22] to obtain Cyclo-L-Trp- N$^{\varepsilon}$-Cbz-L-Lys, which was then dissolved in MeOH and Pd-C (10%) was added to the solution. The reaction mixturewas stirred at room temperature for 30 min under H_2. After which Pd-C was removed by filtration, and the solution was evaporated to get the white solid product (18 mg, 29% yield). ^{1}H-NMR (400 MHz, MeOD): 7.65 (d,1H), 7.35 (d,1H), 7.11 (t,2H), 7.02 (t,1H), 4.34 (m,1H), 3.66 (m,1H), 3.52 (dd, 1H), 3.12 (dd,1H), 1.96 (s,1H), 1.16 (m,2H), 0.94 (m,1H), 0.60 (m,1H), 0.43 (m,2H); ^{13}C-NMR(100 MHz, MeOD): 168.38, 168.28, 136.36, 128.06, 124.06, 121.04, 118.78, 118.70, 110.73, 107.72, 55.98, 54.15, 38.81, 32.49, 28.86, 26.55,20.42.

Procedure For the Synthesis of Compound Cyclo-L-Trp-L-Arg(s18). Prepared from L-tryptophan methyl ester hydrochloride (50 mg) and N$^{\alpha}$-Boc-N$^{\omega}$-Nitro-L-Arg according to the procedure that synthesis compound ***17***, and got the white solid compound ***s18*** (13.4 mg, 20% yield). ^{1}H-NMR(400 MHz, MeOD): 7.56 (d, J = 7.9 Hz, 1H), 7.29 (d, J = 8.1 Hz, 1H), 7.01 (m,2H), 6.95 (t,1H), 4.24 (t,1H), 3.61 (t,1H), 3.42 (m,1H), 3.08 (m,1H), 2.55 (t,2H), 0.78 (m,2H), 0.63 (m,1H), 0.33 (m,1H); ^{13}C-NMR (100 MHz, MeOD): 169.12, 167.84, 157.06, 136.44, 127.72, 124.42, 121.15, 118.84, 110.85, 108.04, 56.06, , 53.80, 40.31, 30.67, 29.07, 23.14, 22.95.

Procedure For the Synthesis of Compound Cyclo-L-Trp-L-Asp(s19) & Cyclo-L-Trp-L-Glu(s20). To a solution of L-tryptophan methyl ester hydrochloride (50 mg, 1.0 equiv) in CH_2Cl_2 (0.1 M) at 0 °C was added Et_3N (4.5 equiv) dropwise. HOBt•H_2O (1.5 equiv) and N-Boc-L-Asp-OBzl (1.5 equiv) were sequentially added and stirred vigorously. Once homogenous, EDC•HCl (1.5 equiv) was added in a single portion and the solution allowed to warm to 23°C. The reaction was stirred for 15 hours, at which time it was quenched by the addition of 1N HCl, and the aqueous

layer extracted with CH_2Cl_2 (2 x). The combined organics were then washed with saturated aqueous $NaHCO_3$, and the aqueous layer back extracted with CH_2Cl_2 (2 x). The organics were pooled, then dried over Na_2SO_4, filtered, and concentrated in vacuo. The resulting oil/foam was subsequently dissolved in CH_2Cl_2 (0.2 M), and cooled to 0 °C. TFA (1.5 mL / 5 mL CH_2Cl_2) was added dropwise, then the solution was warmed to 23 °C and stirred for 2 h. The mixture was concentrated in vacuo and the resulting viscous residue dissolved in methanol (0.25 M), and cooled to 0 °C. The residue was dissolved in 0.1 M acetic acid-2-butanol, and one equivalent N-methyl morpholine (NMM) was added to the solution. The reaction mixture was refluxed for 3 h under N_2. The reaction mixture was concentrated in vacuo to a small volume, and the residue was purified by flash chromatography on silica gel (eluent: DCM/MeOH, 40:1) to afford Cyclo-L-Trp-L-Asp-OBzl. In the following steps, Cyclo-L-Trp-L-Asp-OBzl was dissolved in MeOH and Pd-C (10%) was added to the solution. The reaction mixture was stirred at room temperature for 30 min under H_2. After which Pd-C was removed by filtration, and the solution was evaporated to get the white solid product (14.2 mg, 24% yield). ^{1}H-NMR (400 MHz, MeOD): 7.58 (d, *J* = 8.0 Hz,1H), 7.36 (d, *J* = 8.1 Hz, 1H), 7.09 (t,2H), 7.00 (t,1H), 4.29 (t,1H), 4.03 (m,1H), 3.46 (dd, *J* = 14.6, 4.3 Hz, 1H), 3.17 (dd, *J* = 14.6, 4.3 Hz,1H), 2.10(dd, *J* = 17.4, 3.3 Hz,1H), 0.92(dd, *J* = 17.4, 9.4 Hz,1H); ^{13}C-NMR (100 MHz, MeOD): 172.65, 168.39, 167.08, 136.10, 127.64, 124.48, 121.02, 118.95, 118.08, 110.96, 107.96, 55.91, 51.34, 37.37, 29.43.

Cyclo-L-Trp-L-Glu(s20) Prepared from L-tryptophan methyl ester hydrochloride (50 mg) and N-Boc-L-Glu-OBzl according to the procedure that synthesis compound ***s19***, and got the white solid compound ***s20*** (15.5 mg, 25% yield). ^{1}H-NMR (400 MHz, MeOD) : 7.26 (d, *J* = 7.9 Hz,1H), 7.34 (d, *J* = 8.1 Hz,1H), 7.11 (s,1H), 7.08 (d,1H), 7.01 (t,1H), 4.27 (t,1H), 3.76 (m,1H), 3.42 (dd,1H), 3.21 (dd,1H), 1.79 (m,1H), 1.69 (m,1H), 1.47 (m,1H), 0.64 (M,1H); ^{13}C-NMR (100 MHz, MeOD): 172.27, 168.43, 166.25, 136.44, 137.63,124.16,121.09,118.67,118.34, 110.89, 108.05, 55.93, 54.05, 32.64, 30.72, 29.56.

General methods for analyzing the structures of NASs

Through analysis of the 1D and 2D NMR data, we confirmed the way that how two DKP subunits connected. Here, two representative compounds **NAS-17** and **NAS-18**, which were all composed of two molecules of cW_L-V_L, were took as examples to illustrate the way of thinking. **NAS-17** had a C^3-$C^{7'}$ linkage between the two subunits, HMBC correlations from two aromatic protons, H-6' and H-8' and the indole NH proton to a quaternary carbon at δ_C126.6 (C-4') indicated that the indole was substituted at

C-7’ (Supplementary Fig. 12). In addition, HMBC correlations from H-6’ and H-8’ to δ_C 58.5 (C-3) confirmed this assignment. **NAS-18** had a C^3-$C^{6'}$ linkage between the two subunits, HMBC correlations from two aromatic protons, H-5’ and H-7’ and the indole NH proton to a quaternary carbon at δ_C134.9 (C-9’) indicated that the indole was substituted at C-6’.In addition, HMBC correlations from H-5’ and H-7’ to δ_C 58.4 (C-3) confirmed this assignment.

Correlations observed in a ROESY experiment (Supplementary Fig. 12) were used to assign the relative configuration of the C2 and C3 stereogenic centres in the products. In **NAS-17**, ROESY correlations between H-2 and the aromatic protons H-6’ and H-7’ clearly showed that H-2 and the upper cyclic dipeptide were *cis* to each other. The H-11 resonance at showed ROESY correlations to H-2 indicating that these two protons were on the same face; In **NAS-18**, ROESY correlations between H-2 and the aromatic protons H-5’ and H-7’ clearly showed that H-2 and the upper cyclic dipeptide were *cis* to each other. However, the H-11 resonance at showed no ROESY correlations to H-2 indicating that these two protons were on the different face. With the assistance of the known configuration of H-11, we can confirm that the relative configurations of C2 and C3 in **NAS-17** and **NAS-18** were (***2R, 3S***) and (***2S, 3R***) respectively.

Bioactivity assay

To investigate the protective effects of compounds **NAS-1-28** against glutamate-induced PC-12 cells apoptosis, PC12 cells were dealt with glutamate to establish cell models. The cell viability was measured by [3-(4,5-dimethylthiazol-2-yl)-2,5-diphenyltetrazolium bromide] (MTT) assay. PC-12 cells were seeded at a density of 1 × 10^4 per well (160 μL) in 96-well plates and incubated 24 h, and each compound (20 μL, 10 μM) was added to four wells separately. At the same time, we set the blank group, control group (15 mM L-Glu), positive drug group (Nimodipine 20 μM). After 1 h treatment, L-Glu (20 μL,15 mM) was added to each well. After 24 h treatment, remove the supernatant, add 10% MTT new medium and incubate at 37°C for 4 h. The viability was determined using an MTT kit according to the manufacturer’s instructions. The inhibition rate was calculated by the following formula: Inhibition rate %=(OD of the blank group - OD of test group)/ (OD of the blank group) *100 (see Supplementary Table 6).

To observe the effect of compounds on PC12 cell injury induced by $A\beta_{25\text{-}35}$ (A4559), PC12 cells were treated with $A\beta_{25\text{-}35}$ to build an AD cell model. MTT method was applied in the measurement of cell viability. The PC-12 cells in the logarithmic growth phase were digested, centrifuged and resuspended in complete medium at a density

of 5 × 10^4 mL^{-1}, which were seeded in 96-well plates (8 × 10^3 / well, 160 μL). After 24 h incubation, remove the old medium, add the new medium (160 μL) and the compounds (20 μL, 10 μM) to per well. After 1 h treatment, $A\beta_{25-35}$ (20 μL, 30 μM) was added to each well. After 48 h treatment, MTT (20 μL) was added to per well and incubated at 37°C for 4 h. The viability was determined using an MTT kit according to the manufacturer's instructions. The inhibition rate was calculated by the following formula: Inhibition rate %=(OD of the blank group - OD of test group)/ (OD of the blank group) *100 (see Supplementary Table 5).

Supplementary References

1. Saruwatari, T. et al. Cytochrome P450 as dimerization catalyst in diketopiperazine alkaloid biosynthesis. ChemBioChem 15, 656-659 (2014).
2. Kishimoto, S., Sato, M., Tsunematsu, Y. & Watanabe, K. Evaluation of biosynthetic pathway and engineered biosynthesis of alkaloids. Molecules 21, 1078 (2016).
3. Fu, J.et al. Full-length RecE enhances linear-linear homologous recombination and facilitates direct cloning for bioprospecting. *Nat. Biotechnol*. **30**, 440-446 (2012).
4. Sun, Y., He, X., Liang, J., Zhou, X. & Deng, Z. Analysis of functions in plasmid pHZ1358 influencing its genetic and structural stability in Streptomyces lividans 1326. *Appl. Microbiol. Biotechnol*. **82**, 303-310 (2009).
5. Sambrook, J. & Russell, D.W. Molecular Cloning: A Laboratory Manual, 3rd ed. Cold Spring Harbor Laboratory Press, New York. (2001).
6. Kieser, T., Bibb, M., Butter, M., Chater, K.F. & Hopwood, D.A. Practical Streptomyces genetics. The John Innes Foundation, Norwich. (2001)
7. Becke, A. D. A new mixing of Hartree-Fock and local density-functional theories. *J. Chem. Phys.* **98**, 1372-1377 (1993).
8. Becke, A. D. Density-functional thermochemistry. III. The role of exact exchange. *J. Chem. Phys.* **98**, 5648-5652 (1993).
9. Lee, C., Yang, W. & Parr, R. G. Development of the Colle-Salvetti correlation-energy formula into a functional of the electron density. *Phys. Rev. B* **37**, 785-789 (1988).
10. Jaguar, version 8.8, Schrodinger, LLC, New York, (2015).
11. Hay, P. J. & Wadt, W. R. Ab initio effective core potentials for molecular calculations. Potentials for K to Au including the outermost core orbitals. *J. Chem. Phys.* **82**, 299-310 (1985).
12. Tannor, D. J. *et al*. Accurate first principles calculation of molecular charge distributions and solvation energies from Ab initio quantum mechanics and continuum dielectric theory. *J. Am. Chem. Soc.* **116**, 11875-11882 (1994).
13. Marten, B. *et al.* New model for calculation of solvation free energies: correction of self-consistent reaction field continuum dielectric theory for short-range hydrogen-bonding effects. *J. Phys. Chem.* **100**, 11775-11788 (1996).
14. Blomberg, M. R., Borowski, T., Himo, F., Liao, R. -Z. & Siegbahn, P. E. M. Quantum chemical studies of mechanisms for metalloenzymes. *Chem. Rev.* **114**, 3601-3658 (2014).

15. Himo, F. & Siegbahn, P. E. M. Quantum chemical studies of radical-containing enzymes. *Chem. Rev.* **103**, 2421-2456 (2003).
16. Siegbahn, P. E. M. Mechanisms of metalloenzymes studied by quantum chemical methods. *Q. Rev. Biophys.* **36**, 91-145 (2003).
17. Frisch, M. J. *et al.* Gaussian 09, Revision D.01, Gaussian, Inc., Wallingford CT (2013).
18. Grimme, S. Accurate description of van der Waals complexes by density functional theory including empirical corrections. *J. Comput. Chem.* **25**, 1463-1473 (2004).
19. Grimme, S. Semiempirical GGA-type density functional constructed with a long-range dispersion correction. *J. Comput. Chem.* **27**, 1787-1799 (2006).
20. Grimme, S., Antony, J. Ehrlich, S. & Krieg, H. A consistent and accurate ab initio parametrization of density functional dispersion correction (DFT-D) for the 94 elements H-Pu. *J. Chem. Phys.* **132**, 154104 (2010).
21. Goerigk, L. & Grimme, S. A thorough benchmark of density functional methods for general main group thermochemistry, kinetics, and noncovalent interactions. *Phys. Chem. Chem. Phys.*__13__, 6670-6688 (2011).
22. Kieffer, M. E., Chuang, K. V. & Reisman, S. E. Copper-catalyzed diastereoselective arylation of tryptophan derivatives: total synthesis of (+)-naseseazines A and B. *J Am Chem Soc*.**135**, 5557-5560 (2013)
23. Paizs, C., Katona, A., Rétey, J. The interaction of heteroaryl-acrylates and alanines with phenylalanine ammonia-lyase from parsley. *Chem. Eur. J.* **12**, 2739-2744 (2006).
