# Supplementary material for: Efficient biosynthesis of heterodimeric C3-aryl pyrroloindoline alkaloids
Source: Nat Commun. 2018 Oct 24;9:4428. doi: 10.1038/s41467-018-06528-z (PMC6200733; doi:10.1038/s41467-018-06528-z)
Supplement: Supplementary file 3 — Description of Additional Supplementary Files [file 41467_2018_6528_MOESM3_ESM.pdf]

Description of Additional Supplementary Files

File Name: Supplementary Data 1
Description: Cartesian coordinates of optimized structures
